# Rerouting and Improving Dauc‐8‐en‐11‐ol Synthase from *Streptomyces venezuelae* to a High Yielding Biocatalyst

**DOI:** 10.1002/chem.202100962

**Published:** 2021-05-01

**Authors:** Lukas Lauterbach, Anwei Hou, Jeroen S. Dickschat

**Affiliations:** ^1^ Kekulé-Institut für Organische Chemie und Biochemie Rheinische Friedrich-Wilhelms Universität Bonn Gerhard-Domagk-Str. 1 53121 Bonn Germany

**Keywords:** biocatalyst, isotopic labelling, mutagenesis, terpene cyclisation

## Abstract

The dauc‐8‐en‐11‐ol synthase from *Streptomyces venezuelae* was investigated for its catalytic activity towards alternative terpene precursors, specifically designed to enable new cyclisation pathways. Exchange of aromatic amino acid residues at the enzyme surface by site‐directed mutagenesis led to a 4‐fold increase of the yield in preparative scale incubations, which likely results from an increased enzyme stability instead of improved enzyme kinetics.

## Graphical abstract

**Daucenol Synthase (DcS)** from *Streptomyces venezuelae* was redirected using substrate analogues with additional, shifted, or lacking methyl groups to yield molecules from a naturally difficult to access chemical space, and improved in its catalytic efficiency through site‐directed mutagenesis of surface residues, turning DcS into a high yielding biocatalyst.

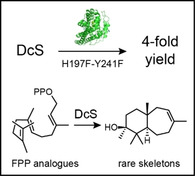

## Introduction

**Scheme 1:**
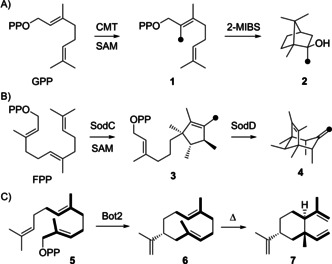
Biosynthesis of A) 2‐methylisoborneol (**2**), and B) sodorifen (**4**). Black dots indicate Me groups from methylations by SAM. C) Enzymatic conversion of FPP analogue **5** with inverted isoprene units (bold) by Bot2.

Terpenes represent one of the structurally most diverse classes of natural products,[^1^, ^2^] although they all originate basically from only two isomeric precursor molecules. Wallach's visionary proposal of a general terpene ensemble by head‐to‐tail connection of isoprene units helped tremendously in their structure elucidation especially at his time.^[3]^ The biochemical background for the biogenetic isoprene rule rests in the terpene monomers dimethylallyl (DMAPP) and isopentenyl diphosphate (IPP), reactive isoprene derivatives, that are the precursors of the oligomeric terpene precursors geranyl (GPP), farnesyl (FPP), geranylgeranyl (GGPP) and geranylfarnesyl diphosphate (GFPP) by prenyltransferases.^[4]^ Their cyclisations by terpene synthases proceed by abstraction of the diphosphate group, yielding a reactive allyl cation that can enter a cascade reaction involving typical carbocation chemistry with cyclisations by intramolecular attack of an olefin to a cationic centre, hydride or proton migrations, Wagner‐Meerwein rearrangements (WMR), eventually water quenching (in case of terpene alcohols) and terminal deprotonation. The terpene cyclisation cascades usually proceed along a series of stabilised tertiary or allylic cations, demonstrating the importance of the methyl branches in the oligoprenyl diphosphate precursors for efficient enzymatic turnover. Today more and more examples are known that escape the logic of Wallach's isoprene rule, for example by methylation of the terpene precursor prior to cyclisation. This can open new reaction pathways, because in the methylated substrates cationic charges can be stabilised at carbons whose analogous positions in canonical substrates would yield secondary cations. Two natural examples include the methylation of GPP to 2‐methyl‐GPP (**1**) followed by its cyclisation to 2‐methylisoborneol (**2**) by 2‐methylisoborneol synthase (2‐MIBS, Scheme 1A),[^5^, ^6^, ^7^] and the methylation‐induced cyclisation of FPP to pre‐sodorifen diphosphate (**3**) by SodC and further cyclisation to sodorifen (**4**) by the sodorifen cyclase SodD (Scheme 1B).[^8^, ^9^] In a recent study, Kirschning and co‐workers have converted non‐natural FPP analogues with altered methylation patterns such as **5**, leading to new reaction pathways. Using the presilphiperfolan‐8β‐ol synthase Bot2 from *Botrytis cinerea*,^[10]^ *“iso*‐germacrene A” (**6**) was obtained that can, like germacrene A, react by Cope rearrangement to *“iso*‐β‐elemene” (**7**, Scheme 1C).^[11]^

In an attempt to systematically expand this work we now report on the conversion of further FPP analogues with altered methylation pattern that were designed to change their cyclisation modes in terpene synthase catalysed reactions. Dauc‐8‐en‐11‐ol synthase from *Streptomyces venezuelae* ATCC 10712 (DcS, introduced as “isodauc‐8‐en‐11‐ol synthase, IdS” in our previous study, cf. Supporting Information Figure S1)^[12]^ was selected as a catalyst, because this enzyme has a high catalytic efficiency in the conversion of FPP. The yield in preparative scale incubations of the wildtype enzyme was increased in this study to more than 4‐fold through site‐directed mutagenesis and the newly obtained enzyme variant was used for the enzymatic preparation of non‐natural sesquiterpene analogues.

## Results and Discussion

**Scheme 2:**
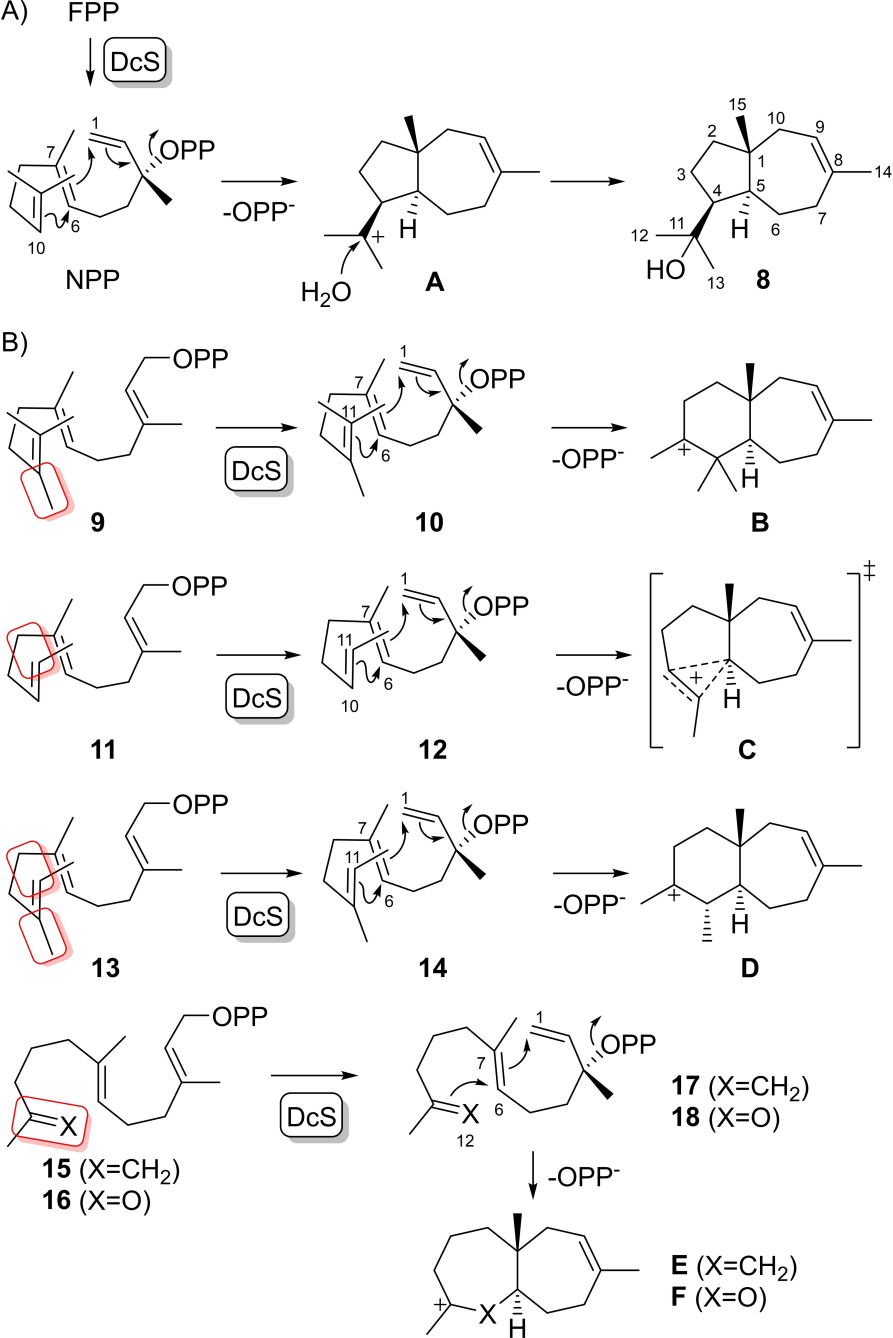
Dauc‐8‐en‐11‐ol synthase DcS. A) Cyclisation mechanism from FPP to **8**. B) Rationale for the design of substrate analogues based on hypothetical alternative cyclisation modes. Structural modifications in comparison to FPP are highlighted by red boxes.

The cyclisation mechanism towards dauc‐8‐en‐11‐ol (**8**) starts with isomerisation of FPP to nerolidyl diphosphate (NPP) and proceeds with a likely concerted 1,7‐6,10‐cyclisation to **A**, avoiding an intermediate secondary cation, followed by the addition of water (Scheme 2A). Substrate analogues with a methylation pattern different to that in FPP could lead to specifically altered cyclisation modes, e. g. 10‐methyl‐FPP (**9**) may not (only) react through the NPP analogue **10** in a 1,7‐6,10‐cyclisation, but could (also) react by 1,7‐6,11‐cyclisation to **B** in which the positive charge would be stabilised as tertiary cation (Scheme 2B). On the contrary, the situation for 13‐desmethyl‐FPP (**11**) would be less clear and thus particularly interesting to investigate, as the natural 1,7‐6,10‐ and the alternative 1,7‐6,11‐cyclisation mode for DcS catalysis would both lead to a secondary cation through collapse of the transition state shown in **C**; however, this hypothetical secondary cation may be a transient species attacked in a concerted reaction by the active site water. Also of interest is 13‐desmethyl‐10‐methyl‐FPP (**13**) for which a 1,7‐6,11‐cyclisation to the tertiary cation **D** may be preferred over the 1,7‐6,10‐cyclisation to a secondary cation. Shifting the 10,11‐double bond of FPP to an 11,12‐double bond as in **15** could allow for a 1,7‐6,12‐cyclisation to **E**, which could also be realised by ketone **16** that may cyclise to **F**.

**Scheme 3:**
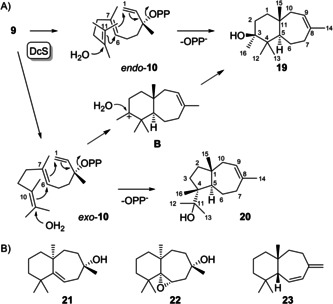
A) Cyclisation mechanism for the conversion of **9** with DcS. B) Structures of known widdranes.

To investigate these hypotheses all FPP analogues were synthesised for enzyme incubations with DcS. The conversion of **9** (for synthesis cf. Supporting Information Scheme S1, Figures S2–S4) with DcS resulted in the formation of two C_16_ alcohols (Supporting Information Figure S5). Both compounds were isolated and their structures were elucidated by NMR (Supporting Information Tables S1 and S2, Figures S6–S20) as the main product 3‐methylwiddr‐8‐en‐3‐ol (**19**) and the minor product 4‐*epi*‐4‐methyldauc‐8‐en‐11‐ol (**20**, Scheme 3A). Widdranes are rarely observed in Nature and only realised by a few representatives including widdrol (**21**) and its epoxide **22** from *Widdringtonia juniperoides* ^[13]^ and *ent*‐widdradiene (**23**) from *Cupressus macrocarpa* (Scheme 3B).^[14]^ The widdrane skeleton is likely difficult to access by FPP cyclisation as it requires transient cationic charges at the secondary carbons C6 and C10 (a type II cyclisation with protonation at C10 could be a reasonable alternative to explain widdrane biosynthesis). The installation of a methyl group at C10 as in **9** allows this reaction to proceed more efficiently towards formation of the methylated widdrane **19**. The formation of **19** can be explained from the NPP analogue **10** adopting a conformation (*endo*‐**10**) that reflects that of NPP in the cyclisation to **8**. However, the configuration at C4 of **20** is only explainable from *exo*‐**10**. It is possible that the additional methyl group increases the substrate's steric bulk which may enforce a conformational flip to *exo*‐**10** in the side reaction to **20**, while the main product **19** still follows the ordinary pathway through *endo*‐**10**. But **19** can also be explained from *exo*‐**10**, which would mean that the main and the side product follow a common reaction trajectory, only in this case a non‐concerted mechanism via the previously proposed cation **B** should be considered in which the cation can be attacked by water from the *Si* face. This face is oriented towards the C6=C7 double bond and is not directly accessible in *exo*‐**10**, thus a conformational rearrangement in **B** is required.

The absolute configurations of **19** and **20** were investigated through stereoselective deuteration, introducing artificial stereogenic centres at deuterated carbons of known configuration. Additional ^13^C‐labellings at these stereogenic centres allow for a sensitive product analysis by HSQC. Conversion of 2‐methyl‐DMAPP (**S4**, Supporting Information Scheme S1, Figures S21–S23) and (*R*)‐ or (*S*)‐(1‐^13^C,1‐^2^H)IPP^[15]^ with FPPS from *Streptomyces coelicolor* A3^[16]^ and DcS (Supporting Information Figure S24) established the absolute configurations of **19** and **20** as shown in Scheme 3, that were confirmed by analogous use of (*E*)‐ and (*Z*)‐(4‐^13^C,4‐^2^H)IPP (Supporting Information Figure S25).^[17]^

**Scheme 4:**
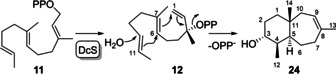
Cyclisation mechanism for the conversion of **11** with DcS.

Conversion of **11** (for synthesis cf. Supporting Information Scheme S2, Figures S26–S28) with DcS yielded one main and two side products (Supporting Information Figure S29). The major compound was isolated and its structure elucidated by NMR spectroscopy (Supporting Information Table S4, Figures S30–S37), resulting in the structure of nor‐widdr‐8‐en‐4‐ol (**24**, Scheme 4). This experiment demonstrated that the intermediate NPP analogue **12** preferentially reacts in a 1,7‐6,11‐cyclisation with formation of a 7–6 bicyclic skeleton. The absolute configuration was tentatively assigned as shown in Scheme 4 based on the assumption that **12** adopts a similar conformation as *exo*‐**10** in the cyclisation to **20**, and on its optical rotation (${\left[\alpha \right]}_{D}^{20}$ =+28.4 (c 0.12, C_6_D_6_)) that presents the same sign as for the compounds **8** (${\left[\alpha \right]}_{D}^{22}$ =+19.4 (c 0.505, C_6_D_6_)),^[12]^ **19** (${\left[\alpha \right]}_{D}^{20}$ =+194.0 (c 0.1, C_6_D_6_)), and **20** (${\left[\alpha \right]}_{D}^{20}$ =+4.8 (c 0.13, C_6_D_6_)).

**Scheme 5:**
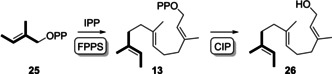
Conversion of **25** into the FPP isomer **13** and dephosphorylation with CIP to **26**.

The FPP isomer 13‐desmethyl‐10‐methyl‐FPP (**13**) can be prepared enzymatically from tiglyl diphosphate (**25**, synthesised as shown in Supporting Information Scheme S3, Figures S38–S40) and IPP through FPPS catalysis. This reaction proceeds with similarly high efficiency as for the conversion of the native substrates DMAPP and IPP into FPP, which was demonstrated by treatment of the products with calf intestinal phosphatase (CIP, Scheme 5) and GC/MS analysis of the product **26** in comparison to farnesol (Supporting Information Figure S41). Further conversion with DcS surprisingly resulted in the formation of the FPP‐derived main product **8**, besides smaller amounts of other products not observed with FPP alone (Supporting Information Figure S42). A deep investigation of this reaction demonstrated that DcS coeluted with a very small amount of *E. coli* IDI^[18]^ from the Ni^2+^‐NTA column (Supporting Information Figure S43), requiring further enzyme purification by FPLC to remove it and to suppress the conversion of IPP into **8** (Supporting Information Figure S44). After optimisation of the reaction conditions, DcS showed only a poor conversion of **13** into a complex product mixture.

**Scheme 6:**
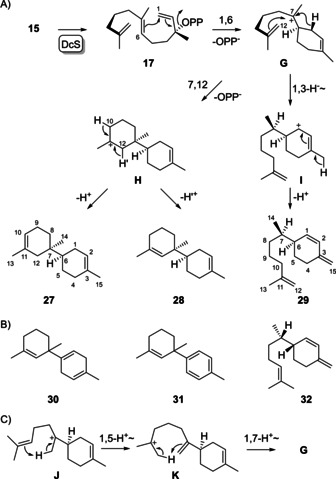
Enzymatic conversion of **15** with DcS. A) Cyclisation mechanism for the conversion of **15**. B) Known natural products that are structurally similar to **27**–**29**. C) Cyclisation mechanism suggested by Hong and Tantillo^[21]^ from the bisabolyl cation **J** to **G** as a proposed intermediate towards tenuifolene (**30**).

Compounds **15** and **16** were synthesised through a common approach (Supporting Information Schemes S4 and S5, Figures S45–S50). The incubation of **15** with DcS resulted in the formation of three hydrocarbons (**27**–**29**, Supporting Information Figure S51) which were isolated and subjected to NMR for structure elucidation (Supporting Information Tables S5–S7, Figures S52–S73). Interestingly, none of the compounds contained the two annulated seven‐membered rings as hypothesised for **E** in Scheme 2. Instead, **27** (tenuifola‐2,10‐diene) and **28** (tenuifola‐2,11‐diene) showed a rare bicyclic carbon skeleton that is only represented in Nature by the structures of tenuifolene (**30**) and *ar*‐tenuifolene (**31**), two sesquiterpenes of unknown absolute configuration from the African sandalwood *Osyris tenuifolia*,^[19]^ while **29** (*iso*‐β‐sesquiphellandrene) is similar to the widespread natural product β‐sesquiphellandrene (**32**) that was first isolated from ginger (Schemes 6A and B).^[20]^ The formation of **29** proceeds through isomerisation to the NPP isomer **17**, followed by 1,6‐cyclisation to cation **G**, a 1,3‐hydride shift to **I** and deprotonation from C15. Compounds **27** and **28** require a 1,6‐7,12‐cyclisation to the tertiary cation **H** and deprotonation from C10 or C12. Based on DFT calculations Hong and Tantillo have suggested a mechanism for the formation of natural **30** through the bisabolyl cation (**J**) involving a 1,5‐proton shift to **K**, another 1,7‐proton shift to **G** and cyclisation to **H** (Scheme 6C), the same intermediates as proposed here for the cyclisation of **15**, and final deprotonation.^[21]^ The natural products **30** and **31** then require downstream oxidations. Thus, the enzyme reaction with **15** gives access to the biosynthetic precursor of tenuifolenes.

**Scheme 7:**
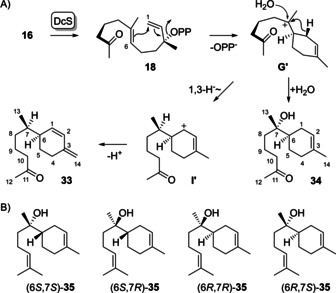
Conversion of methyl ketone **16** by DcS. A) Proposed catalytic mechanism, B) structures of all four stereoisomers of *α*‐bisabolol.

Conversion of **16** with DcS led to the isolation of two compounds (Supporting Information Figures S74 and S75). NMR‐based structure elucidation gave access to the structures of ketone **33** (Supporting Information Table S8, Figures S76–S82) and hydroxy ketone **34** (Supporting Information Table S9, Figures S83–S89), showing that the carbonyl group in **16** does not participate in cyclisations. The cyclisation of **16** proceeds by isomerisation to the NPP analogue **18**, followed by 1,6‐cyclisation to **G’** (Scheme 7A). A subsequent 1,3‐hydride shift to **I’** and deprotonation yield **33**, while the attack of water to **G’** leads to **34**. This alcohol is structurally similar to α‐bisabolol for which all four stereoisomers are known from Nature (Scheme 7B). The stereoisomer (6*S*,7*S*)‐**35** occurs in chamomile oil,[^22^, ^23^] (6*S*,7*R*)‐**35** (“anymol”) is known from *Myoporum crassifolium*,^[24]^ (6*R*,7*R*)‐**35** occurs in *Populus balsamifera* ^[24]^ and (6*R*,7*S*)‐**35** in *Rosa rugosa*.^[25]^

Assignment of the relative and absolute configurations of the obtained compounds **27–29**, **33** and **34** proved to be a particular problem, because in all five compounds the neighbouring stereogenic centres at C6 and C7 are connected by a single bond around which free rotation is possible, which hampers NOE based assignments. Assuming *R* configuration for the NPP analogues **17** and **18**, and a similar conformational fold with C1 in the backside, C6=C7 in the middle and C11=C12 in the front, as required for the natural intermediate (*R*)‐NPP to explain the stereochemistry of **8** (Scheme 2A), the stereostructures as shown in Schemes 6 and 7 should be expected.

**Scheme 8:**
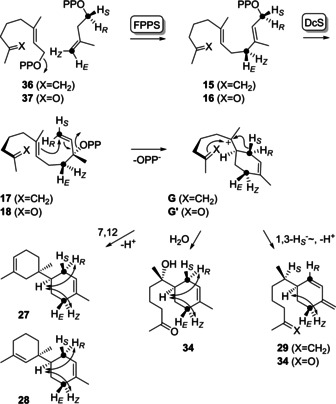
Determination of the configuration at C6 of **27**–**29**, **33** and **34** by stereoselective labelling experiments.

To address this problem experimentally, the GPP analogues **36** and **37** were synthesised (Supporting Information Scheme S6, Figures S90–S95) and incubated with FPPS, DcS and (*R*)‐ or (*S*)‐(1‐^13^C,1‐^2^H)IPP, leading to stereoselectively deuterated and ^13^C‐labelled FPP analogues **15** and **16** (Scheme 8). The *syn*‐allylic transposition of diphosphate results in stereospecifically labelled **17** and **18**, followed by *anti*‐S_N_2’ attack at C1 in the cyclisation to **G** and **G’** that exhibit a stereogenic centre at deuterated C1 of known configuration.^[26]^ Analysis of the products by HSQC spectroscopy (Supporting Information Figures S96–S100) in conjunction with the NOESY interaction between the 1‐*pro*‐*R* hydrogen and H6 revealed 6*R* configuration for **27**, **28** and **34**. For **29** and **33** the specific migration of the 1‐*pro*‐*S* hydrogen to C7 was observed which is in line with 6*R* configuration also for **29** and 6*S* configuration for **33** (this is the same stereochemistry at C6, but there is a change in the priorities of substituents). Similar experiments with (*E*)‐ and (*Z*)‐(4‐^13^C,4‐^2^H)IPP confirmed all assignments for the stereochemistry at C6 (Supporting Information Figures S101–S105).

Comparison of the ^13^C‐NMR data of **29** and **33** to those reported for synthetic *ent*‐**32** and 6‐*epi*‐**32** ^[27]^ suggested that both compounds **29** and **33** have 7*R* configuration (Supporting Information Figure S106), leading to the assignment of the structures of (6*S*,7*R*)‐**29** and (6*S*,7*R*)‐**33**. A similar comparison of the ^13^C‐NMR data of **34** to those of (6*S*,7*S*)‐**35** and (6*S*,7*R*)‐**35** ^[28]^ suggested the structure of (6*R*,7*S*)‐**34**. This result was confirmed by a synthetic transformation of (6*S*,7*S*)‐**35** into (6*S*,7*S*)‐**34** (Supporting Information Scheme S7) that showed different NMR data to those of **34** obtained from **16** with DcS (Supporting Information Figures S107–S113). For compounds **27** and **28** based on the assumed substrate fold in the DcS active site 7*R* configuration is tentatively assigned, leading to (6*R*,7*R*)‐**27** and (6*R*,7*R*)‐**28**. Comparison of the optical rotation of **29** (${\left[\alpha \right]}_{D}^{20}$ =+3.3, *c* 0.03, CH_2_Cl_2_) and **33** (${\left[\alpha \right]}_{D}^{20}$ =+21.4, *c* 0.21, CH_2_Cl_2_) to reported data for **32** (${\left[\alpha \right]}_{D}^{29}$ =−3.99, in substance;^[20]^ [*α*]_D_=−7.48, *c* 0.82, CHCl_3_)^[29]^ were in line with their pseudoenantiomeric relationship. Along similar lines, the optical rotation of **34** (${\left[\alpha \right]}_{D}^{20}$ =+51.7, *c* 0.12, CH_2_Cl_2_) in comparison to that of (6*R*,7*S*)‐**35** (${\left[\alpha \right]}_{D}^{23}$ =+63, *c* 0.29, MeOH)^[25]^ confirmed their stereochemically coinciding skeletons.

**Figure 1:**
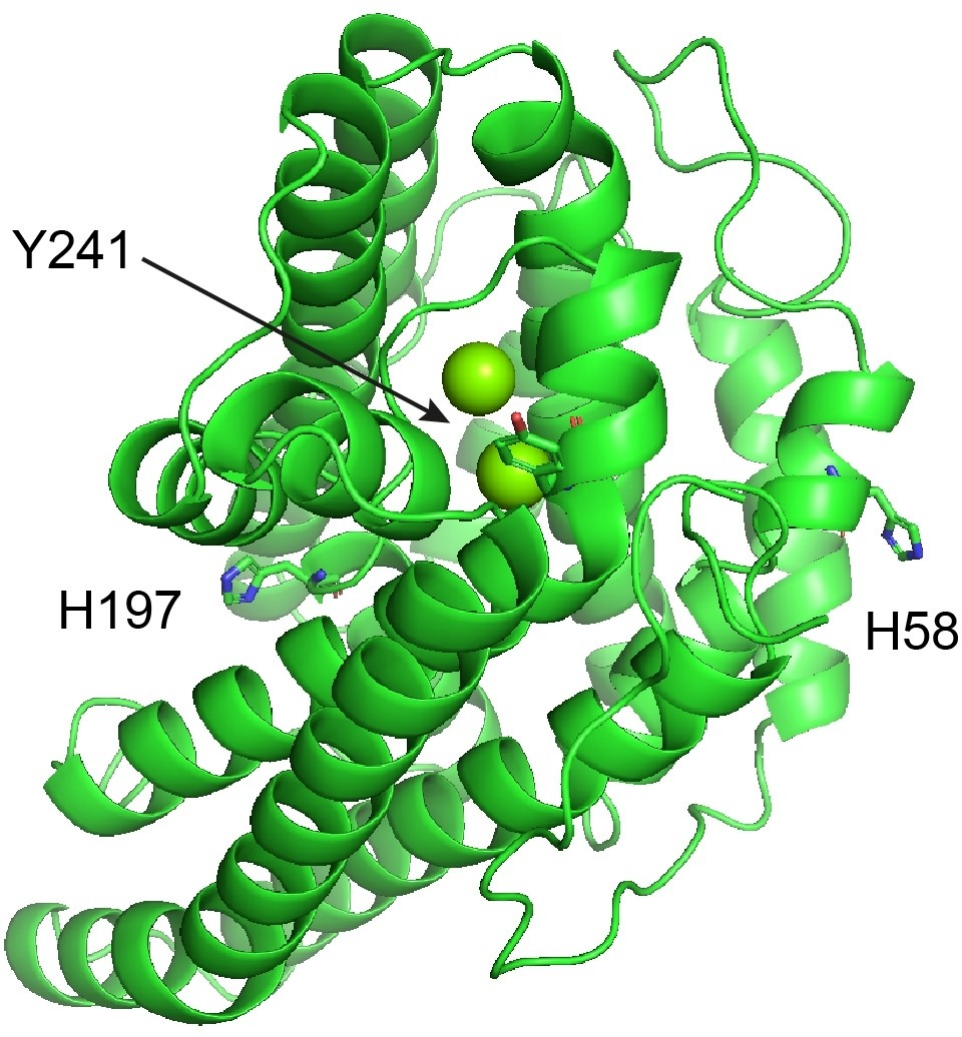
DcS homology model generated by Swiss modelling. Amino acid residues mutated in this study are highlighted.

It is well known that bacterial type I terpene synthases contain several highly conserved motifs that are important for catalytic activity. This includes the aspartate‐rich motif DDXX(X)D,^[30]^ for DcS with a missing third Asp (^92^DDYFA), the pyrophosphate sensor ^190^R,^[31]^ the NSE triad ^236^NDVASYERE, and the RY pair (Supporting Information Figure S114).^[32]^ These motifs are involved in binding of the Mg^2+^ cofactor and in substrate recognition. In addition, active site aromatic residues stabilise cationic intermediates along the terpene cyclisation cascade,^[33]^ and their mutation often causes adverse effects.[^31^, ^34^, ^35^, ^36^] Enzyme modelling of DcS using the Swiss Model server returned the best quality model based on the N‐terminal domain of geosmin synthase from *Streptomyces coelicolor* complexed with three Mg^2+^ and alendronate as template (PDB: 5DZ2).^[37]^ The model shows that the aromatic residues, H58, H197 and Y241 are most likely located at the surface of the protein (Figure 1).

**Figure 2:**
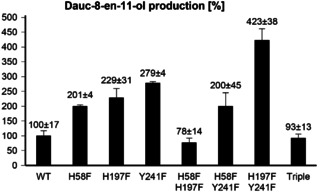
Production of **8** by WT DcS and its enzyme variants obtained by site‐directed mutagenesis. The bars indicate average relative amounts of **8** from triplicates analysed by GC/MS.

In order to expand previous mutational work, these residues were selected for site‐directed mutagenesis, yielding the H58F, H197F and Y241F enzyme variants as soluble proteins (Supporting Information Figure S115). Their catalytic activity in the conversion of FPP was investigated in small scale incubation experiments, showing a 2‐ to 3‐fold production of **8** compared to wildtype (WT) DcS in all three cases (Figure 2). This serendipitous finding prompted us to investigate whether the catalytic activity can be further increased for any of the three possible combinations of double mutations or for the triple mutant. While for the H58F/H197F enzyme variant and the triple mutant the activity was in the range of WT DcS and the H58F/Y241F variant did not show any positive effect in comparison to the single mutants, the H197F/Y241F double mutant exhibited the 4‐fold production of **8**. Large scale incubations of FPP (80 mg) with H197F/Y241F increased the isolated yield of **8** from 11 % to 46 %, showing that the higher catalytic efficiency is preparatively very useful. Also the incubation of **9** with H197F/Y241F increased the yields for **19** from 4 % to 11 % and for **20** from 15 % to 42 %.

Kinetic measurements comparing the WT with the H197F/Y241F mutant revealed an increase of the turnover rate *k*_cat_ from 35.6±2.9 s^−1^ to 65.5±8.6 s^−1^, but also an increased *K*_m_ from 2.90±0.02 mm to 5.95±0.02 mm, and thus only minor differences regarding *k*_cat_/*K*_M_ (1.2×10^4^ s^−1^ m ^−1^ for the WT and 1.1×10^4^ s^−1^ m ^−1^ for H197F/Y241F, Supporting Information Table S11, Figures S116 and S117). The wildtype enzyme showed substrate inhibition at FPP concentrations above 1 mm, but the H197/Y241F tolerated slightly higher substrate concentrations up to 2 mm and can convert the substrate at a higher rate. This is one explanation for the higher product formation in the large scale experiments, performed with a substrate concentration of 2 mm, at which the mutant performs significantly better than the WT.

Enzyme stability issues may give a second explanation. It is well know that the mutations on protein surfaces can have a significant influence on protein stability.^[38]^ This has also been shown for a terpene synthase, tobacco 5‐*epi*‐aristolochene synthase, for which the thermal protein stability was significantly improved through site‐directed mutagenesis of surface residues,^[39]^ but this enzyme variant showed a moderately reduced catalytic performance in comparison to the wildtype. For the DcS H197F/Y241F variant the increased enzyme stability may be responsible for the higher yield in preparative scale reactions in comparison to the wildtype. These reactions are usually carried out over reaction times of 10–12 h in our laboratory, often leading to enzyme denaturation and precipitation during the course of the incubation.

## Conclusion

In summary we have shown that DcS accepts non‐natural FPP analogues that were specifically designed to open new reaction trajectories. 10‐Methyl‐FPP not only gave access to the methylated daucenol **20**, but also to the methylated widdrenol **19**, similar to the findings for 13‐desmethyl‐FPP, which resulted in the nor‐widdrenol **21**. The FPP isomer **15** gave access to tenuifola‐2,10‐diene (**27**) and tenuifola‐2,11‐diene (**28**). Both the widdrane and the tenuifolane skeletons are difficult to form from natural FPP, as is evident from the low number of known natural products from these classes, but can be reached with the altered reactivity of FPP analogues with non‐natural methylation patterns. We and others have recently shown that additional methyl groups can also enzymatically be incorporated into terpenes from methylated IPP derivatives, but in these cases the additional methyl groups were not placed in positions that lead to an altered reactivity of the obtained methylated oligoprenyl diphosphate derivatives.[^40^, ^41^] Also functional groups such as the keto group in the FPP analogue **16** are tolerated by DcS, similar to recent findings for other terpene synthases,[^42^, ^43^, ^44^, ^45^] yielding products **33** and **34** with functionalised side chains that allow further chemical transformations. We have also investigated the effect of mutating aromatic amino acid residues that are, according to a homology model, located on the surface of DcS. These mutations resulted in an enzyme variant that gives significantly improved yields in preparative scale incubations, which provides another nice example for the importance of serendipity in science.

## Conflict of interest

The authors declare no conflict of interest.

## Supporting information

As a service to our authors and readers, this journal provides supporting information supplied by the authors. Such materials are peer reviewed and may be re‐organized for online delivery, but are not copy‐edited or typeset. Technical support issues arising from supporting information (other than missing files) should be addressed to the authors.

Supplementary
