# Supplementary material for: Rerouting and Improving Dauc‐8‐en‐11‐ol Synthase from Streptomyces venezuelae to a High Yielding Biocatalyst
Source: Chemistry. 2021 May 1;27(29):7923–9. doi: 10.1002/chem.202100962 (PMC8252471; doi:10.1002/chem.202100962)
Supplement: Supplementary file 1 — Supplementary [file CHEM-27-7923-s001.pdf]

Chemistry–A European Journal

Supporting Information

Rerouting and Improving Dauc-8-en-11-ol Synthase from *Streptomyces venezuelae* to a High Yielding Biocatalyst

Lukas Lauterbach[+], Anwei Hou[+], and Jeroen S. Dickschat*

Table of Contents

General experimental methods 1

GC/MS and GC/MS-QToF analyses 1

NMR spectroscopy 1

Infrared spectroscopy 1

Strains and culture conditions 1

Dauc-8-en-11-ol (8) 2

Synthetic route to 10-Me-FPP (9) 2

Protein expression and purification 10

Incubation experiments with DcS 10

Isolation of compounds 19 and 20 12

Synthetic route to 13-desmethyl-FPP (11) 35

Isolation of compound 24 42

Synthetic route to (*E*)-2-methyl-2-butenyl diphosphate (25) 50

Synthesis of trisammonium (2*E*,6*E*)-3,7,11-trimethyldodeca-2,6,11-trien-1-yl diphosphate (15) 58

Synthetic route to trisammonium (2*E*,6*E*)-3,7-dimethyl-11-oxododeca-2,6-dien-1-yl diphosphate (16) 65

Isolation of compounds 27, 28 and 29 70

Isolation of compounds 33 and 34 97

Synthetic route to trisammonium (2*E*)-3,7-dimethylocta-2,7-dien-1-yl diphosphate (36) and trisammonium (2*E*)-3-methyl-7-oxooct-2-en-1-yl diphosphate (37) 115

Synthetic route to (*S*)-6-hydroxy-6-((*S*)-4-methylcyclohex-3-en-1-yl)heptan-2-one ((6*S*,7*S*)-34) 135

Site-directed mutagenesis on dauc-8-en-11-ol synthase 146

Homologous recombination in yeast 147

Activity tests with dauc-8-en-11-ol synthase mutants 147

Determination of kinetic parameters 148

References 151

General experimental methods

All synthetic transformations were performed in oven-dried glassware under an argon atmosphere. Dried solvents used for reactions were taken from a solvent purification system (MBraun Inertgassysteme GmbH, Garching, Germany). THF was dried by distillation over Solvona (Dr. Bilger Umwelconsulting GmbH, Freigericht, Germany). Solvents used for column chromatography were distilled before use; chemical reagents were used as supplied by the vendor. Column chromatography was performed on silica gel (0.04 – 0.06 nm, Acros Organics, Geel, Belgium). Heating was performed using oil baths. Cooling was managed with mixtures of ice/water (0 °C) or liquid N_2/acetone (–78 °C) for up to 1 h, for longer times a cooling unit was used.

GC/MS and GC/MS-QToF analyses

GC/MS analyses were carried out using a 7890B GC – 5977A MD system (Agilent, Santa Clara, CA, USA). The GC was fitted with a HP5-MS UI fused silica capillary column (30 m, 0.25 mm i. d., 0.50 μm film). GC parameters were 1) inlet pressure: 77.1 kPa, He flow at 23.3 mL min^{-1}, 2) injection volume: 1 μL, 3) temperature program: 5 min at 50 °C, increasing 10 °C min^{-1} to 320 °C, 4) 60 s valve time and 5) carrier gas flow: He at 1.2 mL min^{-1}. For extracts from incubation experiments the injection volume was increased to 2 μL and the temperature ramp was decreased to 5 °C min^{-1}. MS parameters were 1) source temperature: 230 °C, 2) transfer line temperature: 250 °C, 3) quadrupole temperature: 150 °C and 4) ionization energy 70 eV. Retention indices (*I*) were calculated in comparison to a homologous series of *n*-alkanes (C_7 – C_{40}).

High resolution mass spectroscopy was conducted on a 7890B GC 7200 accurate mass Q-ToF detector system (Agilent). The GC was equipped with a HP5-MS fused silica capillary column (30 m, 0.25 mm i. d., 0.50 μm film). GC parameters were 1) injection volume: 1 μL, 2) split ratio: 5:1, 60 s valve time, 3) carrier gas flow: He at 1 ml min^{-1} and 4) temperature program: 5 min at 50 °C, increasing at 10 °C min^{-1} to 320 °C. MS parameters were 1) inlet pressure: 83.2 kPa, He flow at 24.6 ml min^{-1}, 2) transfer line temperature: 250 °C, 3) ionization energy: 70 eV.

NMR spectroscopy

NMR spectra were recorded at 298 K on a Bruker Avance I 400, Avance I 500, Avance III HD 500 or Avance III HD 700 (Bruker, Billerica, MA, USA). Spectra were referenced against residual proton signals of deuterated solvents ($CDCl_3$: δ_H = 7.26 ppm, δ_C = 77.16 ppm; C_6D_6: δ_H = 7.16 ppm, δ_H = 128.06 ppm; D_2O: δ_H = 4.79 ppm).[1] Signals are given as s = singlet, d = doublet, t = triplet, q = quartet, m = multiplet and br = broad.

Infrared spectroscopy

IR spectra were recorded using a Bruker α infrared spectrometer with a diamond ATR probehead. Band intensities are given as s (strong), m (medium), w (weak) and br (broad).

Strains and culture conditions

Escherichia coli BL21 (DE3) was grown in LB medium (10 g L^{-1} tryptone, 5 g L^{-1} yeast extract, 5 g L^{-1} NaCl, for plates 16 g L^{-1} agar-agar was added) at 37 °C. *Saccharomyces cerevisiae* FY834 was grown at 28 °C in YPAD medium (10.0 g L^{-1} yeast extract, 20.0 g L^{-1} peptone, 20.0 g L^{-1} glucose, 400 mg L^{-1} adenine sulfate) or on selection plates made from SM-URA agar (1.7 g L^{-1} yeast nitrogen base, 5.0 g L^{-1} ammonium sulfate, 20.0 g L^{-1} glucose, 770 mg L^{-1} nutritional supplement minus uracil, 20.0 g L^{-1} agar-agar). Media components were dissolved in distilled H_2O and autoclaved at 121 °C for 20 min prior to use.

Dauc-8-en-11-ol (8)

The enzyme used in this study (DcS) was initially named isodauc-8-en-11-ol synthase (IdS), and its main product isodauc-8-en-11-ol (**8**), in analogy to the known natural product isodaucene (dauca-8,11-diene, **S1**, Figure S1).[2] However, compound **8** represents the daucane skeleton (**S3**) and not the isodaucane skeleton (**S2**) and should thus correctly be named dauc-8-en-11-ol.[3] Therefore, we will use the names dauc-8-en-11-ol for **8** and dauc-8-en-11-ol synthase (DcS) for its producing enzyme.

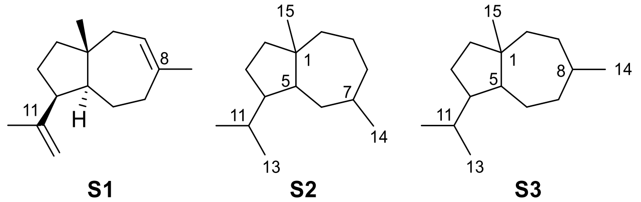

Figure S1. Structures of isodaucene (**S1**), the isodaucane (**S2**) and the daucane (**S3**) skeleton. Atom numbers represent the traditional terpene scaffold numbering.[3]

Synthetic route to 10-Me-FPP (9)

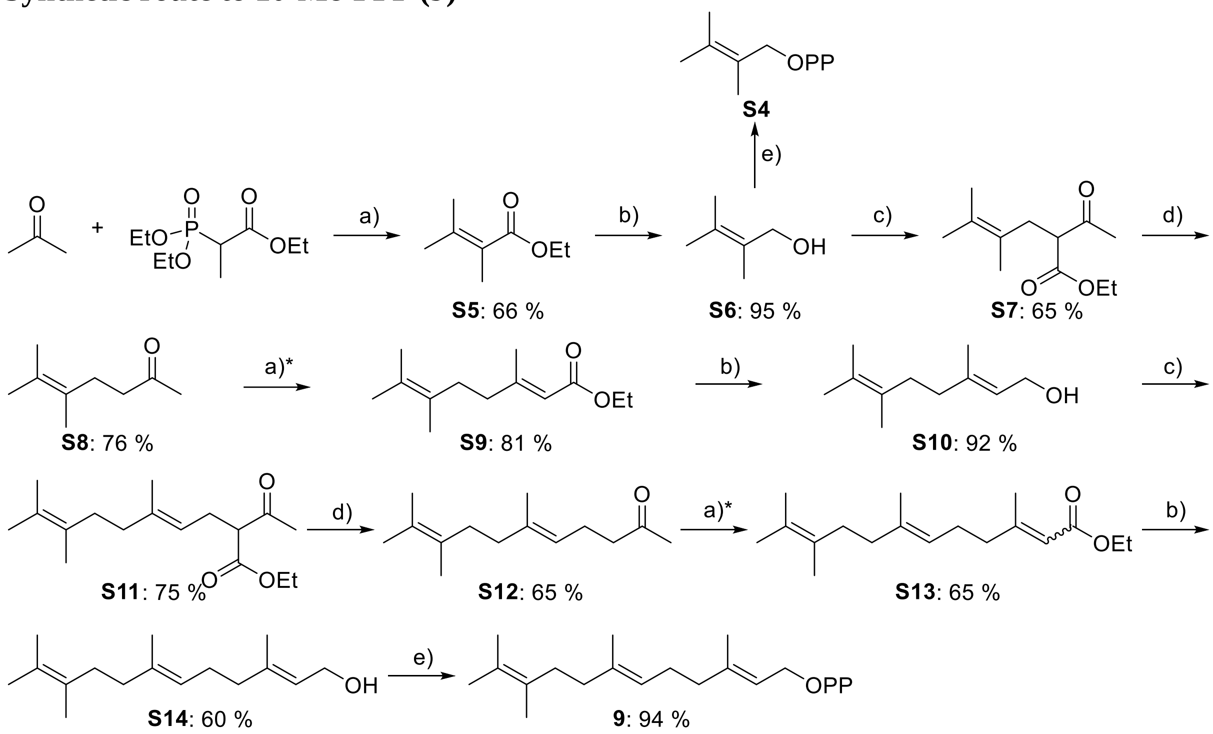

Scheme S1. Synthesis of 10-Me-FPP (**9**). Reaction conditions: a) LDA, –78 °C to room temperature, overnight; a)* LDA, triethyl phosphonoacetate, –78 °C to room temperature, overnight; b) D*i*BAl-H, Et_2O, 0 °C 1 h; c) 1. PBr_3, THF, 1.5 h, 2. K_2CO_3, ethyl acetoacetate, acetone, reflux, overnight; d) 1. KOH, EtOH, reflux, 3 h, 2. HCl; e) 1. PBr_3, THF, 1.5 h, 2. $(N\textit{n}Bu_4)_3HP_2O_7$, MeCN, overnight.

Synthesis of ethyl 2,3-dimethylbut-2-enoate (S5)

To a solution of diisopropylamine (4.63 mL, 32 mmol, 1.06 eq) in THF (200 mL, 0 °C) was added *n*BuLi (1.6 M in hexane, 20 mL, 32 mmol, 1.06 eq) and the mixture was stirred for 1 h. The mixture was cooled to –78 °C and triethyl 2-phosphonopropionate (7.86 g, 33 mmol, 1.1 eq) in THF (50 mL) was added. After 1 h acetone (1.74 g, 30 mmol, 1.0 eq) was added and the mixture was stirred overnight allowing it to warm to room temperature. Water was added to stop the reaction and the aqueous phase was extracted with ethyl acetate (3 x 200 mL). The combined organic layers were dried with $MgSO_4$, evaporated under reduced pressure and subjected to column chromatography (cyclohexane/ethyl acetate, 30:1) to yield the title

compound as a colorless liquid (3.13 g, 22 mmol, 66%). TLC (cyclohexane/ethyl acetate, 30:1): R_f = 0.42. HRMS (APCI): *m*/*z* = 143.1067 (calc. for $[C_8H_{15}O_2]^+$: 143.1067). EI-MS (70 eV): *m*/*z* (%) = 142 (68), 127 (9), 114 (10), 99 (36), 97 (100), 96 (89), 95 (8), 69 (55), 68 (21), 67 (27), 59 (13), 56 (7), 53 (8), 43 (9), 41 (41), 39 (10). GC (HP5-MS): *I* = 1000. ^{1}H-NMR ($CDCl_3$, 500 MHz): δ = 4.18 (q, $^3J_{H,H}$ = 7.2 Hz, 2H), 2.00 (br s, 3H), 1.85 (br s, 3H), 1.80 (br s, 3H), 1.30 (t, $^3J_{H,H}$ = 7.2 Hz, 3H) ppm. ^{13}C-NMR ($CDCl_3$, 125 MHz): δ = 170.0 (C_q), 142.9 (C_q), 122.8 (C_q), 60.2 (CH_2), 23.0 (CH_3), 22.5 (CH_3), 15.8 (CH_3), 14.5 (CH_3) ppm.

General procedure for synthesis of alcohols

To a cooled (0 °C) solution of ester (1.0 eq) in diethyl ether (10 mL mmol^{-1}) was added D*i*BAl-H (1 M in hexane, 2.2 eq) and the mixture was stirred for 1 h at room temperature. The mixture was cooled to 0 C again and a saturated solution of Na-K-tartrate was added. The resulting slurry was stirred for 2 h to dissolve the precipitate and the aqueous phase was extracted with diethyl ether three times. The organic layers were dried with $MgSO_4$, concentrated under reduced pressure and purified by column chromatography (pentane/diethyl ether, 10:1) to yield the desired alcohols.

2,3-Dimethylbut-2-en-1-ol (S6): Yield: 2.37 g, 20.8 mmol, 95%. TLC: (pentane/diethyl ether, 5:1): R_f = 0.38. HRMS (APCI): *m*/*z* = 101.0962 (calc. for $[C_6H_{13}O]^+$: 101.0961). EI-MS (70 eV): *m*/*z* (%) = 100 (46), 85 (100), 82 (26), 67 (43), 65 (9), 57 (12), 55 (12), 53 (8), 43 (22), 41 (36), 39 (14). GC (HP5-MS): *I* = 838. ^{1}H-NMR ($CDCl_3$, 400 MHz, 298 K): δ = 4.12 (s, 2H), 1.76 – 1.72 (m, 6H), 1.68 (s, 1H) ppm. ^{13}C-NMR ($CDCl_3$, 100 MHz): δ = 129.4 (C_q), 127.5 (C_q), 64.1 (CH_2), 21.0 (CH_3), 20.1 (CH_3), 16.7 (CH_3) ppm. IR (diamond ATR): $\tilde{\nu}$ = 3356 (br w), 2960 (m), 2925 (m), 2858 (w), 1665 (w), 1457 (w), 1378 (w), 1261 (s), 1091 (s), 1023 (s), 866 (w), 800 (s), 695 (w) cm^{-1}.

(*E*)-3,6,7-Trimethylocta-2,6-dien-1-ol (S10): Yield: 2.01 g, 12.0 mmol, 92%. TLC (pentane/diethyl ether, 7:1): R_f = 0.40. HRMS (APCI): *m*/*z* = 169.1587 (calc. for $[C_{11}H_{21}O]^+$: 169.1587). EI-MS (70 eV): *m*/*z* (%) = 168 (3), 150 (7) 137 (34), 135 (13), 121 (9), 110 (15), 107 (22), 84 (32), 83 (100), 82 (20), 67 (18), 55 (87), 41 (22). GC (HP5-MS): *I* = 1354. ^{1}H-NMR ($CDCl_3$, 500 MHz): δ = 5.41 (tq, $^3J_{H,H}$ = 7.2 Hz, $^4J_{H,H}$ = 1.2 Hz, 1H), 4.15 (d, $^3J_{H,H}$ = 7.2 Hz, 2H), 2.13 (m, 2H), 2.03 (m, 2H), 1.70 (s, 3H), 1.64 (br s, 9H) ppm. ^{13}C-NMR ($CDCl_3$, 125 MHz): δ = 140.4 (C_q), 127.4 (C_q), 124.5 (C_q), 123.2 (CH), 59.6 (CH_2), 38.2 (CH_2), 33.4 (CH_2), 20.7 (CH_3), 20.2 (CH_3), 18.5 (CH_3), 16.5 (CH_3) ppm. IR (diamond ATR): $\tilde{\nu}$ = 3385 (br w), 3978 (m), 2924 (m), 2867 (m), 1667 (m), 1448 (m), 1376 (s), 1185 (m), 1116 (s), 1003 (s), 899 (w), 850 (m), 807 (w), 662 (w), 537 (m), 494 (m) cm^{-1}.

(2*E*,6*E*)-3,7,10,11-Tetramethyldodeca-2,6,10-trien-1-ol (S14): Yield: 0.54 g, 2.26 mmol, 60%. TLC (cyclohexane/ethyl acetate, 9:1): R_f = 0.30. HRMS (APCI): *m*/*z* = 235.2055 (calc. for $[C_{16}H_{28}O\ \text{-H}]^+$: 235.2056). EI-MS (70 eV): *m*/*z* (%) = 236 (1), 218 (2), 175 (3), 150 (3), 141 (15), 137 (6), 135 (6), 121 (11), 107 (11), 95 (9), 93 (12), 83 (100), 67 (7), 55 (25), 41 (9). GC (HP5-MS): *I* = 1836. ^{1}H-NMR ($CDCl_3$, 500 MHz): δ = 5.45 (tq, $^3J_{H,H}$ = 7.0 Hz, $^4J_{H,H}$ = 1.3 Hz, 1H), 5.13 (tq, $^3J_{H,H}$ = 6.7 Hz, $^4J_{H,H}$ = 1.2 Hz, 1H), 4.18 (d, $^3J_{H,H}$ = 7.2 Hz, 2H), 2.17 – 2.09 (m, 4H), 2.09 – 2.05 (m, 2H), 2.03 – 1.98 (m, 2H) ppm. ^{13}C-NMR ($CDCl_3$, 125 MHz): δ = 140.0 (C_q), 136.0 (C_q), 127.8 (C_q), 124.1 (C_q), 123.7 (CH), 123.5 (CH), 59.6 (CH_2), 39.7 (CH_2), 38.4 (CH_2), 33.8 (CH_2), 26.5 (CH_2), 20.7 (CH_3), 20.2 (CH_3), 18.6 (CH_3), 16.4 (CH_3), 16.3 (CH_3) ppm. IR (diamond ATR): $\tilde{\nu}$ = 3437 (br w), 2975 (m), 2935 (m), 2865 (m), 1717 (w), 1449 (w), 1377 (s), 1215 (w), 1113 (s), 957 (m), 843 (w), 797 (w), 443 (m) cm^{-1}.

General procedure for the synthesis of allyl bromides

To a cooled (0 °C) solution of alcohol (1.0 eq) in THF (5 mL mmol^{-1}) PBr_3 (0.4 eq) was added carefully. The mixture was stirred for 30 min at 0 °C and 1 h at room temperature before transferring it directly to an ice/water mixture. The aqueous layer was extracted with hexane twice and the organic layers were dried with $MgSO_4$, concentrated under reduced pressure and the bromides were directly used for subsequent reactions without purification.

General procedure for the synthesis of β-keto esters

The crude bromide was diluted with acetone (3 mL mmol^{-1}), potassium carbonate (1.5 eq) and ethyl acetoacetate (3.0 eq) were added and the mixture was refluxed overnight. The suspension was filtered and the filtrate was evaporated to dryness under reduced pressure. The residue was subjected to column chromatography (cyclohexane/ethyl acetate, 7:1) to yield the products as colorless oils.

Ethyl 2-acetyl-4,5-dimethylhex-4-enoate (S7): Yield: 2.91 g, 13.7 mmol, 65%. TLC (cyclohexane/ethyl acetate, 7:1): R_f = 0.40. HRMS (APCI): *m/z* = 213.1486 (calc. for $[C_{12}H_{21}O_3]^+$: 213.1485). EI-MS (70 eV): *m/z* (%) = 194 (69), 167 (12), 151 (8), 139 (13) 130 (19), 123 (100), 121 (89), 107 (11), 102 (11), 97 (14) 95 (36), 83 (28), 82 (34), 67 (23), 55 (19), 43 (25) 41 (9). GC (HP5-MS): *I* = 1407. ^{1}H-NMR ($CDCl_3$, 500 MHz): δ = 4.16 (qq, $^3J_{H,H}$ = 10.8, 7.2 Hz, 2H), 3.57 (dd, $^3J_{H,H}$ = 8.1, 7.3 Hz, 1H), 2.67 (dd, $^2J_{H,H}$ = 14.0 Hz, $^3J_{H,H}$ = 8.2 Hz, 1H), 2.57 (dd, $^2J_{H,H}$ = 14.0 Hz, $^3J_{H,H}$ = 7.1 Hz, 1H), 2.21 (s, 3H), 1.66 (br s, 3H), 1.62 (br s, 6H), 1.25 (t, $^3J_{H,H}$ = 7.2 Hz, 3H) ppm. ^{13}C-NMR ($CDCl_3$, 125 MHz): δ = 203.5 (C_q), 170.1 (C_q), 127.9 (C_q), 123.4 (C_q), 61.4 (CH_2), 58.5 (CH), 33.2 (CH_2), 20.9 (CH_3), 20.6 (CH_3), 18.1 (CH_3), 14.2 (CH_3) ppm. IR (diamond ATR): $\tilde{\nu}$ = 2984 (w), 2920 (w), 2865 (w), 1737 (s), 1715 (s), 1640 (w), 1447 (w), 1361 (m), 1329 (w), 1270 (m), 1223 (m), 1182 (m), 1150 (m), 1058 (w), 1023 (w), 856 (w) cm^{-1}.

Ethyl (*E*)-2-acetyl-5,8,9-trimethyldeca-4,8-dienoate (S11): Yield: 2.51 g, 8.97 mmol, 75%. TLC (cyclohexane/ethyl acetate, 10:1): R_f = 0.40. HRMS (APCI): *m/z* = 281.2110 (calc. for $[C_{17}H_{29}O_3]^+$: 281.2111). EI-MS (70 eV): *m/z* (%) = 280 (12), 262 (15), 237 (20), 197 (29), 189 (15), 155 (40), 151 (17), 150 (17), 143 (17), 135 (12), 130 (18), 123 (71), 121 (10), 109 (46), 107 (42), 83 (100), 81 (18), 55 (35), 43 (28). GC (HP5-MS): *I* = 1887. ^{1}H-NMR ($CDCl_3$, 500 MHz) δ = 5.03 (tq, $^3J_{H,H}$ = 7.2 Hz, $^4J_{H,H}$ = 1.2 Hz, 1H), 4.18 (q, $^3J_{H,H}$ = 7.1 Hz, 2H), 3.42 (t, $^3J_{H,H}$ = 7.7 Hz, 1H), 2.54 (ddd, $^3J_{H,H}$ = 7.6, 7.1 Hz, $^4J_{H,H}$ = 3.0 Hz, 2H), 2.22 (s, 3H), 2.09 – 2.04 (m, 2H), 2.00 – 1.95 (m, 2H), 1.64 (s, 3H), 1.62 (s, 6H), 1.61 (s, 3H) ppm. ^{13}C-NMR ($CDCl_3$, 125 MHz): δ = 203.3 (C_q), 169.8 (C_q), 139.0 (C_q), 127.5 (C_q), 124.3 (C_q), 119.6 (CH), 61.4 (CH_2), 60.0 (CH), 38.4 (CH_2), 33.6 (CH_2), 29.2 (CH_2), 27.1 (CH_2), 20.7 (CH_3), 20.2 (CH_3), 18.5 (CH_3), 16.3 (CH_3), 14.3 (CH_3) ppm. IR (diamond ATR): $\tilde{\nu}$ = 2681 (m), 2928 (m), 2867 (w), 1717 (s), 1643 (w), 1446 (m), 1368 (m), 1238 (s), 1195 (s), 1150 (s), 1098 (m), 1028 (m), 957 (w), 898 (w), 856 (w), 665 (w), 540 (w) cm^{-1}.

General procedure for the synthesis of methyl ketones

A solution of β-keto esters (1.0 eq) in ethanol (2.5 mL mmol^{-1}) was mixed with an aqueous potassium hydroxide solution (3M, 2.0 eq) and heated to reflux for 3.5 h. After cooling to room temperature 2 M hydrochloric acid was added and the mixture was diluted with ethyl acetate. The phases were separated and the aqueous phase was extracted with ethyl acetate twice more. The combined organic layers were dried with $MgSO_4$, concentrated under reduced pressure and subjected to column chromatography (cyclohexane/ethyl acetate, 10:1) to give the pure methyl ketones as colorless oils.

5,6-Dimethylhept-5-en-2-one (S8): 1.46 g, 10.4 mmol, 76%. TLC (cyclohexane/ethyl acetate, 10:1): R_f = 0.35. HRMS (APCI): *m/z* = 141.1274 (calc. for $[C_9H_{17}O_1]^+$: 141.1274).

EI-MS (70 eV): m/z (%) = 140 (17), 125 (21), 122 (100), 107 (62), 97 (27), 83 (45), 82 (44), 69 (14) 67 (54), 58 (10), 55 (77), 43 (48), 41 (29). GC (HP5-MS): I = 1085. ^{1}H-NMR ($CDCl_3$, 400 MHz): 2.46 (t, $^{3}J_{H,H}$ = 7.9 Hz, 2H), 2.28 (t, $^{3}J_{H,H}$ = 7.9 Hz, 2H), 2.14 (s, 3H), 1.64 (br s, 3H), 1.63 (br s, 3H), 1.61 (br s, 3H) ppm. ^{13}C-NMR ($CDCl_3$, 100 MHz): δ = 200.4 (C_q), 126.1 (C_q), 125.4 (C_q), 42.5 (CH_2), 30.0 (CH_3), 28.9 (CH_2), 20.7 (CH_3), 20.2 (CH_3), 18.2 (CH_3) ppm. IR (diamond ATR): $\tilde{\nu}$ = 2967 (s), 2932 (s), 2880 (m), 1714 (m), 1460 (m), 1373 (s), 1313 (w), 1258 (m), 1159 (m), 1092 (s), 1026 (s), 959 (m), 890 (w), 802 (m), 671 (w), 470 (w) cm^{-1}.

(*E*)-6,9,10-Trimethylundeca-5,9-dien-2-one (S12): Yield: 1.18 g, 5.8 mmol, 65%. TLC (cyclohexane/ethyl acetate, 12:1): R_f = 0.37. HRMS (APCI): m/z = 209.1897 (calc. for $[C_{14}H_{25}O]^+$: 209.1900). EI-MS (70 eV): m/z (%) = 208 (11), 190 (6), 175 (4), 165 (15), 150 (20), 137 (7), 125 (28), 123 (18), 108 (10), 107 (33), 93 (6), 83 (100), 82 (8), 67 (10), 55 (44), 43 (50), 41 (11). GC (HP5-MS): I = 1558. ^{1}H-NMR ($CDCl_3$, 500 MHz): δ = 5.07 (tq, $^{3}J_{H,H}$ = 7.2 Hz, $^{4}J_{H,H}$ = 1.2 Hz, 1H), 2.45 (dd, $^{3}J_{H,H}$ = 7.5, 7.3 Hz, 2H), 2.25 (ddd, $^{3}J_{H,H}$ = 7.5, 7.2, 7.2 Hz, 2H), 2.13 (s, 3H), 2.08 (dd, $^{3}J_{H,H}$ = 9.1, 6.8 Hz, 2H), 1.97 (dd, $^{3}J_{H,H}$ = 9.0, 7.1 Hz, 2H), 1.63 (br s, 12H) ppm. ^{13}C-NMR ($CDCl_3$, 125 MHz): δ = 209.0 (C_q), 137.0 (C_q), 127.6 (C_q), 124.2 (C_q), 122.4 (CH), 43.9 (CH_2), 38.3 (CH_2), 33.6 (CH_2), 30.1 (CH_3), 22.6 (CH_2), 20.7 (CH_3), 20.2 (CH_3), 18.5 (CH_2), 16.2 (CH_2) ppm. IR (diamond ATR): $\tilde{\nu}$ =2974 (w), 2920 (m), 2864 (w), 1713 (s), 1443 (m), 1364 (s), 1231 (w), 1157 (s), 1121 (m), 1049 (w), 1021 (w), 954 (w), 900 (w), 858 (w), 731 (w), 577 (m), 537 (m) cm^{-1}.

General procedure for HWE reactions

To a solution of diisopropylamine (1.06 eq) in THF (8 mL $mmol^{-1}$, 0 °C) was added *n*BuLi (1.6 M in hexane, 1.06 eq) and the mixture was stirred for 1 h. The mixture was cooled to –78 °C and triethyl phosphonoacetate (1.1 eq) in THF (1.5 mL $mmol^{-1}$) was added. After 1 h a solution of the methyl ketone (1.0 eq) in THF (0.5 mL $mmol^{-1}$) was added and the mixture was stirred overnight allowing it to warm to room temperature. Water was added to stop the reaction and the aqueous phase was extracted with ethyl acetate three times. The combined organic layers were dried with $MgSO_4$, evaporated under reduced pressure and subjected to column chromatography (cyclohexane/ethyl acetate, 45:1) to yield the desired esters as colorless oils.

Ethyl (*E*)-3,6,7-trimethylocta-2,6-dienoate (S9): Yield: 2.73 g, 13.0 mmol, 81%. TLC (cyclohexane/ethyl acetate, 10:1): R_f = 0.40. HRMS (APCI): m/z = 211.1690 (calc. for $[C_{13}H_{23}O_2]^+$: 211.1693). EI-MS (70 eV): m/z (%) = 210 (6), 165 (12), 137 (15), 128 (50), 122 (20), 100 (19), 83 (100), 55 (36), 41 (9). GC (HP5-MS): I = 1492 ^{1}H-NMR ($CDCl_3$, 500 MHz): δ = 5.68 (q, $^{4}J_{H,H}$ = 1.2 Hz, 1H)), 4.17 (q, $^{3}J_{H,H}$ = 7.1 Hz, 2H), 2.20 (d, $^{4}J_{H,H}$ = 1.3 Hz, 3H), 2.20 – 2.17 (m, 4H), 1.66 (s, 9H), 1.30 (t, $^{3}J_{H,H}$ = 7.1 Hz, 3H) ppm. ^{13}C-NMR ($CDCl_3$, 125 MHz): δ = 167.1 (C_q), 160.4 (C_q), 126.6 (C_q), 125.2 (C_q), 115.6 (CH), 59.6 (CH_2), 39.7 (CH_2), 33.1 (CH_2), 20.7 (CH_3), 20.2 (CH_3), 19.1 (CH_3), 18.5 (CH_3), 14.5 (CH_3) ppm. IR (diamond ATR): $\tilde{\nu}$ = 2982 (w), 2923 (w), 2866 (w), 1713 (s), 1647 (m), 1447 (w), 1376 (m), 1273 (w), 1218 (s), 1144 (s), 1045 (m), 861 (m), 733 (m), 553 (w), 483 (w) cm^{-1}.

Ethyl (6*E*)-3,7,10,11-tetramethyldodeca-2,6,10-trienoate (S13): Yield: 1.05, 3.77 mmol, 65%, *E*/*Z* = 3:1. TLC (cyclohexane/ethyl acetate, 10:1): R_f = 0.45. HRMS (APCI): m/z = 279.2321 (calc. for $[C_{18}H_{31}O_2]^+$: 279.2319). EI-MS (70 eV, *E* isomer): m/z (%) = 278 (6), 233 (11), 207 (11), 153 (9), 151 (13), 128 (73), 123 (23), 121 (18), 107 (10), 100 (10), 95 (9), 83 (100), 82 (12), 67 (6), 55 (28), 41 (7). GC (HP5-MS, *E* isomer): I = 1959. ^{1}H-NMR ($CDCl_3$, 500 MHz): δ = 5.66 (q, $^{4}J_{H,H}$ = 1.0 Hz, 1H, *E*), 5.65 (q, $^{4}J_{H,H}$ = 1.0 Hz, 1H, *Z*), 5.17 (tq, $^{3}J_{H,H}$ =7.2 Hz, $^{4}J_{H,H}$ = 1.1 Hz, 1H, *Z*), 5.11 – 5.07 (m, 1H, *E*), 4.15 (q, $^{3}J_{H,H}$ = 7.1 Hz, 2H, *E*), 4.14

(q, $^3J_{H,H}$ = 7.2 Hz, 2H, *Z*), 2.64 (t, $^3J_{H,H}$ = 7.8 Hz, 2H, *Z*), 2.19 – 2.14 (m, 3x 2H, 2x *E*, 1x *Z*), 2.16 (d, $^4J_{H,H}$ = 1.2 Hz, 3H, *E*), 2.11 – 2.06 (m, 2x 2H 1x *E*, 1x *Z*), .2.01 – 1.96 (m, 2x 2H, 1x *E*, 1x *Z*), 1.89 (d, $^4J_{H,H}$ = 1.3 Hz, 3H, *Z*), 1.64 – 1.61 (m, 8x 3H, 4x *E*, 4x *Z*), 1.27 (t, $^3J_{H;H}$ = 7.1 Hz, 3H, *E*), 1.26 (t, $^3J_{H,H}$ = 7.2 Hz, 3H, *Z*) ppm. ^{13}C-NMR ($CDCl_3$, 125 MHz): δ = 167.0 (C_q, *E*), 166.5 (C_q, *Z*), 160.3 (C_q, *Z*), 159.9 (C_q, *E*), 136.7 (C_q, *E*), 136.3 (C_q, *Z*), 127.8 (C_q, *Z*), 127.7 (C_q, *E*), 124.0 (C_q, *E*), 124.1 (C_q, *Z*), 123.5 (CH, *Z*), 122.8 (CH, *E*), 116.4 (CH, *Z*), 115.8 (CH, *E*), 59.59 (CH_2, *E*), 59.56 (CH_2, *Z*), 41.1 (CH_2, *E*), 38.4 (CH_2, *Z*), 38.3 (CH_2, *E*), 33.71 (CH_2, *Z*), 33.68 (CH_2, *E*), 33.6 (CH_2, *Z*), 26.9 (CH_2, *Z*), 26.2 (CH_2, *E*), 25.5 (CH_3, *Z*), 20.7 (CH_3, *E*), 20.18 (CH_3, *E*), 20.17 (CH_3, *Z*), 19.0 (2x CH_3, 1x *E*, 1x *Z*), 18.6 (CH_3, *Z*), 18.5 (CH_3, *E*), 16.3 (CH_3, *E*), 16.2 (CH_3, *Z*), 14.5 (2x CH_3, 1x *E*, 1x *Z*) ppm. IR (diamond ATR): $\tilde{\nu}$ = 2976 (w), 2934 (w), 2865 (w), 1714 (s), 1647 (w), 1447 (m), 1377 (m), 1271 (w), 1219 (s), 1146 (s), 1115 (s), 1038 (m), 854 (m), 733 (w), 667 (w), 567 (w), 527 (w), 494 (w) cm^{-1}.

General procedure for synthesis of diphosphates

To a solution of tris(tetra-*n*-butylammonium) hydrogen diphosphate (1.4 eq) in acetonitrile (7.5 mL $mmol^{-1}$) a solution of crude allyl bromide (1.0eq) was added and the mixture was stirred at room temperature overnight. Acetonitrile was removed at the rotary evaporator and the resulting residue was redissolved in aqueous NH_4HCO_3 solution (0.25 M) and loaded onto a DOWEX 50WX8 ion-exchange column (NH_4^+ form, pH ~ 7.0). The column was flushed slowly with 1.5 CV of NH_4HCO_3 solution (25 mM, 5% *i*PrOH) and the eluate was lyophilized to yield the diphosphates. The salt was purified by redissolving in NH_4HCO_3 solution (50 mM) followed by precipitation of inorganic diphosphates by addition of *iso*-propanol/acetonitrile (1:1). The salts were removed by centrifugation (14.000 x g, 10 min) and the supernatant was transferred to a flask. The procedure was repeated with the salts twice and the combined supernatant fractions were lyophilized again giving the pure diphosphates as off-white hygroscopic powder.

2,3,3-Trimethylallyl pyrophosphate (S4): Yield: 0.57 g, 1.83 mmol, 91%. ^{1}H-NMR (D_2O, 500 MHz): δ = 4.45 (d, $^3J_{H,P}$ = 4.9 Hz, 2H), 1.76 (s, 3H), 1.73 (s, 3H), 1.70 (s, 3H) ppm. ^{13}C-NMR (D_2O, 125 MHz): δ = 132.5 (C_q), 124.3 (d, $^3J_{C,P}$ = 8.6 Hz, C_q), 66.8 (d, $^2J_{C,P}$ = 5.4 Hz, CH_2), 20.2 (CH_3), 19.5 (CH_3), 16.0 (CH_3) ppm. ^{31}P-NMR (D_2O, 202 MHz), δ = –6.5 (d, $^2J_{P,P}$ = 22.5 Hz), –10.3 (d, $^2J_{P,P}$ = 21.7 Hz) ppm. IR (diamond ATR): $\tilde{\nu}$ = 2958 (m), 2858 (m), 2790 (m), 1914 (w), 1712 (w), 1686 (w), 1432 (m), 1400 (m), 1272 (w), 1201 (m), 1078 (s), 1026 (s), 884 (s), 824 (m), 712 (m), 522 (s) cm^{-1}.

10-Methylfarnesyl pyrophosphate (9): Yield: 0.95 g, 2.11 mmol, 94%. ^{1}H-NMR (D_2O, 500 MHz): δ = 5.38 (t, $^3J_{H,H}$ = 6.6 Hz, 1 H), 5.06 (t, $^3J_{H,H}$ = 6.1 Hz, 1H), 4.39 (dd, $^3J_{H,H}$ = 6.1 Hz, $^3J_{H,P}$ = 5.6 Hz, 2H), 2.05 – 1.93 (m, 6H), 1.92 – 1.86 (m, 2H), 1.64 (s, 3H), 1.54 (br s, 12H) ppm. ^{13}C-NMR (D_2O, 125 MHz): δ = 142.2 (C_q), 135.6 (C_q), 127.5 (C_q), 123.8 (CH), 123.5 (C_q), 119.6 (d, $^3J_{C,P}$ = 8.6 Hz, CH), 62.5 (d, $^2J_{C,P}$ = 5.4 Hz, CH_2), 39.4 (CH_2), 38.1 (CH_2), 33.4 (CH_2), 26.4 (CH_2), 20.1 (CH_3), 19.7 (CH_3), 18.0 (CH_3), 15.9 (CH_3), 15.7 (CH_3) ppm. ^{31}P-NMR (D_2O, 202 MHz): δ = –7.7 (d, $^3J_{P,P}$ = 19.4 Hz), –10.4 (d, $^3J_{P,P}$ = 19.0 Hz) ppm. IR (diamond ATR): $\tilde{\nu}$ = 3022 (m), 2906 (m), 2848 (m), 2116 (w), 1875 (w), 1676 (w), 1435 (m), 1406 (m), 1263 (w), 1195 (m), 1162 (m), 1114 (s), 1078 (s), 1017 (s), 898 (s), 802 (m), 715 (m), 503 (s) cm^{-1}.

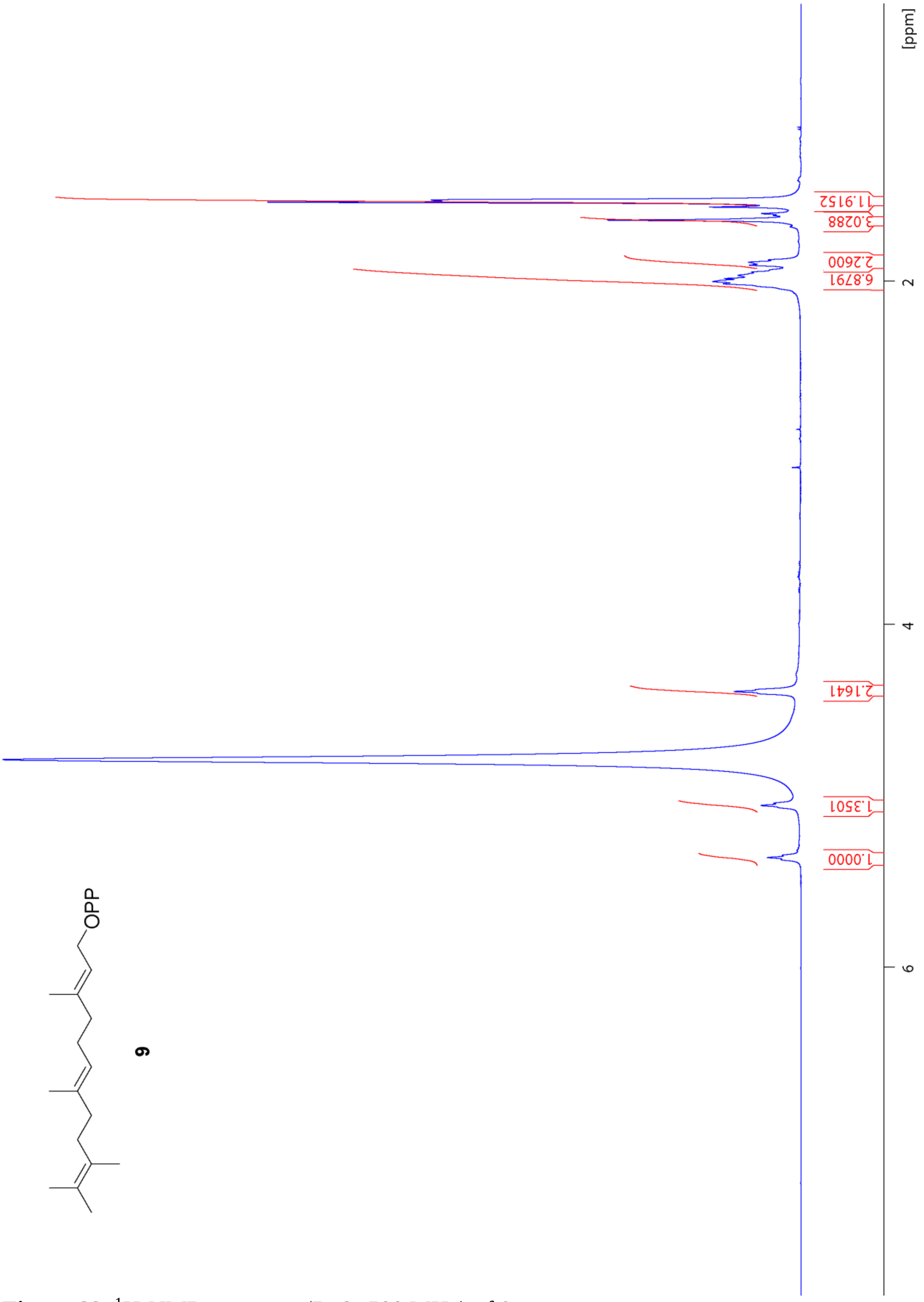

Figure S2. ^{1}H-NMR spectrum (D_2O, 500 MHz) of **9**.

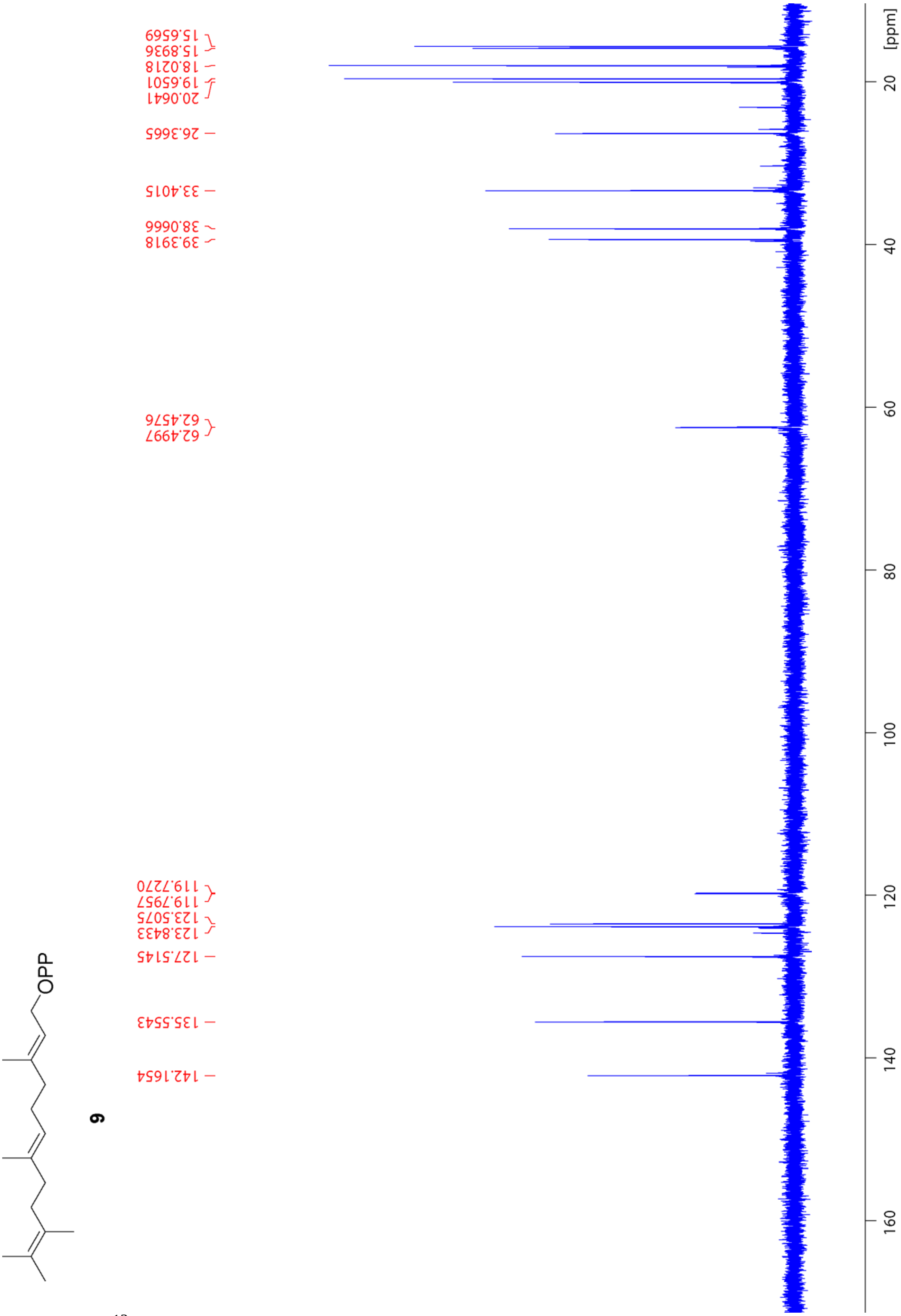

Figure S3. ^{13}C-NMR spectrum (D_2O, 125 MHz) of **9**.

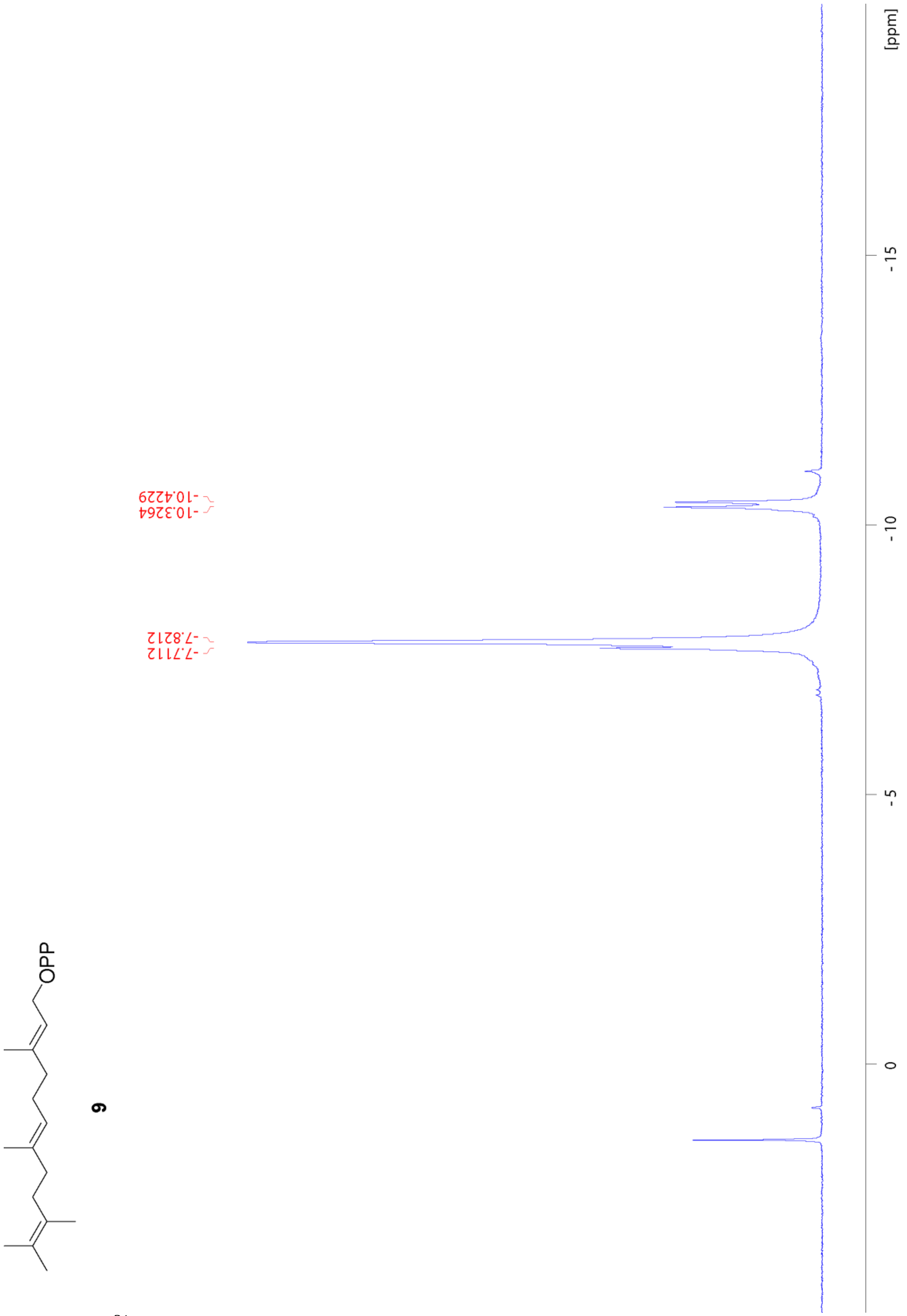

Figure S4. ^{31}P-NMR spectrum (D_2O, 202 MHz) of **9**.

Protein expression and purification

LB medium with kanamycin (50 µg mL^{-1}) was inoculated with a glycerol stock of *E. coli* BL21 containing a pET28c derived expression plasmid for DcS[4] and was grown with shaking overnight. The preculture was used to inoculate a larger amount of LB medium (1 ml L^{-1}) containing kanamycin. The culture was grown until an OD_{600} between 0.4 and 0.6 was reached and was cooled to 18 °C. After 30 min IPTG (final conc. 400 µM) was added to induce expression and the culture was shaken overnight. The cells were harvested by centrifugation (3.600 x g, 45 min, 4 °C) and the cell pellet was resuspended in binding buffer (20 mM Na_2HPO_4, 0.5 M NaCl, 20 mM imidazole, 1 mM $MgCl_2$, pH 7.4, 10 mL L^{-1} culture). The cells were lysed by sonication on ice (6 x 1 min, 1 min pause between pulses) and the resulting slurry was centrifuged (14.100 x g, 2 x 7 min, 4 °C) to remove the cell debris. The supernatant was filtered through a syringe filter (regenerated cellulose, 0.45 µm) and loaded to a Ni-NTA column (Protino Ni-NTA, Macherey-Nagel, Düren, Germany) equilibrated with binding buffer. The column was washed with binding buffer three times (10 mL L^{-1} culture) and the desired protein was eluted with elution buffer (20 mM Na_2HPO_4, 0.5 M NaCl, 500 mM imidazole, 1 mM $MgCl_2$, pH 7.4, 10 mL L^{-1} culture).

Incubation experiments with DcS

In small scale incubation experiments DcS (final concentration 75 µM) was mixed with substrate (1 mg mL^{-1}) dissolved in NH_4HCO_3 solution (25 mM, 0.3 mL mg^{-1}) and the reaction mixtures were diluted in a 1:1 ratio with incubation buffer (50 mM TRIS, 10 mM $MgCl_2$, 20% glycerol, pH 8.2) and incubated at 28 °C for 3 h. Extracts were directly subjected to NMR or GC-MS. For experiments with shorter substrates FPPS[5] (final concentration 1.5 µM) and IPP (0.5 mg mL^{-1}) were added. Large scale incubations for the preparative isolation of enzyme products were performed with enzyme concentrations similar to that for the small scale (see below) and with a substrate concentration of 0.5 mg mL^{-1}. Reaction mixtures were diluted with incubation buffer in a 1:1 ratio and were incubated at 28 °C overnight.

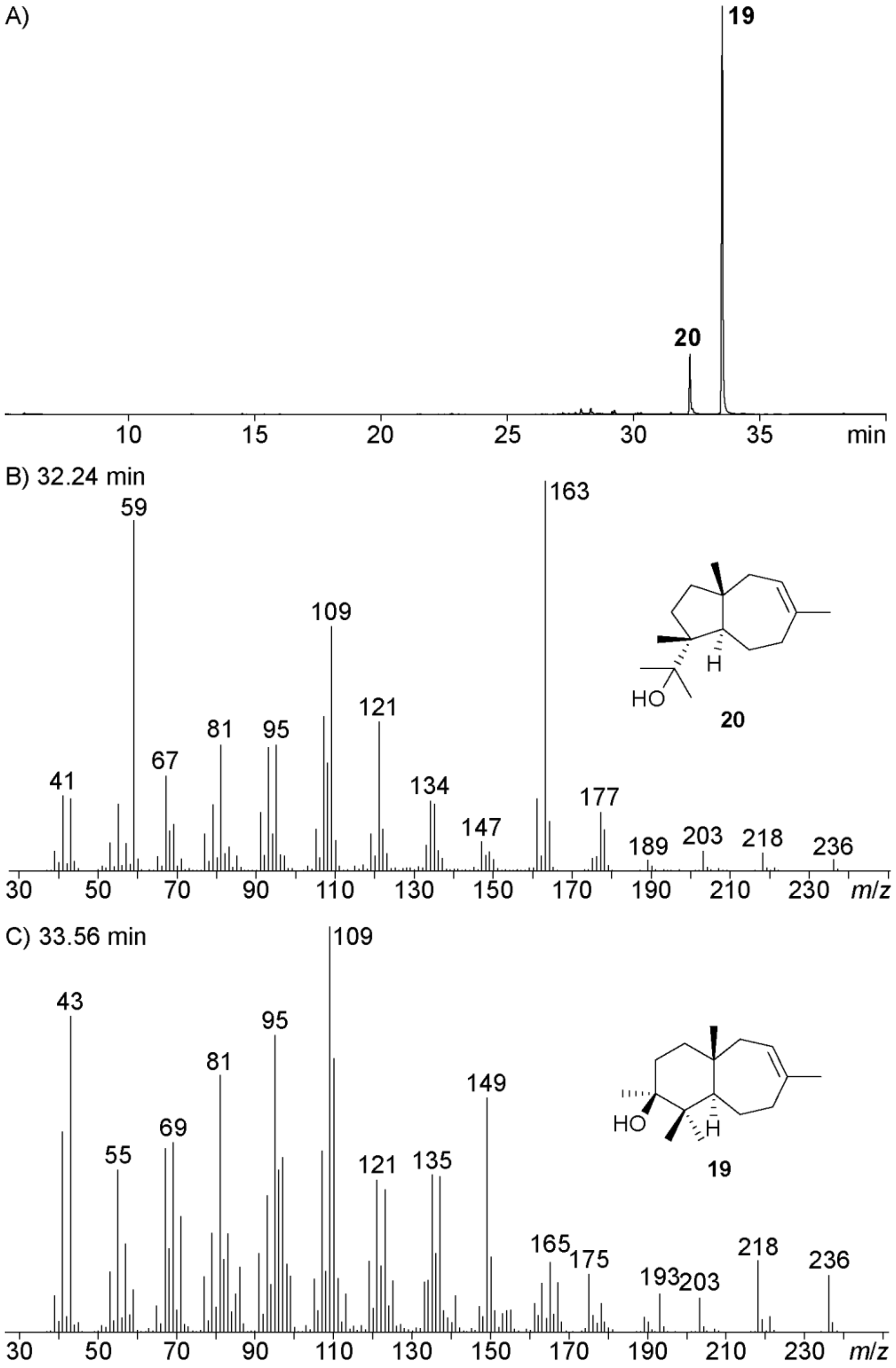

Figure S5. Incubation of DcS with 10-Me-FPP (**9**). A) Total ion chromatogram of the crude mixture. EI-MS spectra of compounds B) **20** and C) **19**.

Isolation of compounds 19 and 20

A solution of recombinant DcS (20 mL, concentration: 149 μmol L^{-1}) obtained from 8 L of *E. coli* culture was incubated with 10-Me-FPP (**9**, 80 mg, 0.18 mmol) as described above. The reaction mixture was extracted with *n*-hexane three times and the organic layers were dried with $MgSO_4$, concentrated under reduced pressure and subjected to column chromatography (pentane/diethyl ether, 5:1) to yield compounds **19** (6.2 mg, 0.03 mmol, 15%) and **20** (1.6 mg, 0.01 mmol, 4%) as colorless oils.

3-Methylwiddr-8-en-3-ol, (2*S*,4a*R*,9a*R*)-1,1,2,4a,7-pentamethyl-2,3,4,4a,5,8,9,9a-octahydro-1H-benzo[7]annulen-2-ol (19): TLC (pentane/diethyl ether, 5:1): R_f = 0.35. Optical rotation: $[\alpha]_D^{20}$ = +194.0 (*c* 0.1, C_6D_6). GC (HP5-MS): *I* = 1768. HRMS (QToF): *m*/*z* = 236.2131 (calc. for $[C_{16}H_{28}O]^+$ 236.2135). MS (EI, 70 eV) *m*/*z* (%) = 236 (13), 218 (16), 203 (7), 193 (8), 175 (14), 167 (12), 165 (17), 163 (12), 150 (18), 137 (38), 136 (19), 135 (36), 134 (11), 133 (11), 125 (13), 123 (35), 122 (16), 121 (34), 119 (15), 110 (70), 109 (199), 107 (43), 97 (45), 96 (42), 95 (73), 93 (31), 83 (24), 81 (64), 79 (23), 71 (30), 69 (47), 67 (45), 57 (22), 55 (40), 43 (79), 41 (48) (Figure S5C). IR (diamond ATR): $\tilde{\nu}$ = 3419 (w), 2965 (s), 2918 (s), 2857 (s), 1672 (w), 1457 (m), 1380 (w), 1260 (m), 1107, (m), 1048 (w), 794 (s) cm^{-1}. NMR data are given in Table S1 and Figures S7 – S13.

4-*epi*-4-Methyldauc-8-en-11-ol 2-((1*R*,3a*R*,8a*R*)-1,3a,6-trimethyl-1,2,3,3a,4,7,8,8a-octahydroazulen-1-yl)propan-2-ol (20): TLC (pentane/diethyl ether, 5:1): R_f = 0.31. Optical rotation $[\alpha]_D^{20}$ = +4.8 (*c* 0.13, C_6D_6). GC (HP5-MS): *I* = 1710. HRMS (QToF): *m*/*z* = 218.2027 (calc. for $[C_{16}H_{28}O - H_2O]^+$ 218.2029). MS (EI, 70 eV): *m*/*z* (%) = 236 (3), 218 (4), 203 (4), 177 (15), 163 (100), 161 (18), 147 (6), 135 (15), 134 (17), 122 (19), 121 (37), 109 (62), 108 (26), 107 (37), 95 (30), 93 (28), 91 (13), 81 (30), 79 (15), 69 (11), 67 (23), 59 (87), 55 (15), 43 (15), 41 (17) (Figure S5B). IR (diamond ATR): $\tilde{\nu}$ = 3351 (w), 2960 (m), 2924 (s), 2855 (m), 1663 (w), 1458 (w), 1376 (w), 1260 (m), 1091 (s), 1018 (s), 799 (s) cm^{-1}. NMR data are given in Table S2 and Figures S14 – S20.

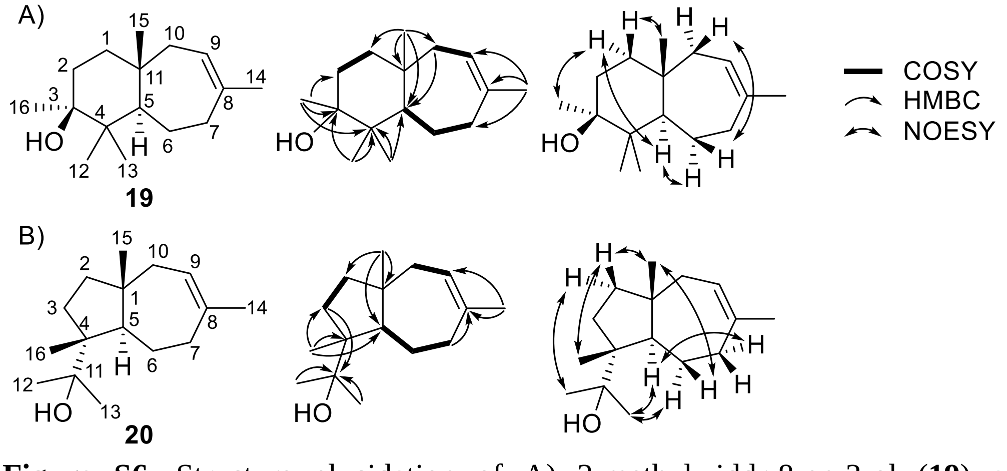

Figure S6. Structure elucidation of A) 3-methylwiddr-8-en-3-ol (**19**) and B) 4-*epi*-4-methyldauc-8-en-11-ol (**20**). Bold lines indicate $^1H,^1H$-COSY correlations, single headed arrows HMBC correlations and double headed arrows NOESY correlation.

Table S1. NMR data of **19** recorded in C_6D_6.

C[a]		^{1}H[b]	^{13}C[b]
1	CH_2	1.27 (ddd, $^{2}J_{H,H}$ = 14.0, $^{3}J_{H,H}$ = 13.4, 3.8, 1H)	40.1
		1.14 (d, $^{3}J_{H,H}$ = 2.7, 1H)	
2	CH_2	1.78 (ddd, $^{2}J_{H,H}$ = 13.8, $^{3}J_{H,H}$ = 13.2, 3.6, 1H)	35.1
		1.20 (ddd, $^{2}J_{H,H}$ = 13.2, $^{3}J_{H,H}$ = 3.5, 3.2, 1H)	
3	C_q	–	74.5
4	C_q	–	35.9
5	CH	1.14 –1.12 (m, 1H)	57.8
6	CH_2	1.51 – 1.47 (m, 1H)	22.4
		1.17 – 1.14 (m, 1H)	
7	CH_2	2.04 (dd, $^{2}J_{H,H}$ = 13.6, $^{3}J_{H,H}$ = 11.0, 1H)	35.2
		1.92 (ddd, $^{2}J_{H,H}$ = 14.6, $^{3}J_{H,H}$ = 7.0, 1.3, 1H)	
8	C_q	–	141.1
9	CH	5.44 – 5.40 (m, 1H)	123.0
10	CH_2	1.86 (d, $^{2}J_{H,H}$ = 13.9, 1H)	46.6
		1.61 (dd, $^{2}J_{H,H}$ = 14.2, $^{3}J_{H,H}$ = 8.9, 1H)	
11	C_q	–	42.4
12	CH_3	0.87 (s, 3H)	24.8
13	CH_3	0.83 (s, 3H)	19.0
14	CH_3	1.73 (br s, 3H)	25.7
15	CH_3	0.85 (s, 3H)	20.0
16	CH_3	1.11 (br s, 3H)	23.6

[a] Carbon numbering as shown in Figure S6. [b] Chemical Shifts δ in ppm, coupling constants J in Hertz.

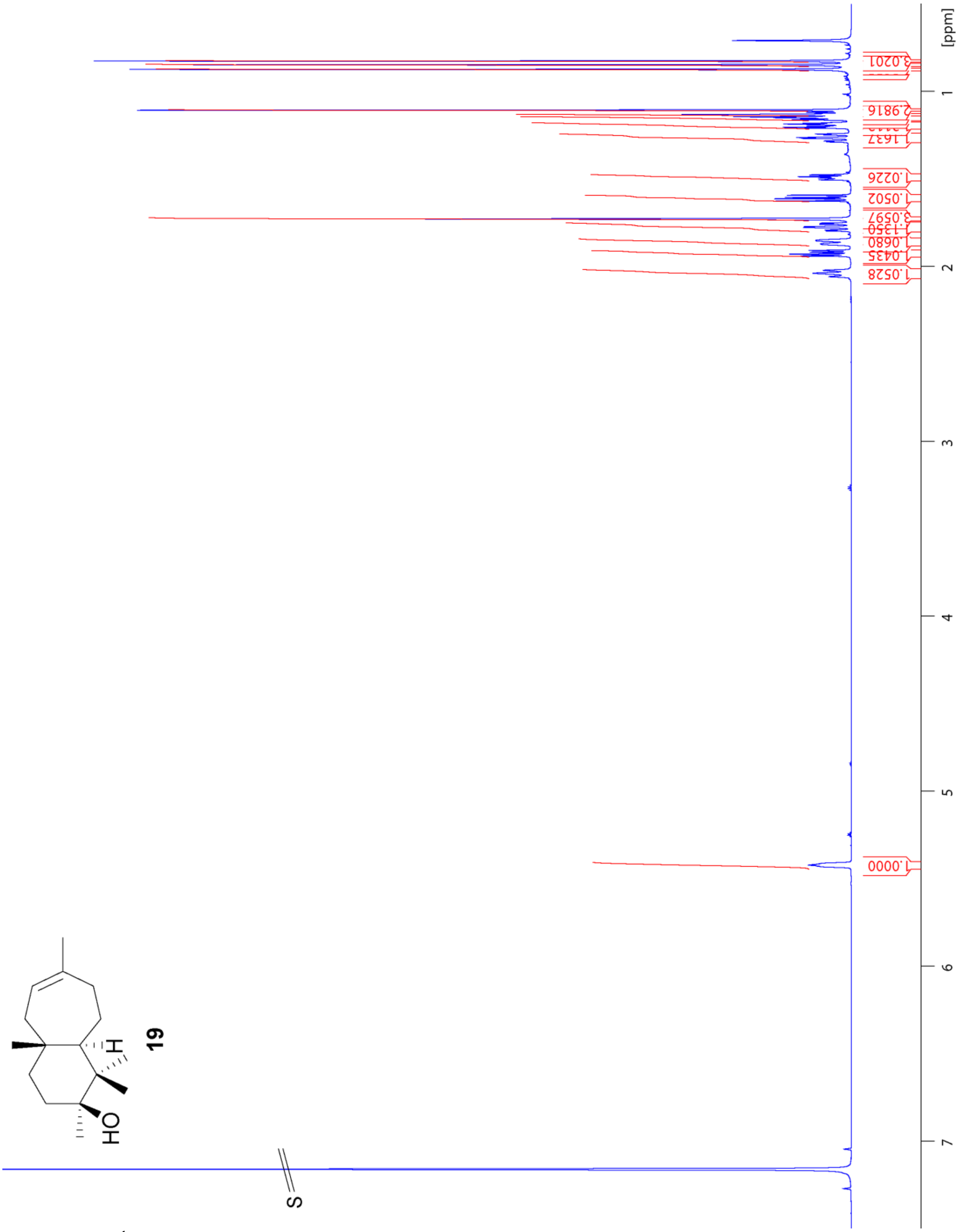

Figure S7. ^{1}H-NMR spectrum (C_6D_6, 700 MHz) of **19**. S indicates solvent peak.

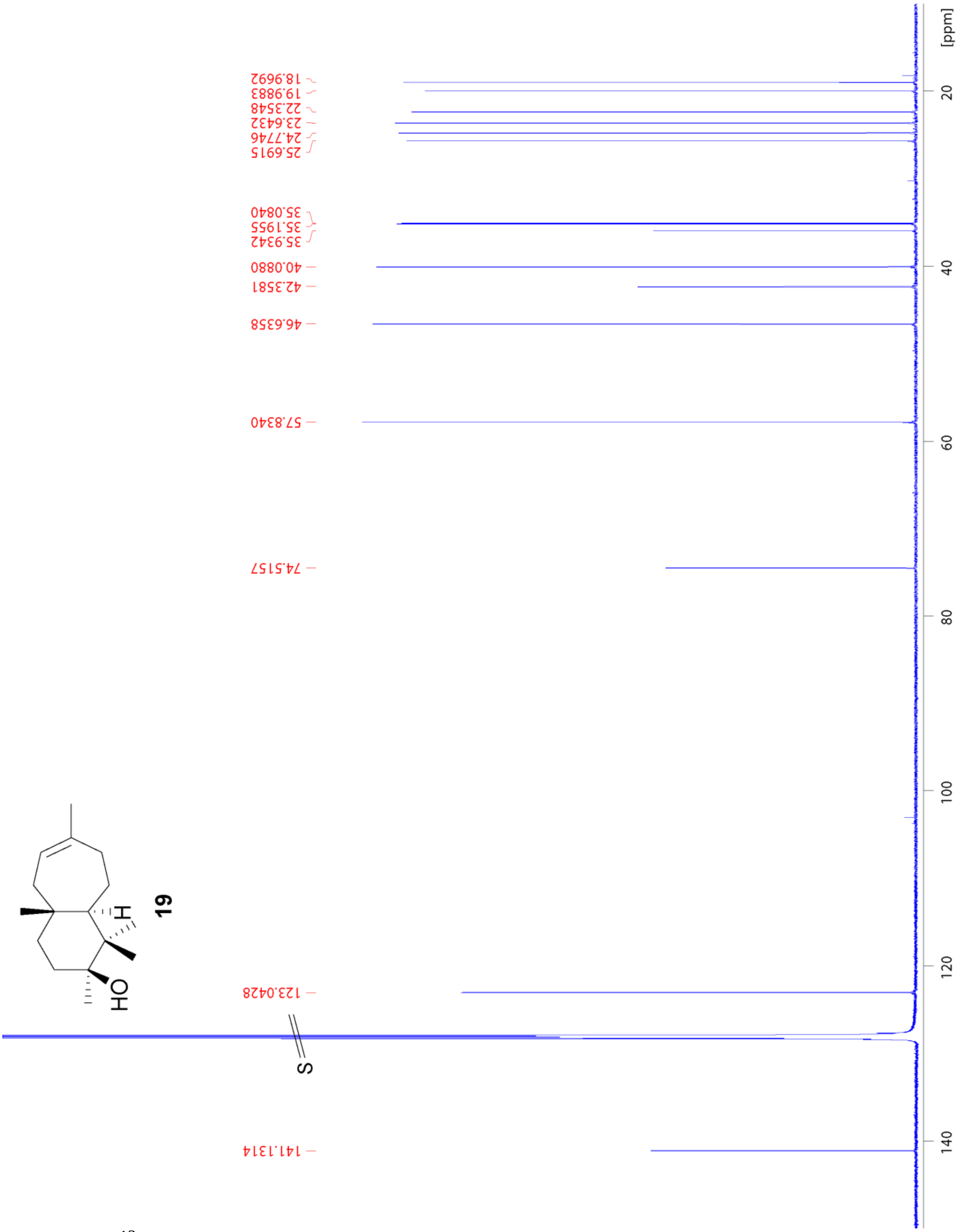

Figure S8. ^{13}C-NMR spectrum (C_6D_6, 175 MHz) of **19**. S indicates solvent peak.

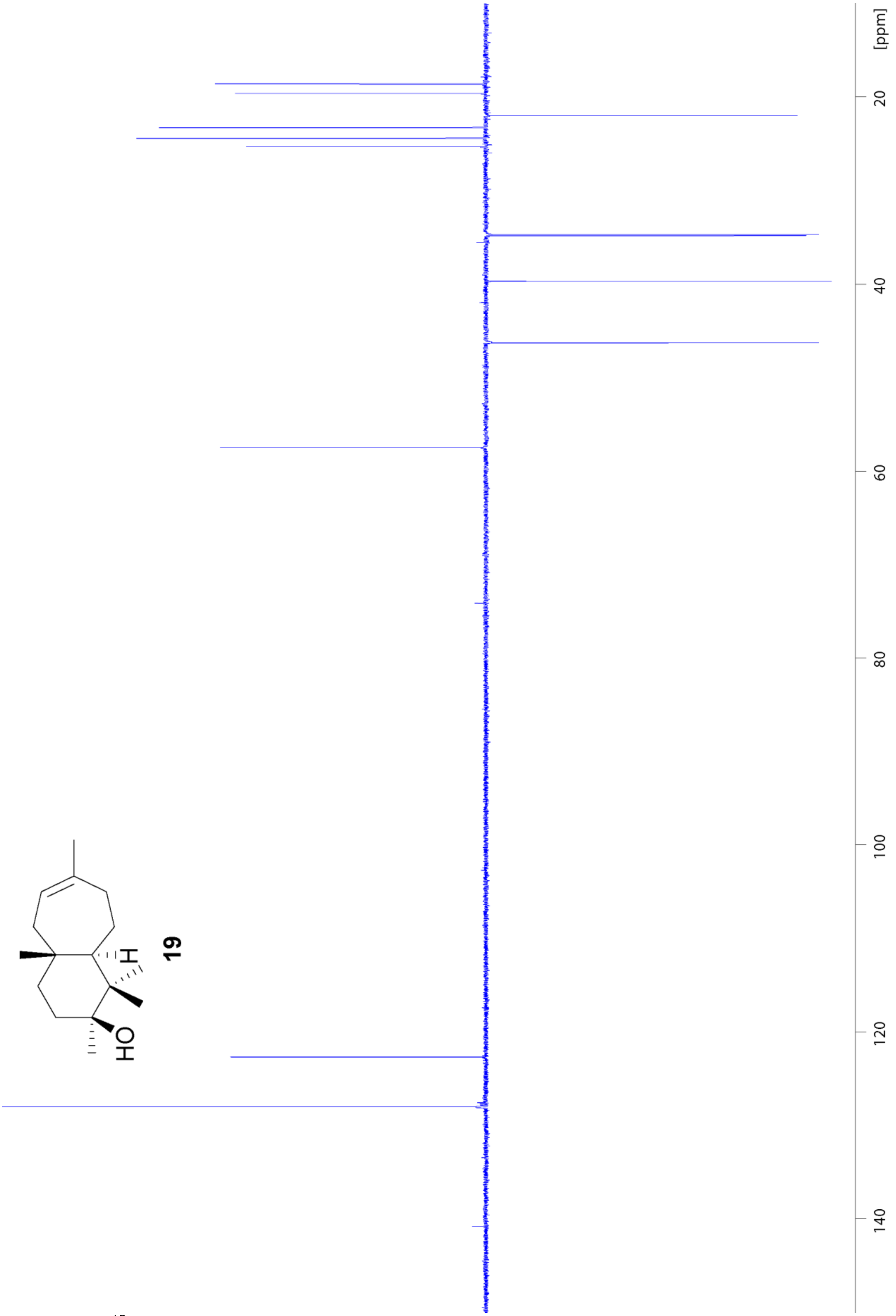

Figure S9. ^{13}C-DEPT135 spectrum (C_6D_6, 175 MHz) of **19**.

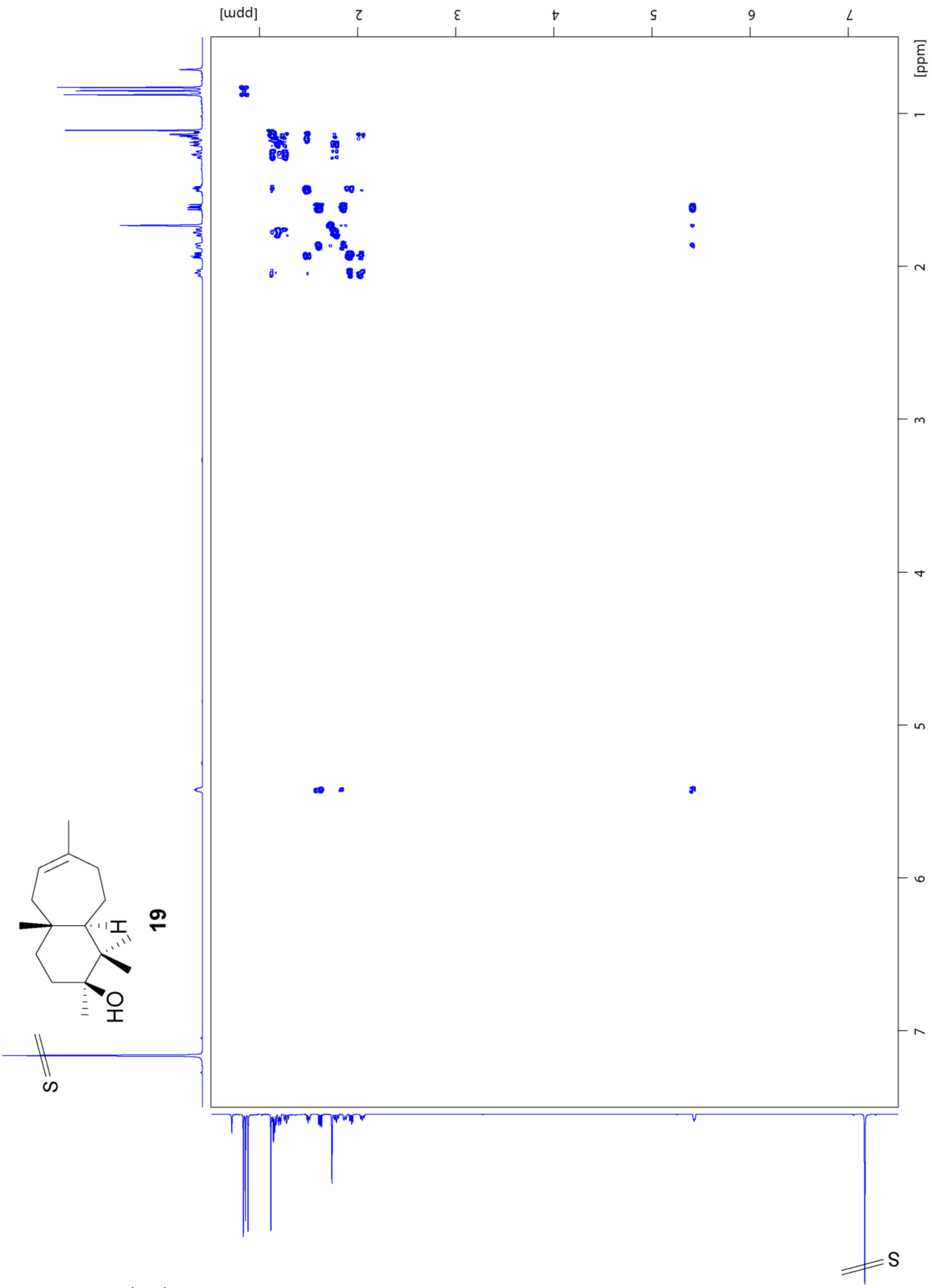

Figure S10. ^{1}H,^{1}H-COSY spectrum (C_6D_6) of **19**. S indicates solvent peaks.

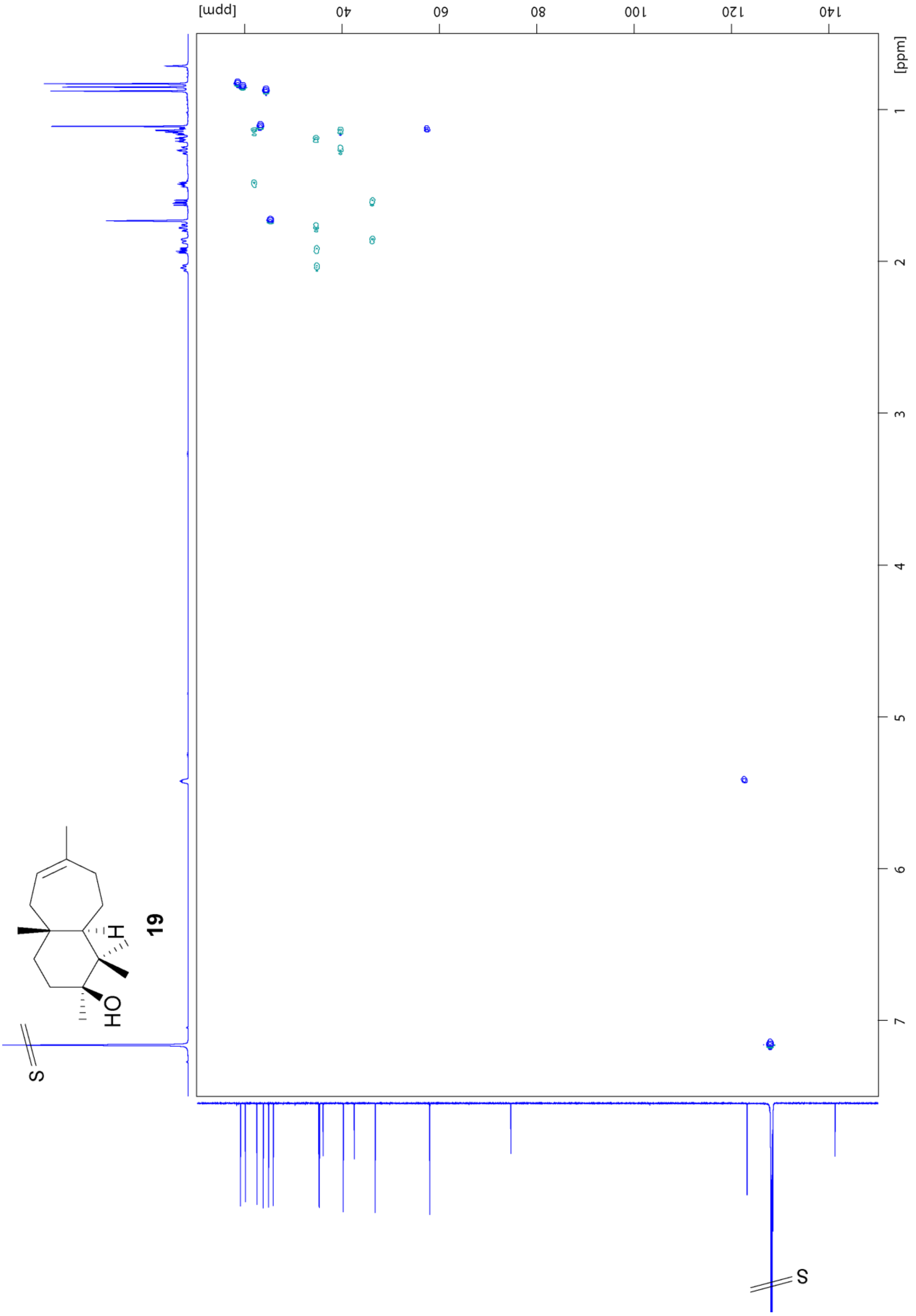

Figure S11. HSQC spectrum (C_6D_6) of **19**. S indicates solvent peaks.

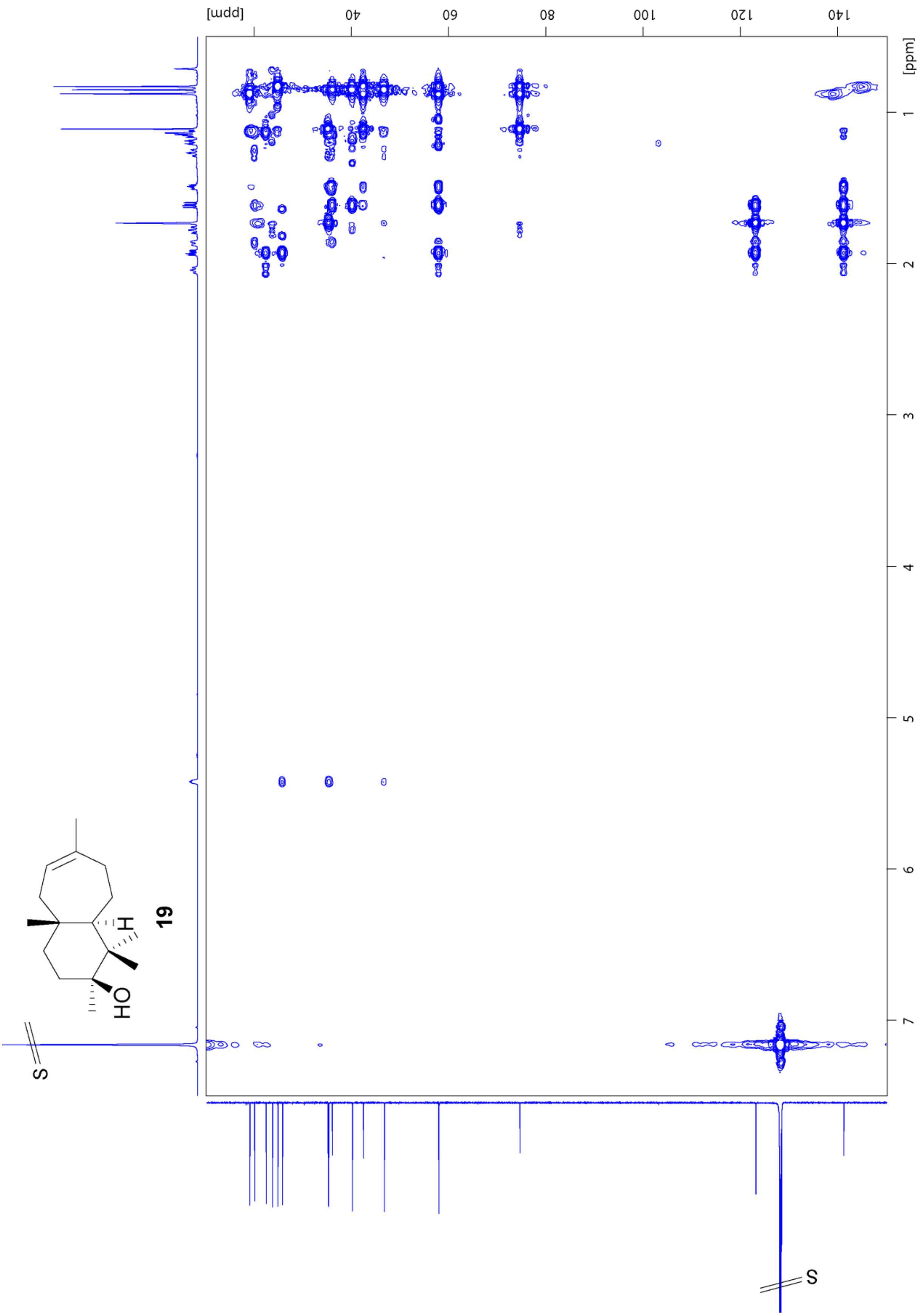

Figure S12. HMBC spectrum (C_6D_6) of **19**. S indicates solvent peaks.

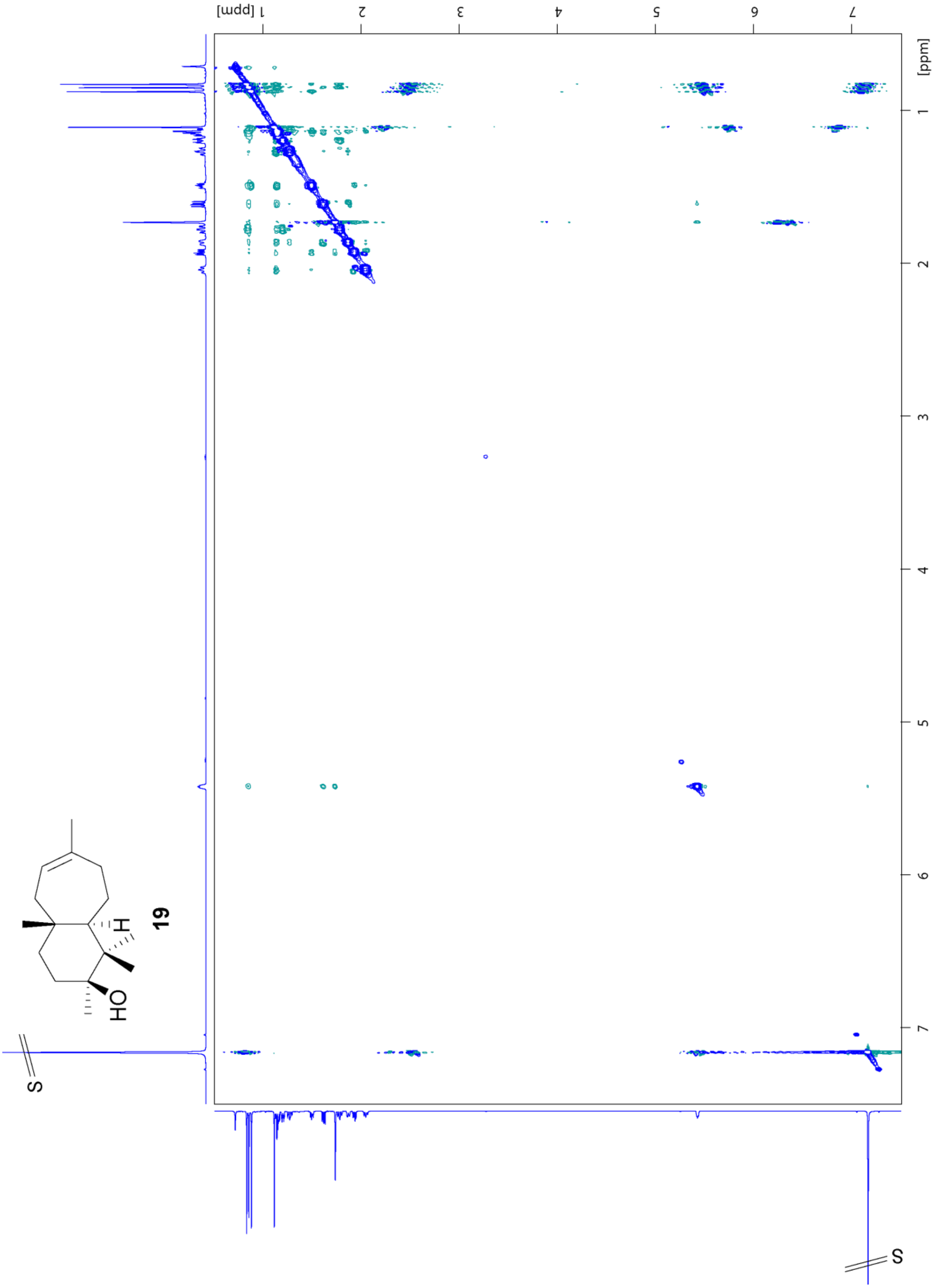

Figure S13. NOESY spectrum (C_6D_6) of **19**. S indicates solvent peaks.

Table S2. NMR data of **20** recorded in C_6D_6.

C[a]		^{1}H[b]	^{13}C[b]
1	C_q	–	44.3
2	CH_2	1.43 – 1.39 (m, 1H)	42.1
		1.38 – 1.33 (m, 1H)	
3	CH_2	1.91 (dd, $^{2}J_{H,H}$ = 13.8, $^{3}J_{H,H}$ = 7.3, 1H)	34.8
		1.37 – 1.33 (m, 3H)	
4	C_q	–	76.3
5	CH	1.75 (d, $^{3}J_{H,H}$ = 12.2, 1H)	56.2
6	CH_2	1.47 (dm, $^{2}J_{H,H}$ = 14.4, 1H)	24.3
		1.31 – 1.28 (m, 1H)	
7	CH_2	2.04 – 2.01 (m, 1H)	35.1
		1.98 (dddd, $^{2}J_{H,H}$ = 15.0, $^{3}J_{H,H}$ = 5.5, 2.5, $^{4}J_{H,H}$ = 1.3, 1H)	
8	C_q	–	138.7
9	CH	5.49 – 5.46 (m, 1H)	123.2
10	CH_2	2.06 – 2.04 (m, 2H)	43.4
11	C_q	–	51.2
12	CH_3	1.00 (s, 3H)	26.0
13	CH_3	1.01 (s, 3H)	26.7
14	CH_3	1.76 (br s, 3H)	27.8
15	CH_3	0.92 (s, 3H)	19.7
16	CH_3	0.85 (s, 3H)	21.7

[a] Carbon numbering as shown in Figure S6. [b] Chemical Shifts δ in ppm, coupling constants J in Hertz.

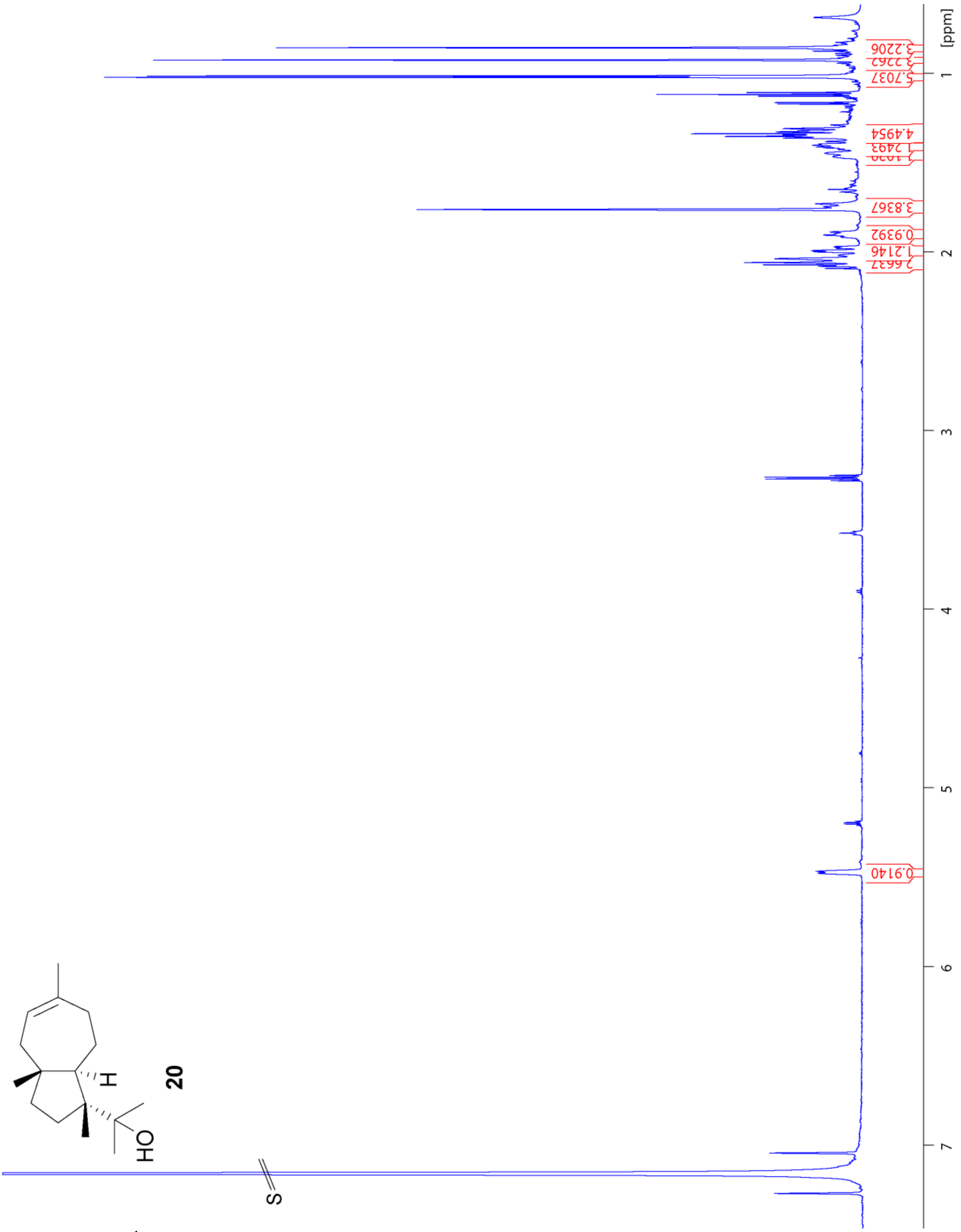

Figure S14. ^{1}H-NMR spectrum (C_6D_6, 700 MHz) of **20**. S indicates solvent peak.

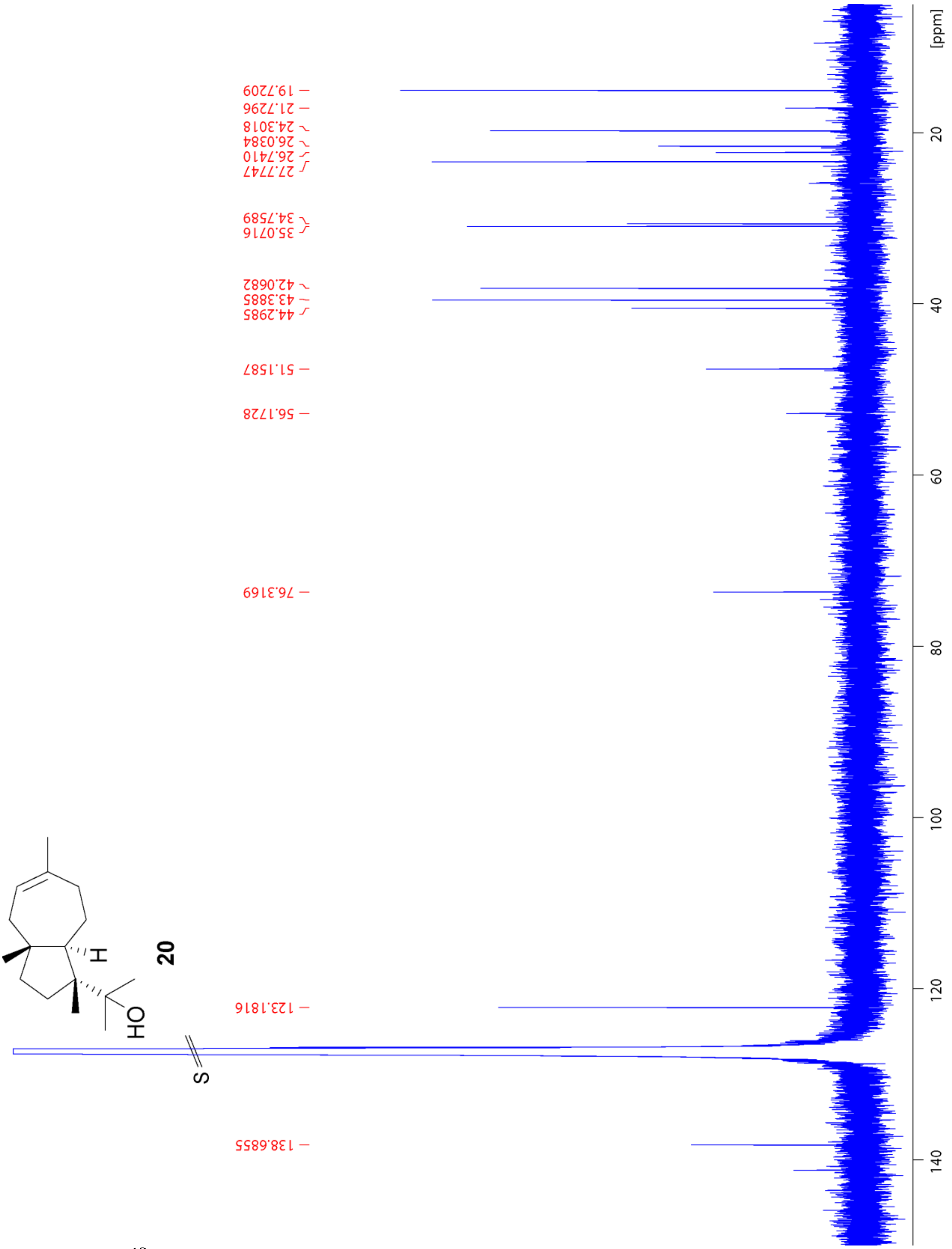

Figure S15. ^{13}C-NMR spectrum (C_6D_6, 175 MHz) of **20**. S indicates solvent peak.

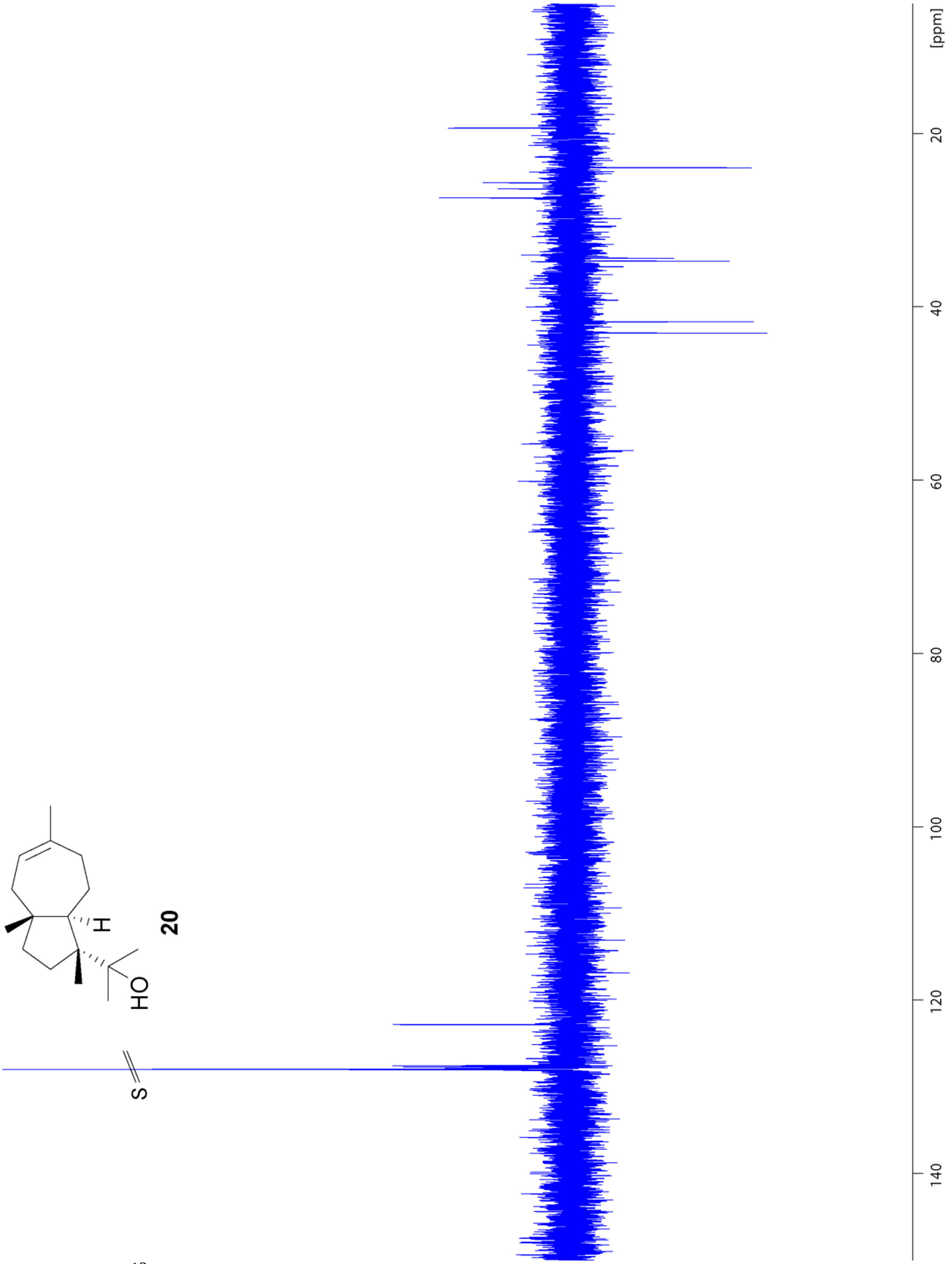

Figure S16. ^{13}C-DEPT135 spectrum (C_6D_6, 175 MHz) of **20**. S indicates solvent peak.

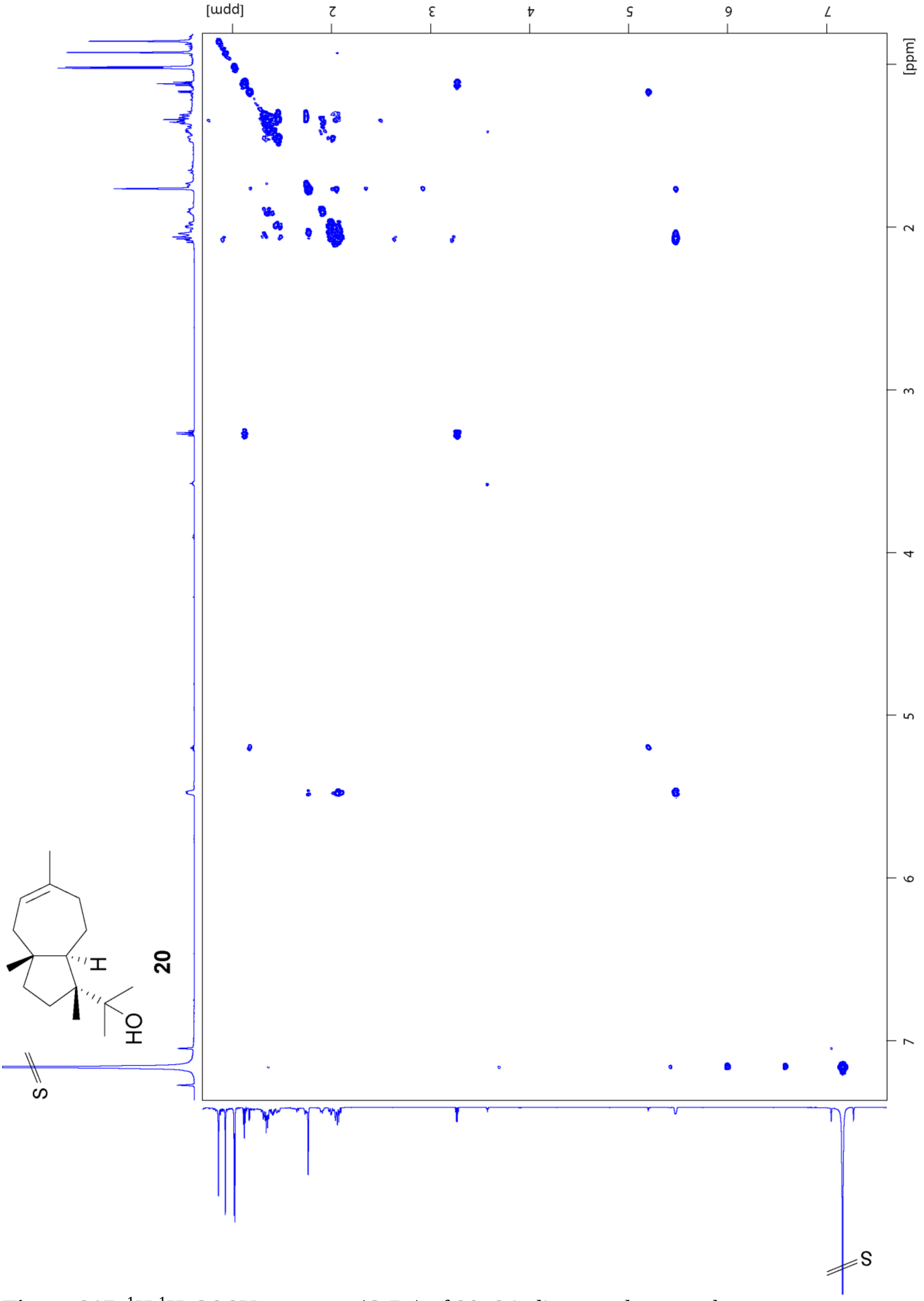

Figure S17. ^{1}H,^{1}H-COSY spectrum (C_6D_6) of **20**. S indicates solvent peaks.

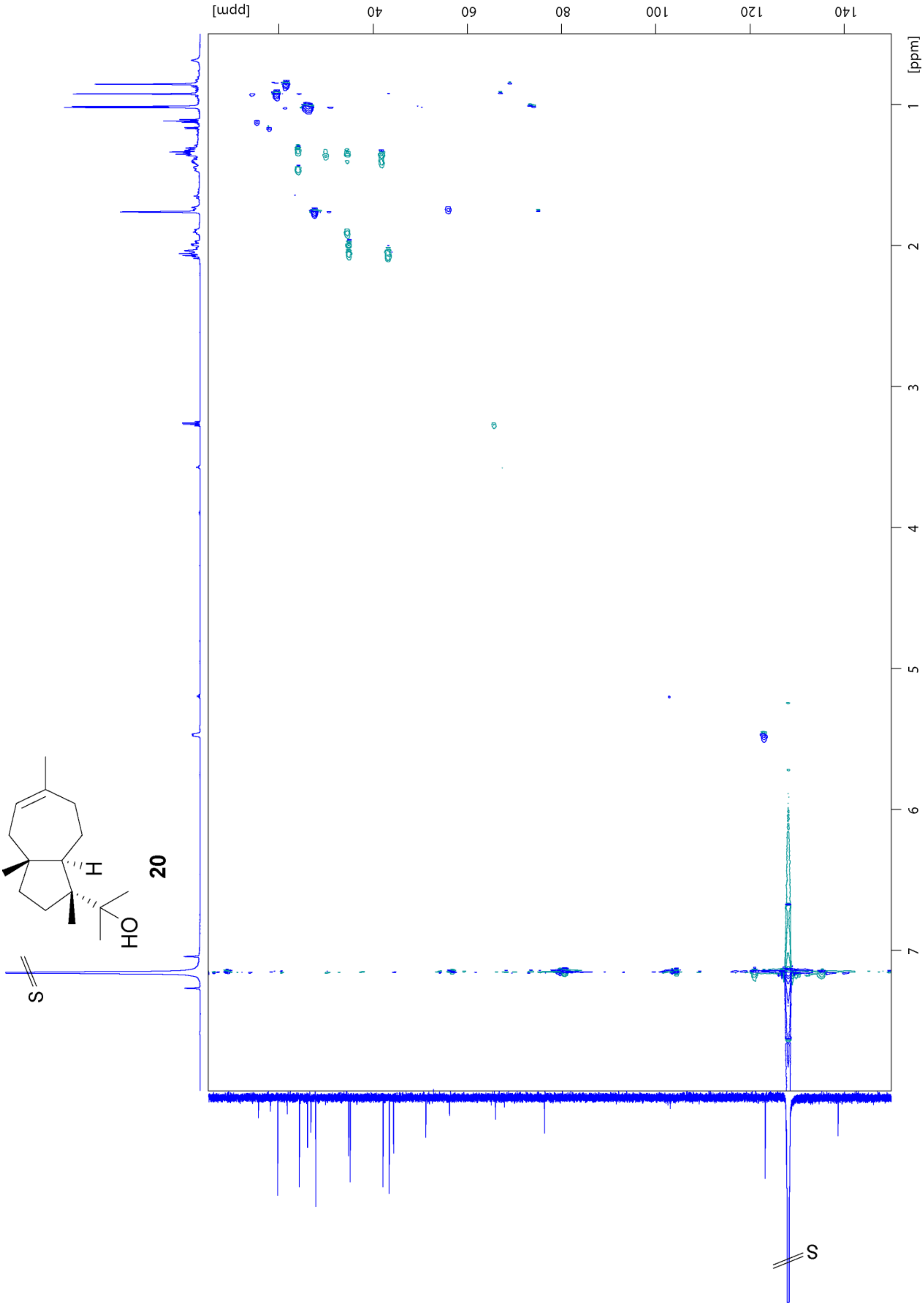

Figure S18. HSQC spectrum (C_6D_6) of **20**. S indicates solvent peaks.

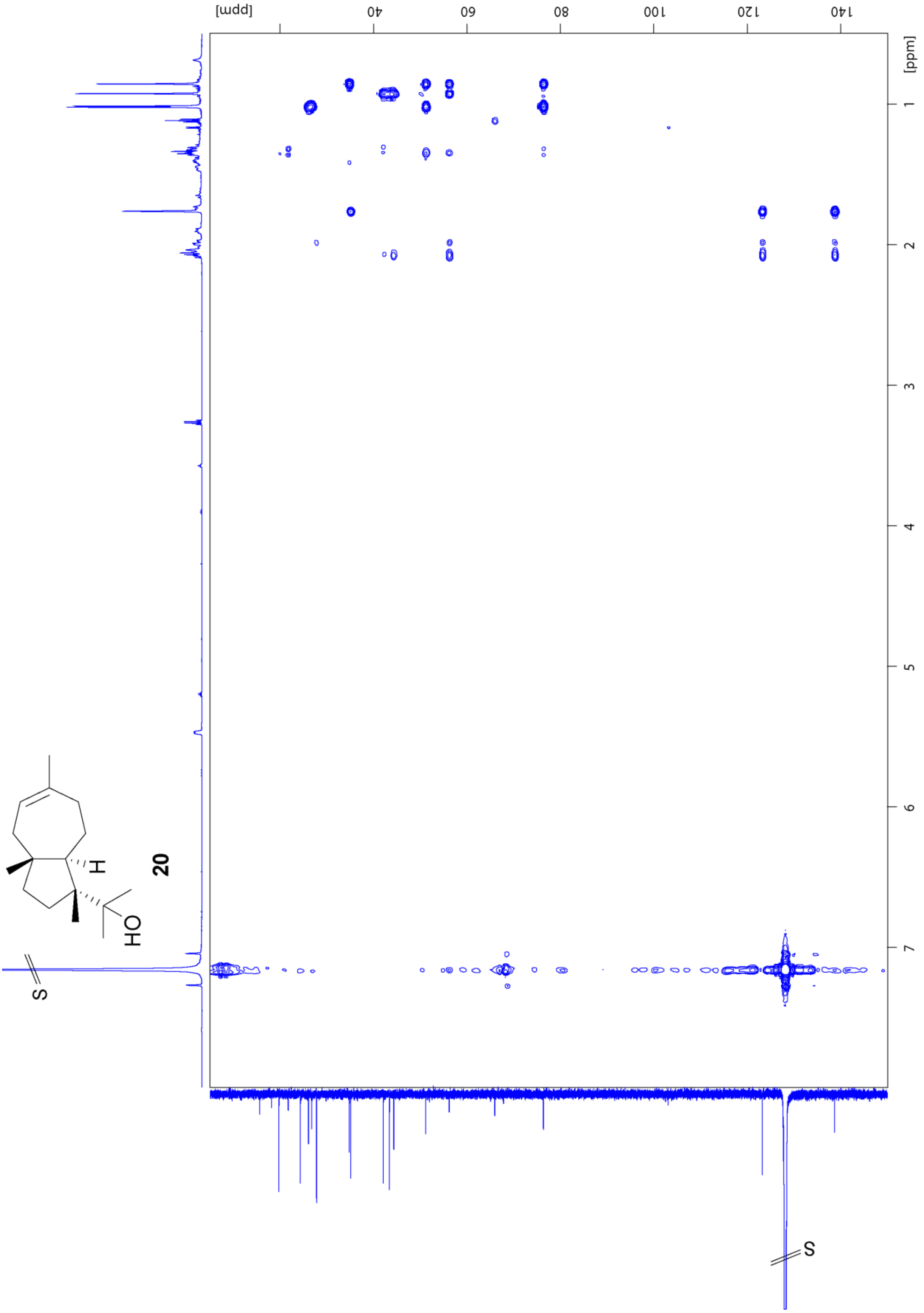

Figure S19. HMBC spectrum (C_6D_6) of **20**. S indicates solvent peaks.

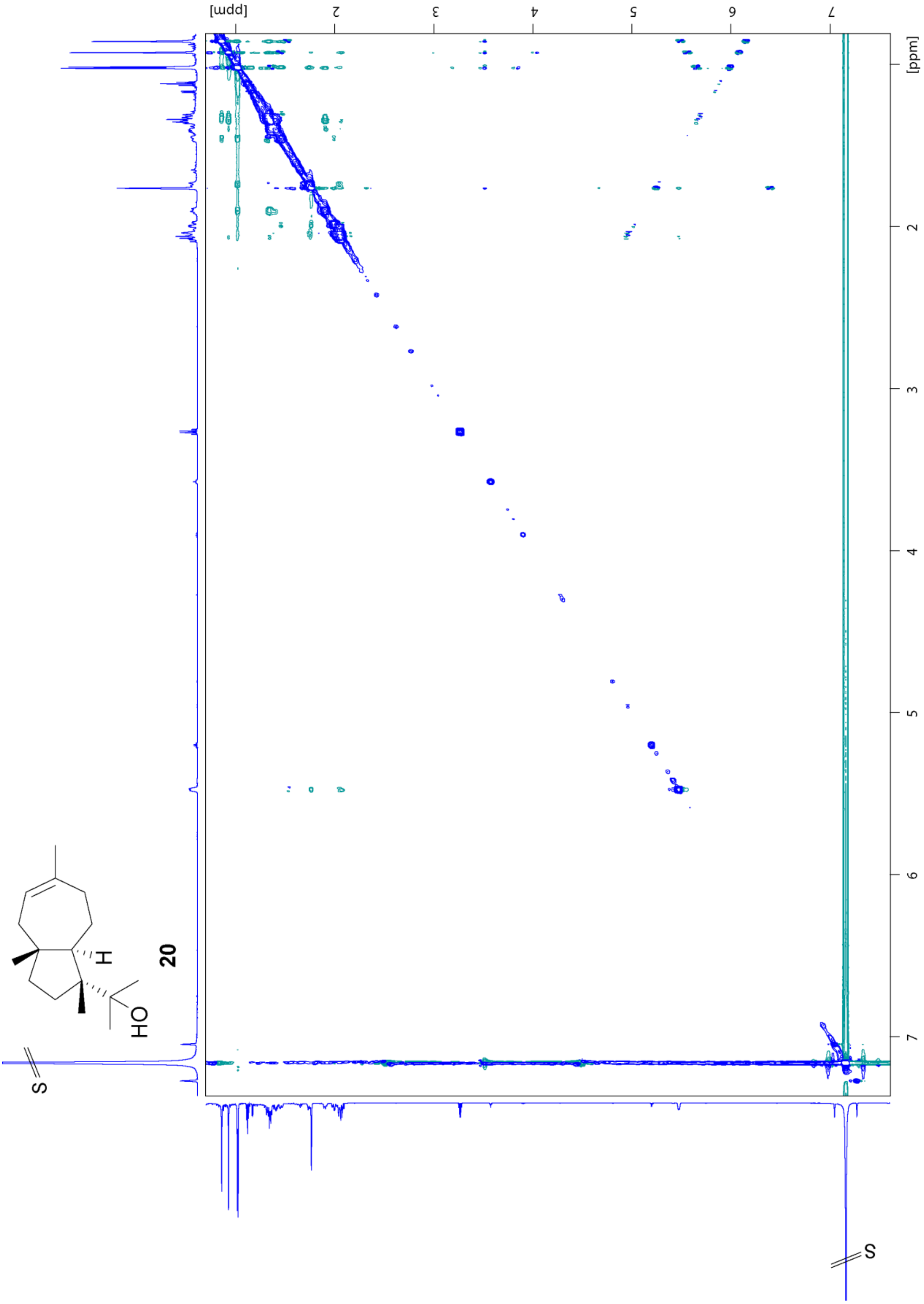

Figure S20. NOESY spectrum (C_6D_6) of **20**. S indicates solvent peaks.

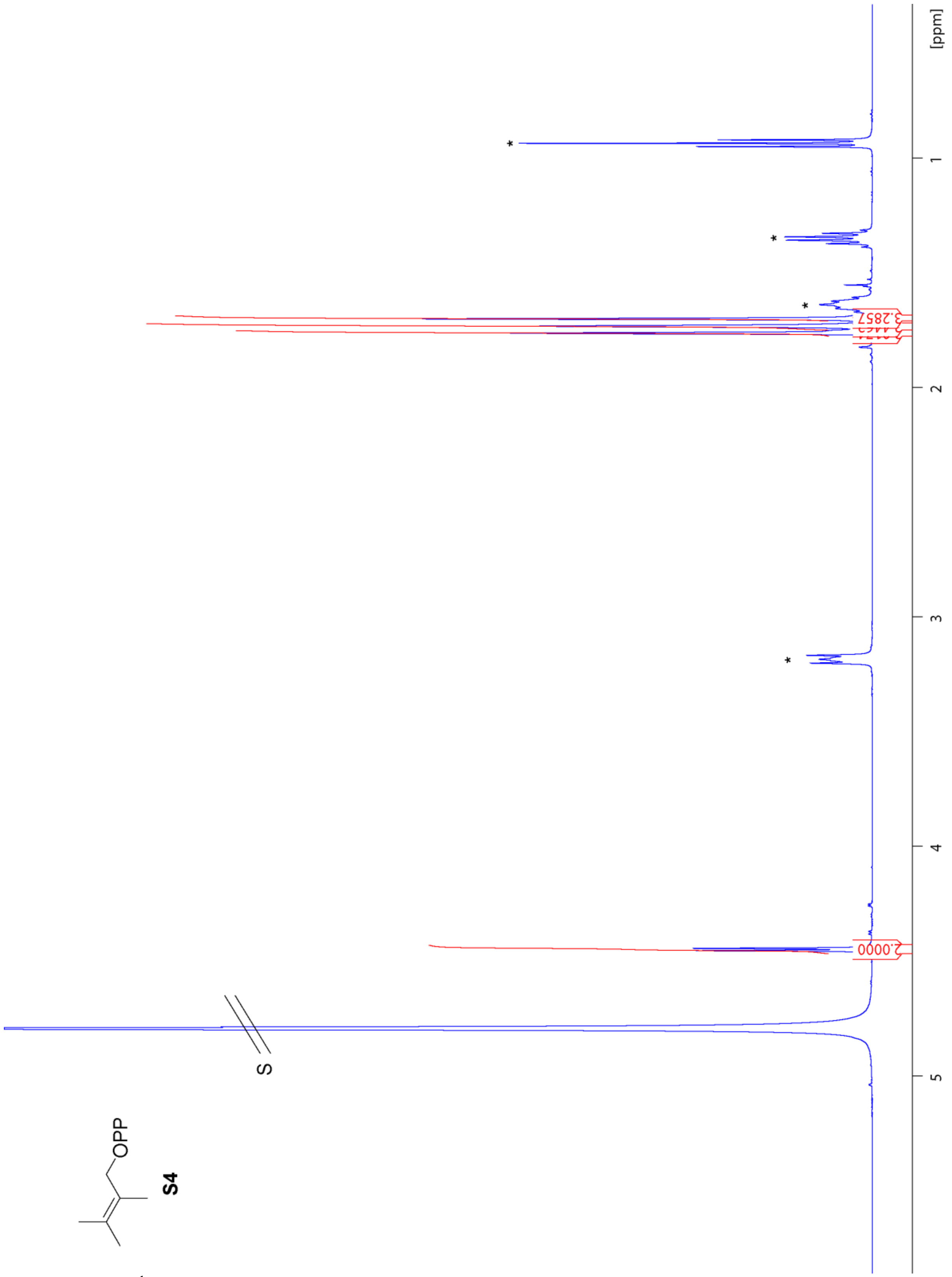

Figure S21. ^{1}H-NMR spectrum (D_2O, 500 MHz) of **S4**. S indicates solvent peak. Asterisks indicate signals from residual tetrabutyl ammonium salt.

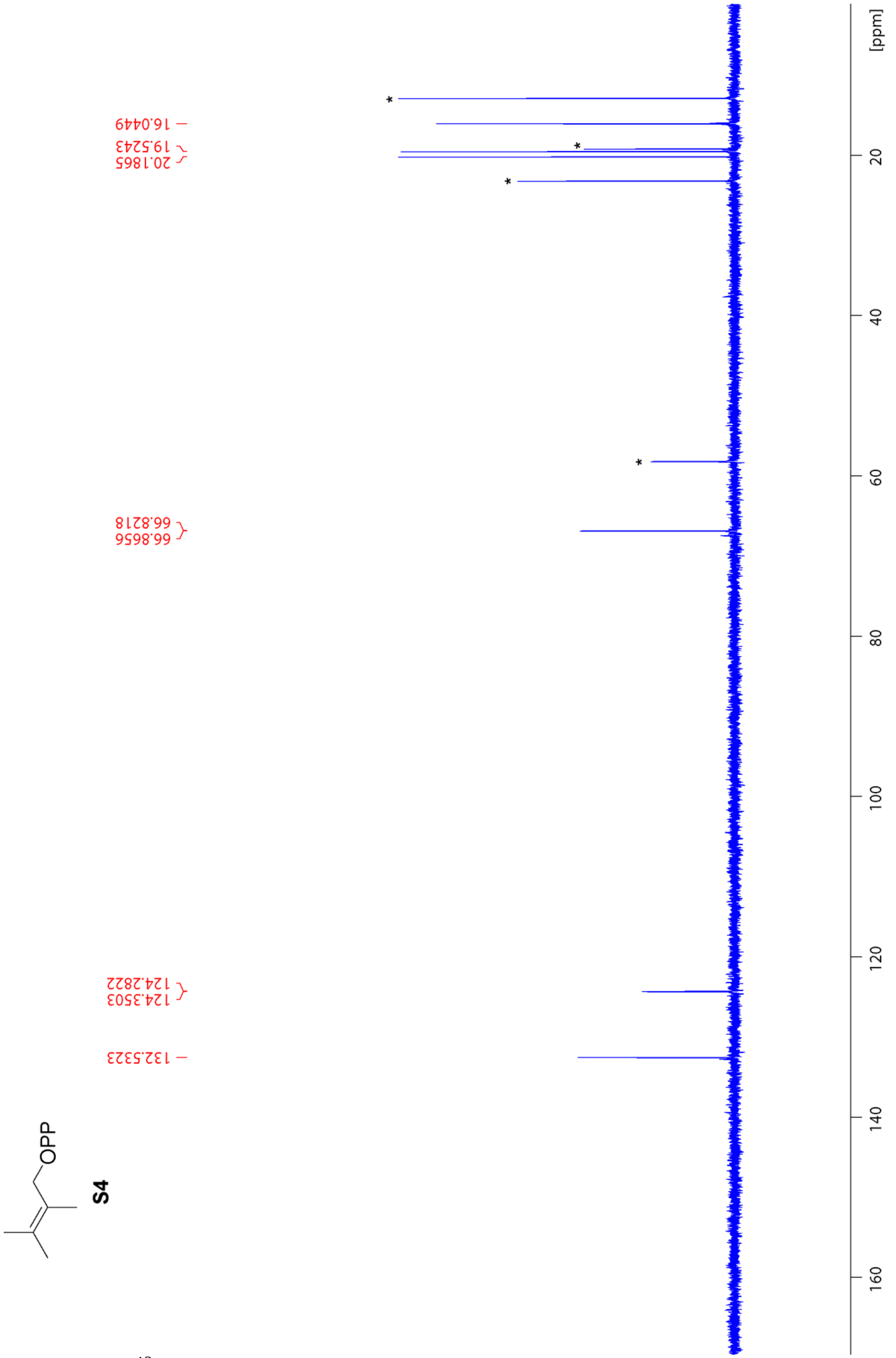

Figure S22. ^{13}C-NMR spectrum (D_2O, 126 MHz) of **S4**. Asterisks indicate signals from residual tetrabutyl ammonium salt.

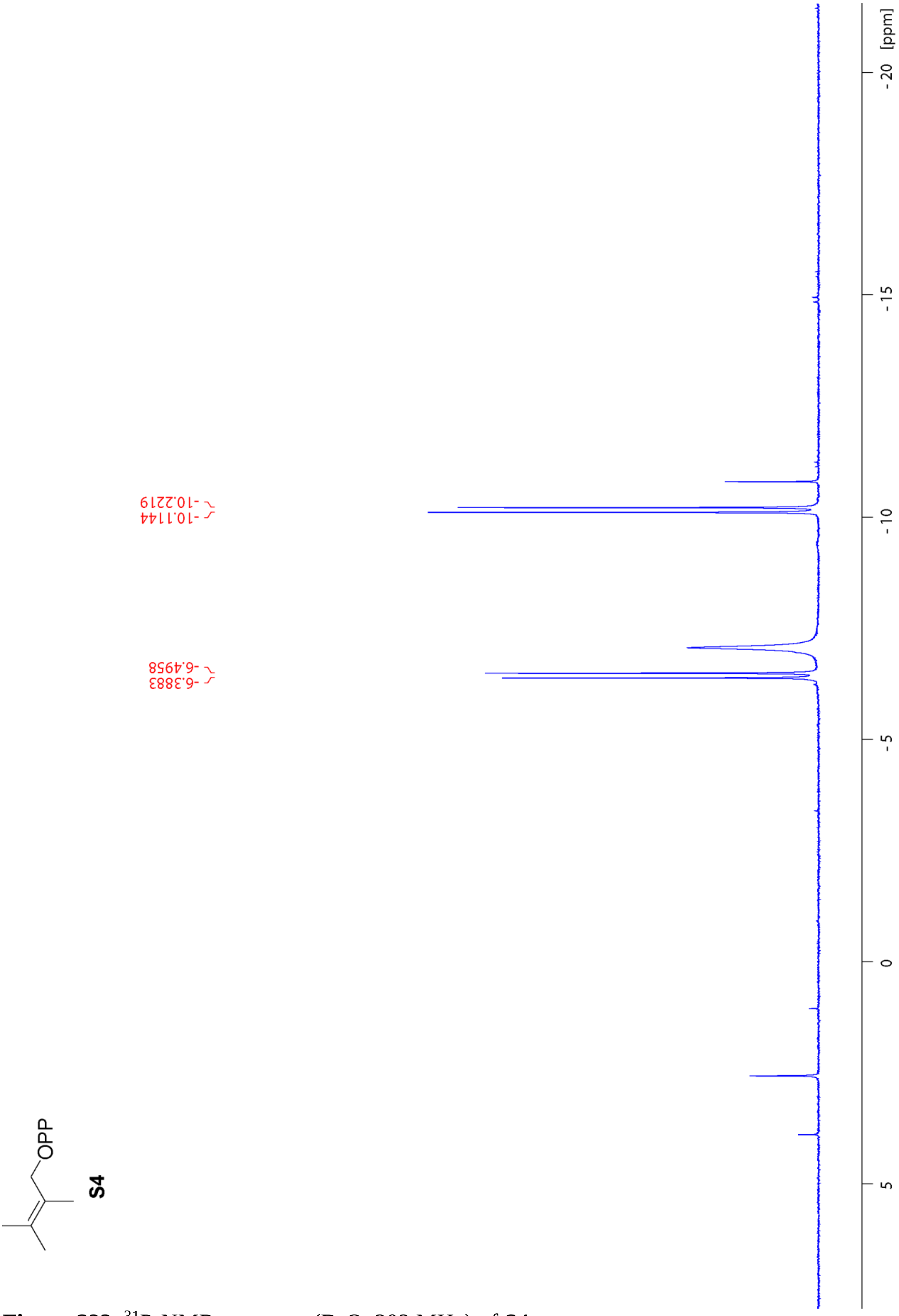

Figure S23. ^{31}P-NMR spectrum (D_2O, 202 MHz) of **S4**.

Isotopic labelling experiments with DcS

For isotopic labelling experiments 2-Me-DMAPP (**S4**) was incubated with FPPS, DcS and stereoselectively deuterated, ^{13}C-labelled IPP-isotopomers as described above. Experiments were extracted with C_6D_6, the extracts were dried with $MgSO_4$ and subjected to NMR.

Table S3. Isotopic labelling experiments performed with DcS and FPPS.

entry	substrates	result shown in
1	2-Me-DMAPP + (*R*)-(1-^{13}C,1-^{2}H)IPP[6]	Figure S24
2	2-Me-DMAPP + (*S*)-(1-^{13}C,1-^{2}H)IPP[6]	Figure S24
3	2-Me-DMAPP + (*E*)-(4-^{13}C,4-^{2}H)IPP[7]	Figure S25
4	2-Me-DMAPP + (*Z*)-(4-^{13}C,4-^{2}H)IPP[7]	Figure S25

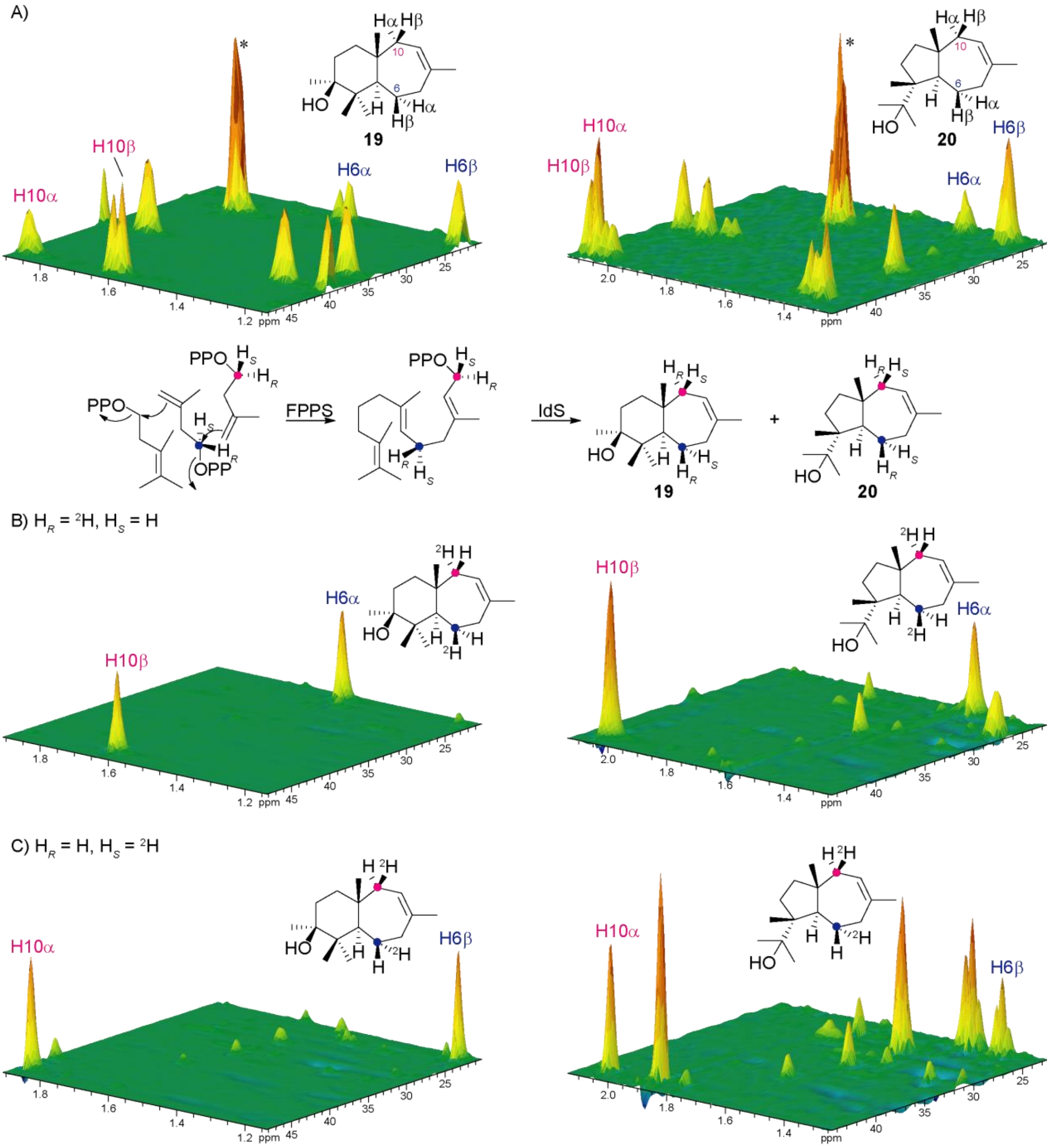

Figure S24. Absolute configuration of **19** and **20**. A) Partial HSQC spectra of purified **19** (left column, asterisks indicate signals for Me groups) and **20** (right column). Spectra resulting from incubation of DcS, FPPS and 2-Me-DMAPP (**S4**) with B) (*R*)-(1-^{13}C,1-^{2}H)IPP and C) (*S*)-(1-^{13}C,1-^{2}H)IPP.

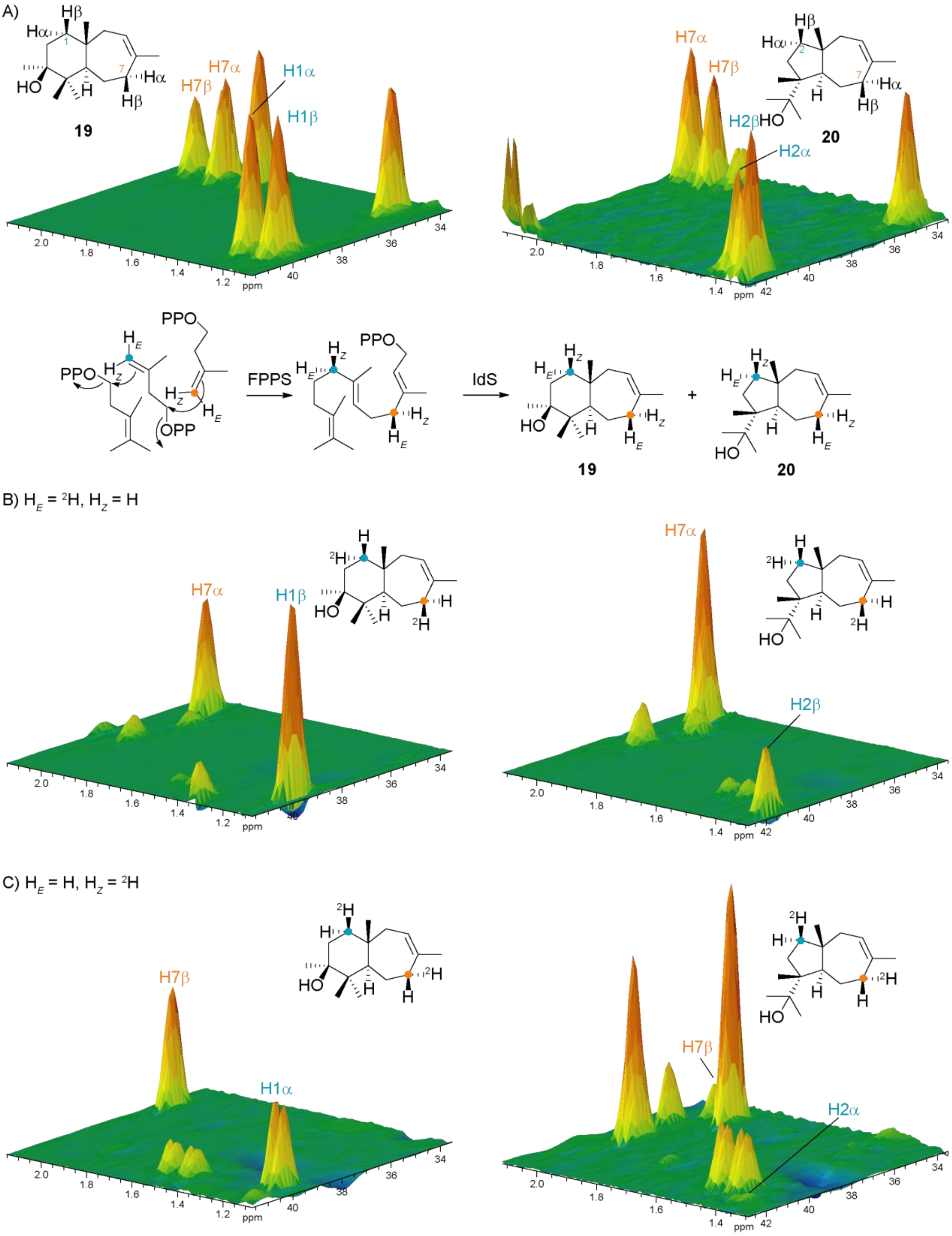

Figure S25. Absolute configuration of **19** and **20**. A) Partial HSQC spectra of purified **19** (left column) and **20** (right column). Spectra resulting from incubation of DcS, FPPS and 2-Me-DMAPP (**S4**) with B) (*E*)-(4-^{13}C,4-^{2}H)IPP and C) (*Z*)-(4-^{13}C,4-^{2}H)IPP.

Synthetic route to 13-desmethyl-FPP (11)

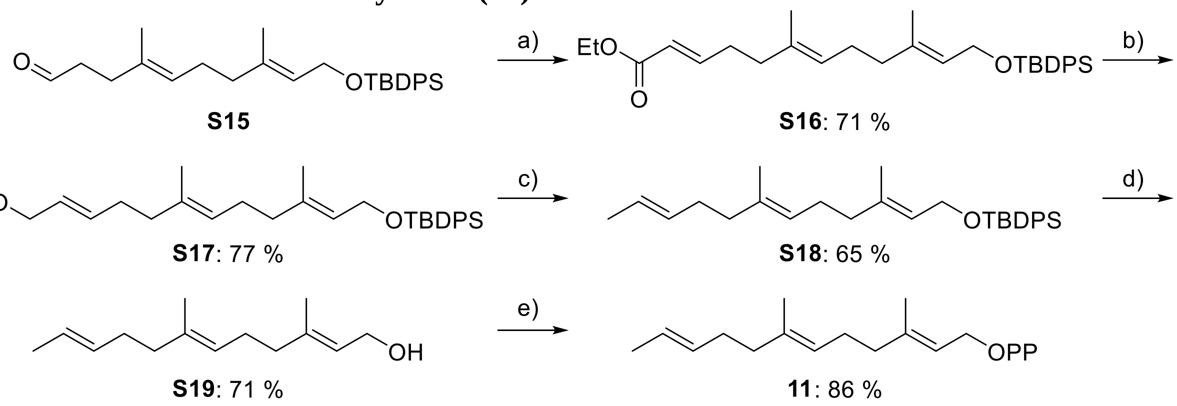

Scheme S2. Synthesis of 13-desmethyl-FPP (**11**). Reaction conditions: a) LDA, triethyl phosphonoacetate, –78 °C to room temperature, overnight; b) D*i*BAl-H, THF, 3 h; c) 1. DMAP, NEt_3, MsCl, DCM, 0 °C, 3 h, 2. LAH, THF, overnight; d) TBAF, THF, 60 h; e) 1. PBr_3, THF, 3 h, 2. $(\mathrm{N}n\mathrm{Bu}_4)_3\mathrm{HP}_2\mathrm{O}_7$, MeCN, overnight.

Synthesis of ethyl (2*E*,6*E*,10*E*)-12-((*tert*-butyldiphenylsilyl)oxy)-6,10-dimethyldodeca-2,6,10-trienoate (S16)

To a suspension of sodium hydride (60% dispersion in mineral oil, 0.223 g, 5.57 mmol, 1.2 eq) in THF (40 mL, –78 °C), a solution of triethyl phosphonoacetate (1.249 g, 5.57 mmol, 1.2 eq) in THF (10 mL) was added. After 30 min aldehyde **S15**[8] (2.02 g, 4.64 mmol, 1.0 eq) was added and the mixture was warmed to room temperature with stirring overnight. Water was added to the reaction mixture and the aqueous phase was extracted with ethyl acetate three times. The organic phases were dried with $MgSO_4$, concentrated under reduced pressure and subjected to column chromatography (cyclohexane/ethyl acetate, 45:1) to yield the desired compound as a colorless oil (1.66 g, 3.29 mmol, 71%). TLC (cyclohexane/ethyl acetate, 30:1): R_f = 0.37. GC (HP5-MS): *I* = 3400. HRMS (ESI) *m*/*z* = 505.3134 (calc. for $[C_{32}H_{45}O_3Si]^+$: 505.3132). EI-MS (70 eV): *m*/*z* (%): 504 (1), 448 (22), 447 (62), 281 (25), 253 (13), 209 (14), 208 (18), 200 (16), 199 (100), 197 (13), 193 (11), 191 (10), 135 (23), 133 (10), 73 (19). ^{1}H-NMR ($CDCl_3$, 300 MHz): 7.73 – 7.69 (m, 4H), 7.47 – 7.36 (m, 6H), 6.96 (dt, $^3J_{H,H}$ = 15.6, 6.8 Hz, 1H), 5.83 (dt, $^3J_{H,H}$ = 15.6 Hz, $^4J_{H,H}$ = 1.4 Hz, 1H), 5.40 (tq, $^3J_{H,H}$ = 6.3, $^4J_{H,H}$ = 1.2 Hz, 1H), 5.16 (tq, $^3J_{H,H}$ = 6.5 Hz, $^4J_{H,H}$ = 1.1 Hz, 1H), 4.24 (d, $^3J_{H,H}$ = 6.2 Hz, 2H), 4.20 (q, $^3J_{H,H}$ = 7.1 Hz, 2H), 2.36 – 2.27 (m, 2H), 2.17 – 2.07 (m, 4H), 2.04 – 1.96 (m, 2H), 1.63 (s, 3H), 1.42 (s, 3H), 1.30 (t, $^3J_{H,H}$ = 7.1 Hz, 3H) ppm. ^{13}C-NMR ($CDCl_3$, 75 MHz): δ = 166.9 (C_q), 149.1 (CH), 137.0 (C_q), 135.8 (4x CH), 134.2 (2x C_q), 133.8 (C_q), 129.6 (2x CH), 127.7 (4x CH), 125.2 (CH), 124.3 (CH), 121.5 (CH), 61.3 (CH_2), 60.3 (CH_2), 39.5 (CH_2), 38.1 (CH_2), 30.9 (CH_2), 27.0 (3x CH_3), 26.4 (CH_2), 19.3 (C_q), 16.5 (CH_3), 16.1 (CH_3), 14.4 (CH_3) ppm. IR (diamond ATR): $\tilde{\nu}$ = 3061 (w), 2931 (w), 2857 (w), 1717 (m), 1655 (w), 1460 (w), 1434 (w), 1375 (w), 1310 (w), 1267 (m), 1194 (m), 1156 (m), 1106 (s), 1045 (s), 911 (m), 855 (w), 822 (m), 781 (w), 733 (s), 699 (s), 611 (m), 497 (s).

Synthesis of (2*E*,6*E*,10*E*)-12-((*tert*-butyldiphenylsilyl)oxy)-6,10-dimethyldodeca-2,6,10-trien-1-ol (S17)

To a cooled (–30 °C) solution of **S16** (1.66 g, 3.29 mmol, 1.0 eq) in THF (50 mL) was added D*i*BAl-H (1 M in hexane, 7.94 mL, 7.94 mmol, 2.4 eq) and the mixture was stirred for 3 h warming to room temperature. A saturated aqueous solution of potassium sodium tartrate was added and the mixture was stirred for 30 min. The aqueous phase was extracted with ethyl acetate three times. The organic layers were combined, dried with $MgSO_4$, concentrated under reduced pressure and purified by column chromatography (cyclohexane/ethyl acetate, 5:1) to

yield the pure alcohol **S17** as a colorless oil (1.17 g, 2.53 mmol, 77%). TLC (cyclohexane/ethyl acetate, 2:1): R_f = 0.42. GC (HP5-MS): *I* = 3277. HRMS (ESI) *m/z* = 485.2843 (calc. for $[C_{30}H_{42}O_2SiNa]^+$: 485.2846). EI-MS (70 eV): *m/z* (%) = 405 (9), 265 (8), 209 (8), 200 (12), 199 (100), 197 (12), 193 (13), 187 (18), 183 (14), 181 (11), 149 (21), 147 (9), 135 (10), 93 (10), 79 (9), 78 (10), 75 (10), 67 (9). ^{1}H-NMR ($CDCl_3$, 700 MHz): δ = 7.70 – 7.68 (m, 4H), 7.41 (tt, $^3J_{H,H}$ = 7.4 Hz, $^4J_{H,H}$ = 1.3 Hz, 2H), 7.39 – 7.36 (m, 4H), 5.70 – 5.61 (m, 2H), 5.38 (tq, $^3J_{H,H}$ = 6.3 Hz, $^4J_{H,H}$ = 1.1 Hz, 1H). 5.12 (tq, $^3J_{H,H}$ = 6.8 Hz, $^4J_{H,H}$ = 1.3 Hz, 1H), 4.22 (d, $^3J_{H,H}$ = 6.5 Hz, 2H), 4.07 (d, $^3J_{H,H}$ = 5.7 Hz, 2H), 2.15 (ddd, $^3J_{H,H}$ = 7.5, 6.9, 6.7 Hz, 2H), 2.10 – 2.03 (m, 4H), 1.98 (dd, $^3J_{H,H}$ = 7.8, 7.1 Hz, 2H), 1.60 (s, 3H), 1.44 (s, 3H), 1.05 (s, 9H) ppm. ^{13}C-NMR ($CDCl_3$, 175 MHz): δ = 137.1 (C_q), 135.8 (4x CH), 134.6 (C_q), 134.2 (2x C_q), 133.2 (CH), 129.6 (2x CH), 129.1 (CH), 127.7 (4x CH), 124.6 (CH), 124.2 (CH), 64.0 (CH_2), 61.3 (CH_2), 39.6 (CH_2), 39.3 (CH_2), 30.9 (CH_2), 27.0 (3x CH_3), 26.4 (CH_2), 19.3 (C_q), 16.5 (CH_3), 16.2 (CH_3) ppm. IR (diamond ATR): $\tilde{\nu}$ = 3312 (br w), 2921 (s), 2852 (m), 1667 (w), 1442 (m), 1380 (m), 1234 (w), 1182 (w), 999 (s), 965 (s), 839 (w), 780 (w), 744 (w), 591 (m), 475 (m).

Synthesis of *tert*-butyl(((2*E*,6*E*,10*E*)-3,7-dimethyldodeca-2,6,10-trien-1-yl)oxy)diphenylsilane (S18)

To a cooled (0 °C) solution of **S17** (1.17 g, 2.53 mmol, 1.0 eq) in dichloromethane (30 mL) were added 4-dimethylamino pyridine (0.62 g, 5.06 mmol, 2.0 eq), and triethylamine (0.7 mL, 5.06 mmol, 2.0 eq), and mesyl chloride (0.39 mL, 5.06 mmol, 2.0 eq) was added dropwise. After 45 min another portion of triethylamine and mesyl chloride were added and the mixture was stirred for 1 h at 0 °C and for 2 h at room temperature. The mixture was directly poured into ice-water and the aqueous layer was extracted six times with *n*-hexane. The combined organic layers were washed subsequently with hydrochloric acid (5%), saturated $NaHCO_3$ solution and brine, dried with $MgSO_4$ and concentrated under reduced pressure. The crude extract was directly transferred to a suspension of $LiAlH_4$ (1.76 g, 46.3 mmol, 18.3 eq) and stirred overnight at room temperature. An ethyl acetate/water mixture was added carefully to the reaction mixture to remove excess of $LiAlH_4$. Saturated aqueous NH_4Cl solution was added, the resulting precipitate was filtered off and the aqueous phase was extracted with *n*-hexane four times. The combined organic layers were dried over $MgSO_4$, concentrated under reduced pressure and subjected to column chromatography (cyclohexane/ethyl acetate, 20:1) to yield **S15** as a colorless oil (0.739 g, 1.65 mmol, 65%). TLC (cyclohexane/ethyl acetate, 10:1): R_f = 0.74. GC (HP5-MS): *I* = 2957. HRMS (EI) *m/z* = 389.2296 (calc. for $[C_{30}H_{42}OSi – C_4H_9]^+$: 389.2295). EI-MS (70 eV): *m/z* (%) = 389 (9), 311 (7), 201 (18), 200 (65), 199 (100), 197 (18), 189 (10), 188 (7), 181 (14), 135 (18), 121 (8), 81 (10), 77 (12), 67 (7), 57 (7), 55 (41), 41 (7). ^{1}H-NMR ($CDCl_3$, 400 MHz): δ = 7.71 – 7.66 (m, 4H), 7.44 – 7.35 (m, 6H), 5.43 – 5.35 (m, 3H), 5.11 (t, $^3J_{H,H}$ = 6.6 Hz, 1H), 4.22 (d, $^3J_{H,H}$ = 6.2 Hz, 2H), 2.28 (s, 1H), 2.11 – 2.03 (m, 4H), 2.03 – 1.95 (m, 4H), 1.63 (d, $^3J_{H,H}$ = 3.4 Hz, 3H), 1.59 (s, 3H), 1.44 (s, 3H), 1.04 (s, 9H) ppm. ^{13}C-NMR ($CDCl_3$, 100 MHz): 137.1 (C_q), 136.1 (4x CH), 135.0 (C_q), 134.6 (2x C_q), 131.5 (CH), 129.9 (2x CH), 127.1 (4x CH), 124.9 (CH), 124.8 (CH), 124.6 (CH), 61.6 (CH_2), 40.2 (CH_2), 39.9 (CH_2), 31.8 (CH_2), 27.2 (CH_2), 27.1 (3x CH_3), 26.7 (CH_2), 19.5 (C_q), 18.1 (CH_3), 16.3 (CH_3), 16.1 (CH_3) ppm. δ = IR (diamond ATR): $\tilde{\nu}$ = 3061 (w), 2928 (m), 2855 (m), 1957 (w), 1893 (w), 1825 (w), 1667 (w), 1590 (w), 1459 (w), 1433 (m), 1384 (w), 1261 (w), 1190 (w), 1108 (s), 1053 (s), 966 (m), 935 (w), 851 (w), 820 (m), 781 (m), 738 (m), 698 (s), 611 (s), 496 (s) cm^{-1}.

Synthesis of (2*E*,6*E*,10*E*)-3,7-dimethyldodeca-2,6,10-trien-1-ol (S19)

To a solution of **S18** (0.69 g, 1.54 mmol, 1.0 eq) in THF (15 mL, 0 °C) was added tetra-*n*-butylammonium fluoride solution (1 M in THF, 1.85 mL, 1.85 mmol, 1.2 eq) and the mixture was stirred 60 h at room temperature. Water was added and the aqueous phase was extracted with diethyl ether three times. The organic layers were dried with $MgSO_4$, concentrated under reduced pressure and purified by column chromatography (cyclohexane/ethyl acetate, 5:1) to give the alcohol as colorless liquid (0.23 g, 1.09 mmol, 71%). TLC (cyclohexane/ethyl acetate, 2:1): R_f = 0.34. GC (HP5-MS): *I* = 1639. HRMS (APCI) *m*/*z* = 209.1899 (calc. for $[C_{14}H_{25}O]^+$: 209.1900). EI-MS (70 eV): *m*/*z* (%): 193 (2), 177 (66), 123 (10), 109 (9), 107 (17), 95 (14), 93 (29), 91 (15), 81 (91), 80 (10), 79 (18), 77 (11), 49 (15), 48 (15), 67 (35), 57 (11), 55 (100), 53 (19), 43 (11), 41 (38), 38 (21). ^{1}H-NMR ($CDCl_3$, 500 MHz): 5.44 – 5.39 (m, 3H), 5.10 (tq, $^3J_{H,H}$ = 6.8 Hz, $^4J_{H,H}$ = 1.3 Hz, 1H), 4.15 (d, $^3J_{H,H}$ = 6.9 Hz, 2H), 2.14 – 2.08 (m, 2H), 2.07 – 2.03 (m, 4H), 2.02 – 1.98 (m, 2H), 1.68 (s, 3H), 1.63 (dm, $^3J_{H,H}$ = 4.7 Hz, 3H), 1.59 (s, 3H) ppm. ^{13}C-NMR ($CDCl_3$, 125 MHz): δ = 140.0 (C_q), 135.3 (C_q), 131.3 (CH), 124.9 (CH), 124.0 (CH), 123.4 (CH), 59.5 (CH_2), 39.8 (CH_2), 39.7 (CH_2), 31.4 (CH_2), 26.4 (CH_2), 18.1 (CH_3), 16.4 (CH_3), 16.1 (CH_3) ppm. IR (diamond ATR): $\tilde{\nu}$ = 3348 (br w), 3062 (w), 2929 (m), 2856 (m), 1776 (w), 1667 (w), 1460 (w), 1431 (m), 1384 (w), 1260 (w), 1190 (w), 1107 (s), 1054 (s), 1003 (m), 972 (m), 910 (w), 858 (w), 821 (m), 779 (s), 698 (s), 610 (m), 496 (s).

Synthesis of 13-desmethyl-FPP (11)

To a solution of alcohol **S19** (0.23 g, 1.09 mmol, 1.0 eq) in THF (10 mL, 0 °C) was added PBr_3 (0.12 g, 0.44 mmol, 0.4 eq) and the mixture was stirred 3 h at room temperature. The mixture was directly poured into ice-water and the aqueous layer was extracted twice with *n*-hexane. The organic layers were dried over $MgSO_4$, concentrated under reduced pressure, and diluted with acetonitrile (5 mL). The crude product was then added to a solution of tris(tetra-*n*-butylammonium) hydrogen pyrophosphate (1.08 g, 1.20 mmol, 1.1 eq) in acetonitrile (5 mL) and the mixture was stirred overnight. The acetonitrile was removed under reduced pressure and the residue was directly loaded onto a DOWEX® 50WX8 ion exchange column (NH_4^+ form, pH ~ 7.0) and the desired compound was eluted slowly with 1.5 CV of NH_4HCO_3 buffer (0.25 M, 5% *i*PrOH). The eluate was lyophilized to yield the pure product as an off-white powder (0.395 g, 0.94 mmol, 86%). ^{1}H-NMR (D_2O, 500 MHz): δ = 5.44 – 5.33 (m, 3H), 5.13 – 5.06 (m, 1H), 4.39 (br s, 2H), 2.11 – 1.87 (m, 8H), 1.64 (s, 3H), 1.55 – 1.49 (m, 6H) ppm. ^{13}C-NMR (D_2O, 125 MHz): δ = 142.8 (C_q), 136.7 (C_q), 131.6 (CH), 125.8 (CH), 124.4 (CH), 120.0 (d, $^3J_{C,P}$ = 8.6 Hz, CH), 62.5 (d, $^2J_{C,P}$ = 5.3 Hz, CH_2), 38.9 (CH_2), 30.3 (CH_2), 25.6 (CH_2), 22.7 (CH_2), 17.3 (CH_3), 15.7 (CH_3), 15.3 (CH_3) ppm. ^{31}P-NMR (D_2O, 200 MHz): δ = –6.5 (d, $^2J_{P,P}$ = 22.6 Hz), –10.2 (d, $^2J_{P,P}$ = 22.0 Hz) ppm. IR (diamond ATR): $\tilde{\nu}$ = 3018 (m), 2963 (m), 2921 (m), 2852 (s), 2123 (w), 1888 (w), 1672 (w), 1442 (m), 1199 (m), 1087 (s), 1024 (m), 960 (m), 913 (s), 809 (w), 712 (w), 518 (s) cm^{-1}.

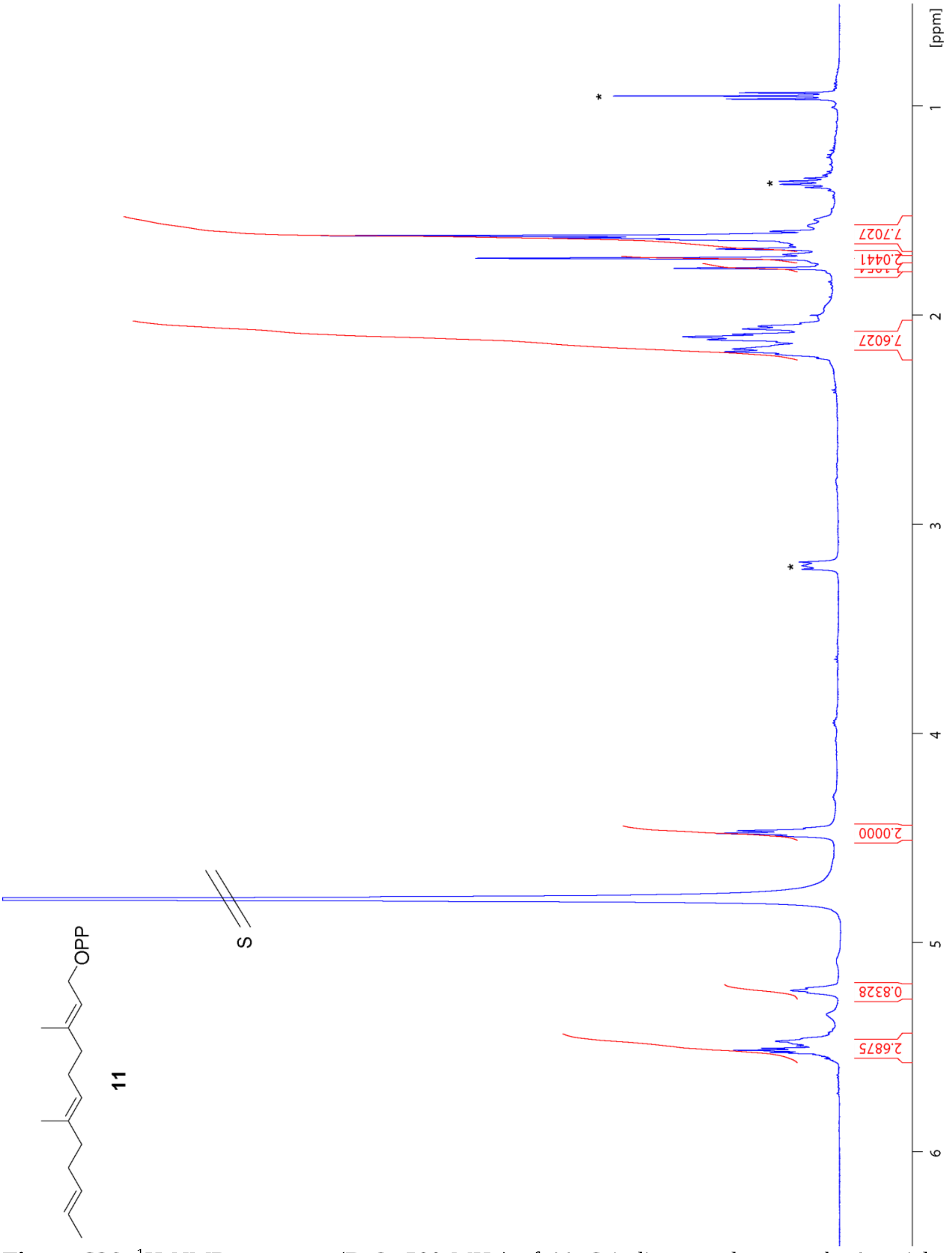

Figure S26. ^{1}H-NMR spectrum (D_2O, 500 MHz) of **11**. S indicates solvent peak. Asterisks indicate signals from residual tetrabutyl ammonium salt.

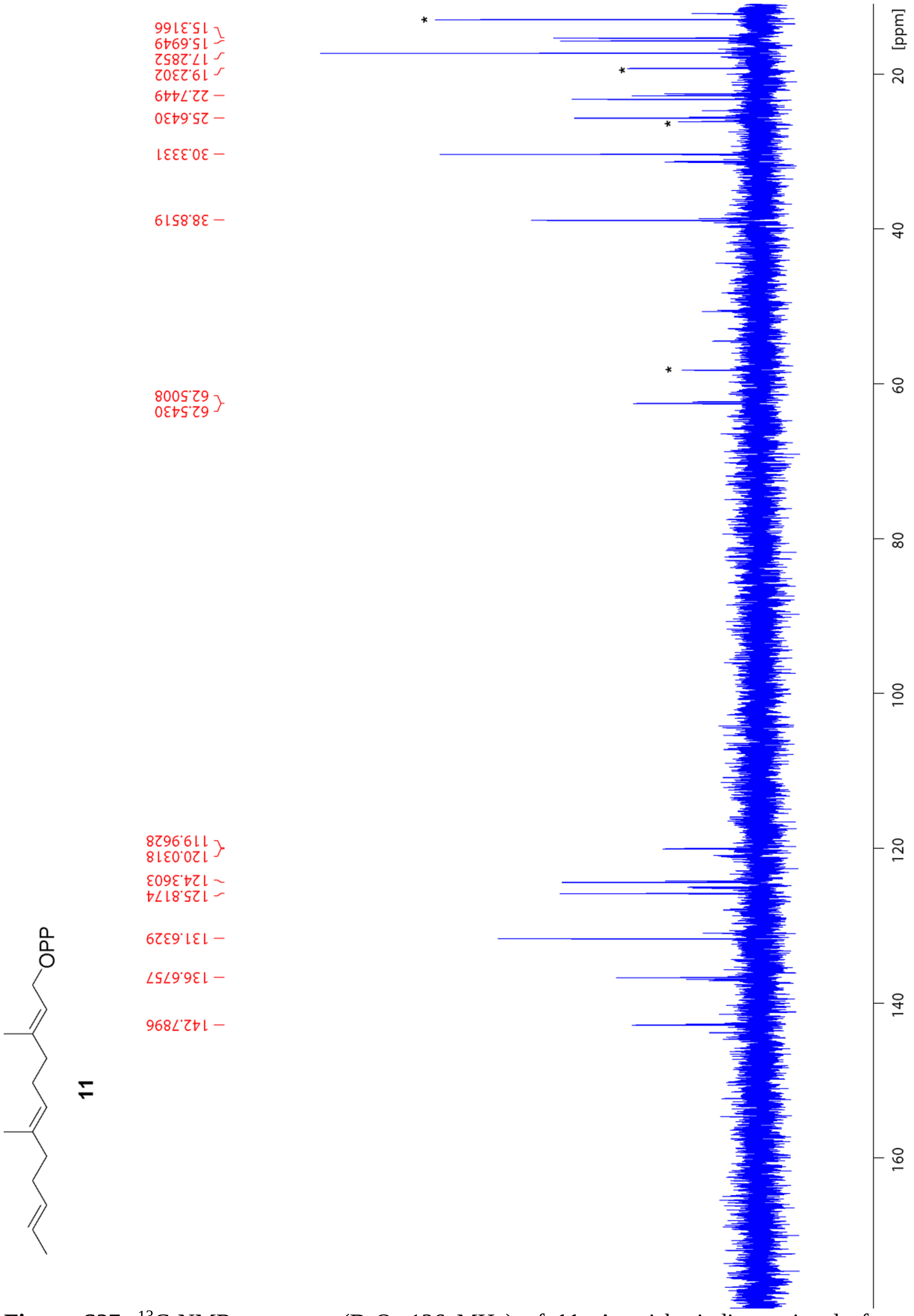

Figure S27. ^{13}C-NMR spectrum (D_2O, 126 MHz) of **11**. Asterisks indicate signals from residual tetrabutyl ammonium salt.

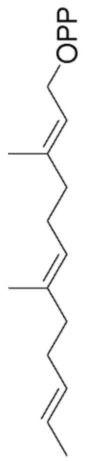

11

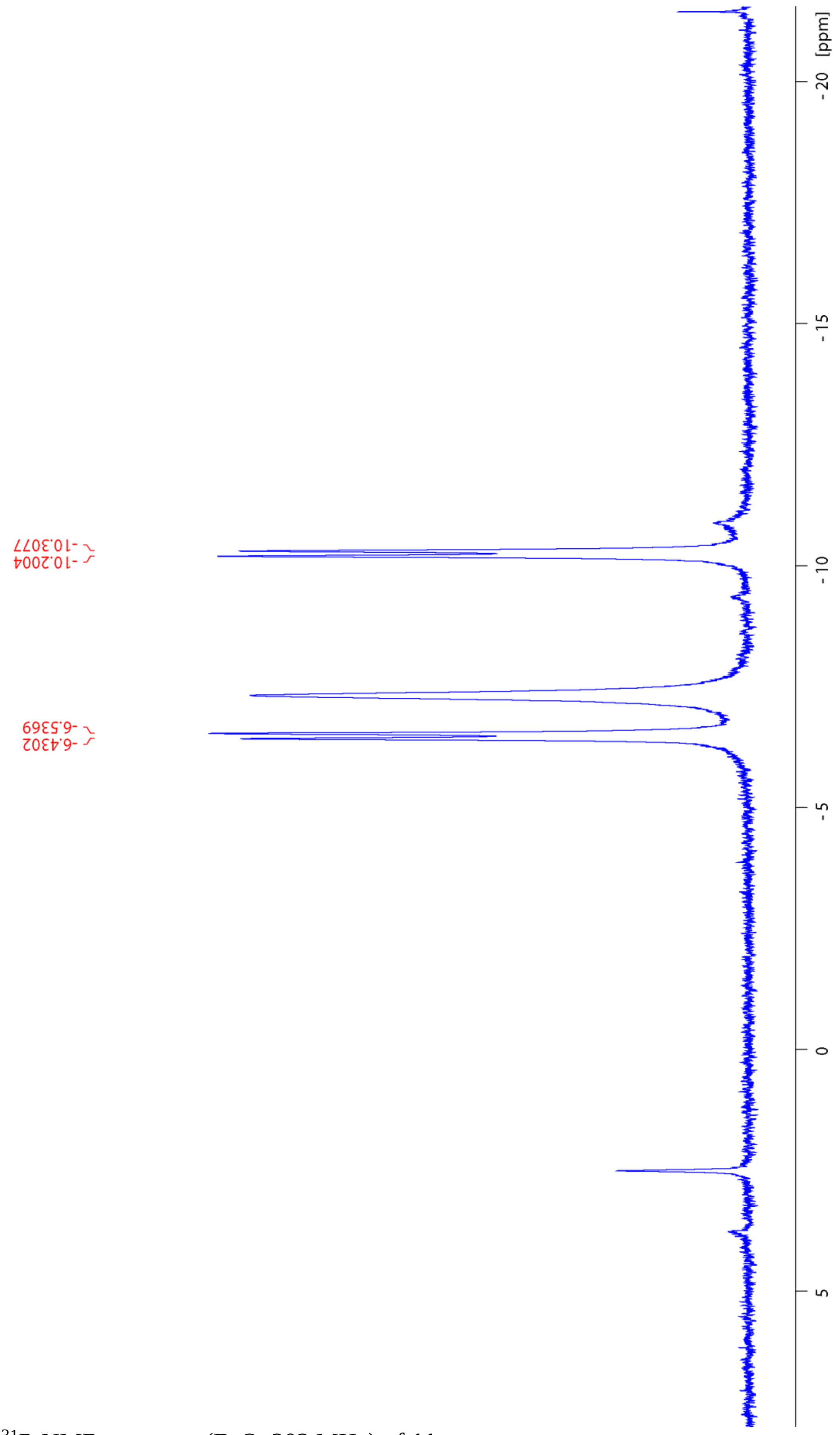

Figure S28. ^{31}P-NMR spectrum (D_2O, 202 MHz) of **11**.

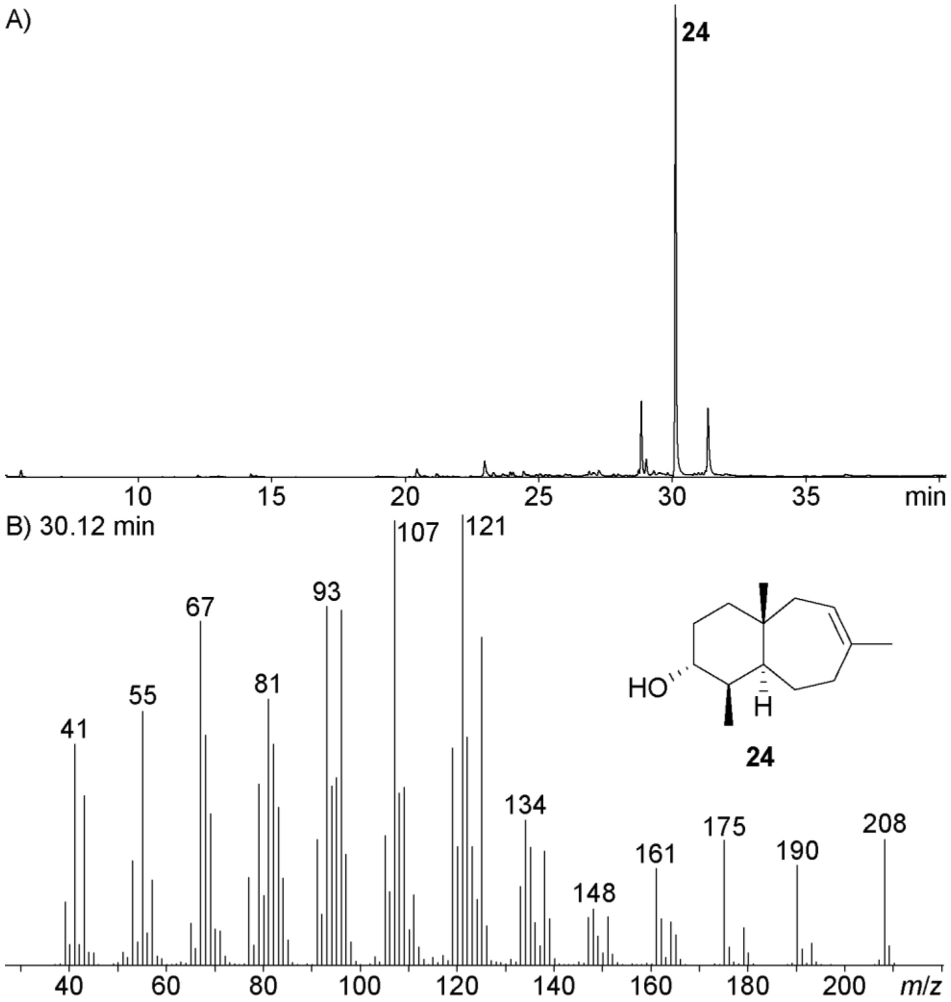

Figure S29. Incubation of DcS with 13-demethyl-FPP. A) TIC of the obtained extract. B) EI-MS spectrum of compound **24**.

Isolation of compound 24

A solution of recombinant DcS (20 mL, concentration: 141 µmol L^{-1}) obtained from 8 L of *E. coli* was incubated with 13-desmethyl FPP (**11**, 80 mg, 0.19 mmol) as described above. The reaction mixture was extracted with *n*-hexane three times and the organic layers were dried with $MgSO_4$, concentrated under reduced pressure and subjected to column chromatography (pentane/diethyl ether, 5:1) to yield compound **24** as colorless oil (1.1 mg, 0.005 mmol, 3%).

***nor*-Widdr-8-en-4-ol, (1*R*,2*R*,4a*R*,9a*S*)-1,4a,7-trimethyl-2,3,4,4a,5,8,9,9a-octahydro-1H-benzo[7]annulen-2-ol (24):** TLC (pentane/diethyl ether, 5:1): R_f = 0.36. Optical rotation: $[\alpha]_D^{20}$ = +28.4 (c 0.12, C_6D_6). GC (HP5-MS): *I* = 1627. HRMS (QToF): *m/z* = 208.1826 (calc. for $[C_{14}H_{24}O]^+$ 208.1822). MS (EI, 70 eV): *m/z* (%) = 208 (26), 190 (21), 175 (26), 161 (20), 151 (10), 138 (25), 135 (26), 134 (33), 125 (73), 123 (26), 122 (51), 121 (100), 120 (26), 119 (49), 109 (40), 108 (40), 107 (99), 105 (29), 96 (81), 95 (43), 94 (41), 93 (82),83 (37), 81 (61), 79 (42), 68 (35), 67 (80), 55 (59), 43 (40), 41 (51) (Figure S29). IR (diamond ATR): $\tilde{\nu}$ = 3364 (w), 2957 (m), 2922 (s), 2853 (m), 1670 (w), 1445 (m), 1376 (m), 1260 (m), 1080 (m), 1028 (s), 799 (s) cm^{-1}.

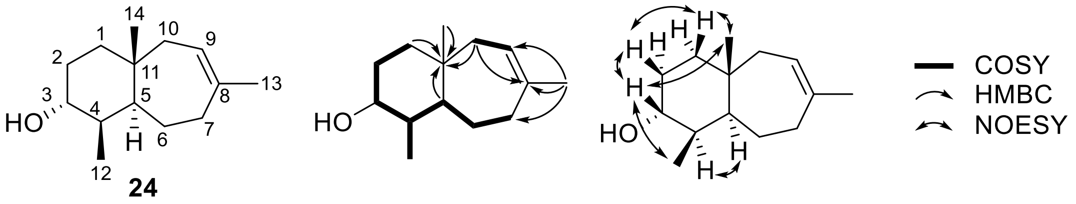

Figure S30. Structure elucidation for compound **24**. Bold lines indicate $^1H,^1H$-COSY correlations, single headed arrows HMBC correlations and double headed arrows NOESY correlations.

Table S4. NMR data of **24** recorded in C_6D_6.

C[a]		^1H[b]	^{13}C[b]
1	CH_2	1.13(ddd, $^2J_{H,H}$ = 13.3, $^3J_{H,H}$ = 5.2, 3.6, 1H)	40.9
		1.12 (dd, $^2J_{H,H}$ = 13.6, $^3J_{H,H}$ = 3.9, 1H)	
2	CH_2	1.62 – 1.59 (m, 1H)	32.1
		1.40 (dddd, $^2J_{H,H}$ = 13.4 Hz, $^3J_{H,H}$ = 12.7, 11.6, 5.0, 1H)	
3	CH	2.85 (dddd, $^3J_{H,H}$ = 10.5, 9.7, 5.8, $^4J_{H,H}$ = 3.8, 1H)	76.0
4	CH	1.08 (qdd, $^3J_{H,H}$ = 6.2, 4.5, 4.2, 1H)	40.8
5	CH	0.76 (dd, $^3J_{H,H}$ = 11.0, 2.3, 1H)	55.8
6	CH_2	1.62 – 1.57 (m, 1H)	24.0
		0.87 (dddd, $^2J_{H,H}$ = 13.7, $^3J_{H,H}$ = 12.2, 11.8, 3.4, 1H)	
7	CH_2	2.09 (dd, $^2J_{H,H}$ = 14.5, $^3J_{H,H}$ = 12.0, 1H)	34.2
		1.86 (dddd, $^2J_{H,H}$ = 14.6, $^3J_{H,H}$ = 6.8, 1.6, $^4J_{H,H}$ =1.4, 1H)	
8	C_q	–	140.7
9	CH	5.43 – 5.38 (m, 1H)	123.1
10	CH_2	1.95 (d, $^2J_{H,H}$ = 14.7, 1H)	43.7
		1.64 (dd, $^2J_{H,H}$ = 14.3, $^3J_{H,H}$ = 8.7, 1H)	
11	C_q	–	35.0
12	CH_3	0.99 (d, 6.3, 3H)	15.7
13	CH_3	1.71 (br s, 3H)	25.5
14	CH_3	0.77 (s, 3H)	17.4

[a] Carbon numbering as shown in Figure S30. [b] Chemical Shifts δ in ppm, coupling constants *J* in Hertz.

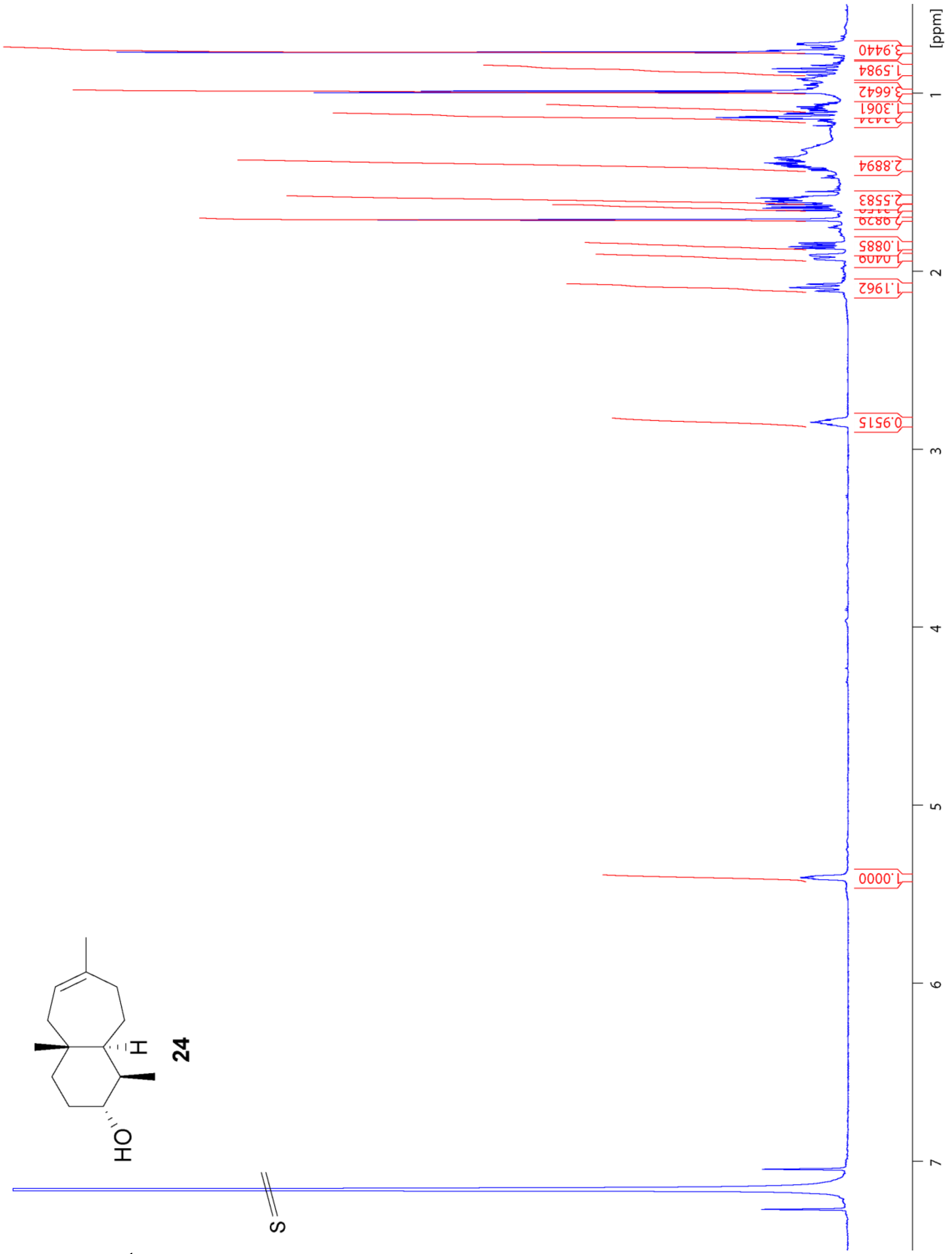

Figure S31. ^{1}H-NMR spectrum (C_6D_6, 700 MHz) of **24**. S indicates solvent peak.

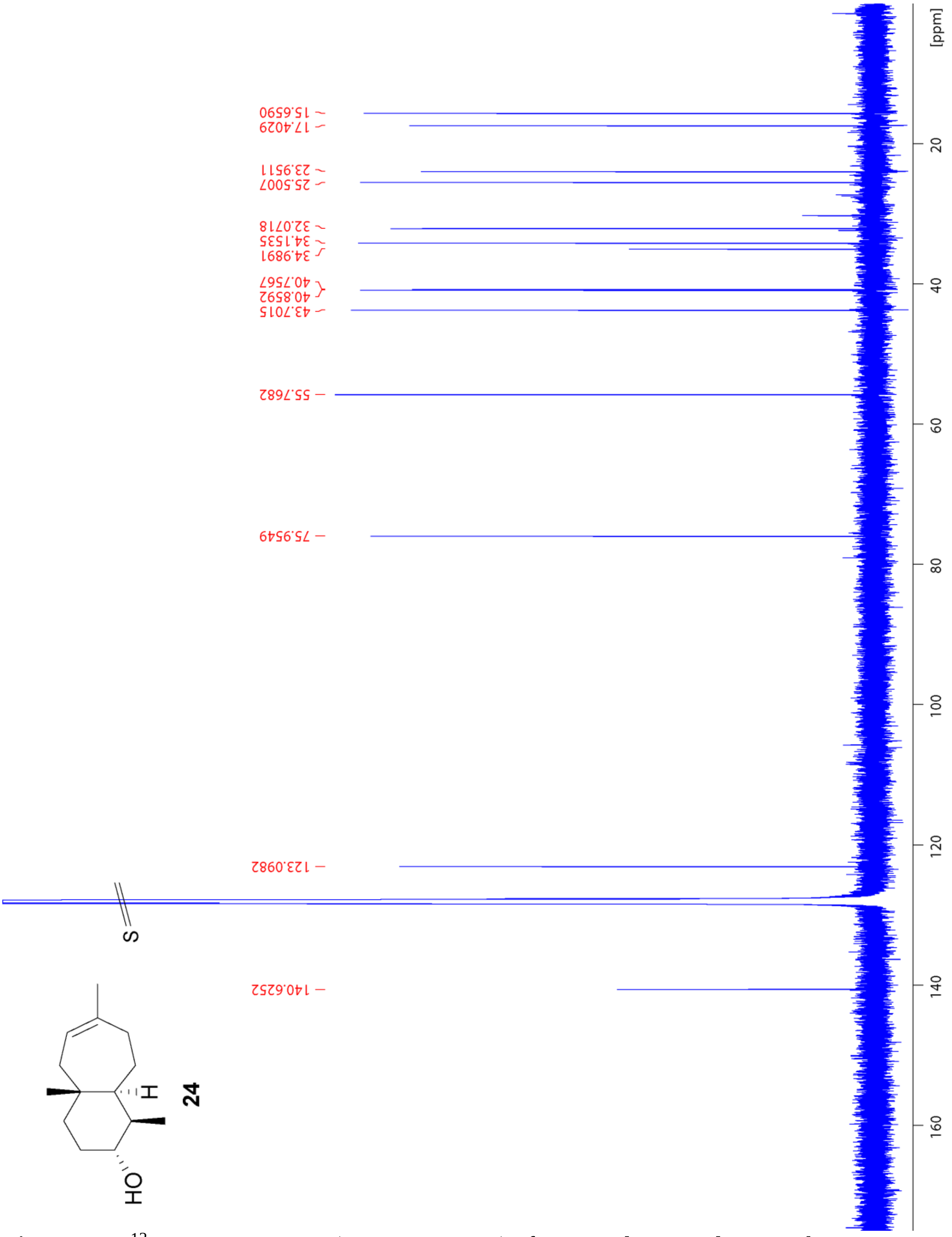

Figure S32. ^{13}C-NMR spectrum (C_6D_6, 175 MHz) of **24**. S indicates solvent peak.

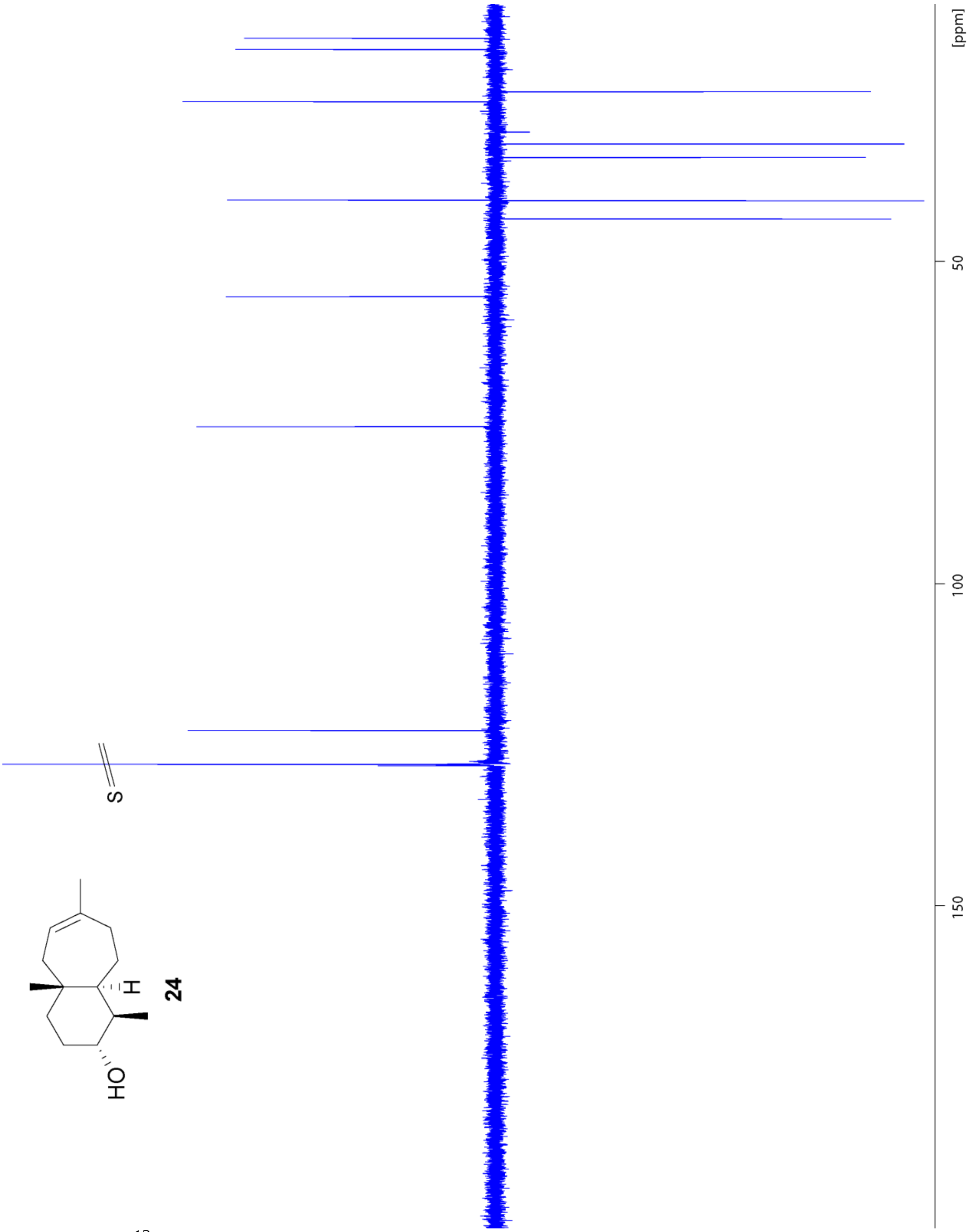

Figure S33. ^{13}C-DEPT135 spectrum (C_6D_6, 175 MHz) of **24**. S indicates solvent peak.

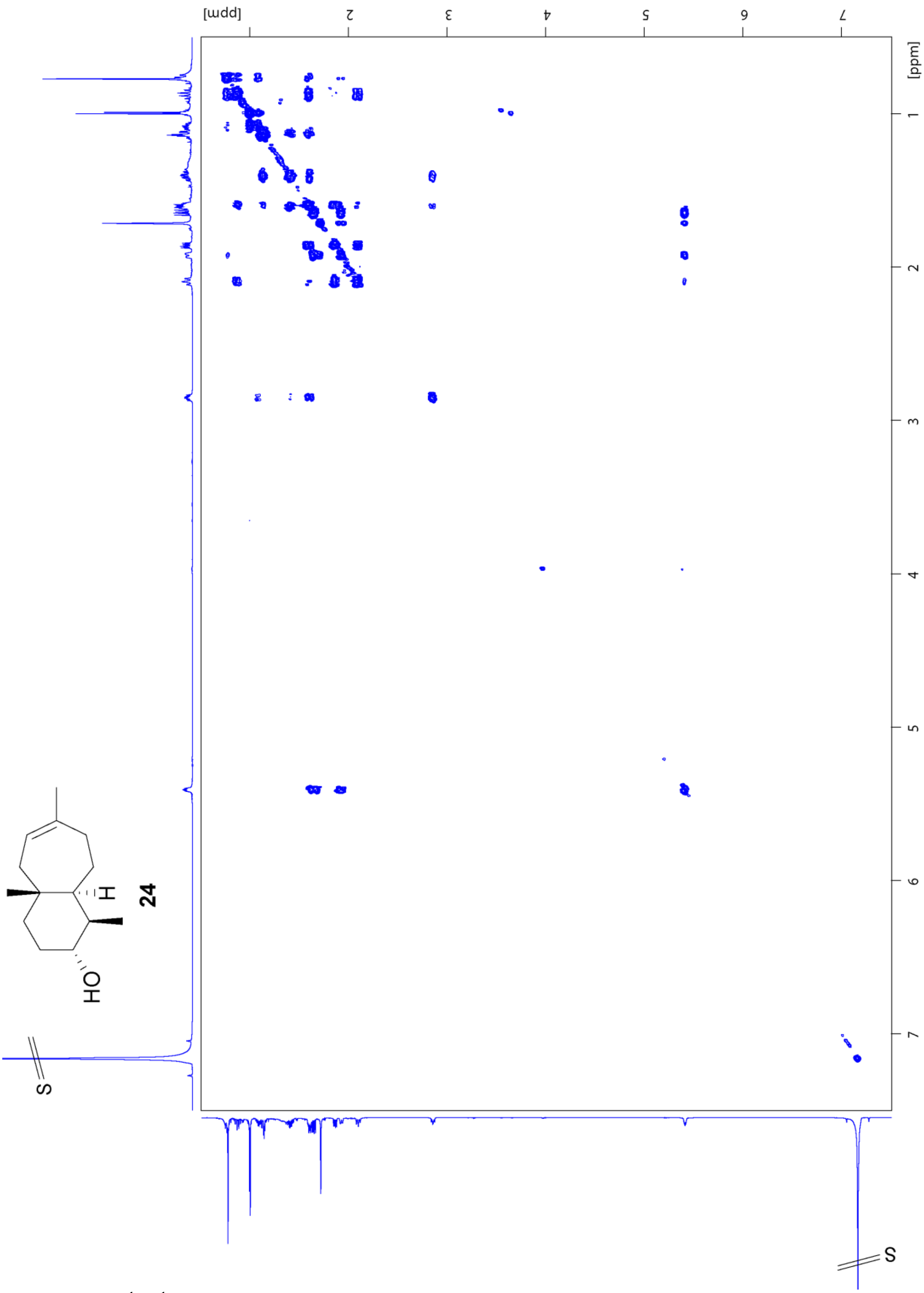

Figure S34. $^{1}H,^{1}H$-COSY spectrum (C_6D_6) of **24**. S indicates solvent peaks.

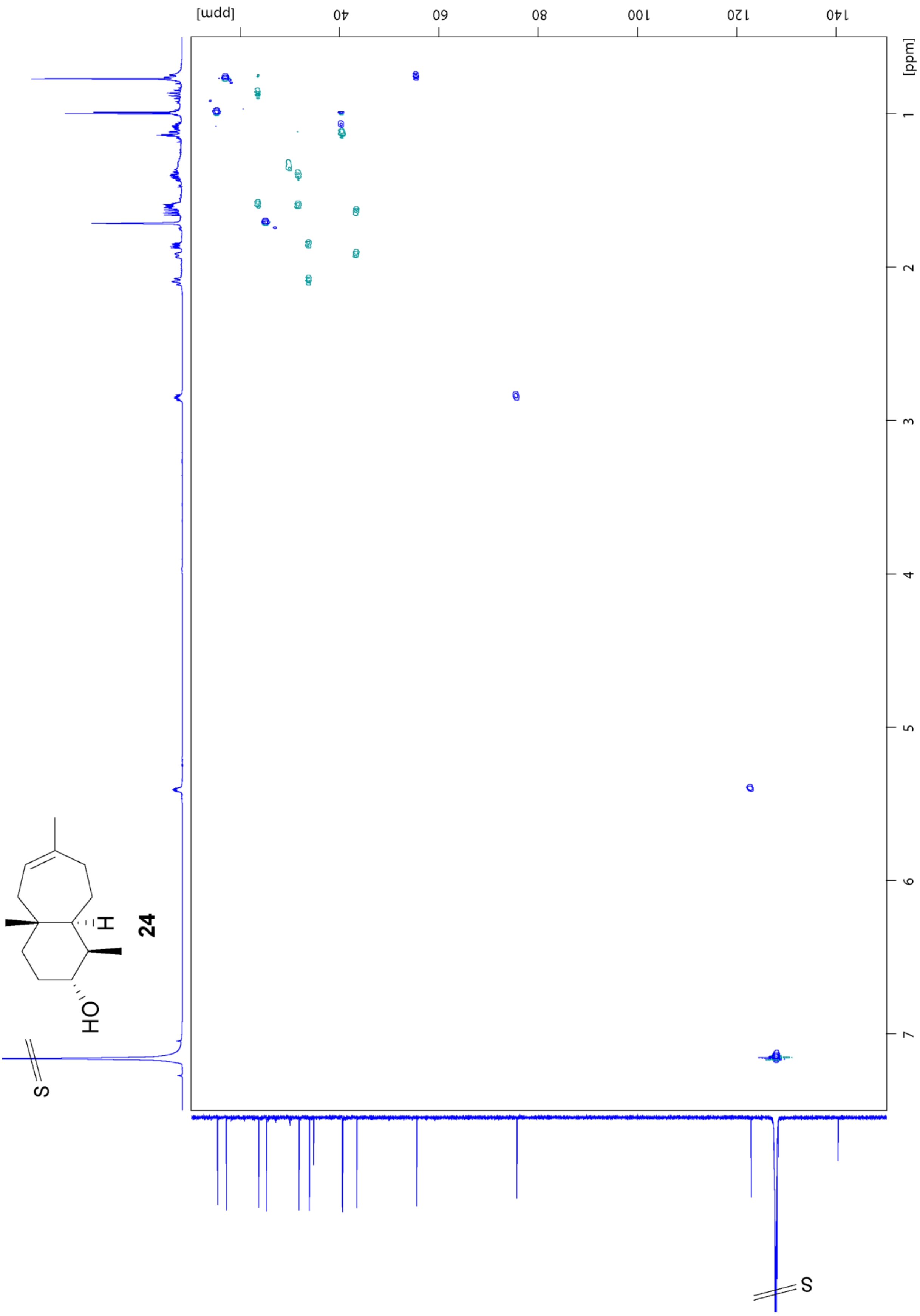

Figure S35. HSQC spectrum (C_6D_6) of **24**. S indicates solvent peaks.

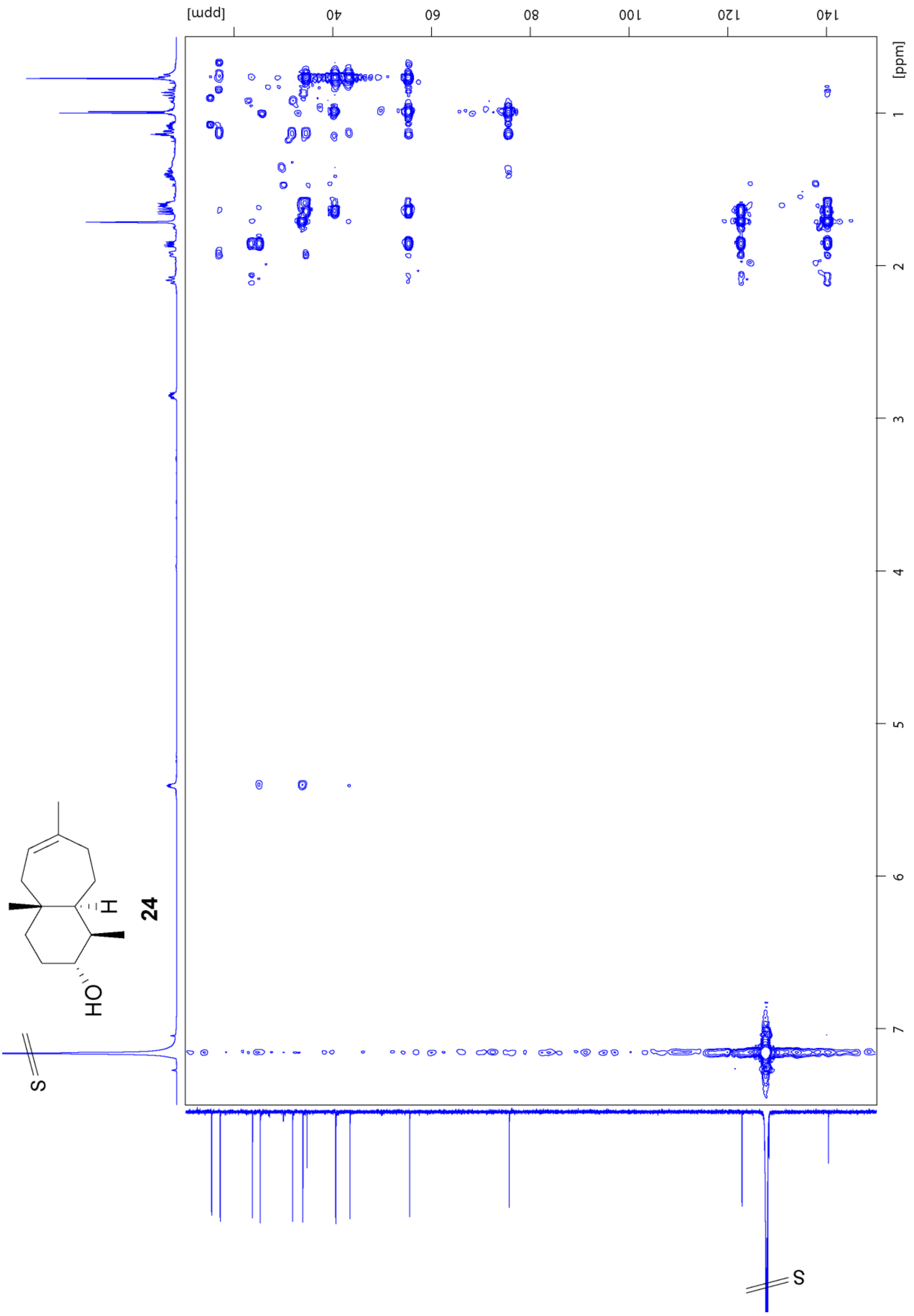

Figure S36. HMBC spectrum (C_6D_6) of **24**. S indicates solvent peaks.

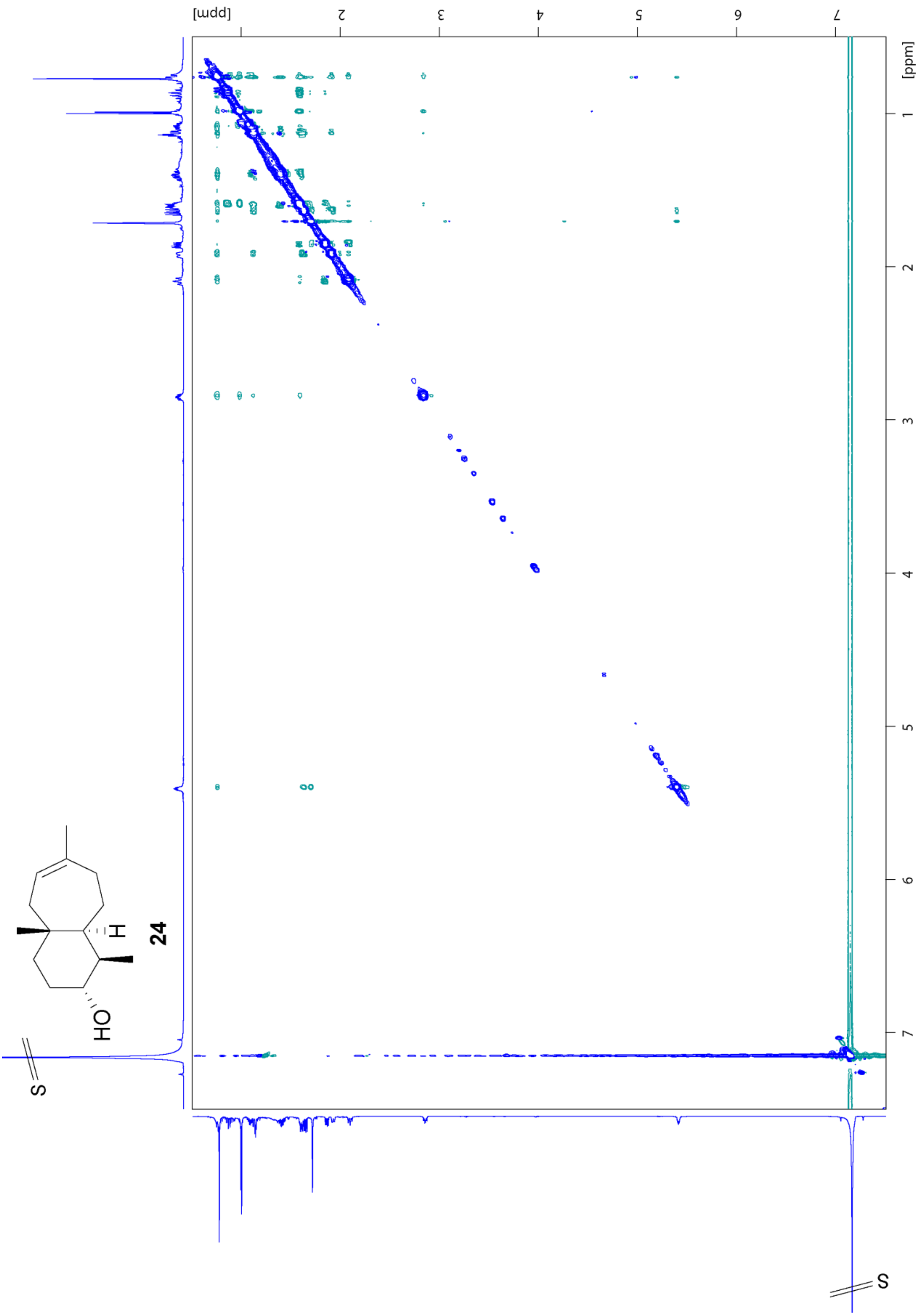

Figure S37. NOESY spectrum (C_6D_6) of **24**. S indicates solvent peaks.

Synthetic route to (*E*)-2-methyl-2-butenyl diphosphate (25)

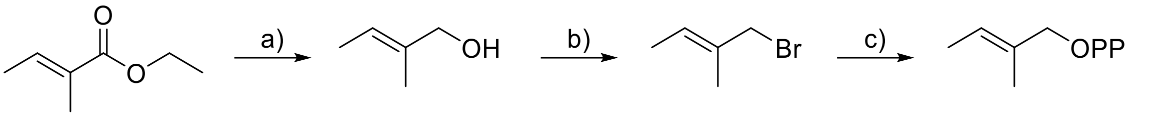

S20: 90 % **25:** 92 % (2 steps)

Scheme S3. Synthesis of **25**. Reaction conditions: a) D*i*BAl-H, Et_2O, overnight; b) PBr_3, THF, 2 h, c) $(\text{N}n\text{Bu}_4)_3HP_2O_7$, MeCN, overnight.

Synthesis of (*E*)-2-methyl-2-buten-1-ol (S20)

To a cooled (–30 °C) solution of ethyl tiglate (3.60 g, 26.1 mmol, 1.0 eq) in diethyl ether (250 mL) was slowly added D*i*BAl-H (1 M in hexane, 57.5 mL, 57.5 mmol, 2.2 eq) and the mixture was stirred overnight warming to room temperature. The reaction was cooled to 0 °C and saturated aqueous Na-K-tartrate solution was added. The aqueous phase was extracted three times with diethyl ether and the combined organic phases were dried with $MgSO_4$, carefully concentrated under reduced pressure (350 mbar) and purified by column chromatography (pentane/diethyl ether, 2:1) to yield the product as colorless liquid (2.02 g, 23.5 mmol, 90%). TLC (pentane/diethyl ether, 2:1): R_f = 0.38, ^{1}H-NMR ($CDCl_3$, 500 MHz): δ = 5.47 (br s, 1H), 3.93 (br s, 2H), 1.65 (br s, 3H), 1.63 – 1.58 (m, 3H) ppm. ^{13}C-NMR ($CDCl_3$, 125 MHz): δ = 135.6 (C_q), 120.8 (CH), 69.2 (CH_2), 13.5 (CH_3), 13.2 (CH_3) ppm.

Synthesis of (*E*)-2-methyl-2-butenyl diphosphate (tiglyl diphosphate, 25)

A solution of **S20** (2.02 g, 23.5 mmol, 1.0 eq) in diethyl ether (50 mL) was cooled to 0 °C and phosphorous tribromide (0.89 mL, 9.4 mmol, 0.4 eq) was slowly added. After 2 h ice cold saturated K_2CO_3 solution was added and quickly extracted with diethyl ether three times. The organic phase was dried with $MgSO_4$, concentrated carefully under reduced pressure (600 mbar) and directly added to a solution of tris(tetra-*n*butyl)ammonium pyrophosphate (5.31 g, 5.88 mmol, 0.25 eq) in acetonitrile (40 mL). the mixture was stirred overnight and was concentrated under reduced pressure. The residue was dissolved in aqueous ammonium bicarbonate solution (25 mM) and loaded onto a DOWEX® 50WX8 cation exchange column (NH_4^+ form, pH ~ 7.0). The desired compound was eluted with 1.5 CV of NH_4HCO_3 buffer (25 mM, 5% *i*PrOH), the eluate was frozen with liquid N_2 and lyophilized to yield the diphosphate as a white powder (1.64 g, 5.51 mmol, 92%). ^{1}H-NMR (D_2O, 500 MHz): δ = 5.64 (qq, $^3J_{H,H}$ = 6.9 Hz, $^4J_{H,H}$ = 1.4 Hz, 1H), 4.33 (br s, 2H), 1.69 (br s, 3H), 1.65 (d, $^3J_{H,H}$ = 6.8 Hz, 3H) ppm. ^{13}C-NMR (D_2O, 125 MHz): δ = 132.3 (d, $^3J_{C,P}$ = 7.8 Hz, C_q), 123.8 (CH), 72.1 (d, $^2J_{C,P}$ = 5.6 Hz, CH_2), 12.9 (CH_3), 12.6 (CH_3) ppm. ^{31}P-NMR (D_2O, 200 MHz): δ = –6.9 (d, $^2J_{P,P}$ = 22.4 Hz), –10.7 (d, $^2J_{P,P}$ = 22.3 Hz) ppm.

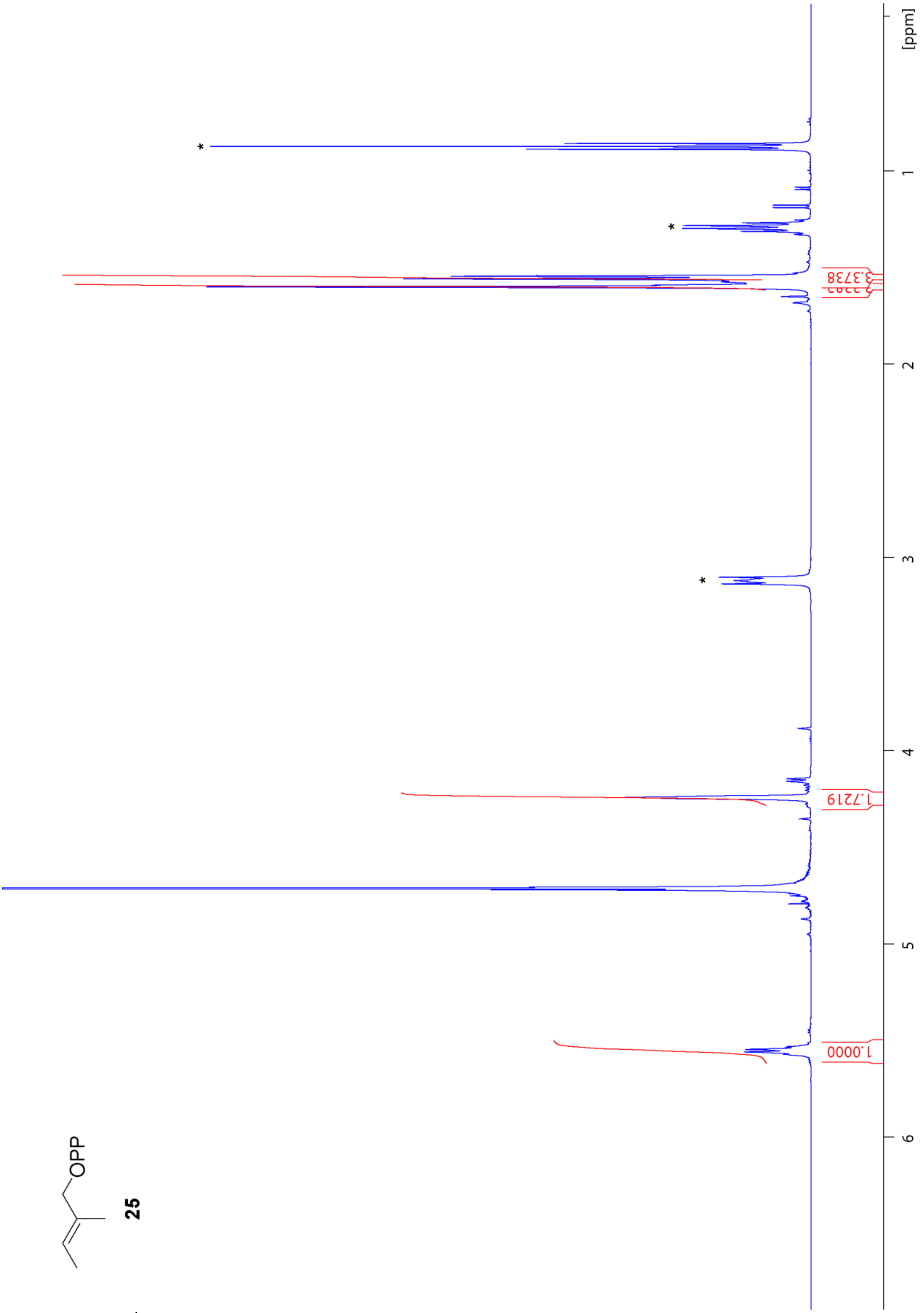

Figure S38. ^{1}H-NMR spectrum (D_2O, 500 MHz) of **25**. S indicates solvent peak. Asterisks indicate signals from residual tetrabutyl ammonium salt.

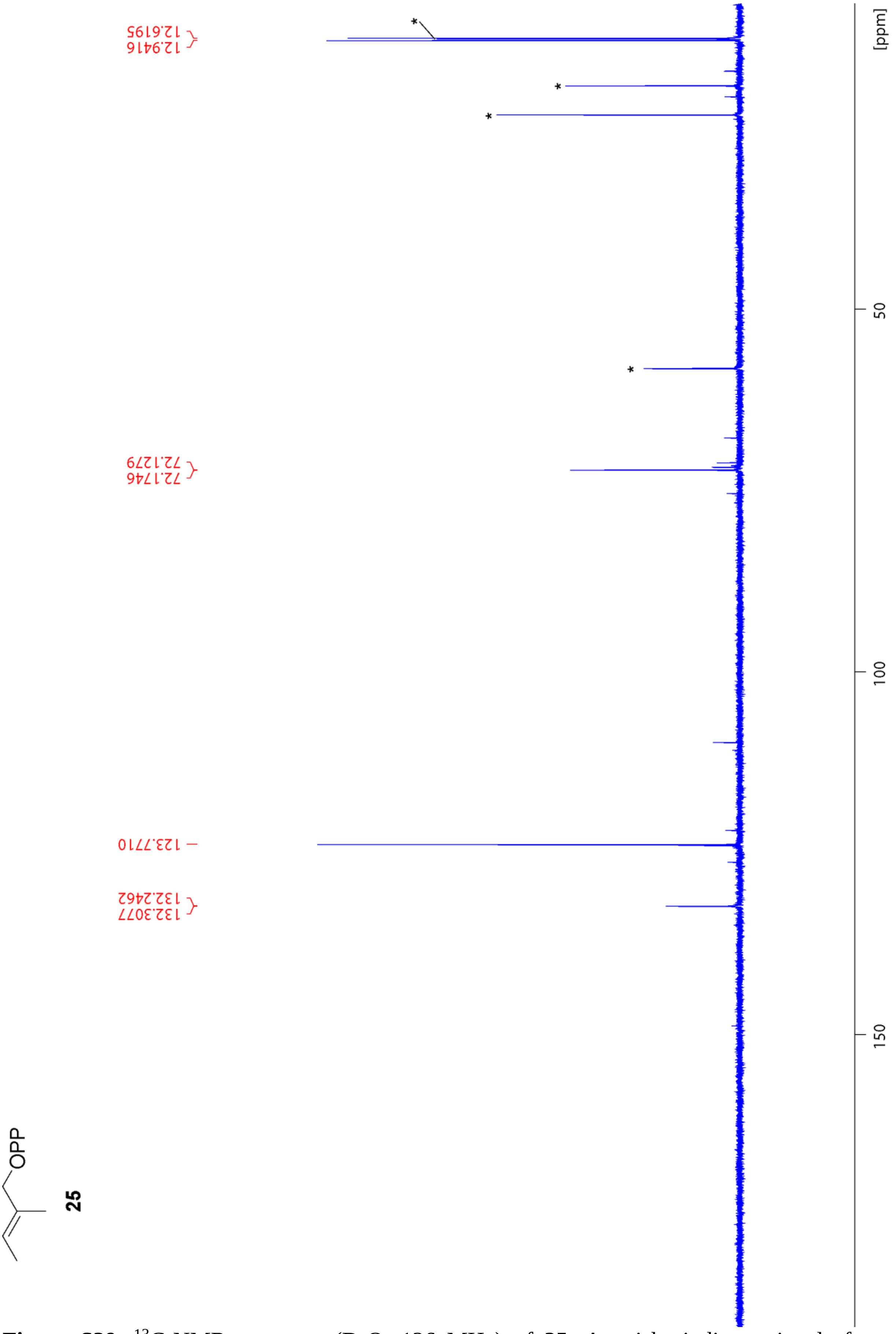

Figure S39. ^{13}C-NMR spectrum (D_2O, 126 MHz) of **25**. Asterisks indicate signals from residual tetrabutyl ammonium salt.

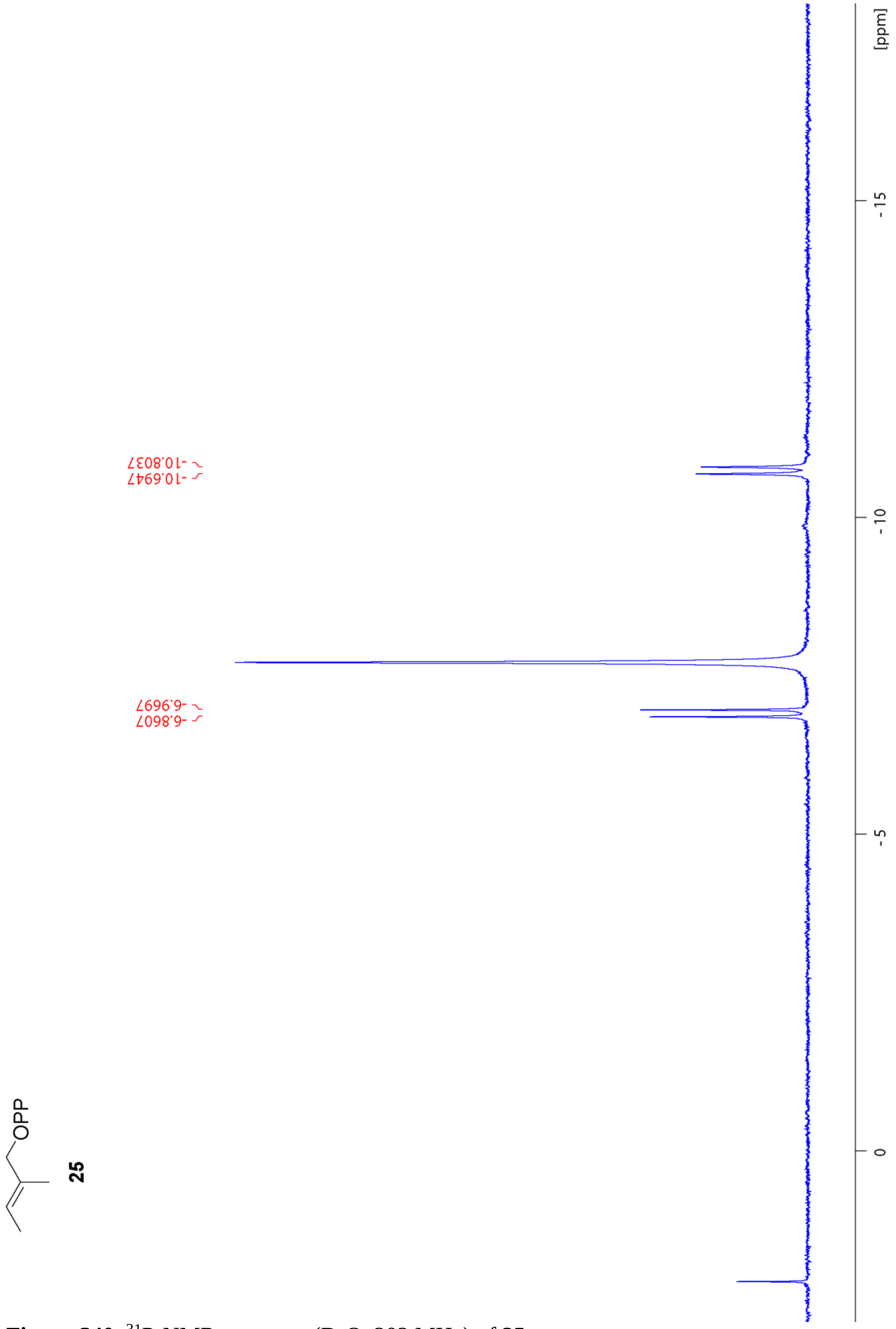

Figure S40. ^{31}P-NMR spectrum (D_2O, 202 MHz) of **25**.

Incubation experiments with calf intestinal phosphatase

Incubation experiments were performed on a 1 mL scale as described for shorter substrates above, after 3 h, calf intestinal phosphatase (CIP, New England Biolabs, 10 units) and CutSmart Buffer (10 µL) were added and the mixture was incubated for 1 h at 37 °C. The mixture was extracted with hexane (200 µL), the extracts were dried and subjected to GC/MS (Figure S41).

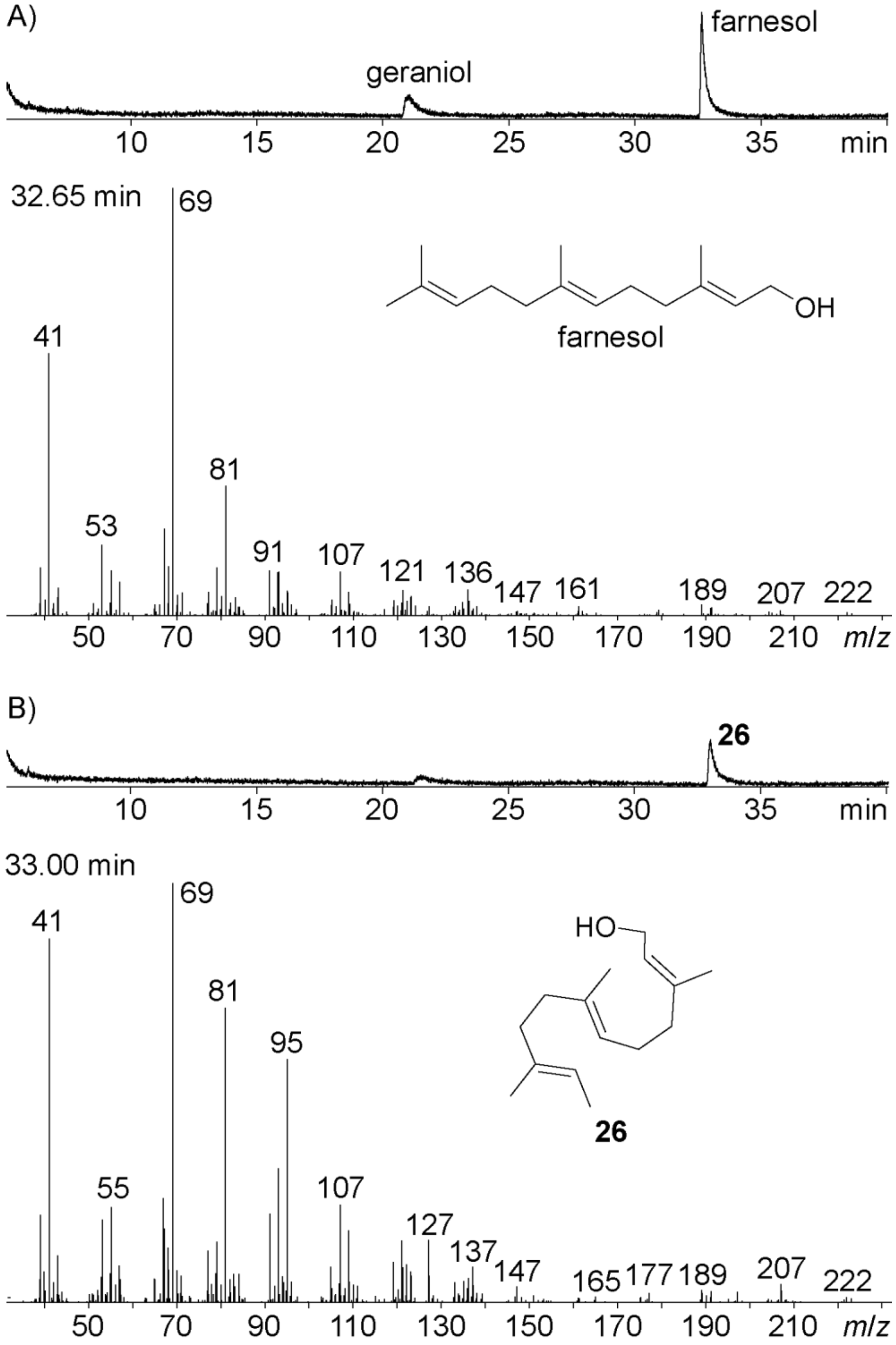

Figure S41. Incubation of A) DMAPP and B) tiglyl diphosphate with IPP, FPPS and CIP. Mass spectra correspond to the resulting C_{15} alcohol.

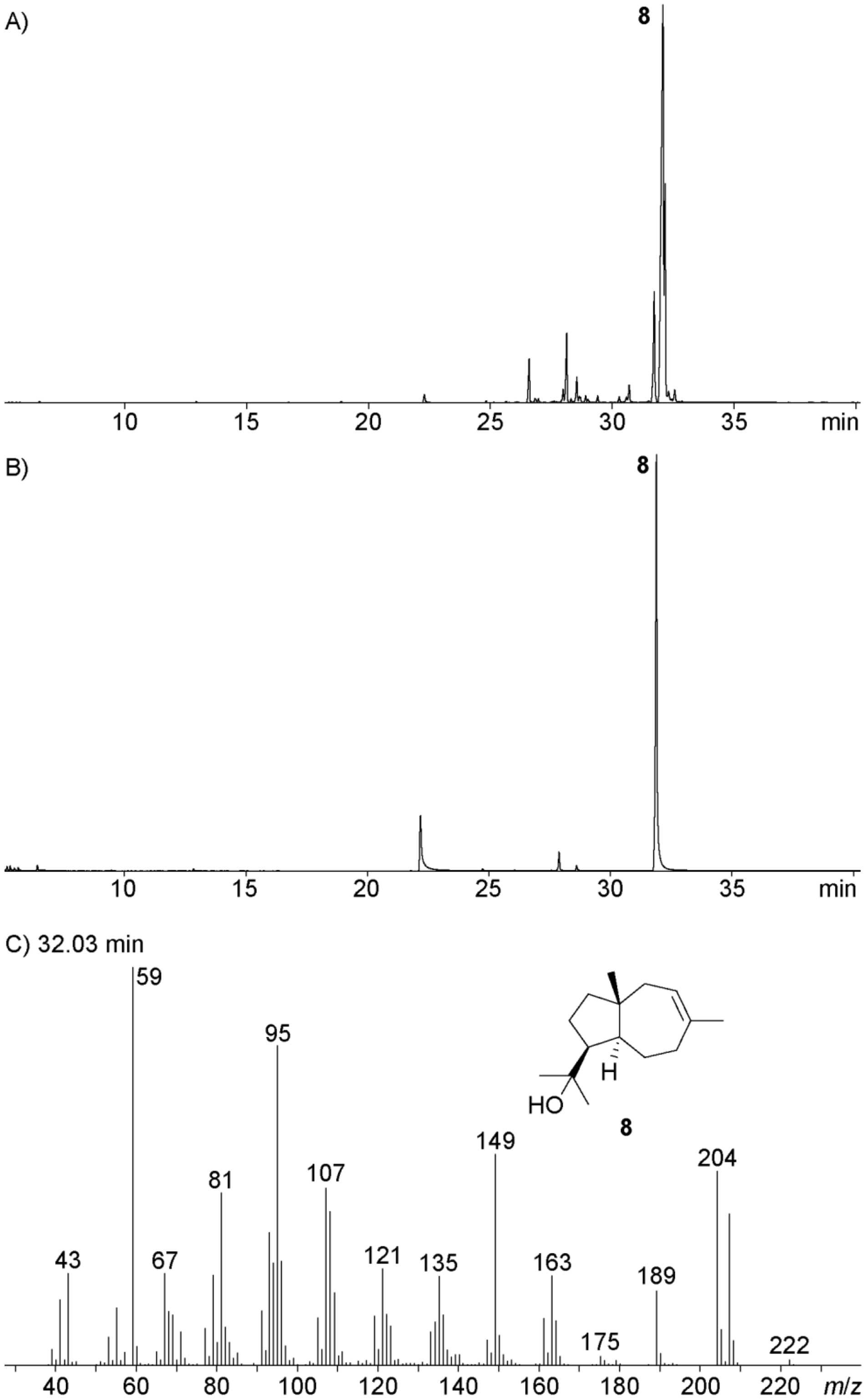

Figure S42. Total ion chromatogram of the extract from incubation of DcS and FPPS purified by Ni^{2+}-NTA chromatography with A) tiglyl diphosphate and IPP, B) only IPP. C) EI-MS spectrum of the main compound dauc-8-en-11-ol (**8**).

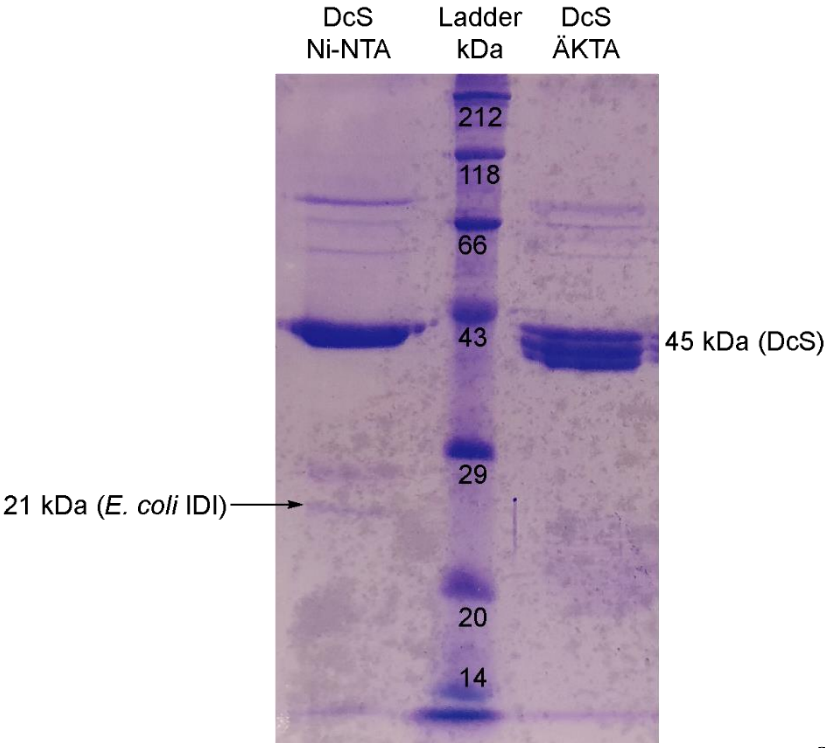

Figure S43. SDS-PAGE of recombinant DcS purified by Ni^{2+}-NTA chromatography (left, DCS Ni-NTA) and after further purification by FPLC (DcS ÄKTA). The slight band between 20 and 29 kDa in the protein sample only purified by Ni^{2+}-NTA chromatography is *E. coli* isopentenyl diphosphate isomerase.

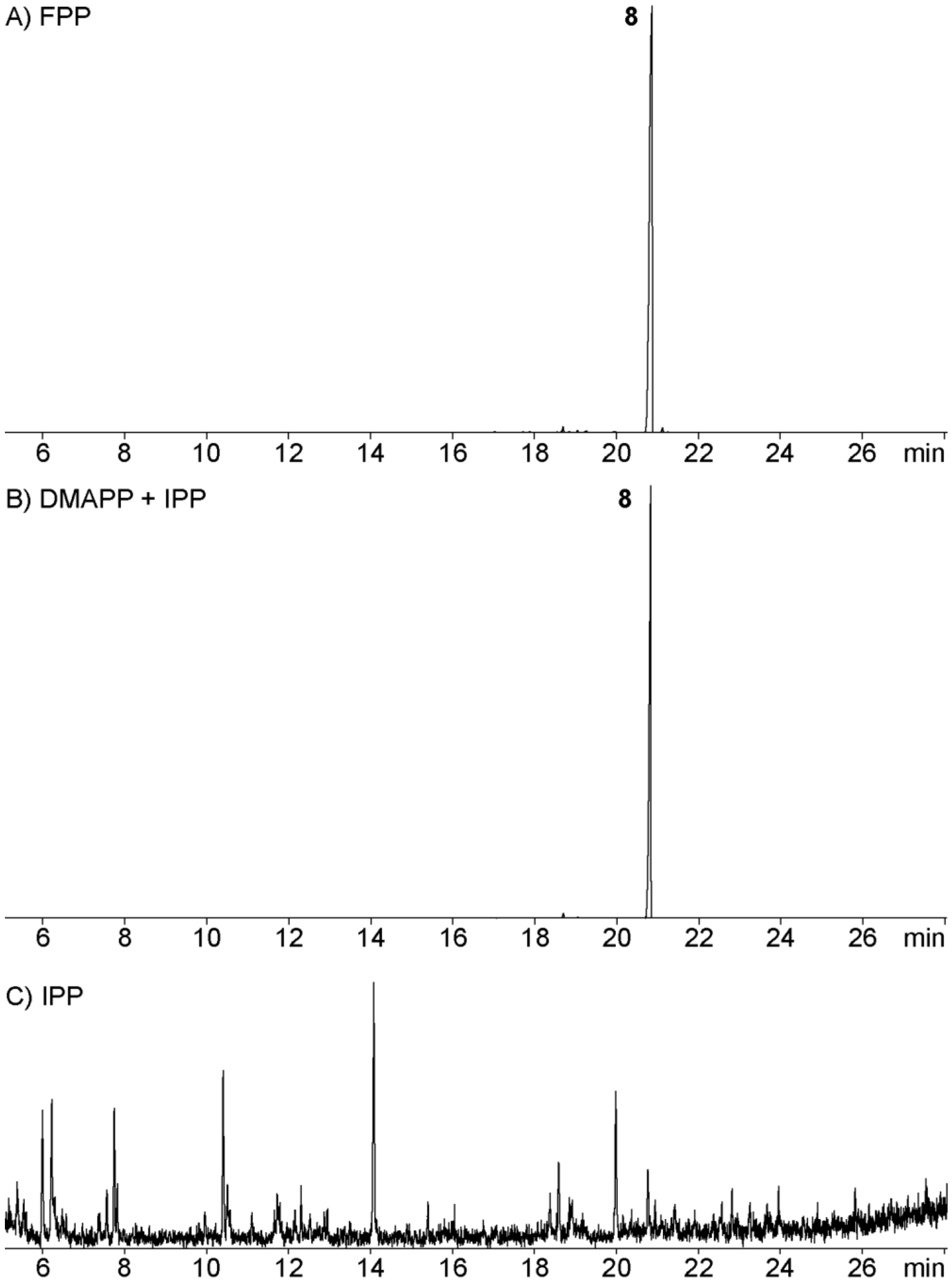

Figure S44. Total ion chromatograms of incubation experiments using FPLC-purified DcS. Incubation of DcS with A) FPP, B) DMAPP, IPP and FPPS and C) IPP and FPPS. Vanishing of the peak for **8** in experiment C) shows absence of IDI activity.

Synthesis of trisammonium (2*E*,6*E*)-3,7,11-trimethyldodeca-2,6,11-trien-1-yl diphosphate (15)

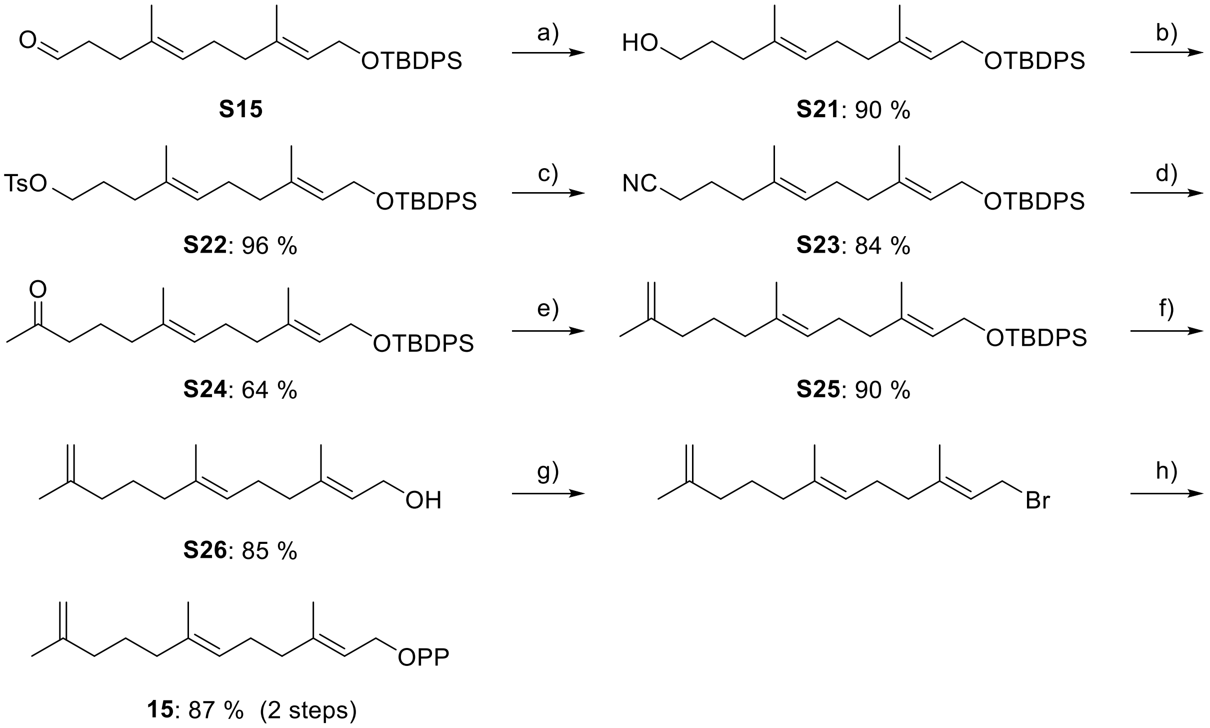

Scheme S4. Synthesis of trisammonium (2*E*,6*E*)-3,7,11-trimethyldodeca-2,6,11-trien-1-yl diphosphate (**15**). Reaction conditions: a) $NaBH_4$, EtOH, 0 °C, 1 h; b) TsCl, DMAP, Et_3N, CH_2Cl_2, 2 h; c) KCN, DMSO, 6 h; d) MeLi, Et_2O, –78 °C to room temperature, overnight; e) CH_3PPh_3I, n-BuLi, THF, –78 °C to room temperature, overnight; f) TBAF, THF, 1.5 h; g) PBr_3, Et_2O, 0 °C, 1 h; h) $(NBu_4)_3HP_2O_7$, MeCN, overnight.

Synthesis of (*4E,8E*)-10-((*tert*-butyldiphenylsiyl)oxy)-4,8dimethyldeca-4,8-dien-1-ol (S21)

S15 (8.93 g, 2.54 mmol) was dissolved in EtOH (240 mL, 0 °C), followed by the addition of $NaBH_4$ (1.55 g, 41.08 mmol, 2.0 eq) in 5 batches (every batch around 5 min). The reaction mixture was stirred at 0 °C for 1h and quenched by pouring into ice-water (400 mL). The product was extracted with Et_2O (3 x 150 mL), the combined organic layers were washed with sat. NaCl solution and dried with $MgSO_4$. The solvent was evaporated, and the residue was purified via silica gel chromatography (cyclohexane/ethyl acetate, 5:1) to afford compound **S21** (8.1 g, 18.55 mmol, 90%) as a colorless oil. TLC (cyclohexane/ethyl acetate, 5:1): R_f = 0.26. EI-MS (70 eV): *m*/*z* (%) = 379 (1), 269 (1), 251 (1), 229 (4), 225 (2), 211 (2), 199 (100), 188 (3), 181 (8), 163 (21), 149 (3), 135 (15), 121 (14), 107 (24), 95 (41), 81 (27), 67 (12), 55 (7), 41 (5). GC (HP5-MS): *I* = 3046. ^{1}H-NMR ($CDCl_3$, 500 MHz): δ = 7.71 – 7.67 (m, 4H), 7.44 – 7.35 (m, 6H), 5.38 (tq, $^3J_{H,H}$ = 6.3 Hz, $^4J_{H,H}$ = 1.3 Hz, 1H), 5.15 (tq, $^3J_{H,H}$ = 7.0 Hz, $^4J_{H,H}$ = 1.3 Hz, 1H), 4.22 (d, $^3J_{H,H}$ = 6.2 Hz, 2H), 3.61 (t, $^3J_{H,H}$ = 6.5 Hz, 2H), 2.11 – 2.03 (m, 4H), 2.01 – 1.97 (m, 2H), 1.70 – 1.63 (m, 2H), 1.62 (s, 3H), 1.44 (d, $^4J_{H,H}$ = 1.2 Hz, 3H), 1.05 (s, 9H) ppm. ^{13}C-NMR ($CDCl_3$, 126 MHz,): δ = 137.02 (C_q), 135.76 (4 x CH), 134.94 (C_q), 134.22 (2 x C_q), 129.64 (2 x CH), 127.72 (4 x CH), 124.69 (CH), 124.32 (CH), 62.89 (CH_2), 61.32 (CH_2), 39.55 (CH_2), 36.10 (CH_2), 30.82 (CH_2), 27.00 (3 x CH_3), 26.32 (CH_2), 19.33 (C_q), 16.43 (CH_3), 16.03 (CH_3) ppm.

Synthesis of (*4E,8E*)-10-((*tert*-butyldiphenylsiyl)oxy)-4,8dimethyldeca-4,8-dien-1-yl 4-methylbenzenesulfonate (S22)

S21 (8.10 g, 18.55 mmol), Et_3N (4.49 g, 44.40 mmol, 2.4 eq) and DMAP (1.13 g, 9.28 mmol, 0.5 eq) were dissolved in CH_2Cl_2 (60 mL). The solution mixture was cooled to 0 °C, followed by adding TsCl (5.29 g, 27.75 mmol, 1.5 eq, suspended in 20 mL CH_2Cl_2) dropwise. The mixture was stirred at room temperature for 2 h and diluted with Et_2O (150 mL), followed by washing with water (200 mL). The aqueous layer was extracted twice with Et_2O (100 mL). The organic layers were combined and washed with 0.5 M HCl, sat. $NaHCO_3$ solution, brine, and dried with $MgSO_4$. The solvent was removed under reduced pressure and the product **S22** (10.51 g, 17.79 mmol, 96%) was obtained by silica gel chromatography (cyclohexane/ethyl acetate, 10:1) as a colorless oil. TLC (cyclohexane/ethyl acetate, 10:1): R_f = 0.32. ^{1}H-NMR ($CDCl_3$, 500 MHz): δ = 7.81 – 7.76 (m, 2H), 7.71 – 7.66 (m, 4H), 7.43 – 7.35 (m, 6H), 7.36 – 7.30 (m, 2H), 5.35 (tq, $^3J_{H,H}$ = 6.3 Hz, $^4J_{H,H}$ = 1.3 Hz, 1H), 5.02 (dd, $^3J_{H,H}$ = 7.0 Hz, $^4J_{H,H}$ = 1.3 Hz, 1H), 4.21 (d, $^3J_{H,H}$ = 6.2 Hz, 2H), 3.99 (t, $^3J_{H,H}$ = 6.4 Hz, 2H), 2.44 (s, 3H), 2.05 – 1.94 (m, 4H), 1.96 – 1.90 (m, 2H), 1.76 – 1.68 (m, 2H), 1.53 (d, $^4J_{H,H}$ = 1.3 Hz, 3H), 1.43 (s, 3H), 1.04 (s, 9H) ppm. ^{13}C-NMR ($CDCl_3$, 126 MHz): δ = 144.75 (C_q), 136.99 (C_q), 135.75 (4 x CH), 134.94 (C_q), 134.22 (2 x Cq), 133.41 (C_q), 129.94 (2 x CH), 129.65 (2 x CH), 128.05 (2 x CH), 127.72 (4 x CH), 125.47 (CH), 124.27 (CH), 70.28 (CH_2), 61.26 (CH_2), 39.47 (CH_2), 35.25 (CH_2), 27.17 (CH_2), 26.99 (3 x CH_3), 26.41 (CH_2), 21.78 (CH_3), 19.32 (C_q), 16.47 (CH_3), 15.93 (CH_3) ppm.

Synthesis of (5*E*,9*E*)-11-((*tert*-butyldiphenylsilyl)oxy)-5,9-dimethylundeca-5,9-dienenitrile (S23)

To a solution of **S22** (10.5 g, 17.8 mmol) in DMSO (80 mL), KCN (1.34 g, 20.6 mmol, 1.2 eq) was added at room temperature. The mixture was stirred for 6 h and then diluted with water (500 mL). The product was extracted with Et_2O (3 x 120 mL), the combined organic layers were washed with sat. $NaHCO_3$ solution and brine and were dried with $MgSO_4$. The solvent was removed under reduced pressure and the product **S23** (6.63 g, 14.9 mmol, 84%) was purified by silica gel chromatography (cyclohexane/ethyl acetate, 10:1). TLC (cyclohexane/ethyl acetate, 10:1): R_f = 0.31. EI-MS (70 eV): *m/z* (%) = 388 (38), 251 (1), 239 (1), 199 (100), 188 (2), 181 (6), 173 (1), 151 (1), 135 (9), 121 (4), 105 (3), 81 (6), 77 (6), 67 (2), 55 (2), 41 (2). GC (HP5-MS): I = 3163. ^{1}H-NMR ($CDCl_3$, 500 MHz): δ = 7.72 – 7.68 (m, 4H), 7.43 – 7.36 (m, 6H), 5.38 (tq, $^3J_{H,H}$ = 6.3 Hz, $^4J_{H,H}$ = 1.3 Hz, 1H), 5.17 (tq, $^3J_{H,H}$ = 7.0 Hz, $^4J_{H,H}$ =1.3 Hz, 1H), 4.22 (d, $^3J_{H,H}$ = 6.3 Hz, 2H), 2.26 (t, $^3J_{H,H}$ = 7.2 Hz, 2H), 2.14 – 2.07 (m, 4H), 2.00 (dd, $^3J_{H,H}$ = 8.6 Hz, $^3J_{H,H}$ = 6.6 Hz, 2H), 1.75 (p, $^3J_{H,H}$ = 7.2 Hz, 2H), 1.60 (d, $^4J_{H,H}$ = 1.3 Hz, 3H), 1.45 (d, $^4J_{H,H}$ = 1.3 Hz, 3H), 1.05 (s, 9H) ppm. ^{13}C-NMR ($CDCl_3$, 126 MHz): δ = 136.87 (C_q), 135.75 (4 x CH), 134.21 (2 x C_q), 132.67 (C_q), 129.65 (2 x CH), 127.72 (4 x CH), 126.44 (CH), 124.40 (CH), 119.93 (C_q), 61.26 (CH_2), 39.42 (CH_2), 38.34 (CH_2), 26.99 (3 x CH_3), 26.35 (CH_2), 23.54 (CH_2), 19.33 (C_q), 16.44 (CH_3), 16.40 (CH_2), 15.79 (CH_3) ppm.

Synthesis of (6*E*,10*E*)-12-((*tert*-butyldiphenylsilyl)oxy)-6,10-dimethyldodeca-6,10-dien-2-one (S24)

S23 (6.63 g, 14.9 mmol) was dissolved in Et_2O (80 mL) and cooled to –78 °C, followed by dropwise addition of MeLi (1.6 M in Et_2O, 28 mL, 44.6 mmol, 3.0 eq). The reaction mixture stirred overnight and was allowed to warm to room temperature. The reaction was quenched by pouring the mixture into an ice-cold NH_4Cl solution (150 mL sat. NH_4Cl solution with 200 mL ice-water), and aqueous phase was extracted with Et_2O (3 x 120 mL). The combined organic layers were dried with $MgSO_4$ and evaporated under reduced pressure. The product **S24** (4.40 g, 9.51 mmol, 64%) was obtained via silica gel chromatography (cyclohexane/ethyl acetate, 10:1) as a colorless oil. TLC (cyclohexane/ethyl acetate, 10:1): R_f = 0.28. EI-MS (70

eV): *m*/*z* (%) = 405 (4), 311 (1), 281 (1), 269 (10), 257 (1), 239 (2), 225 (1), 213 (1), 199 (100), 189 (5), 181 (6), 175 (1), 161 (2), 155 (1), 147 (4), 139 (25), 131 (3), 121 (8), 109 (3), 95 (7), 81 (23), 67 (3), 55(3), 43 (7). GC (HP5-MS): *I* = 3152. ^{1}H-NMR ($CDCl_3$, 500 MHz): δ = 7.71 – 7.67 (m, 4H), 7.44 – 7.35 (m, 6H), 5.38 (tq, $^3J_{H,H}$ = 6.4 Hz, $^4J_{H,H}$ = 1.3 Hz, 1H), 5.10 (tq, $^3J_{H,H}$ = 6.8 Hz, $^4J_{H,H}$ = 1.3 Hz, 1H), 4.22 (dq, $^3J_{H,H}$ = 6.3 Hz, $^4J_{H,H}$ = 0.9 Hz, 2H), 2.36 (t, $^3J_{H,H}$ = 7.4 Hz, 2H), 2.11 (s, 3H), 2.09 – 2.05 (m, 2H), 2.04 – 1.94 (m, 4H), 1.73 – 1.64 (m, 2H), 1.58 (s, 3H), 1.44 (s, 3H), 1.04 (s, 9H) ppm. ^{13}C-NMR ($CDCl_3$, 126 MHz): δ = 209.32 (C_q), 137.09 (C_q), 135.76 (4 x CH), 134.49 (C_q), 134.23 (2 x C_q), 129.63 (2 x CH), 127.72 (4 x CH), 125.03 (CH), 124.25 (CH), 61.29 (CH_2), 43.11 (CH_2), 39.60 (CH_2), 39.03 (CH_2), 30.11 (CH_3), 27.00 (3 x CH_3), 26.41 (CH_2), 21.98 (CH_2), 19.33 (C_q), 16.47 (CH_3), 15.87 (CH_3) ppm.

Synthesis of *tert*-butyldiphenyl(((2*E*,6*E*)-3,7,11-trimethyldodeca-2,6,11-trien-1-yl)oxy)silane (S25)

To a cooled (0 °C) suspension of CH_3PPh_3I (1.75 g, 4.32 mmol, 2.4 eq) in THF, *n*-BuLi (1.6 M in hexane, 2.7 mL, 4.32 mmol, 2.4 eq) was added dropwise. The reaction mixture was stirred at 0 °C for 1 h and then cooled to –78 °C, followed by adding **S24** (0.85 g, 1.83 mmol, in 2 mL THF) dropwise. The reaction mixture was allowed to slowly warm to room temperature overnight and quenched by pouring into ice-water (200 mL). The product was extracted with Et_2O (3 x 100 mL), and the combined organic layers were dried with $MgSO_4$ and concentrated under reduced pressure. Purification via silica gel chromatography (cyclohexane/ethyl acetate, 10:1) provided product **S25** (0.76 g, 1.65 mmol, 90%) as a colorless oil. TLC (cyclohexane/ethyl acetate, 10:1): R_f = 0.46. EI-MS (70 eV): *m*/*z* (%) = 403 (1), 281 (1), 267 (4), 207 (1), 199 (100), 188 (2), 181 (3), 147 (1), 135 (5), 121 (2), 105 (1), 95 (1), 81 (4), 69 (2), 55 (2), 41 (2). GC (HP5-MS): *I* = 3026. ^{1}H-NMR ($CDCl_3$, 500 MHz): δ = 7.72 – 7.67 (m, 4H), 7.43 – 7.36 (m, 6H), 5.39 (tq, $^3J_{H,H}$ = 6.3 Hz, $^4J_{H,H}$ = 1.3 Hz, 1H), 5.11 (tq, $^3J_{H,H}$ = 6.9 Hz, $^4J_{H,H}$ =1.3 Hz, 1H), 4.69 (br s, 1H), 4.66 (br s, 1H), 4.22 (d, $^3J_{H,H}$ = 6.2 Hz, 2H), 2.11 – 2.05 (m, 2H), 2.00 – 1.94 (m, 6H), 1.71 (s, 3H), 1.60 (d, $^3J_{H,H}$ = 1.3 Hz, 3H), 1.56 – 1.49 (m, 2H), 1.44 (d, $^3J_{H,H}$ = 1.2 Hz, 3H), 1.04 (s, 9H) ppm. ^{13}C-NMR ($CDCl_3$, 126 MHz): δ = 146.26 (C_q), 137.18 (C_q), 135.77 (4 x CH), 135.28 (C_q), 134.26 (2 x C_q), 129.62 (2 x CH), 127.71 (4 x CH), 124.27 (CH), 124.21 (CH), 109.87 (CH_2), 61.31 (CH_2), 39.67 (CH_2), 39.41(CH_2), 37.53 (CH_2), 27.00 (3 x CH_3), 26.44 (CH_2), 26.09 (CH_2), 22.58 (CH_3), 19.33 (C_q), 16.48 (CH_3), 16.06 (CH_3) ppm.

Synthesis of (2*E*,6*E*)-3,7,11-trimethyldodeca-2,6,11-trien-1-ol (S26)

Compound **S25** (1.00 g, 2.16 mmol) was dissolved in THF (12 mL) and cooled to 0 °C, followed by adding TBAF (1 M in THF, 2.59 mL, 2.59 mmol, 1.2 eq) dropwise. The reaction solution was stirred at room temperature for 1.5 h and quenched by pouring into ice-water (100 mL). The mixture was extracted with Et_2O (3 x 70 mL), and the combined organic phases were dried with $MgSO_4$ and concentrated under reduced pressure. The product **S26** (0.43 g, 1.93 mmol, 89%) was obtained via silica gel chromatography (pentane/diethyl ether, 1:3) as a colorless oil. TLC (pentane/diethyl ether, 1:3): R_f = 0.29. EI-MS (70 eV): *m*/*z* (%) = 191 (2), 161 (1), 148 (1), 135 (4), 121 (7), 109 (16), 95 (36), 81 (100), 67 (23), 55 (31), 41 (34). GC (HP5-MS): *I* = 1714. ^{1}H-NMR (C6D6, 700 MHz): δ = 5.40 (tq, $^3J_{H,H}$ = 6.7 Hz, $^4J_{H,H}$ = 1.3 Hz, 1H), 5.21 (tq, $^3J_{H,H}$ = 7.0 Hz, $^3J_{H,H}$ = 1.3 Hz, 1H), 4.85 – 4.79 (m, 2H), 4.01 – 3.92 (m, 2H), 2.15 – 2.09 (m, 2H), 2.03 – 1.93 (m, 6H), 1.66 (s, 3H), 1.55 (d, $^4J_{H,H}$ = 1.1 Hz, 3H), 1.58 – 1.52 (m, 2H), 1.47 (d, $^4J_{H,H}$ = 1.4 Hz, 3H), 0.56 (t, $^3J_{H,H}$ = 5.6 Hz, 1H) ppm. ^{13}C-NMR (C_6D_6, 176 MHz): δ = 145.84 (C_q), 138.08 (C_q), 135.28 (C_q), 125.04 (CH), 124.58 (CH), 110.41(CH_2), 59.39 (CH_2), 39.89 (CH_2), 39.63 (CH_2), 37.67 (CH_2), 26.70 (CH_2), 26.31 (CH_2), 22.51 (CH_3), 16.17 (CH_3), 15.99 (CH_3) ppm.

Synthesis of trisammonium (2*E*,6*E*)-3,7,11-trimethyldodeca-2,6,11-trien-1-yl diphosphate (15)

To a cooled (0 °C) solution of **S26** (150 mg, 0.68 mmol) in Et_2O (10 mL), PBr_3 was added (73.6 mg, 0.27 mmol, 0.4 eq) dropwise. The mixture was stirred at 0 °C for 1 h, and then quenched by pouring into ice-water (100 mL). The product was extracted with Et_2O (3 x 50 mL), and the organic layers were combined and dried with $MgSO_4$. The solvent was removed carefully under reduced pressure. The residue was added dropwise to a solution of $(NBu_4)_3HP_2O_7$ (1.23 g, 1.36 mmol, 2.0 eq) in acetonitrile (1 mL) and stirred at room temperature overnight. The solvent was removed by evaporation, and the residue was dissolved in aq. NH_4HCO_3 (25 mM, 3 mL) and loaded to DOWEX 50WX8 cation exchange column (NH_4^+ form, pH ~ 7.0). The product was eluted using 1.5 column volumes of wash solution (2% *i*PrOH in 25 mM NH_4HCO_3) and lyophilisation yielded compound **15** (256 mg, 0.59 mmol, 87%) as a white powder. ^{1}H-NMR (D_2O, 500 MHz): δ = 5.45 (t, $^3J_{H,H}$ = 7.0 Hz, 1H), 5.19 (t, $^3J_{H,H}$ = 5.7 Hz, 1H), 4.75 – 4.68 (m, 2H), 4.46 (t, $^3J_{H,H}$ = 6.6 Hz, 2H), 2.20 – 2.04 (m, 2H), 2.02 – 1.90 (m, 6H), 1.71 (d, $^4J_{H,H}$ = 1.3 Hz, 3H), 1.70 (d, $^4J_{H,H}$ = 1.2 Hz, 3H), 1.61 (d, $^4J_{H,H}$ = 1.3 Hz, 3H), 1.55 – 1.49 (m, 2H) ppm. ^{13}C-NMR (D_2O, 126 MHz): δ = 147.60 (C_q), 142.59 (C_q), 136.51 (C_q), 124.32 (CH), 120.04 (d, $^3J_{C,P}$ = 8.7 Hz), 109.51 (CH_2), 62.46 (d, $^2J_{C,P}$ = 5.2 Hz, CH_2), 39.10 (CH_2), 38.71 (CH_2), 36.86 (CH_2), 25.90 (CH_2), 25.41 (CH_2), 21.81 (CH_3), 15.78 (CH_3), 15.32 (CH_3) ppm. ^{31}P NMR (D_2O, 202 MHz): δ = –6.28 (d, $^2J_{P,P}$ = 21.6 Hz, 1P), –10.15 (d, $^2J_{P,P}$ = 21.5 Hz, 1P) ppm.

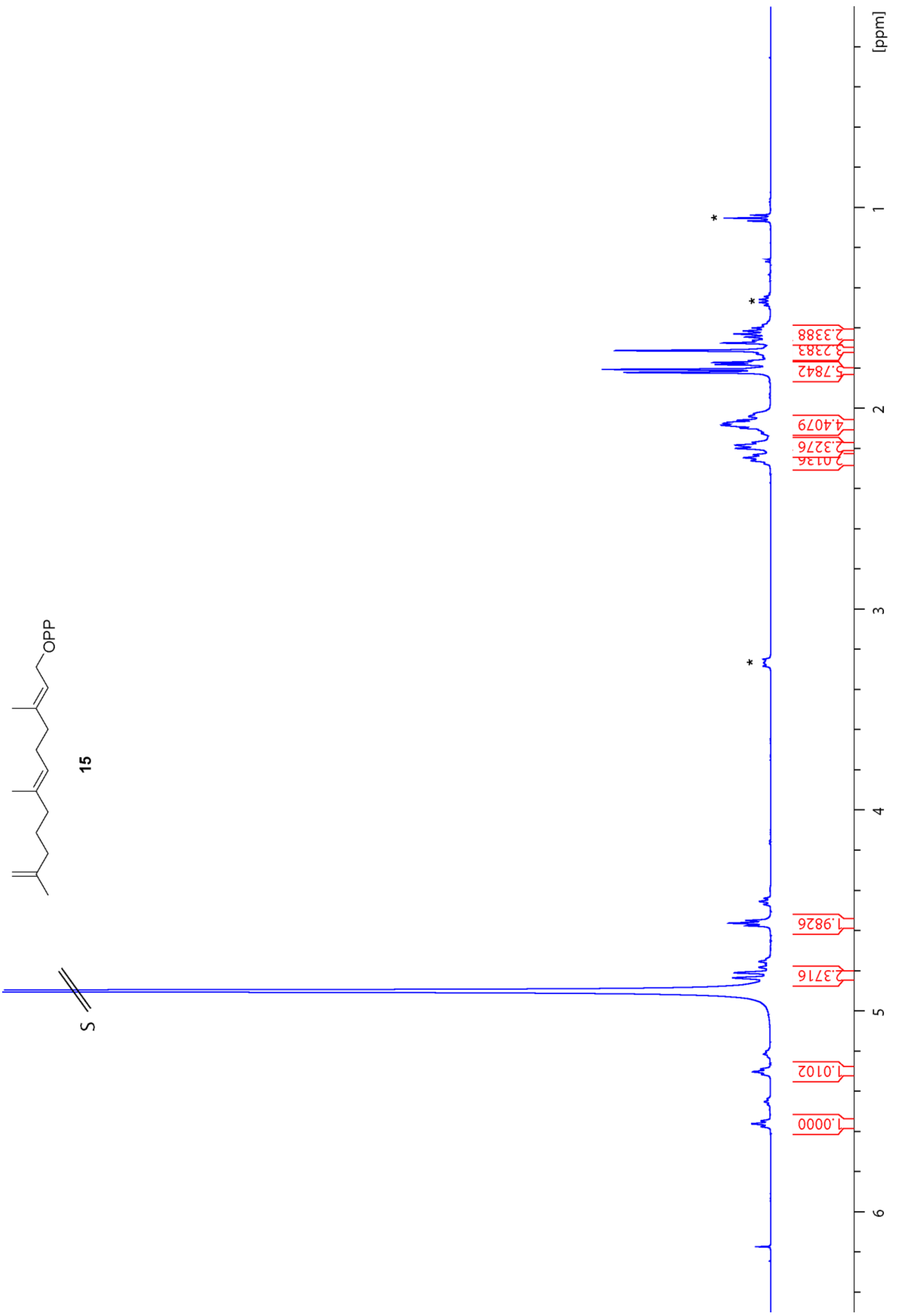

Figure S45. ^{1}H-NMR spectrum (D_2O, 500 MHz) of **15**. S indicates solvent peak. Asterisks indicate signals from residual tetrabutyl ammonium salt.

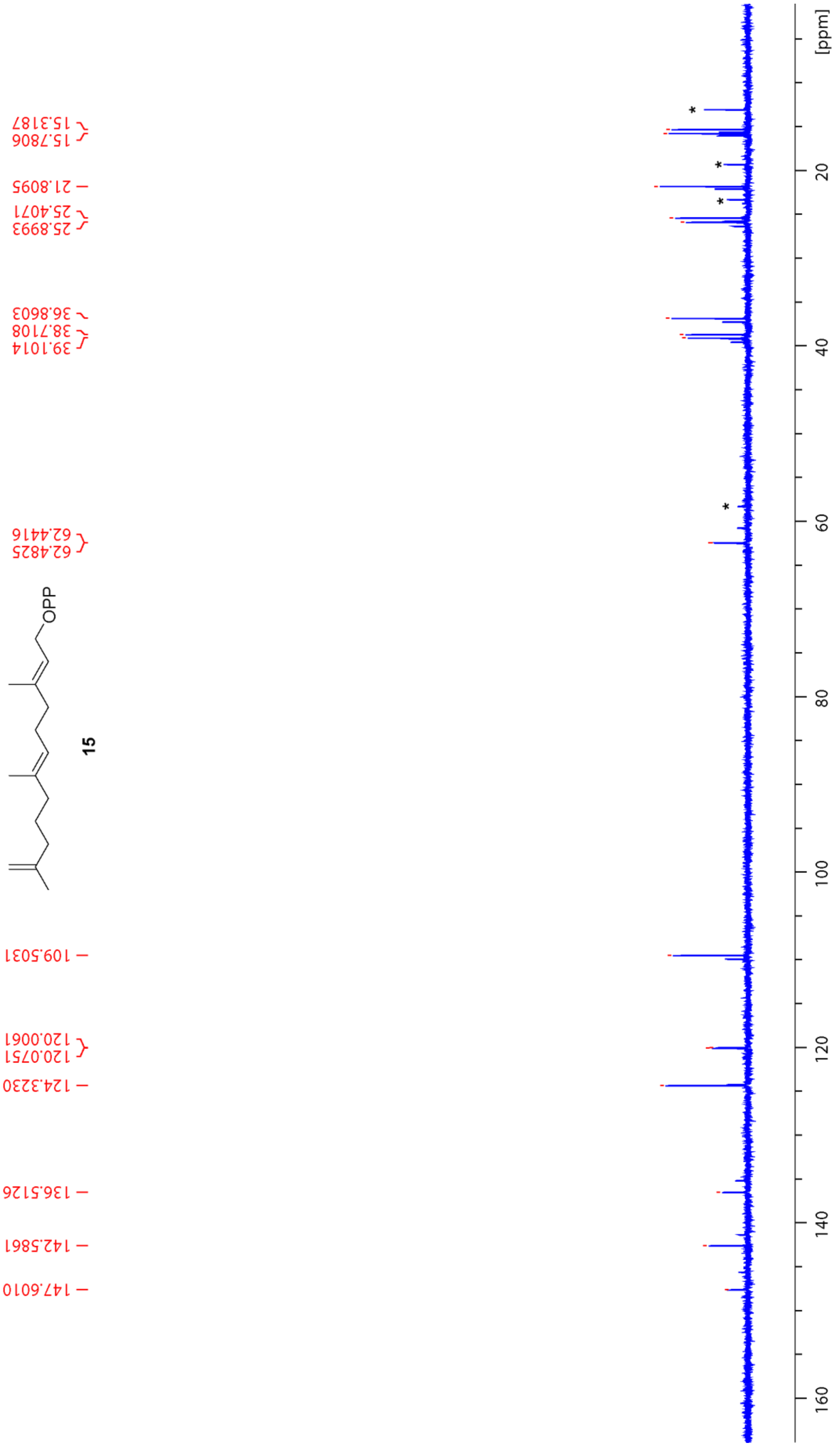

Figure S46. ^{13}C-NMR spectrum (D_2O, 126 MHz) of **15**. Asterisks indicate signals from residual tetrabutyl ammonium salt.

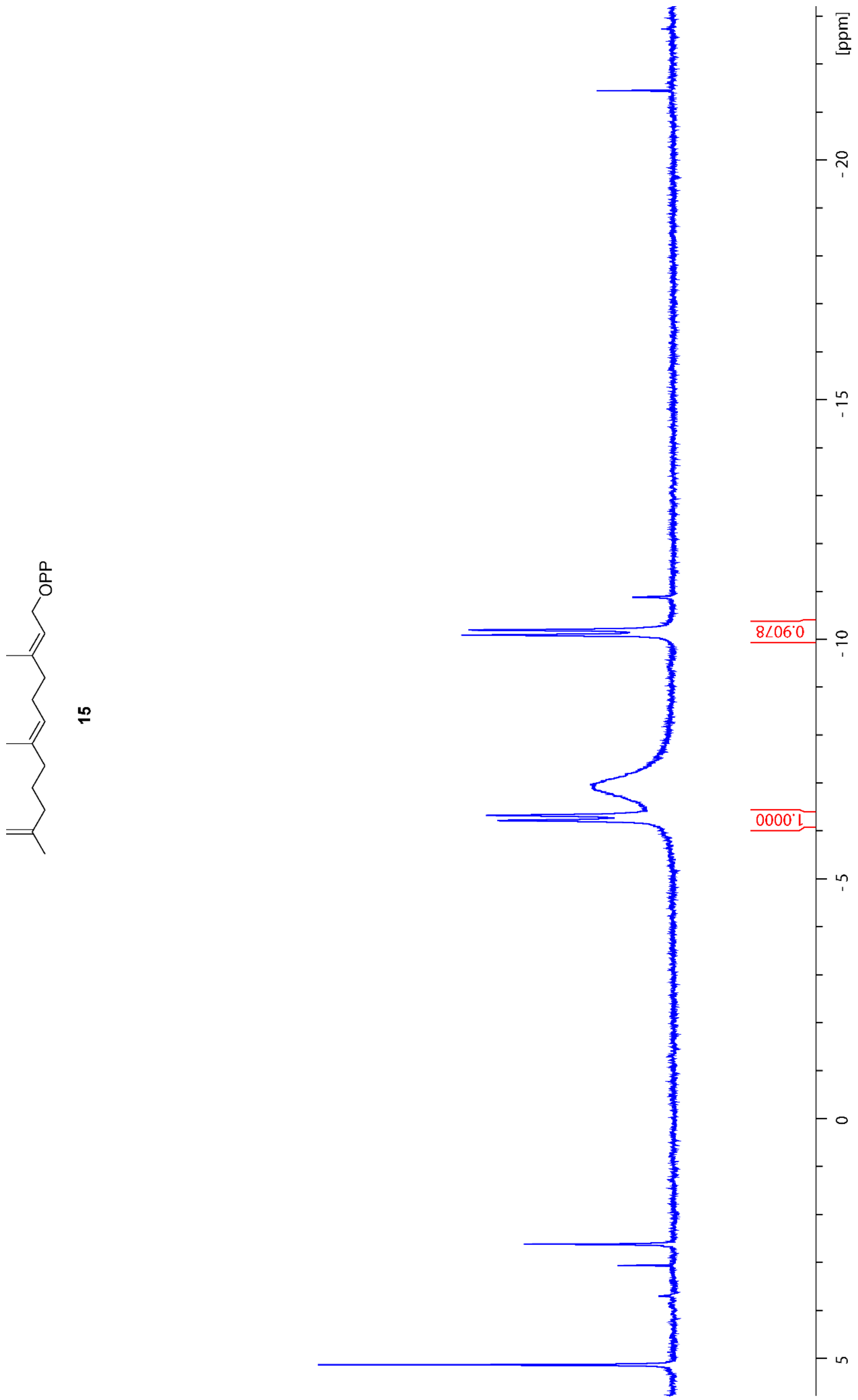

Figure S47. ^{31}P-NMR spectrum (D_2O, 202 MHz) of **15**.

Synthetic route to trisammonium (2*E*,6*E*)-3,7-dimethyl-11-oxododeca-2,6-dien-1-yl diphosphate (16)

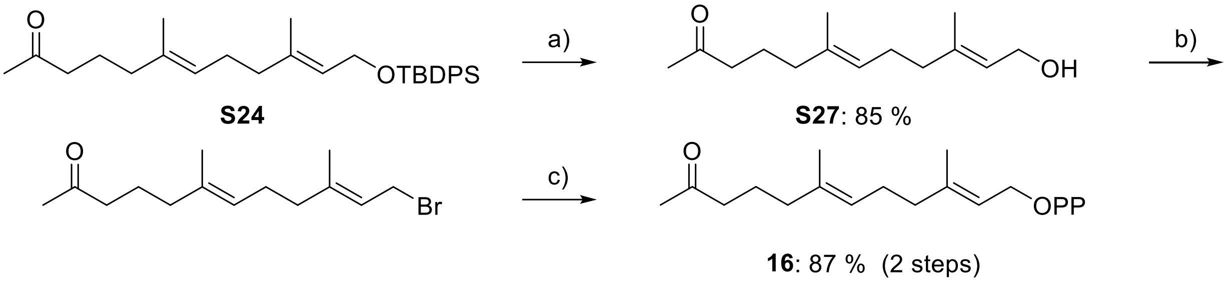

Scheme S5. Synthesis of FPP analog **16**. Reaction conditions: a) TBAF, THF, 1.5 h; b) PBr_3, Et_2O, 0 °C, 1 h; c) $(NBu_4)_3HP_2O_7$, MeCN, overnight.

Compounds **S27** and **16** were synthesized by the same procedures as mentioned for compounds **S26** and **15** above. Through these methods **S24** (1.0 g, 2.16 mmol) was converted into **S27** (433 mg, 1.93 mmol, 89%) and **S27** (200 mg, 0.89 mmol) was converted into **16** (293 mg, 76%).

(2*E*,6*E*)-3,7-Dimethyl-11-oxododeca-2,6-dien-1-ol (**S27**): TLC (pentane/diethyl ether, 1:3): R_f = 0.29. EI-MS (70 eV): *m*/*z* (%) = 193 (1), 173 (2), 163 (1), 148 (8), 138 (15), 121(29), 107 (6), 95 (36), 81 (100), 67 (19), 55 (16), 43 (84). GC (HP5-MS): *I* = 1824. ^{1}H-NMR (C_6D_6, 700 MHz): δ = 5.40 (tq, $^3J_{H,H}$ = 8.0 Hz, $^4J_{H,H}$ = 1.3 Hz, 1H), 5.14 (tq, $^3J_{H,H}$ = 7.0 Hz, $^4J_{H,H}$ = 1.3 Hz, 1H), 4.00 (t, $^3J_{H,H}$ = 5.3 Hz, 2H), 2.12 – 2.07 (m, 2H), 2.00 – 1.96 (m, 2H), 1.94 (t, $^3J_{H,H}$ = 7.3 Hz, 2H), 1.90 – 1.85 (m, 2H), 1.67 (s, 3H), 1.65 – 1.58 (m, 2H), 1.51 (d, $^4J_{H,H}$ = 1.0 Hz, 3H), 1.47 (d, $^4J_{H,H}$ = 1.3 Hz, 3H) ppm. ^{13}C-NMR (C_6D_6, 176 MHz): δ = 206.55 (C_q), 137.75 (C_q), 134.80 (C_q), 125.27 (CH), 125.01 (CH), 59.40 (CH_2), 42.58 (CH_2), 39.78 (CH_2), 39.22 (CH_2), 29.40 (CH_3), 26.51 (CH_2), 22.14 (CH_2), 16.13 (CH_3), 15.83 (CH_3) ppm.

Trisammonium (2*E*,6*E*)-3,7-dimethyl-11-oxododeca-2,6-dien-1-yl diphosphate (16): ^{1}H-NMR (D_2O, 500 MHz): δ = 5.42 (td, $^3J_{H,H}$ = 7.1 Hz, $^4J_{H,H}$ = 1.5 Hz, 1H), 5.15 (t, $^3J_{H,H}$ = 6.8 Hz, 1H), 4.42 (t, $^3J_{H,H}$ = 6.6 Hz, 2H), 2.46 (t, $^3J_{H,H}$ = 7.3 Hz, 2H), 2.15 (s, 3H), 2.14 – 2.09 (m, 2H), 2.08 – 2.04 (m, 2H), 1.94 (t, $^3J_{H,H}$ = 7.2 Hz, 2H), 1.68 (d, $^4J_{H,H}$ = 1.3 Hz, 3H), 1.64 – 1.60 (m, 2H), 1.55 (d, $^4J_{H,H}$ = 1.2 Hz, 3H) ppm. ^{13}C-NMR (D_2O, 126 MHz): δ = 217.56 (C_q), 142.73 (C_q), 135.89 (C_q), 125.06 (CH), 119.94 (d, $^3J_{C,P}$ = 8.6 Hz, CH), 62.48 (d, $^2J_{C,P}$ = 5.2 Hz, CH_2), 42.49 (CH_2), 38.81 (CH_2), 38.16 (CH_2), 29.44 (CH_3), 25.61 (CH_2), 21.39 (CH_2), 15.65 (CH_3), 14.90 (CH_3) ppm. ^{31}P NMR (D_2O, 202 MHz): δ = –6.38 (d, $^2J_{P,P}$ = 21.9 Hz, 1P), –10.20 (d, $^2J_{P,P}$ = 21.8 Hz, 1P) ppm.

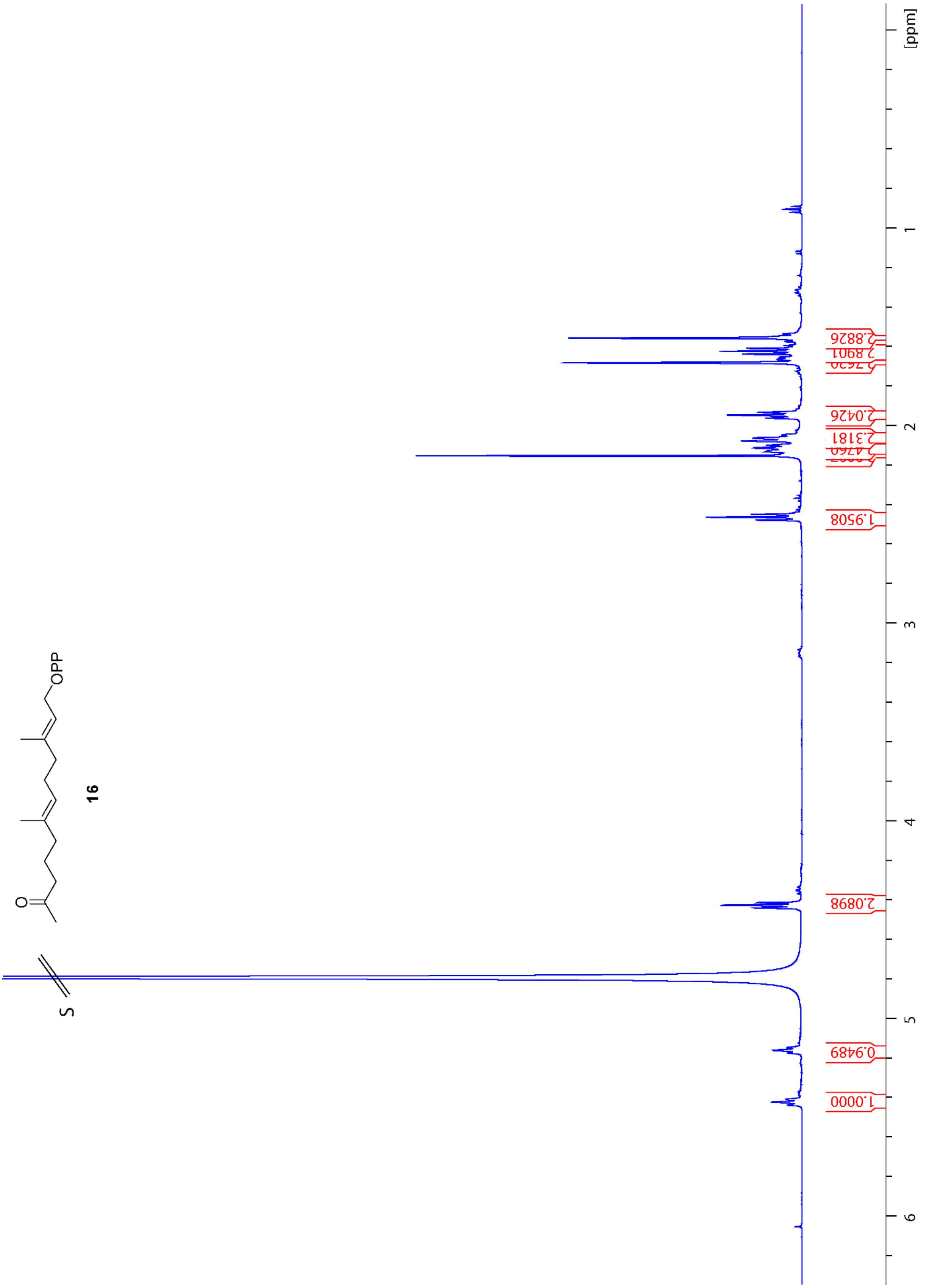

Figure S48. ^{1}H-NMR spectrum (D_2O, 500 MHz) of **16**. S indicates solvent peak.

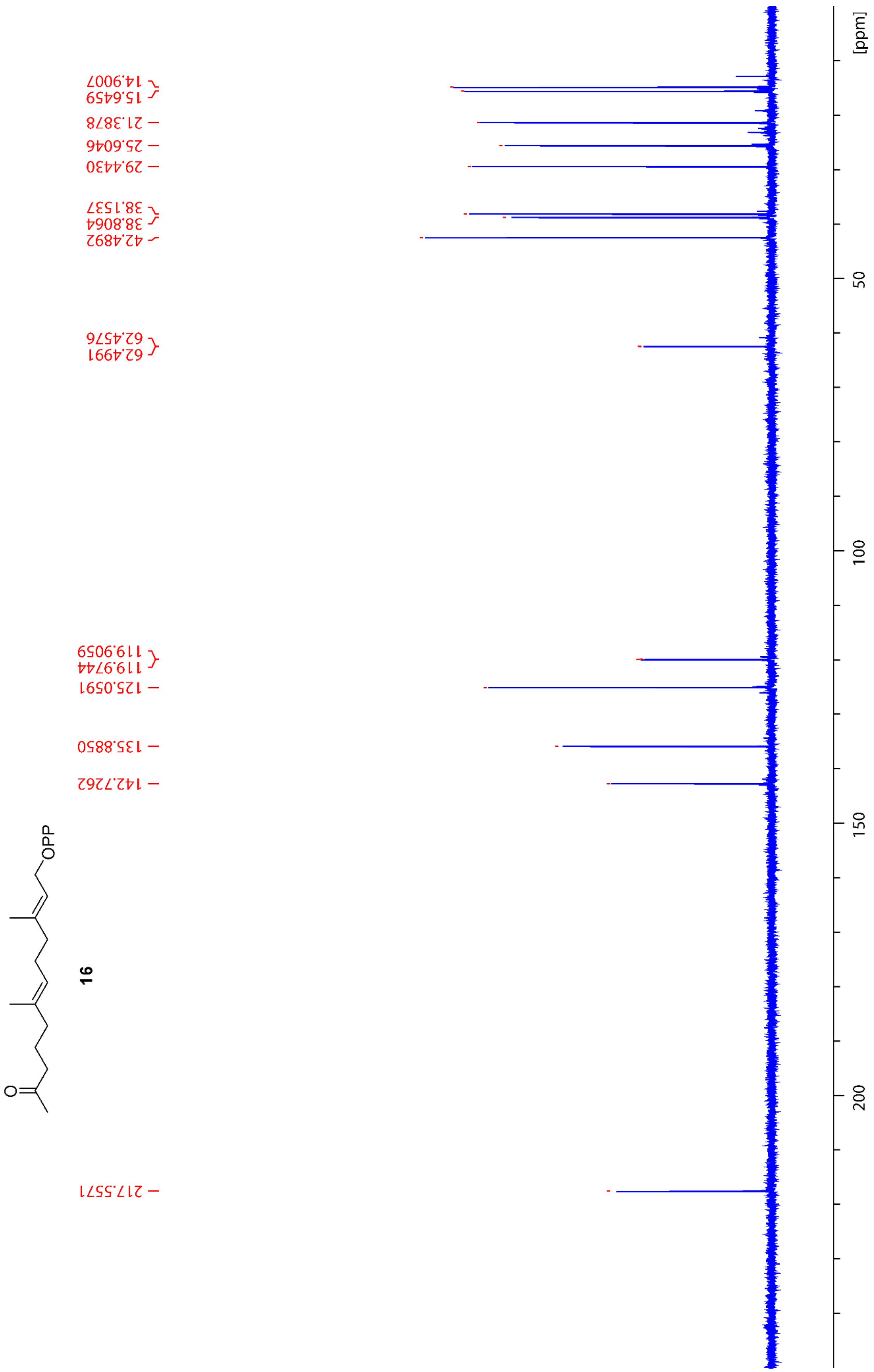

Figure S49. ^{13}C-NMR spectrum (D_2O, 126 MHz) of **16**.

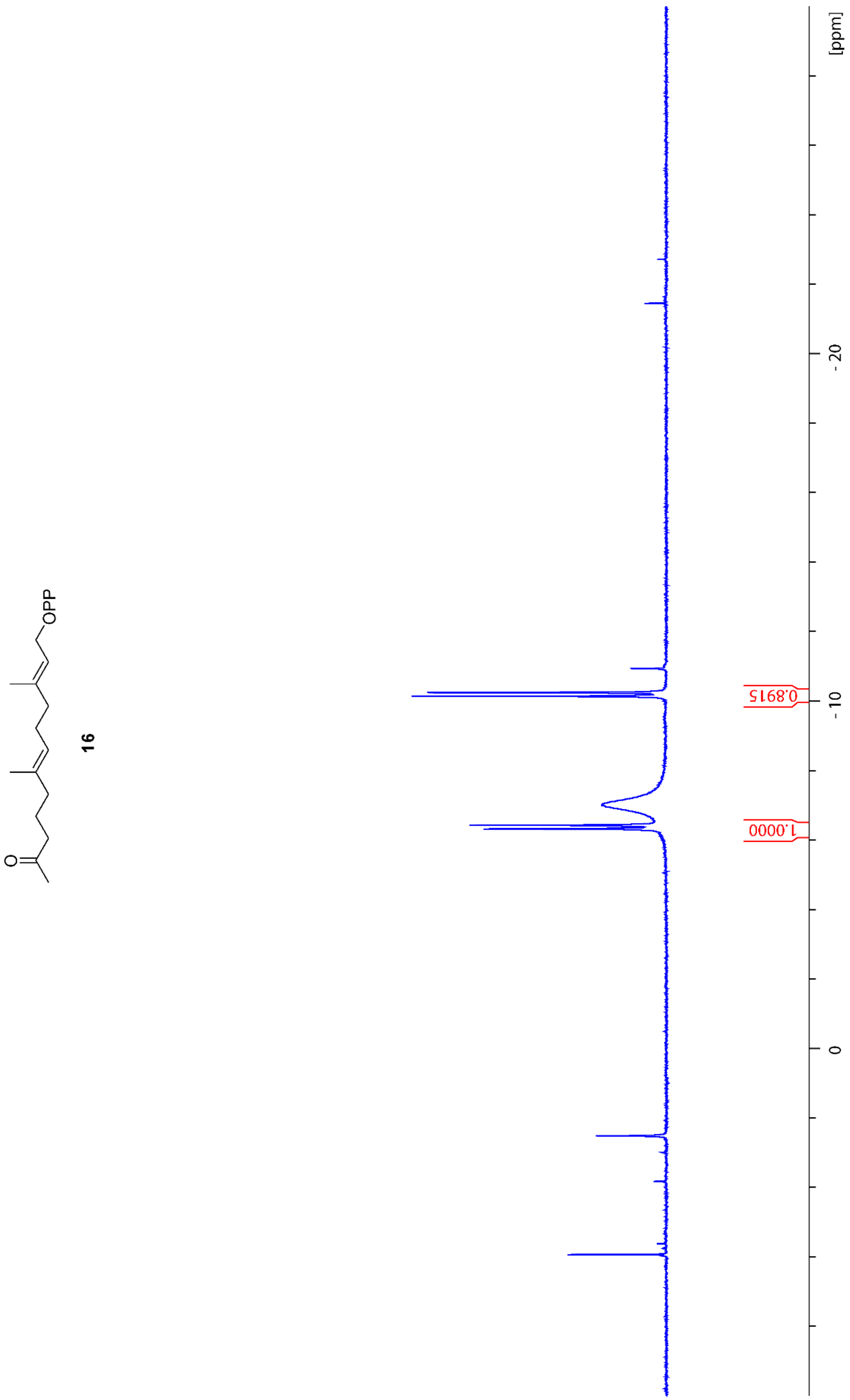

Figure S50. ^{31}P-NMR spectrum (D_2O, 202 MHz) of **16**.

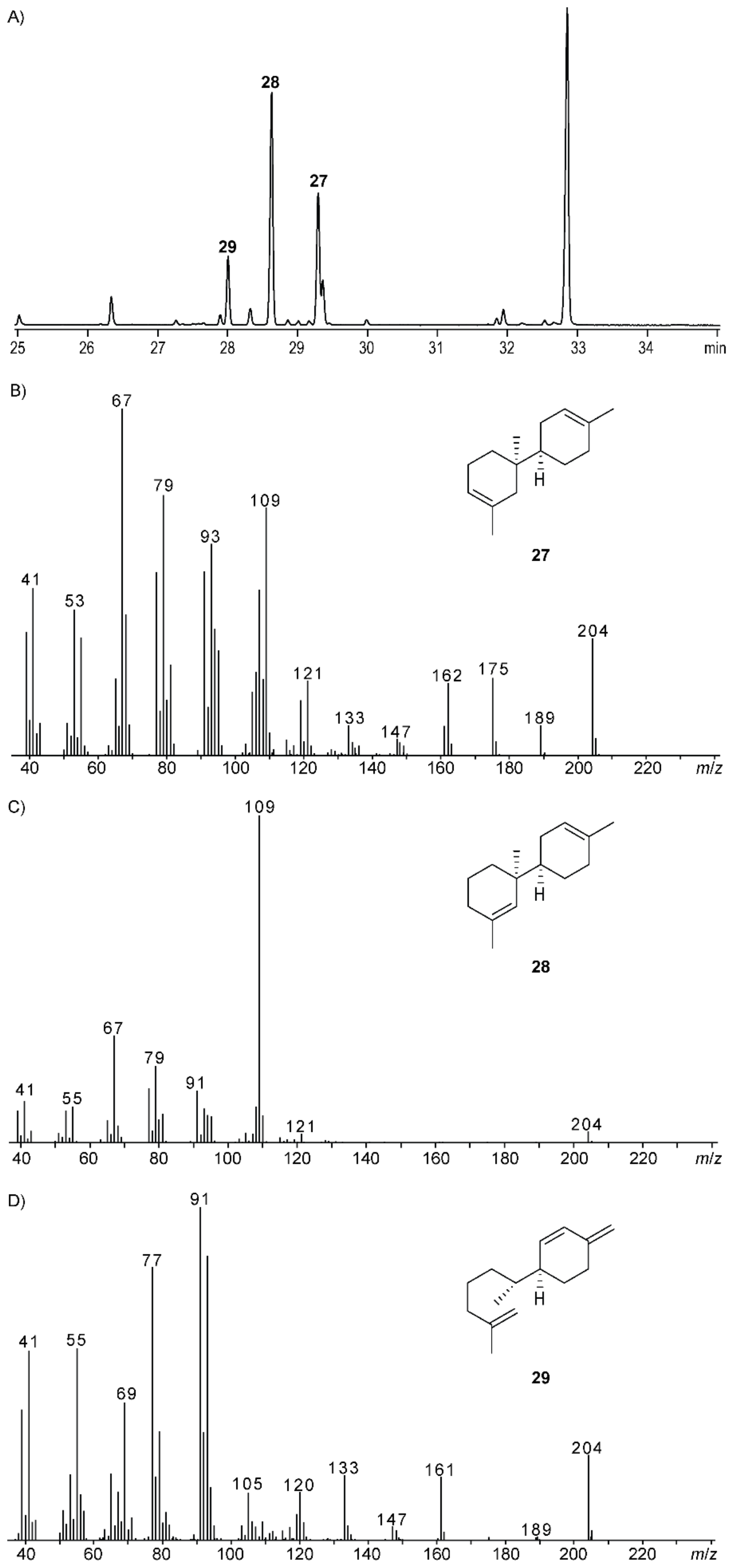

Figure S51. Incubation of DcS with FPP analogue (**15**). A) Total ion chromatogram of the crude mixture. EI-MS spectra of compounds B) **27**, C) **28** and D) **29**.

Isolation of compounds 27, 28 and 29

A purified DcS preparation (120 mL) was obtained from 12 L *E.coli* BL21 culture. Then 120 small scale enzymatic reactions were performed consisting of: incubation buffer (5 mL), protein solution (1 mL), binding buffer (4 mL) and compound **15** (2.5 mg/mL in 25 mM NH_4HCO_3, 0.5 mL). The mixtures were incubated at 28 °C overnight, combined and extracted with pentane (3 x 100 mL), the combined organic layers were concentrated under reduce pressure, the crude residue was purified via silica gel chromatography (pentane) to afford compound **28** and **29**. Compound **27** was obtained via silver nitrate coated TLC[9] (cyclohexane/ethyl acetate, 4:1).

Tenuifola-2,11-diene, (1*R*,1'*R*)-1,3,4'-trimethyl-[1,1'-bi(cyclohexane)]-3,3'-diene (27): Colorless oil. Yield: 0.6 mg, 0.003 mmol, 0.9%. TLC (pentane): R_f = 0.68. Optical rotation: $[\alpha]_D^{20}$ = +41.7 (*c* 0.06, CH_2Cl_2). GC (HP5-MS): *I* = 1580. HRMS (APCI): *m*/*z* = 204.1873 (calc. for $[C_{15}H_{24}]^+$ = 204.1873). MS (EI, 70 eV) = 204 (40), 189 (9), 175 (25), 162 (22), 147 (5), 133 (9), 121 (21), 109 (75), 93 (64), 79 (75), 67 (100), 53 (40), 41 (47) (Figure S51B). IR (diamond ATR): $\tilde{\nu}$ = 2960 (m), 2922 (s), 2852 (m), 1738 (m), 1716 (w), 1678 (w), 1442 (m), 1376 (m), 1345 (w), 1242 (m), 1019 (w), 923 (w), 816 (w), 794 (m), 721 (w), 578 (m), 543 (w), 431 (m) cm^{-1}. NMR data are given in Table S5 and Figures S53 – S59.

Tenuifola-2,10-diene, (1*S*,1'*R*)-1,3,4'-trimethyl-[1,1'-bi(cyclohexane)]-2,3'-diene (28): Colorless oil. Yield: 1.1 mg, 0.005 mmol, 1.4%. TLC (pentane): R_f = 0.69. Optical rotation: $[\alpha]_D^{20}$ = +60.9 (*c* 0.11, CH_2Cl_2). GC (HP5-MS): *I* = 1551. HRMS (EI): *m*/*z* = 204.1862 (calc. for $[C_{15}H_{24}]^+$ = 204.1873). MS (EI, 70 eV) = 204 (3), 121 (3), 109 (100), 91 (16), 79 (24), 67 (33), 55 (11), 41 (13) (Figure S51C). IR (diamond ATR): $\tilde{\nu}$ = 2960 (m), 2926 (s), 2867 (m), 2856 (m), 2832 (m), 1738 (m), 1670 (w), 1455 (m), 1437 (m), 1375 (m), 1345 (w), 1217 (w), 1204 (w), 1019 (w), 797 (m), 543 (m) cm^{-1}. NMR data are given in Table S6 and Figures S60 – S66.

***iso*-β-Sesquiphellandrene, (*S*)-3-methylene-6-((*R*)-6-methylhept-6-en-2-yl)cyclohex-1-ene (29)**: Colorless oil. Yield: 0.3 mg, 0.001 mmol, 0.4%. TLC (pentane): R_f = 0.68. Optical rotation: $[\alpha]_D^{20}$ = +3.3 (*c* 0.03, CH_2Cl_2). GC (HP5-MS): *I* = 1521. HRMS (QToF): *m*/*z* = 204.1898 (calc. for $[C_{15}H_{24}]^+$ = 204.1873). MS (EI, 70 eV) = 204 (22), 189 (21), 161 (23), 147 (4), 133 (19), 120 (16), 105 (15), 91 (100), 77 (76), 69 (39), 55 (55), 41 (54) (Figure S51D). IR (diamond ATR): $\tilde{\nu}$ = 2955 (m), 2923 (s), 2853 (m), 1737 (m), 1650 (w), 1459 (m), 1376 (m), 1260 (m), 1243 (w), 1093 (w), 1019 (m), 879 (m), 800 (m), 543 (m) cm^{-1}. NMR data are given in Table S7 and Figures S67 – S73.

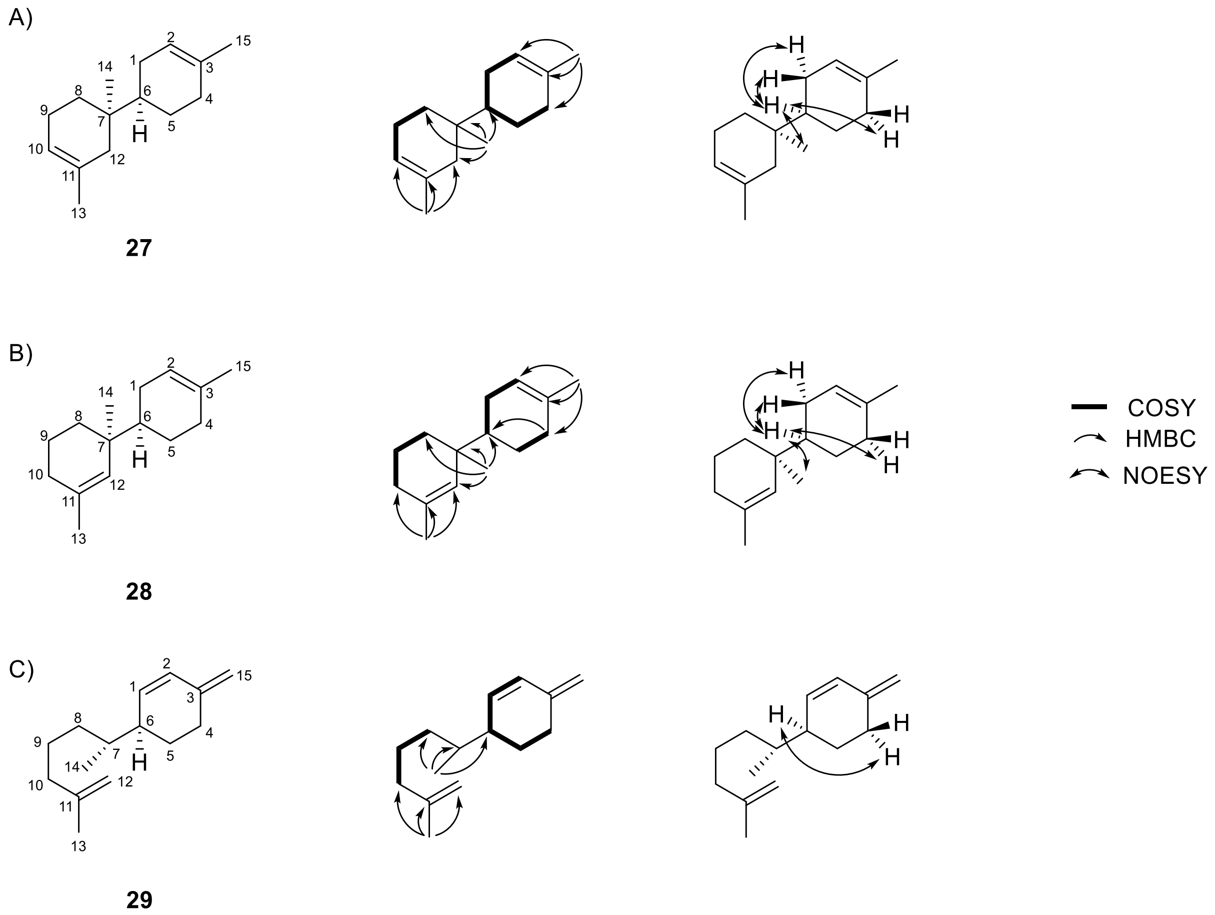

Figure S52. Structure elucidation of A) compound **27**, B) compound **28** and C) compound **29**. Bold lines indicate ^{1}H,^{1}H-COSY correlations, single headed arrows HMBC correlations and double headed arrows NOESY correlation.

Table S5. NMR data of **27** recorded in C_6D_6.

C[a]		^1H[b]	^{13}C[b]
1	CH_2	2.00 – 1.95 (m, 1H)	26.7
		1.82 – 1.76 (m, 1H)	
2	CH	5.47 – 5.44 (m, 1H)	121.9
3	C_q	–	133. 6
4	CH_2	1.95 – 1.91 (m, 1H)	31.9
		1.90 – 1.85 (m, 1H)	
5	CH_2	1.70 (ddt, *J* = 12.3, 5.6, 2.2 Hz, 1H)	23.9
		1.20 (dd, *J* = 12.1, 5.5 Hz, 1H)	
6	CH	1.37 – 1.32 (m, 1H)	42.9
7	C_q	–	33.8
8	CH_2	1.29 (dd, *J* = 7.2, 5.9 Hz, 2H)	31.8
9	CH_2	2.01 – 1.96 (m, 2H)	23.1
10	CH	5.42 – 5.40 (m, 1H)	120.3
11	C_q	–	132.5
12	CH_2	1.84 – 1.80 (m, 1H)	41.3
		1.60 – 1.56 (m, 1H)	
13	CH_3	1.64 (s, 3H)	24.4
14	CH_3	0.78 (s, 3H)	19.8
15	CH_3	1.66 (s, 3H)	23.6

[a] Carbon numbering as shown in Figure S52. [b] Chemical Shifts δ in ppm, coupling constants *J* in Hertz.

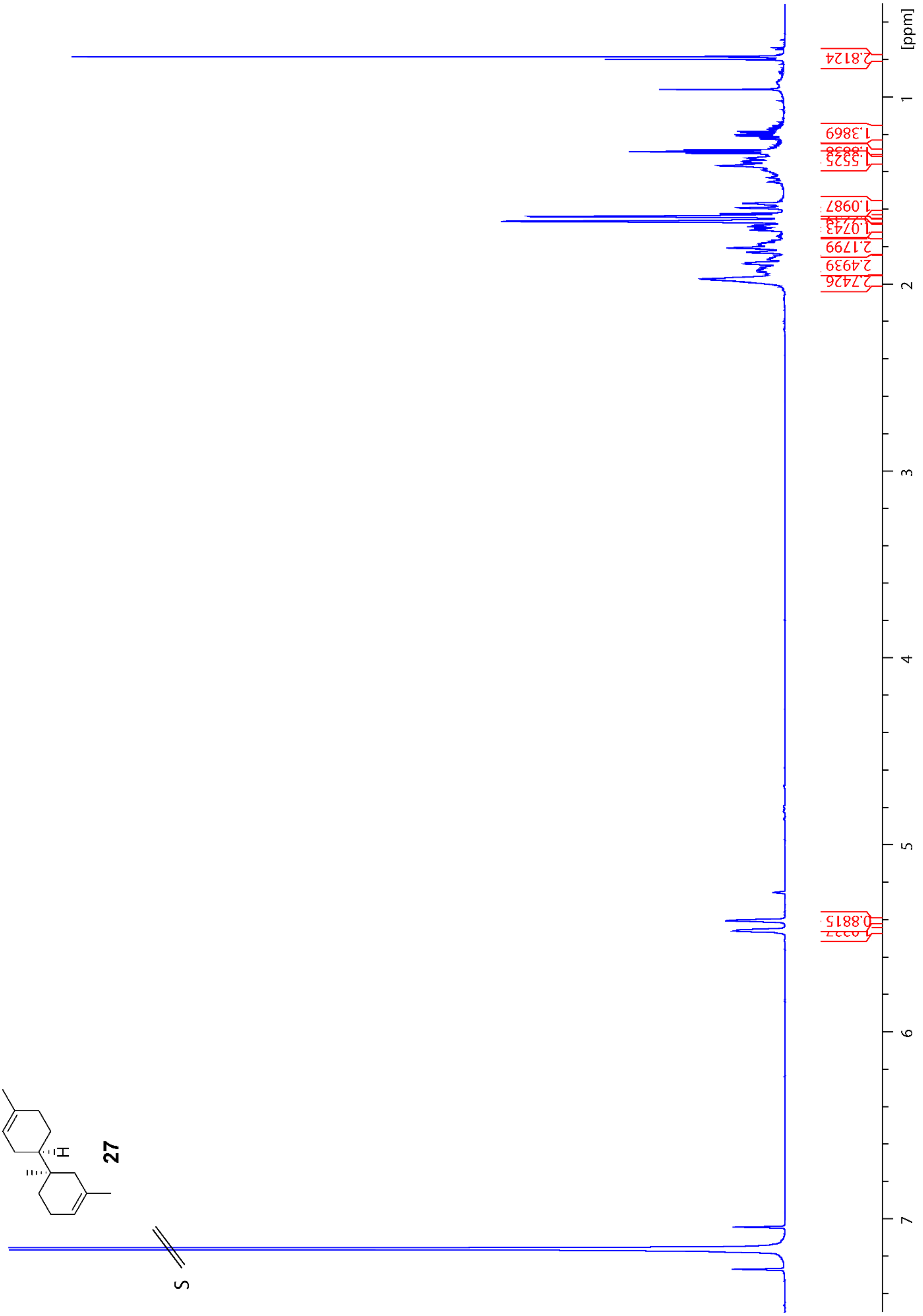

Figure S53. ^{1}H-NMR spectrum (C_6D_6, 700 MHz) of **27**. S indicates solvent peak.

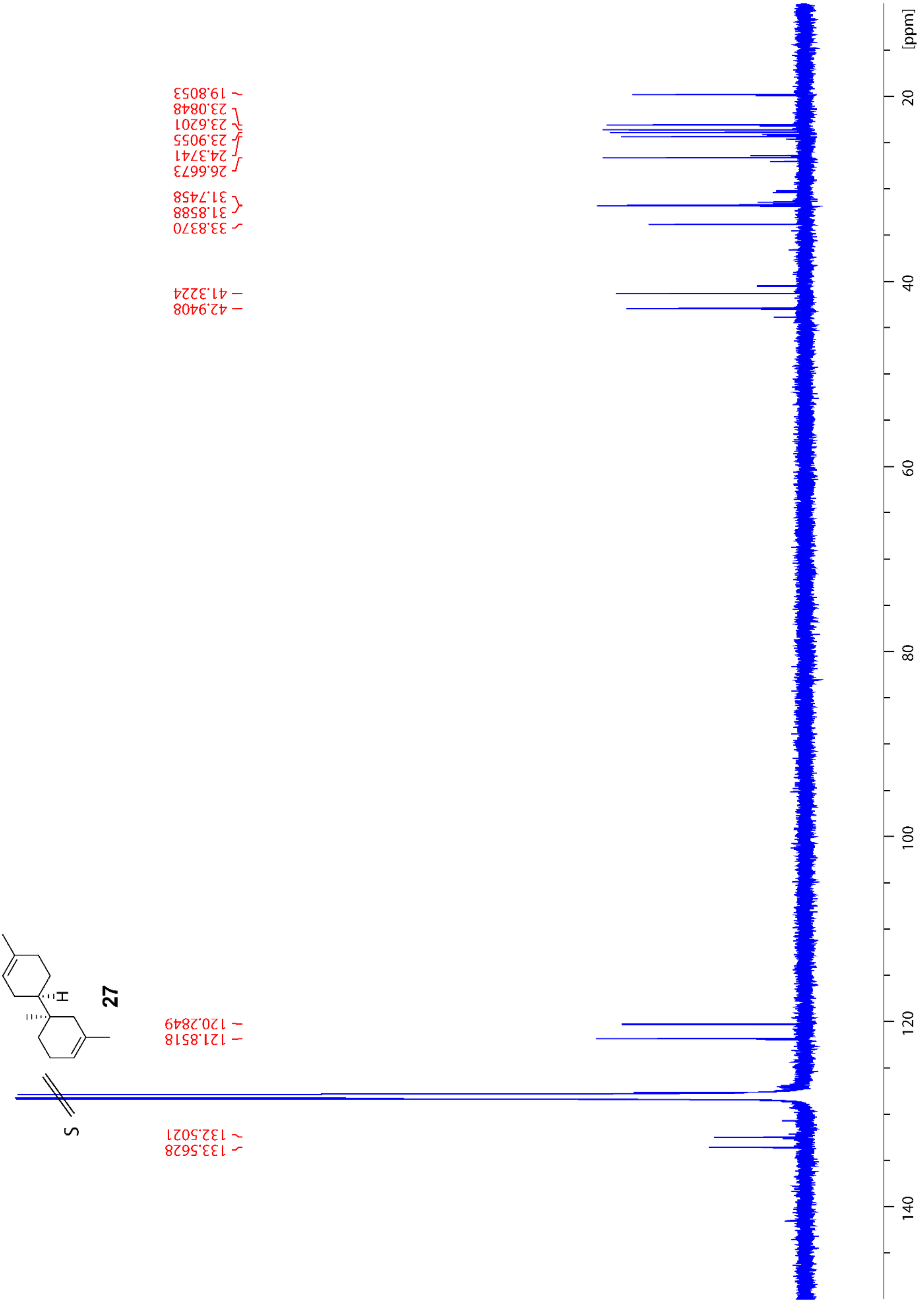

Figure S54. ^{13}C-NMR spectrum (C_6D_6, 175 MHz) of **27**. S indicates solvent peak.

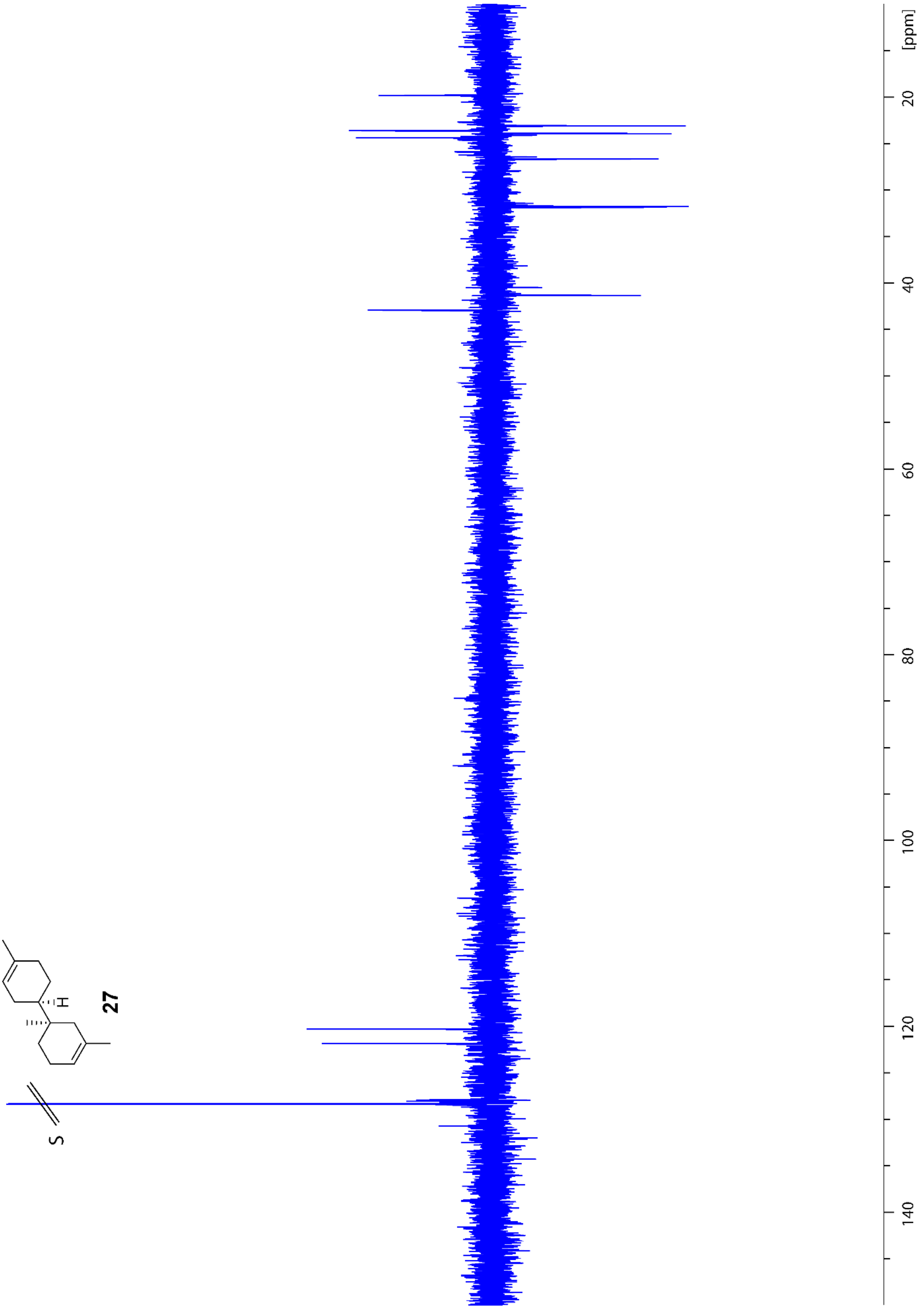

Figure S55. ^{13}C-DEPT135 spectrum (C_6D_6, 175 MHz) of **27**. S indicates solvent peak.

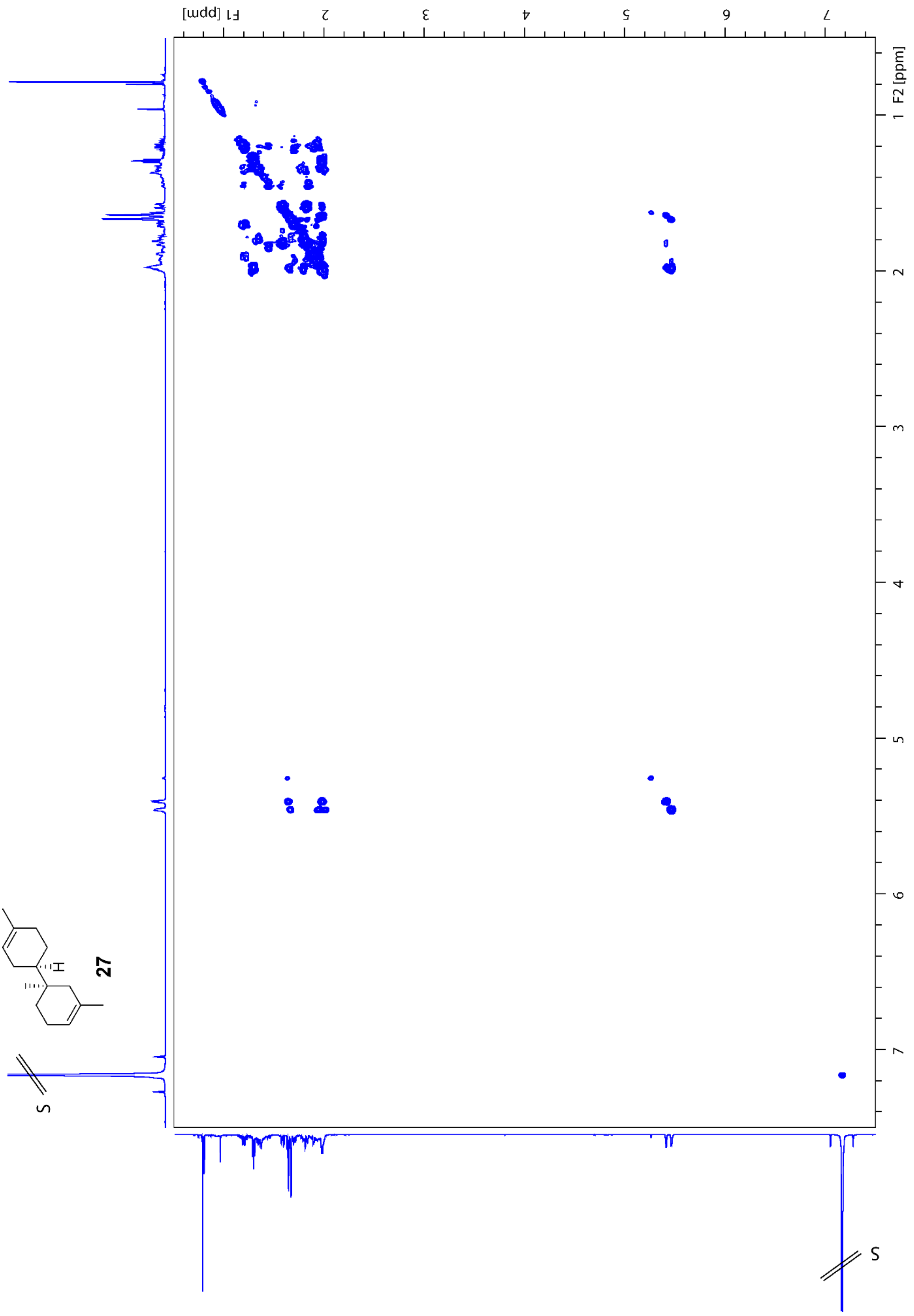

Figure S56. $^{1}H,^{1}H$-COSY spectrum (C_6D_6) of **27**. S indicates solvent peaks.

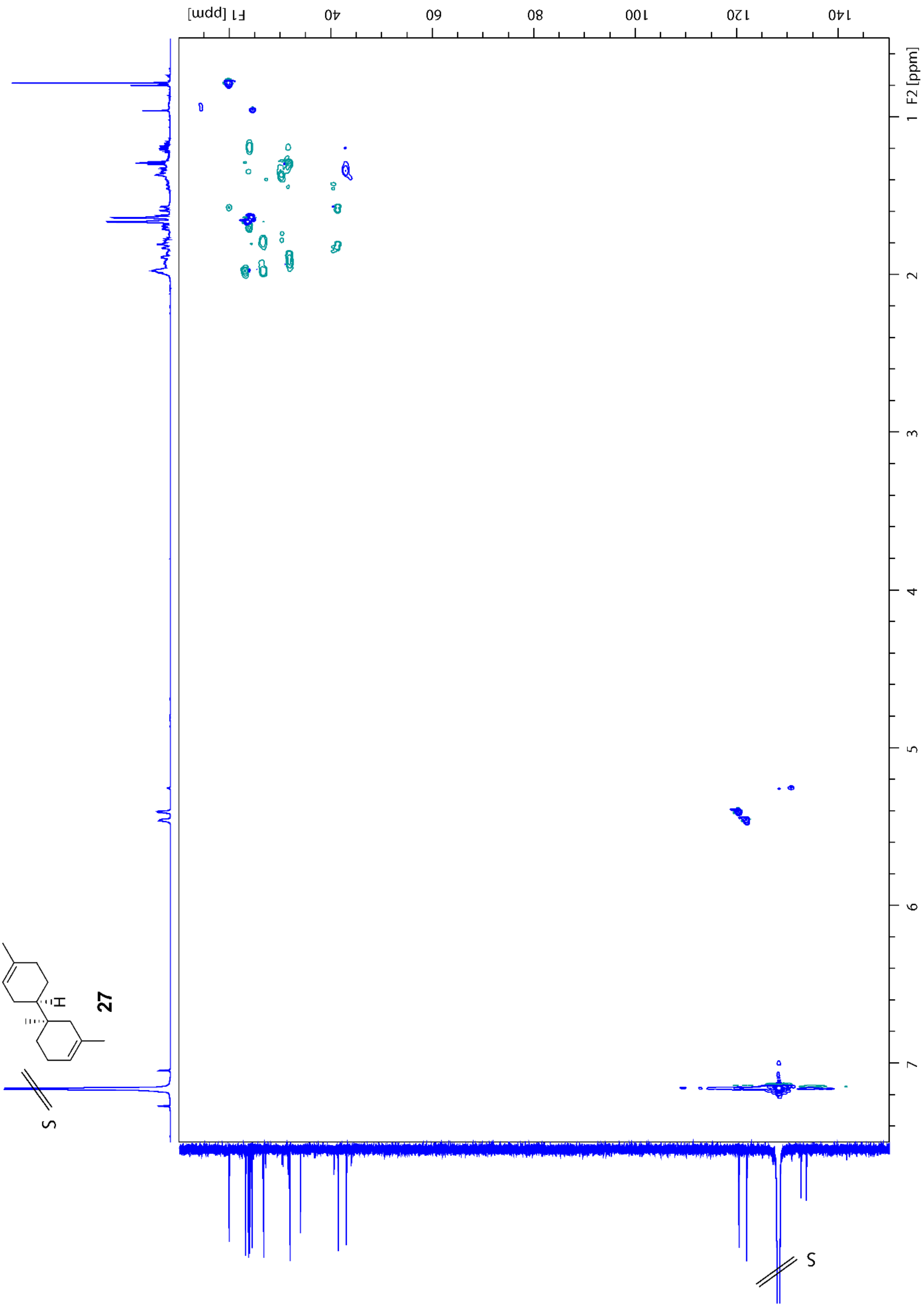

Figure S57. HSQC spectrum (C_6D_6) of **27**. S indicates solvent peaks.

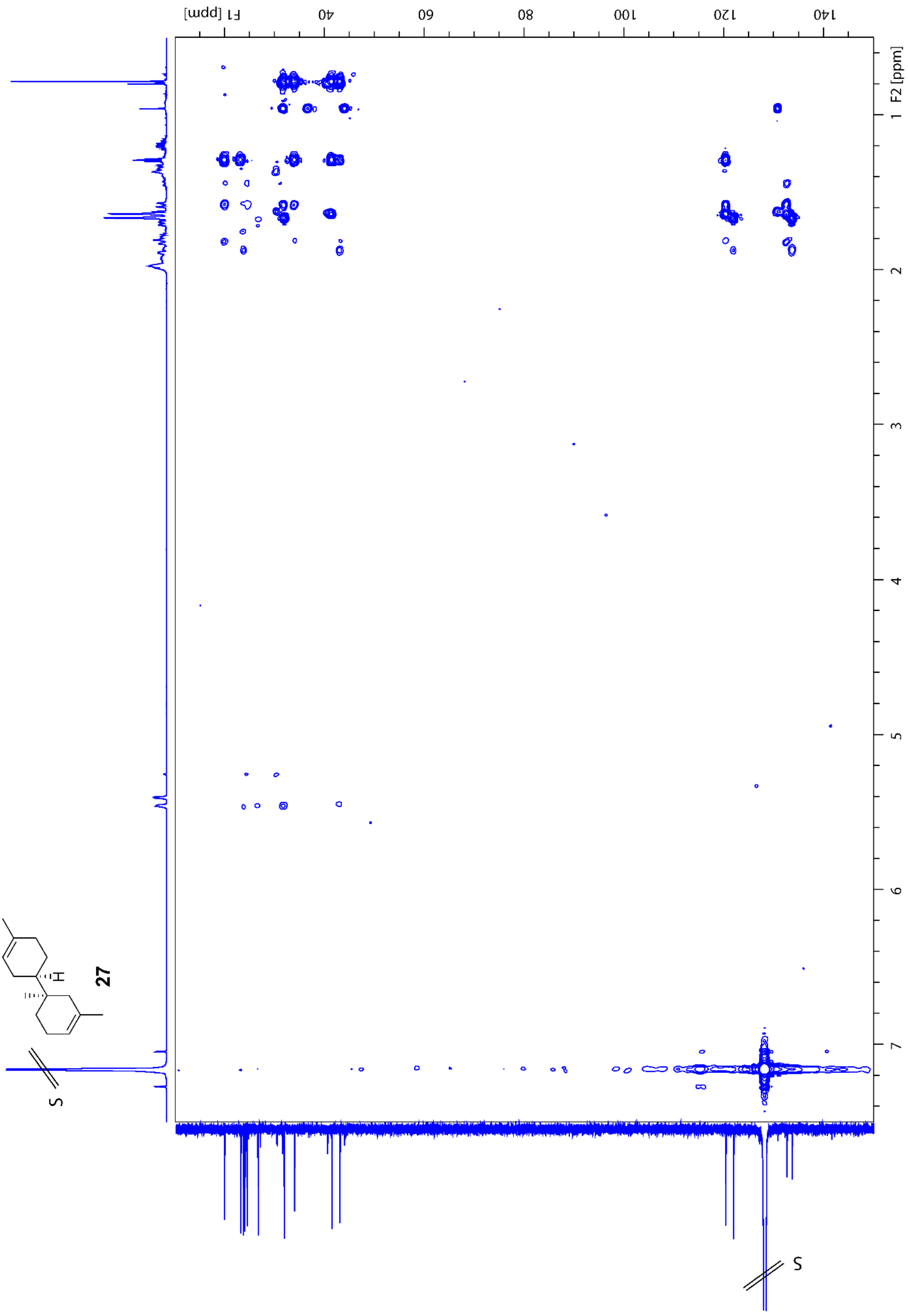

Figure S58. HMBC spectrum (C_6D_6) of **27**. S indicates solvent peaks.

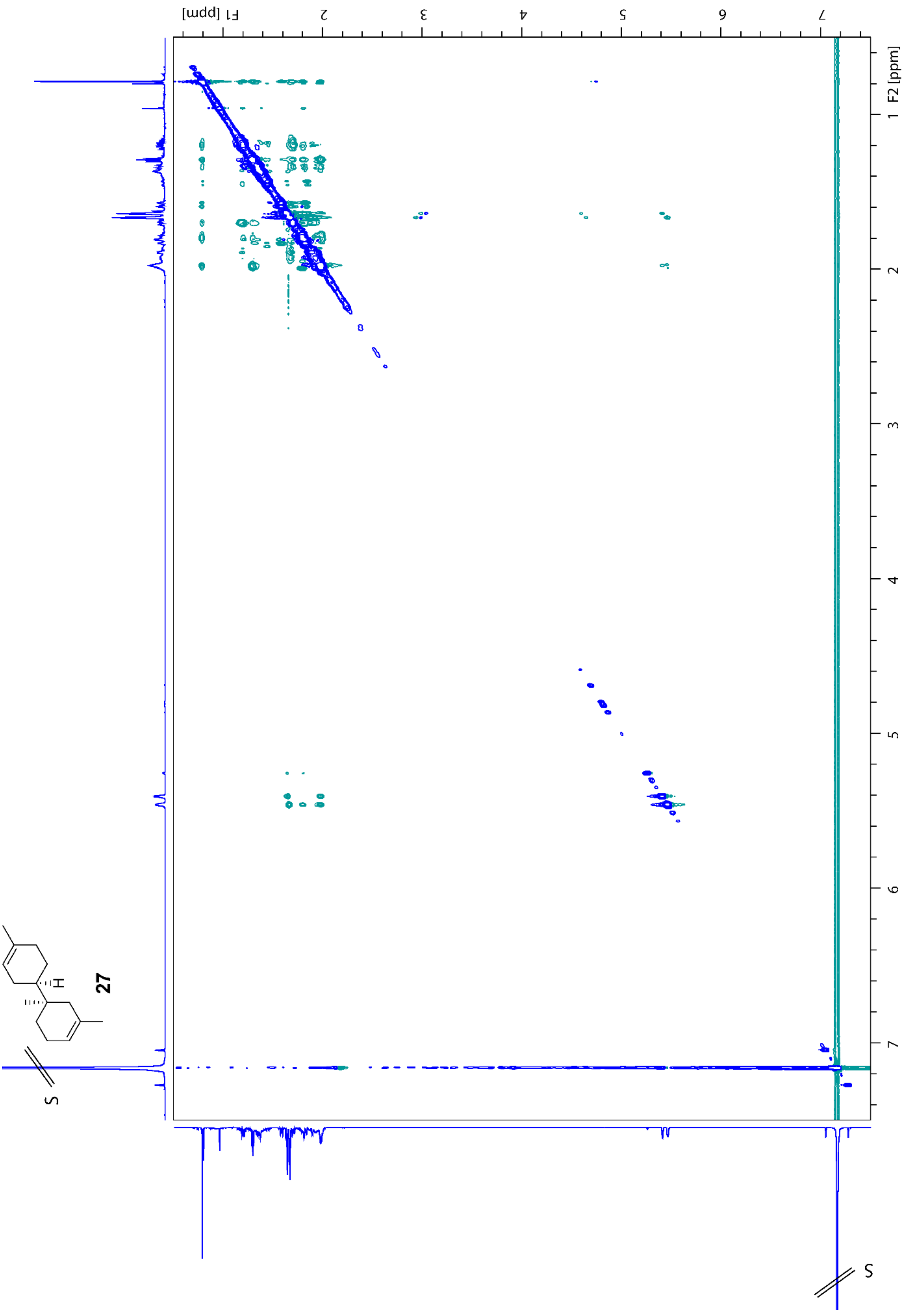

Figure S59. NOESY spectrum (C_6D_6) of **27**. S indicates solvent peaks.

Table S6. NMR data of **28** recorded in C_6D_6.

C[a]		^1H[b]	^{13}C[b]
1	CH_2	2.01 – 1.96 (m, 1H)	27.1
		1.86 – 1.80 (m, 1H)	
2	CH	5.47 (br s, 1H)	121.9
3	C_q	–	133.6
4	CH_2	1.99 – 1.93 (m ,1H)	31.8
		1.93 – 1.87 (m, 1H)	
5	CH_2	1.83 – 1.77 (m, 1H)	24.2
		1.25 – 1.18 (m, 1H)	
6	CH	1.39 (dddd, *J* = 14.0, 11.7, 4.9, 2.4 Hz, 1H)	43.9
7	C_q	–	36.6
8	CH_2	1.48 – 1.41 (m, 1H)	31.6
		1.22 – 1.17 (m, 1H)	
9	CH_2	1.60 – 1.55 (m, 2H)	20.0
10	CH_2	1.82 – 1.77 (m, 1H)	30.4
		1.76 – 1.71 (m,1H)	
11	C_q	–	132.2
12	CH	5.26 (s, 1H)	130.7
13	CH_3	1.62 (s, 3H)	24.3
14	CH_3	0.96 (s, 3H)	24.6
15	CH_3	1.66 (s, 3H)	23.7

[a] Carbon numbering as shown in Figure S52. [b] Chemical Shifts δ in ppm, coupling constants *J* in Hertz.

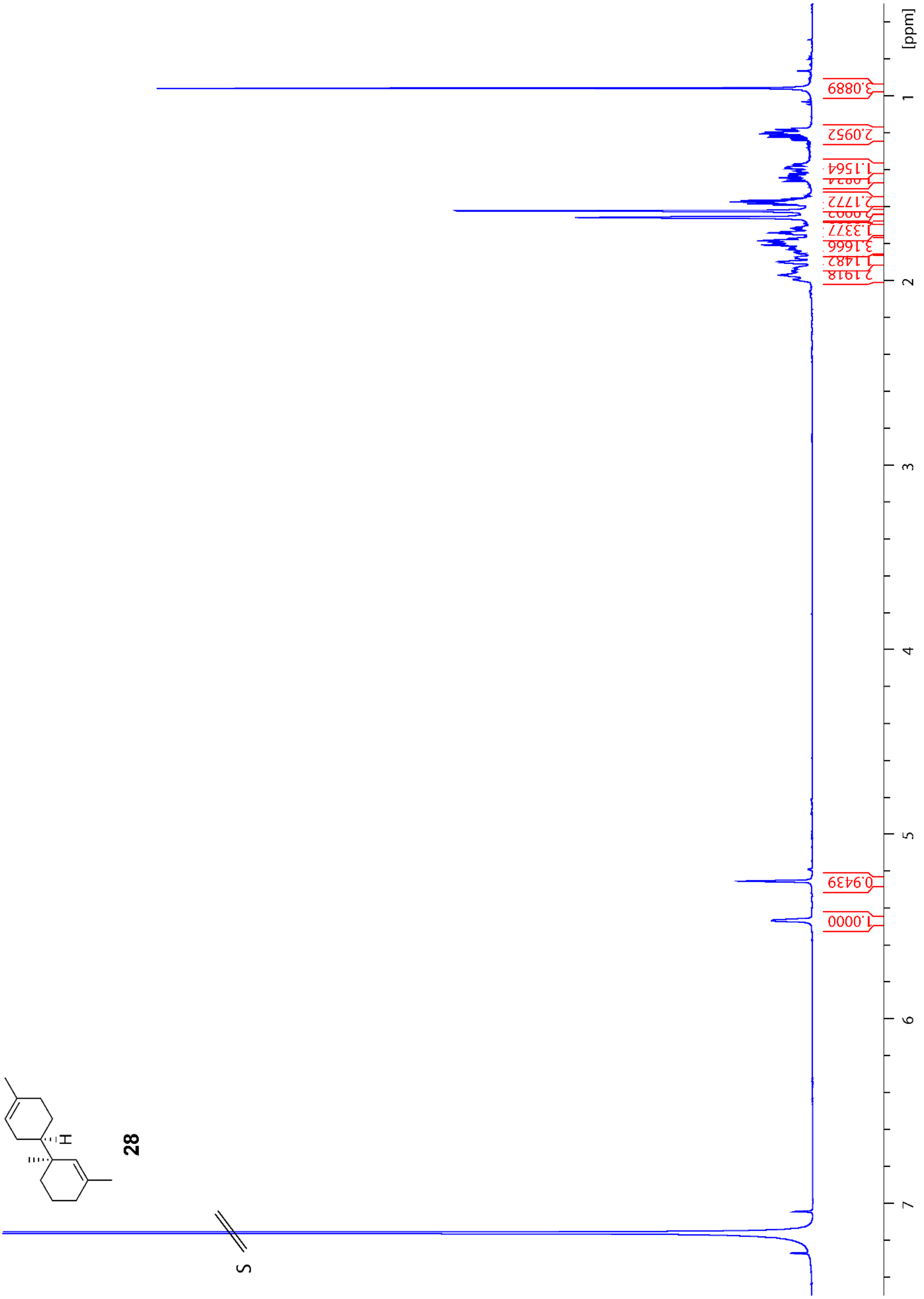

Figure S60. ^{1}H-NMR spectrum (C_6D_6, 700 MHz) of **28**. S indicates solvent peak.

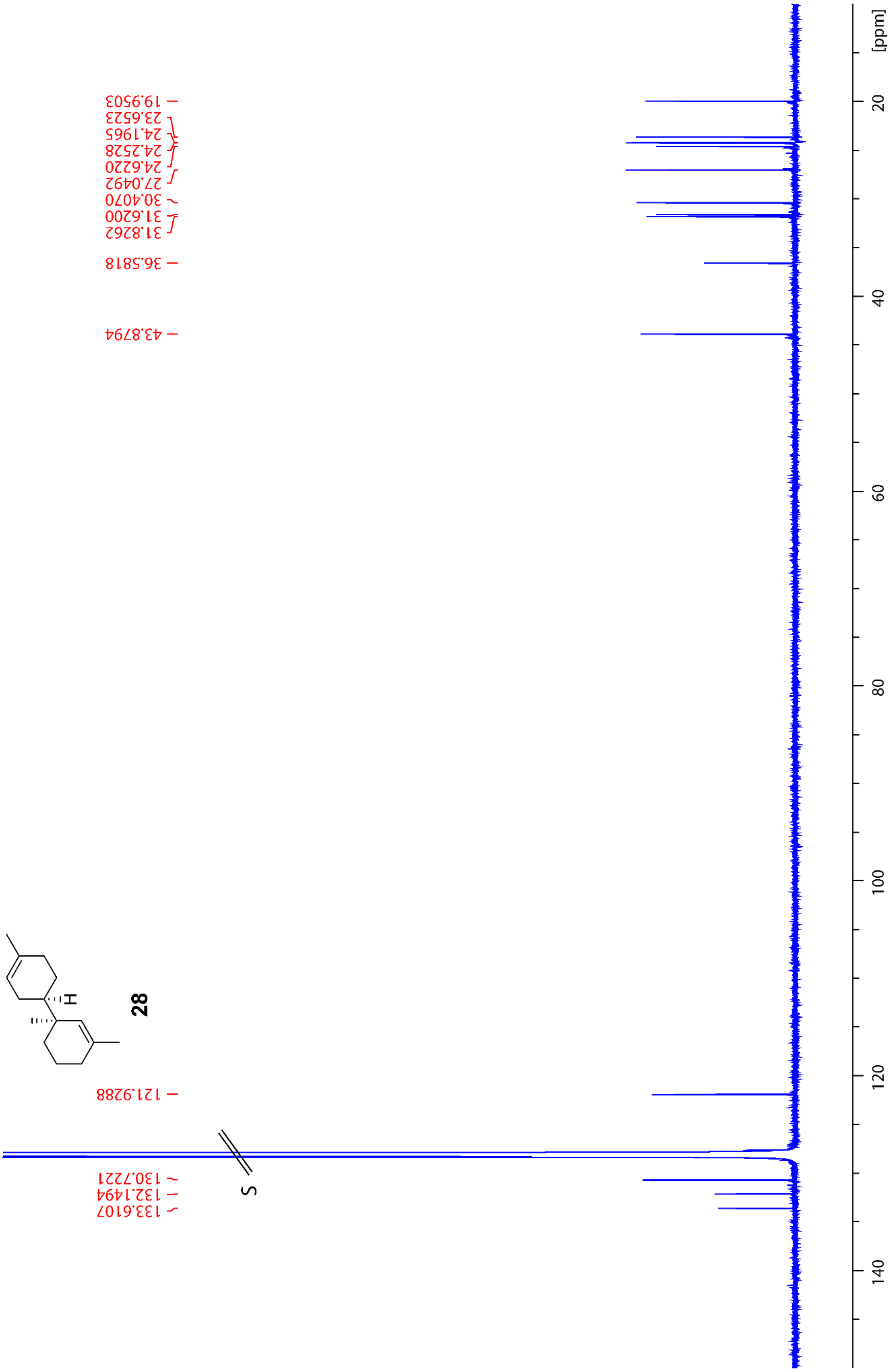

Figure S61. ^{13}C-NMR spectrum (C_6D_6, 175 MHz) of **28**. S indicates solvent peak.

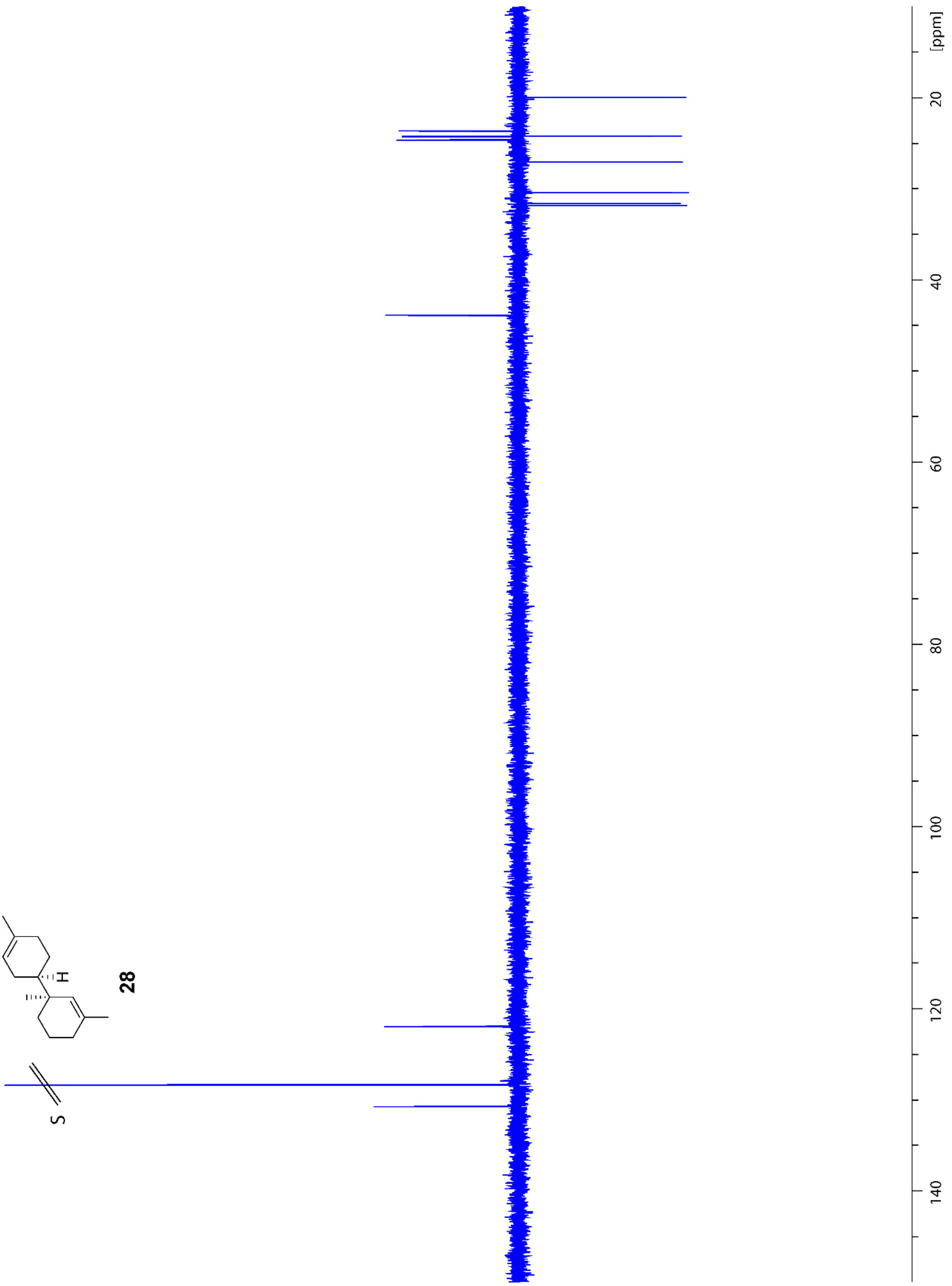

Figure S62. ^{13}C-DEPT135 spectrum (C_6D_6, 175 MHz) of **28**. S indicates solvent peak.

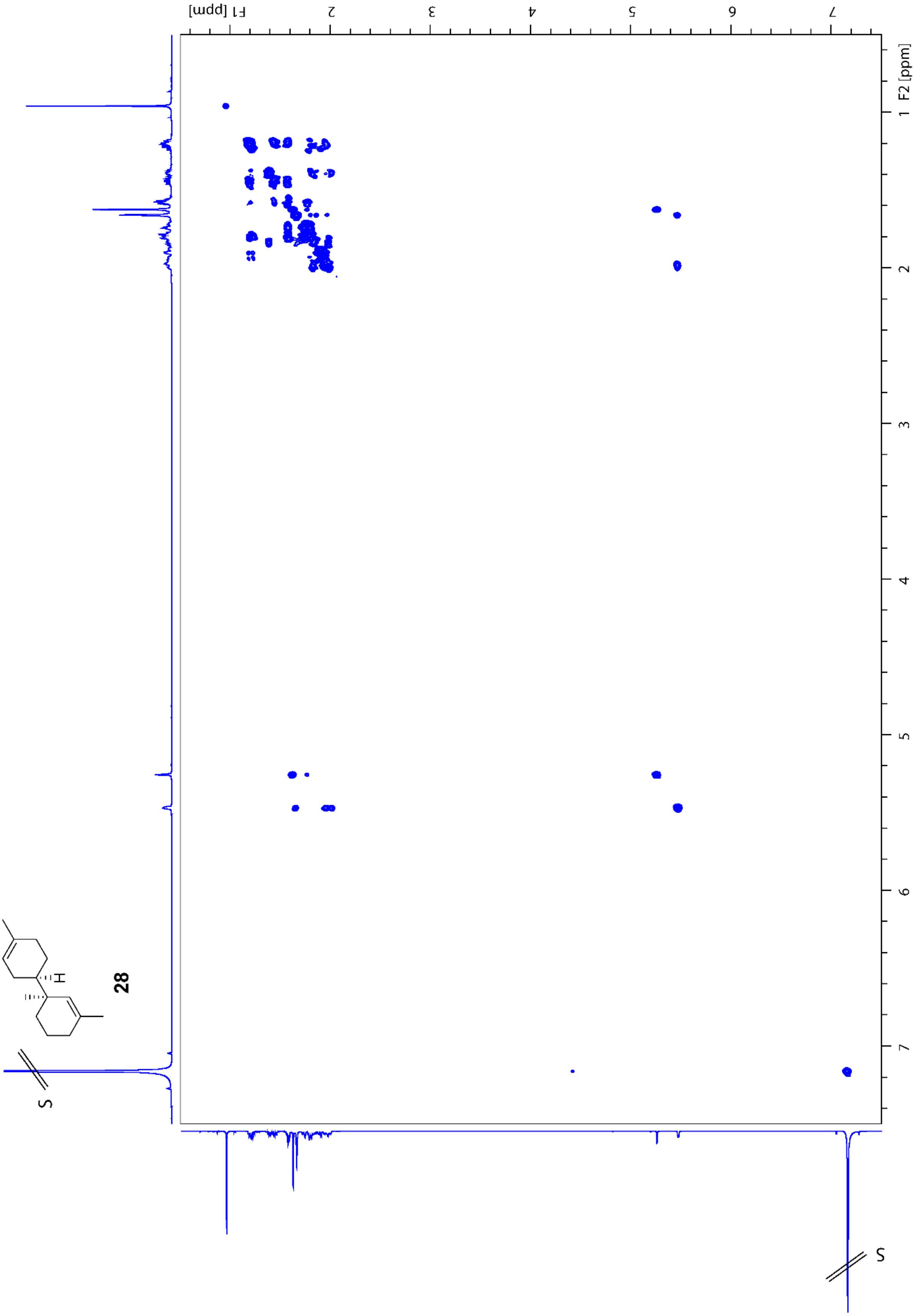

Figure S63. $^{1}H,^{1}H$-COSY spectrum (C_6D_6) of **28**. S indicates solvent peaks.

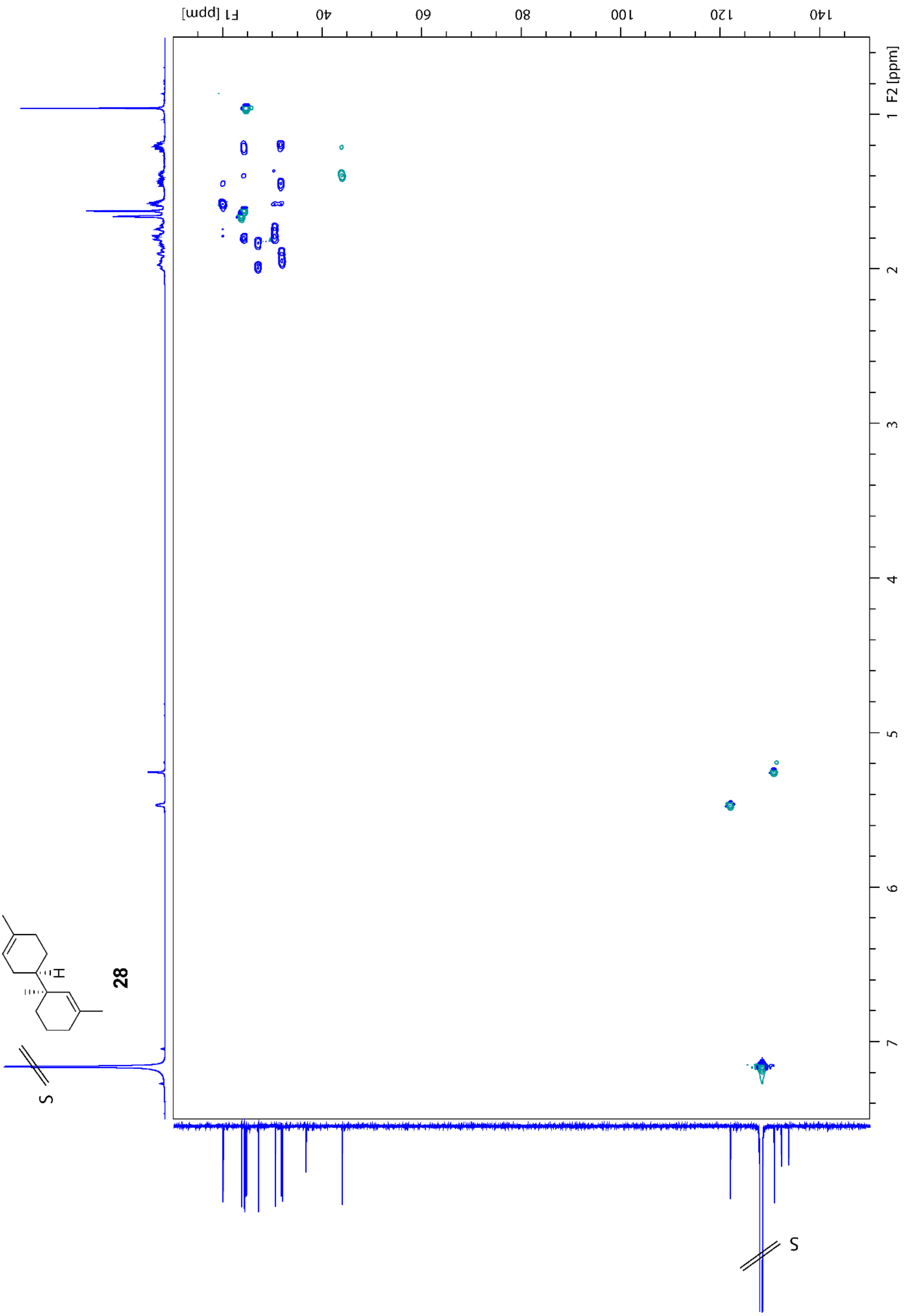

Figure S64. HSQC spectrum (C_6D_6) of **28**. S indicates solvent peaks.

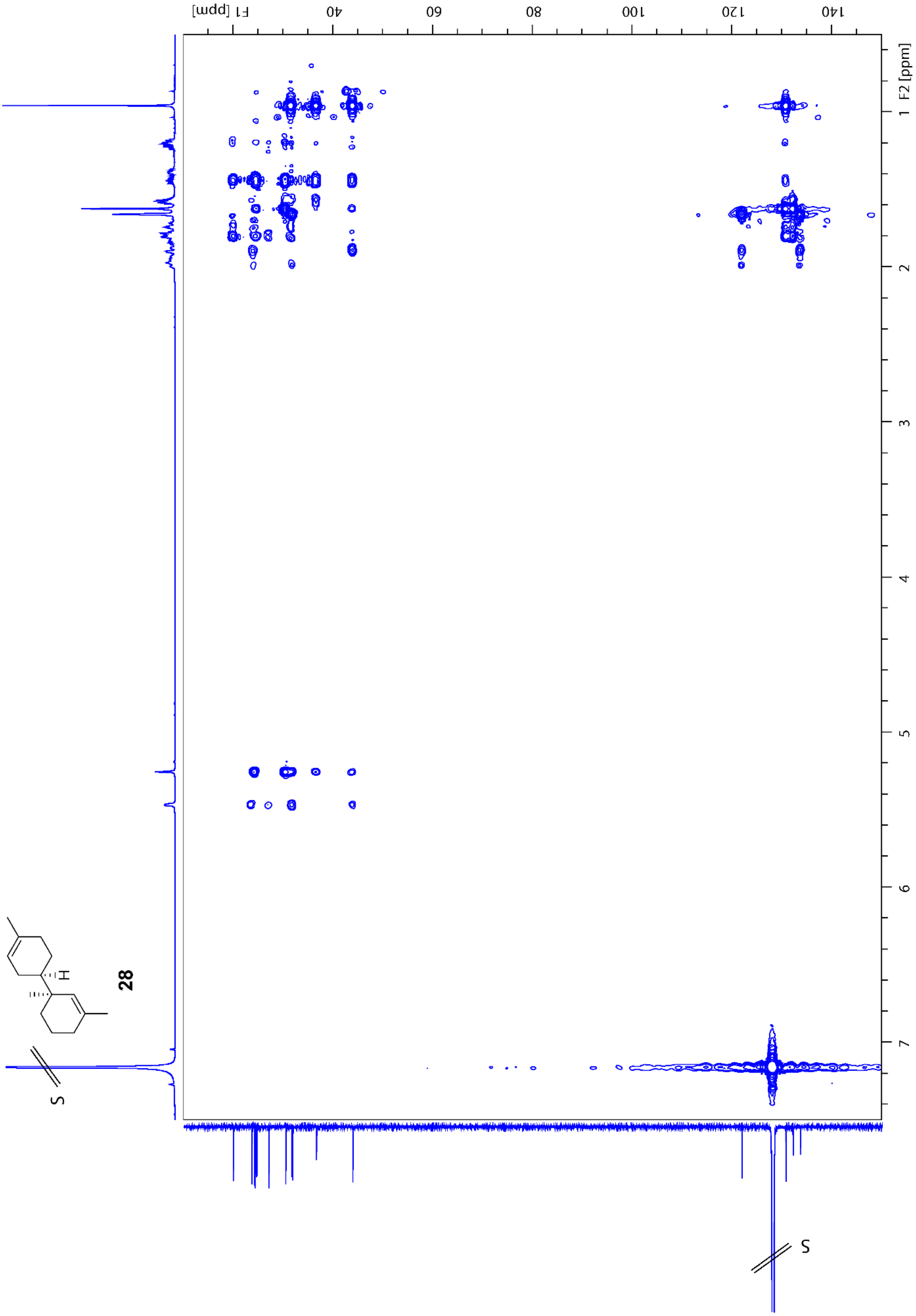

Figure S65. HMBC spectrum (C_6D_6) of **28**. S indicates solvent peaks.

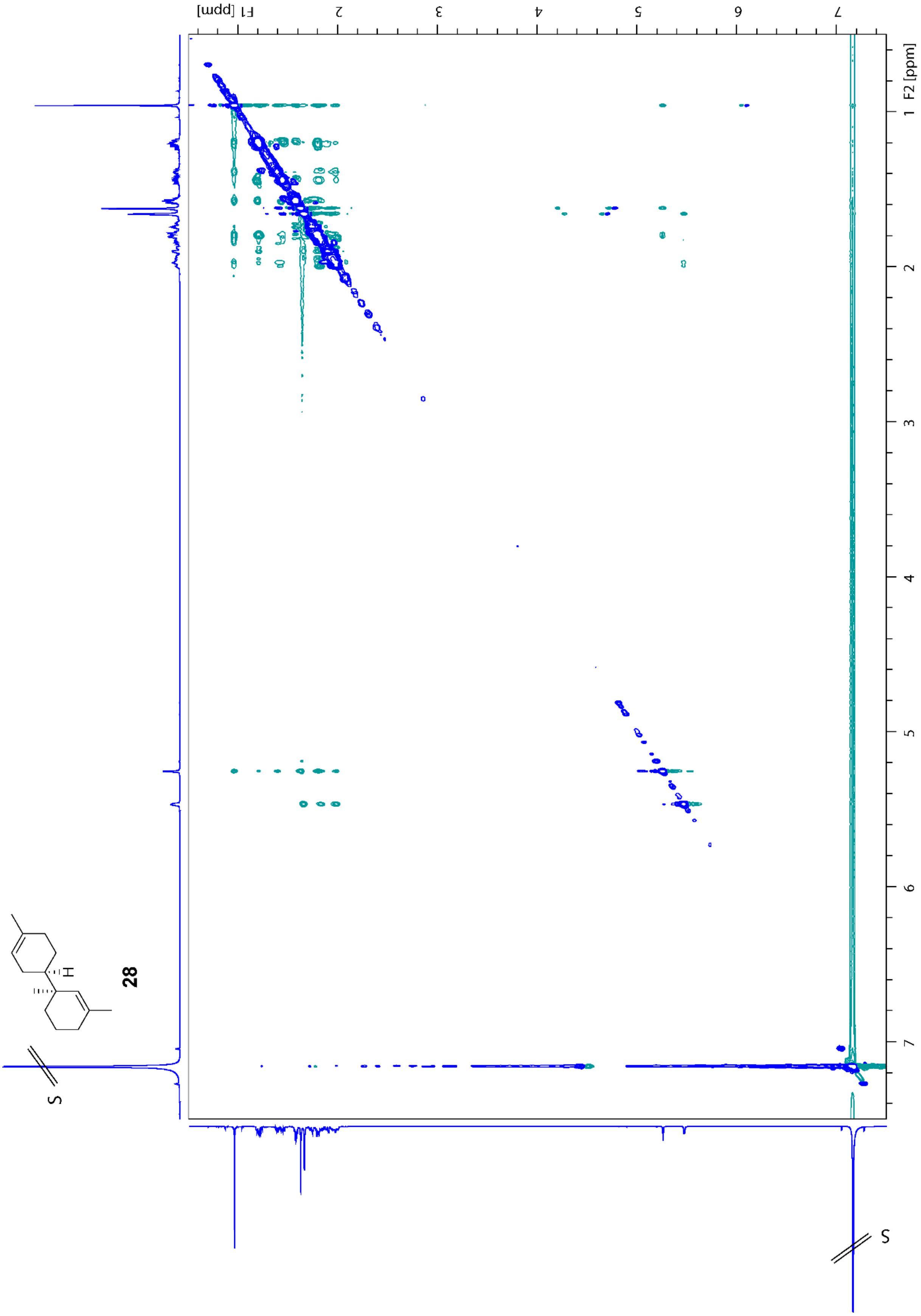

Figure S66. NOESY spectrum (C_6D_6) of **28**. S indicates solvent peaks.

Table S7. NMR data of **29** recorded in C_6D_6.

C[a]		^{1}H[b]	^{13}C[b]
1	CH	5.62 (dq, *J* = 9.9, 1.7 Hz, 1H)	135.2
2	CH	6.24 (dd, *J* = 10.0, 2.7 Hz, 1H)	130.2
3	C_q	–	143.9
4	CH_2	2.38 (dt, *J* = 14.7, 4.0 Hz, 1H)	30.8
		2.28 – 2.22 (m, 1H)	
5	CH_2	1.56 – 1.51 (m, 1H)	24.8
		1.39 – 1.33 (m, 1H)	
6	CH	2.11 – 2.07 (m, 1H)	41.0
7	CH	1.42 – 1.38 (m, 1H)	37.3
8	CH_2	1.28 – 1.24 (m, 1H)	34.1
		1.08 – 1.04 (m, 1H)	
9	CH_2	1.45 – 1.41 (m, 1H)	26.0
		1.32 – 1.28 (m, 1H)	
10	CH_2	1.95 – 1.92 (m, 2H)	38.4
11	C_q	–	145.9
12	CH_2	4.83 – 4.80 (m, 2H)	110.4
13	CH_3	1.66 (s, 3H)	22.5
14	CH_3	0.78 (d, *J* = 6.9 Hz, 3H)	16.1
15	CH_2	4.86 (br s, 1H)	110.4
		4.83 – 4.81 (m, 1H)	

[a] Carbon numbering as shown in Figure S52. [b] Chemical Shifts δ in ppm, coupling constants *J* in Hertz.

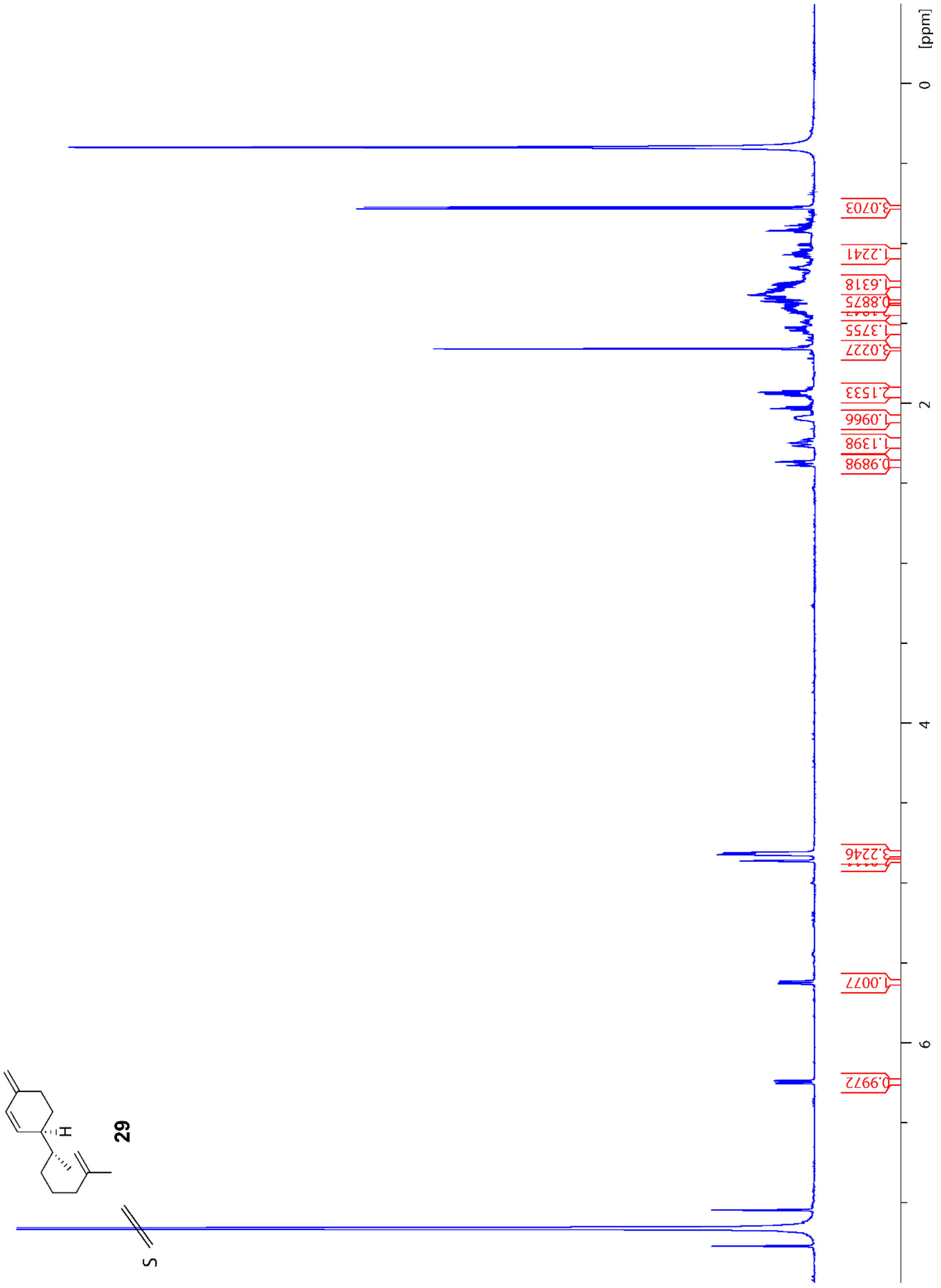

Figure S67. ^{1}H-NMR spectrum (C_6D_6, 700 MHz) of **29**. S indicates solvent peak.

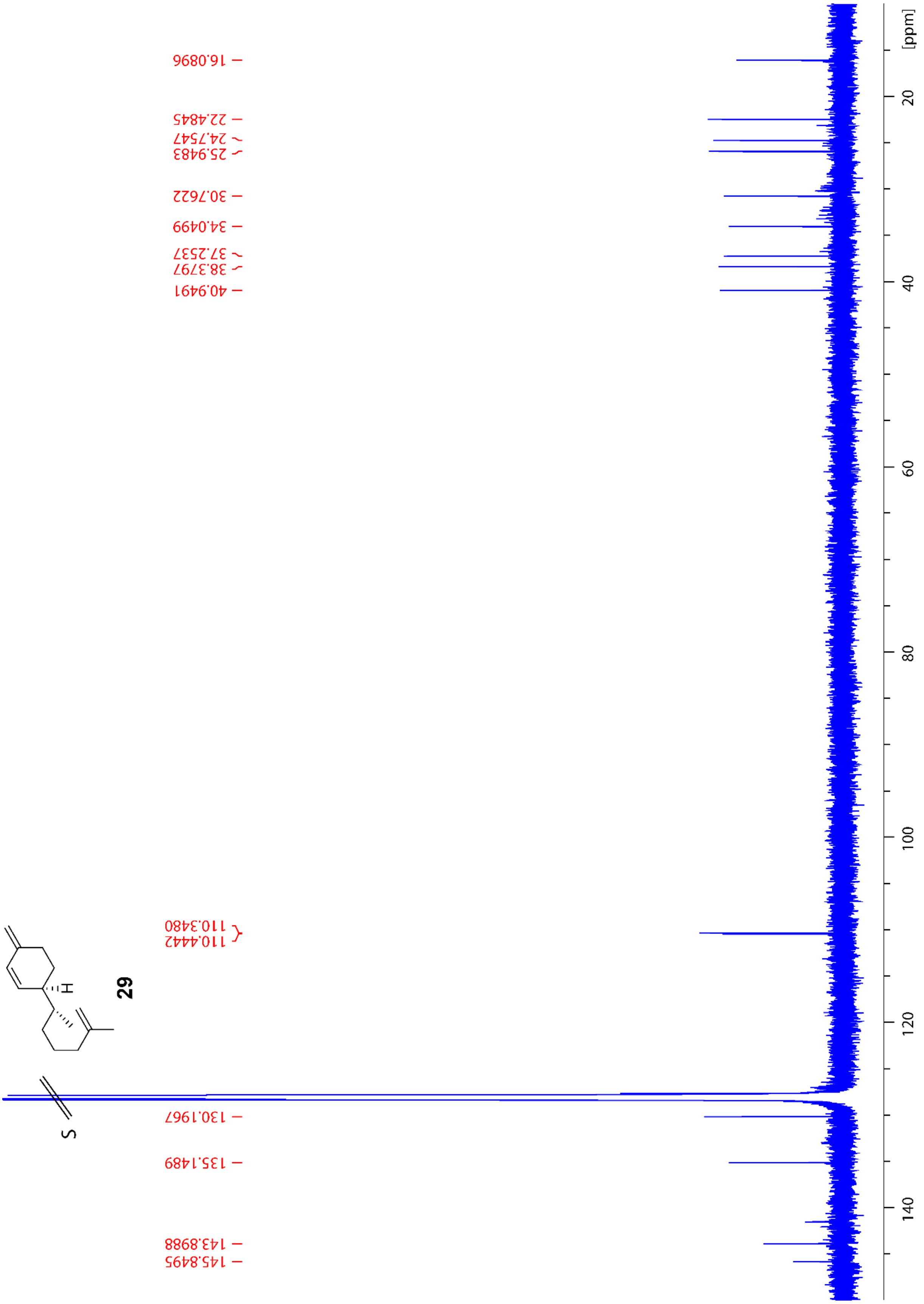

Figure S68. ^{13}C-NMR spectrum (C_6D_6, 175 MHz) of **29**. S indicates solvent peak.

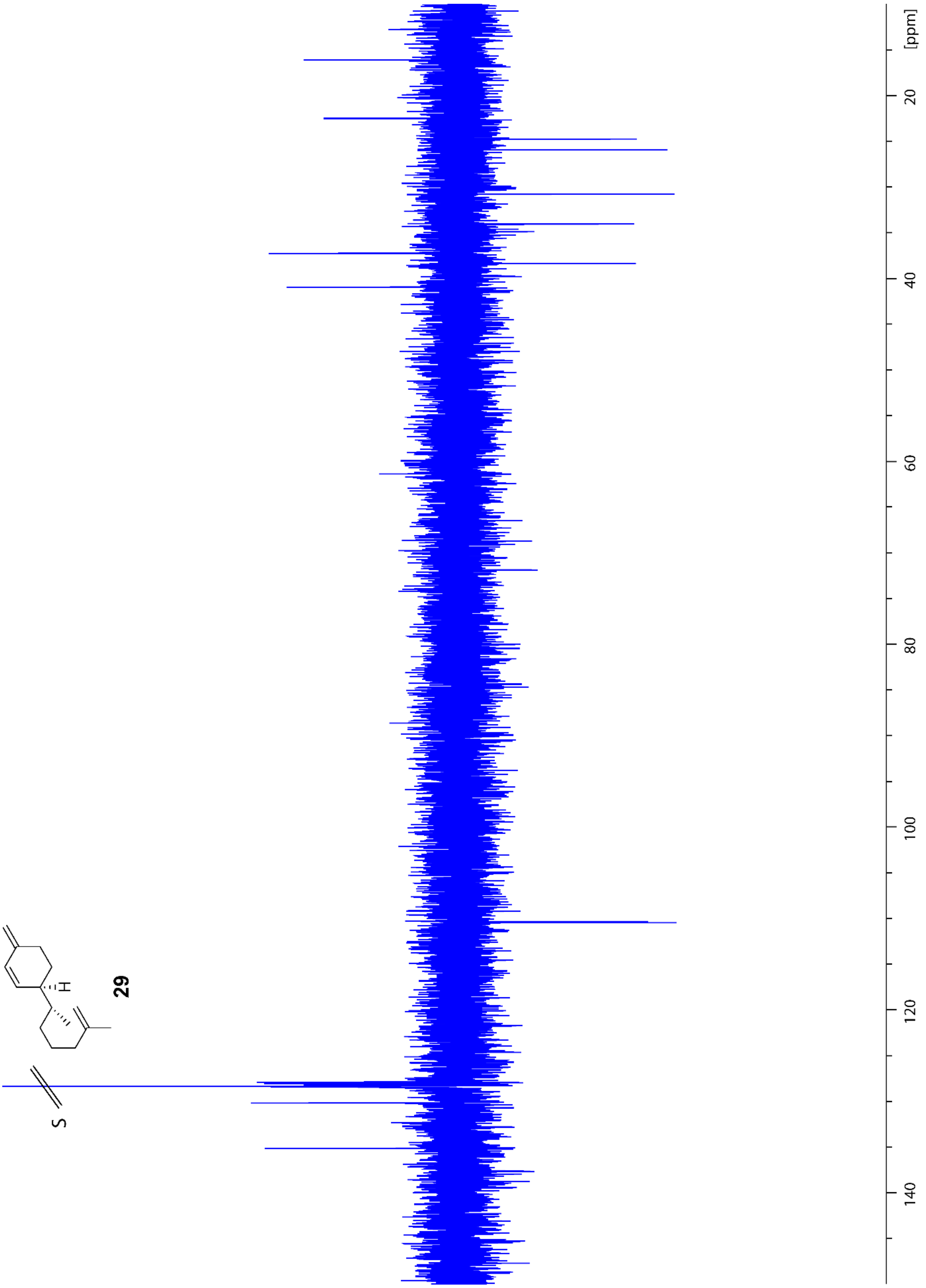

Figure S69. ^{13}C-DEPT135 spectrum (C_6D_6, 175 MHz) of **29**. S indicates solvent peak.

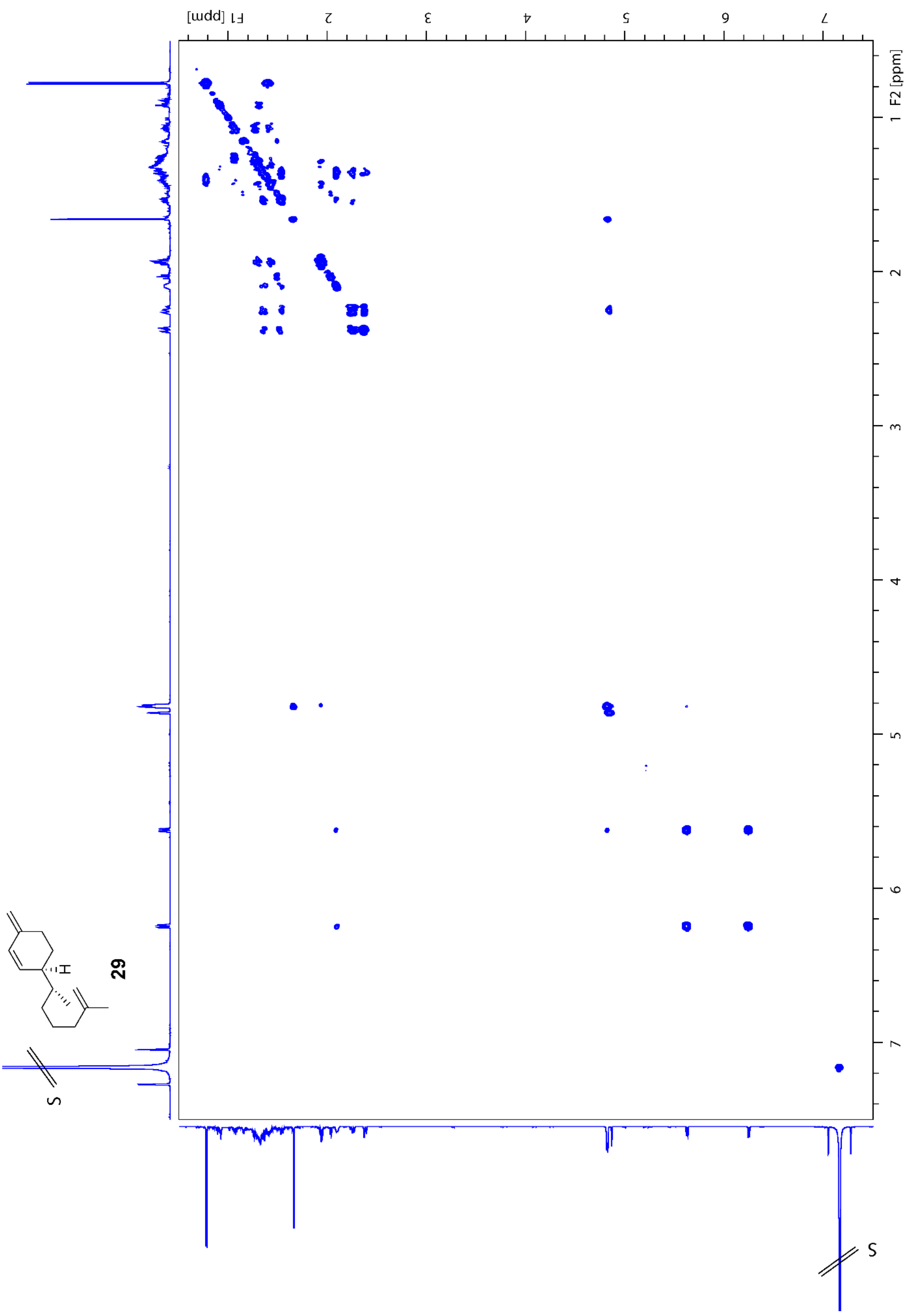

Figure S70. $^{1}H,^{1}H$-COSY spectrum (C_6D_6) of **29**. S indicates solvent peaks.

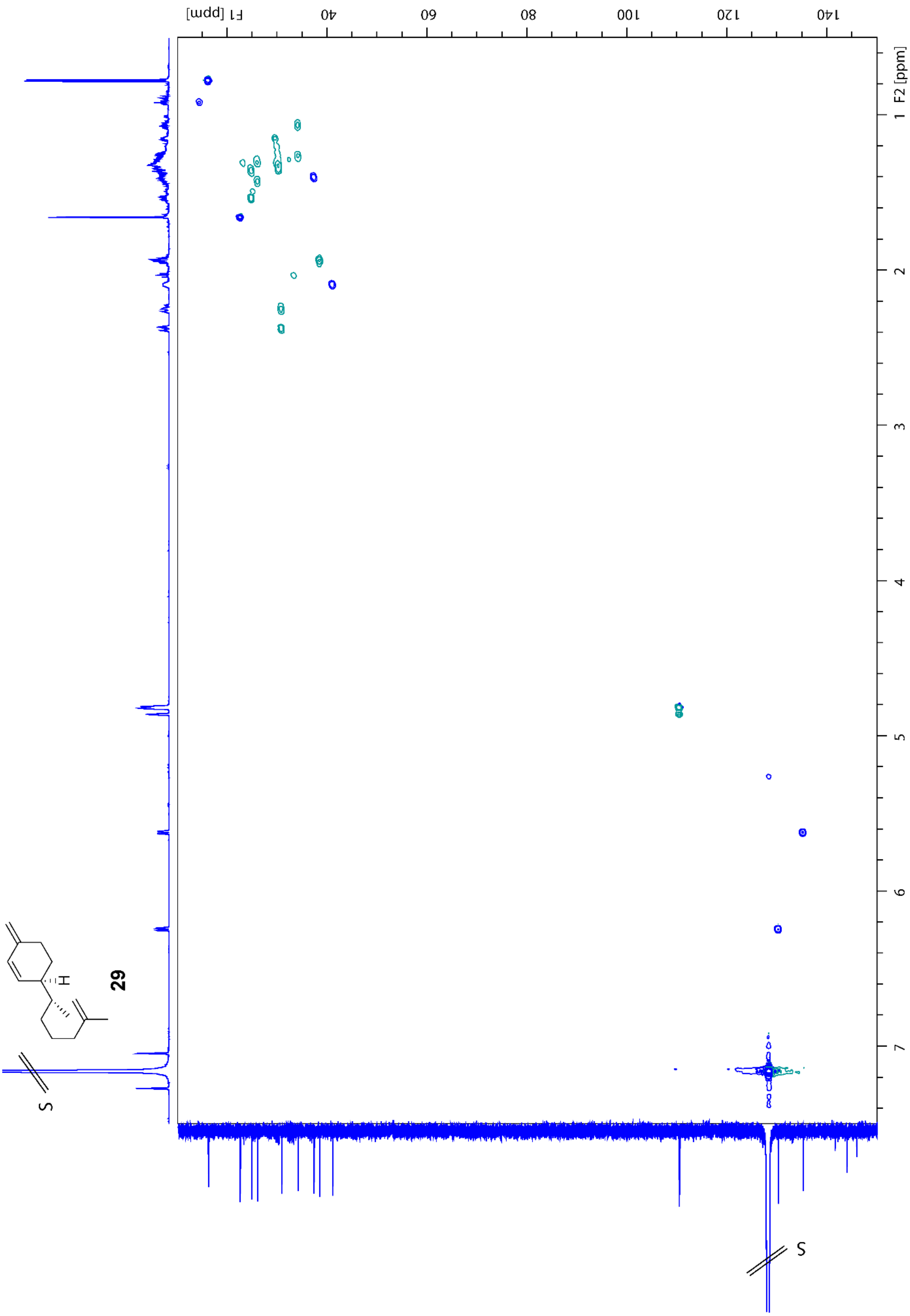

Figure S71. HSQC spectrum (C_6D_6) of **29**. S indicates solvent peaks.

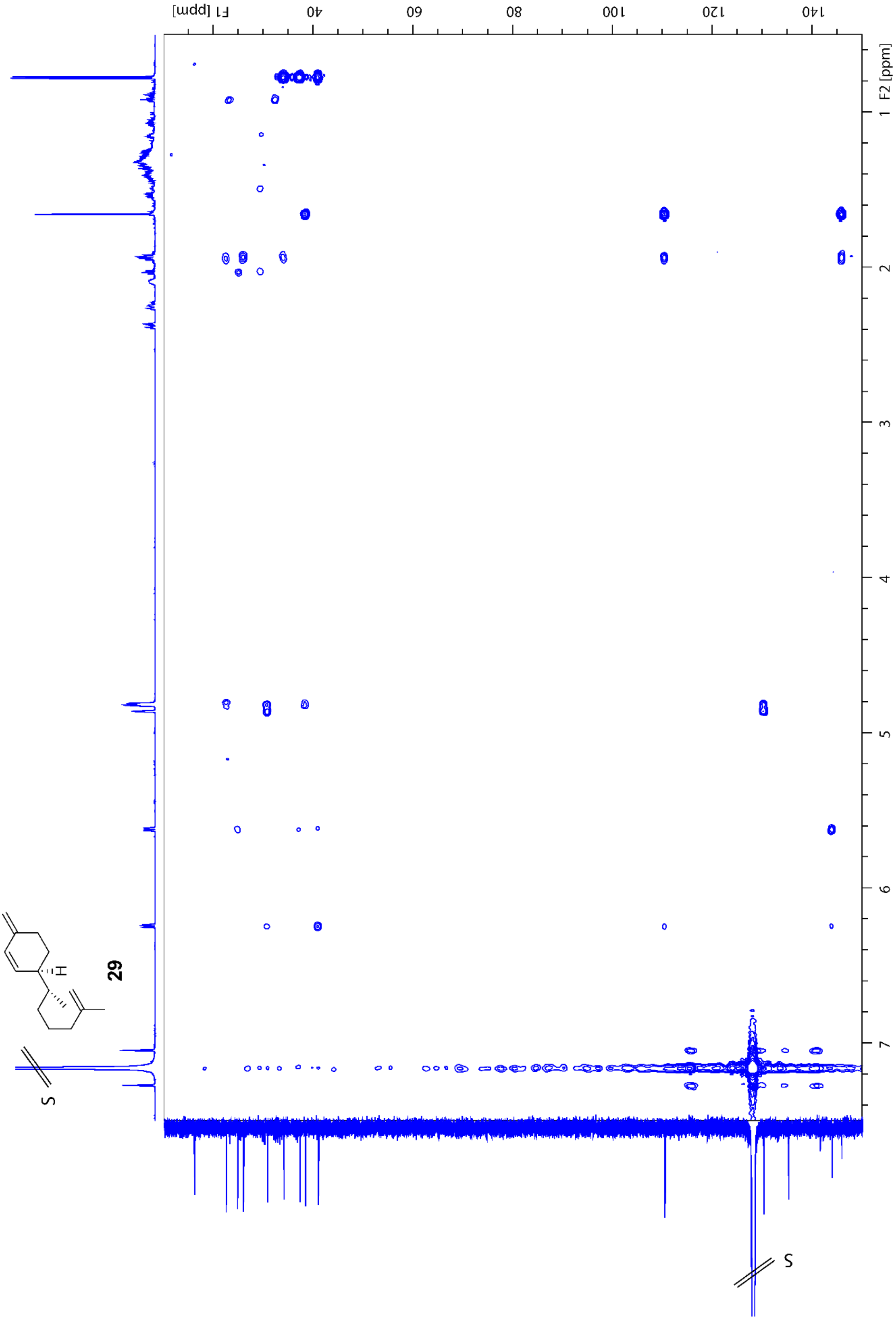

Figure S72. HMBC spectrum (C_6D_6) of **29**. S indicates solvent peaks.

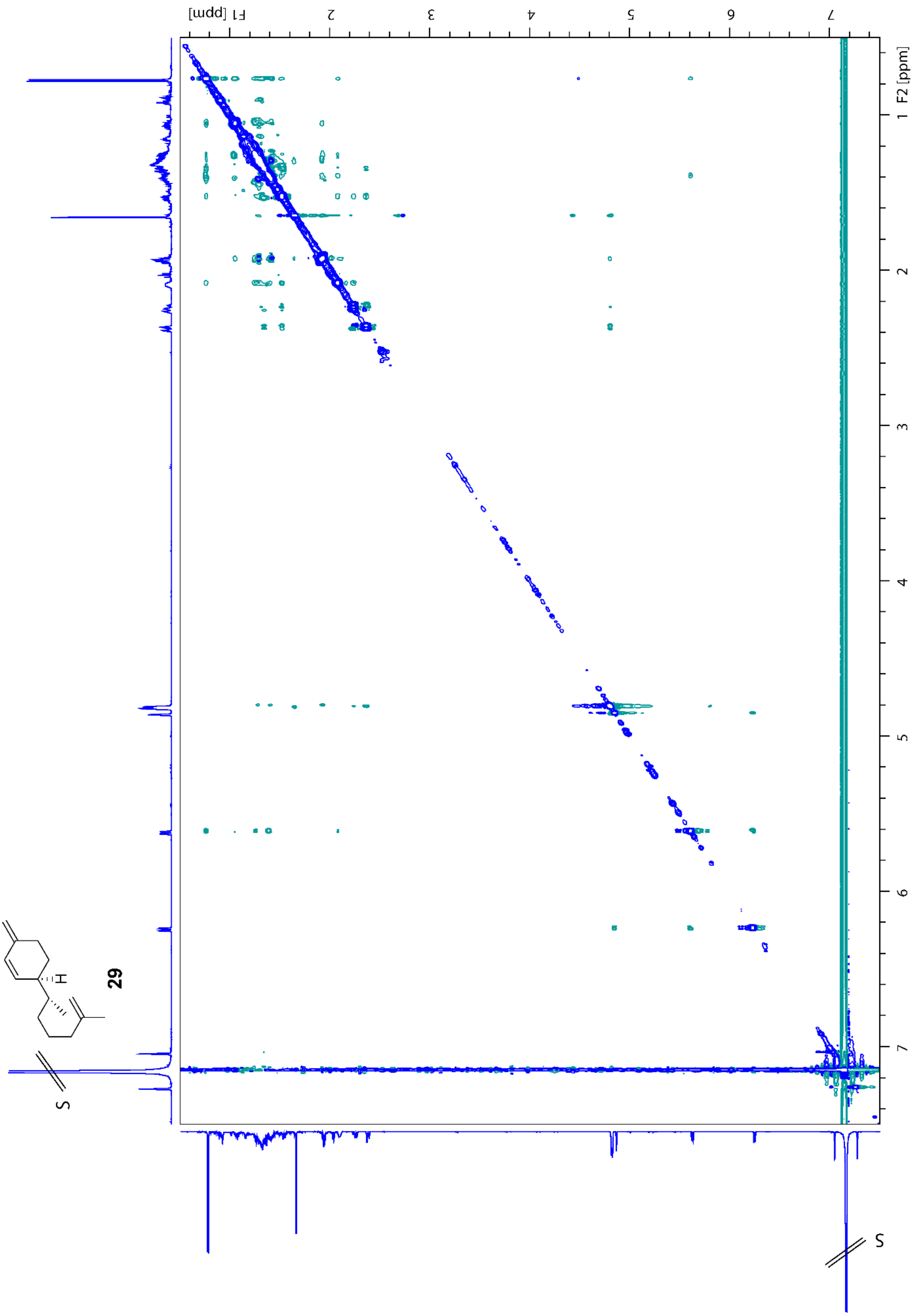

Figure S73. NOESY spectrum (C_6D_6) of **29**. S indicates solvent peaks.

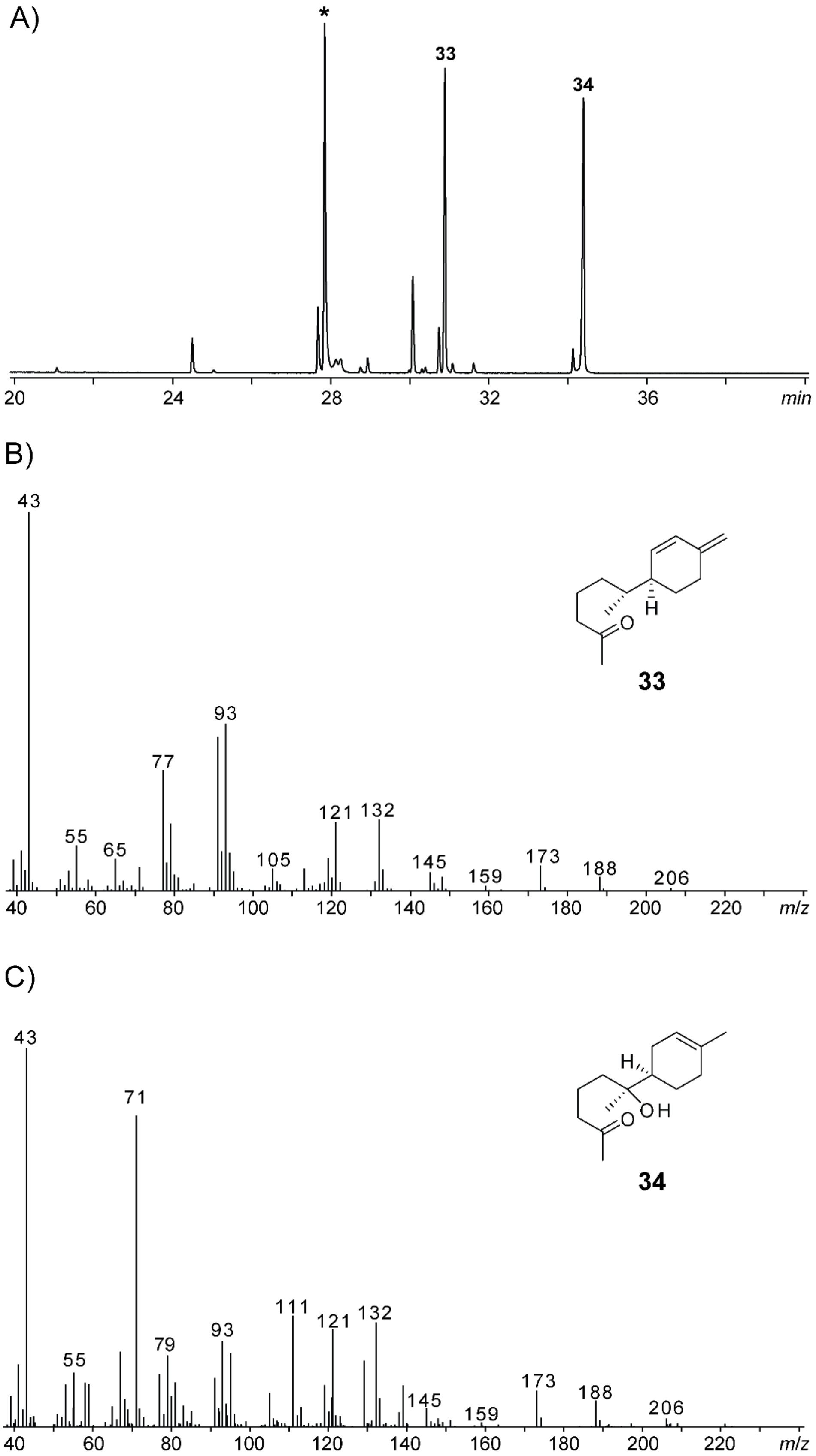

Figure S74. Incubation of DcS with FPP analogue **16**. A) Total ion chromatogram of the crude mixture. EI-MS spectra of compounds B) **33**, C) **34**. The asterisk indicates the loss of H_2O compound of **34** during the thermal GC/MS progress.

Isolation of compounds 33 and 34

DcS protein solution (90 mL) was obtained from 8 L *E.coli* BL21 culture. The protein solution, binding buffer (100 mL) and incubation buffer (200 mL) were mixed, followed by adding **28** (100 mg in 20 mL 25 mM NH_4HCO_3) dropwise. The enzymatic solution was incubated at 28 °C overnight and extracted with pentane (3 x 100 mL). The combined organic layers were concentrated under reduced pressure. Compound **33** was obtained via preparative TLC (cyclohexane/ethyl acetate, 5:1), compound **34** was purified via silica gel chromatography (diethyl ether, 100%).

(*R*)-6-((*S*)-4-Methylenecyclohex-2-en-1-yl)heptan-2-one (33): Colorless oil. Yield: 2.1 mg, 0.01 mmol, 4%. TLC (cyclohexane/ethyl acetate, 5:1): R_f = 0.48. Optical rotation: $[\alpha]_D^{20}$ = +21.4 (*c* 0.21, CH_2Cl_2). GC (HP5-MS): *I* = 1633. HRMS (QToF): *m*/*z* = 188.1564 (calc. for $[C_{14}H_{22}O - H_2O]^+$ = 188.1560). MS (EI, 70 eV): *m*/*z* (%) = 206 (1), 188 (4), 173 (8), 159 (2), 145 (6), 132 (20), 121 (19), 113 (6), 105 (6), 93 (45), 77 (33), 65 (8), 55 (12), 43 (100). (Figure S74B). IR (diamond ATR): $\tilde{\nu}$ = 3015 (w), 2931 (m), 2869 (m), 1716 (s), 1635 (w), 1595 (w), 1437 (m), 1419 (m), 1361 (m), 1226 (w), 1162 (m), 877 (m), 545 (w) cm^{-1}. NMR data are given in Table S8 and Figures S76 – S82.

(*S*)-6-Hydroxy-6-((*R*)-4-methylcyclohex-3-en-1-yl)heptan-2-one (34): Colorless oil. Yield: 1.2 mg, 2%. TLC (diethyl ether, 100%): R_f = 0.49. Optical rotation: $[\alpha]_D^{20}$ = +51.7 (*c* 0.12, CH_2Cl_2). GC (HP5-MS): *I* = 1790. HRMS (APCI): *m*/*z* = 225.1849 (calc. for $[C_{15}H_{24}O_2 + H]^+$ = 225.1850). MS (EI, 70 eV): *m*/*z* (%) = 206 (2), 188 (6), 173 (11), 145 (6), 139 (12), 132 (29), 121 (37), 111 (37), 105 (8), 93 (25), 79 (18), 71 (87), 67 (21), 55 (18), 43 (100). (Figure S74C). IR (diamond ATR): $\tilde{\nu}$ = 3468 (w), 2959 (m), 2923 (m), 2855 (m), 1710 (s), 1676 (w), 1438 (m), 1368 (m), 1260 (m), 1163 (m), 1104 (m), 1017 (m), 935 (w), 916 (w), 800 (m), 545 (m) cm^{-1}. NMR data are given in Table S9 and Figures S83 – S89.

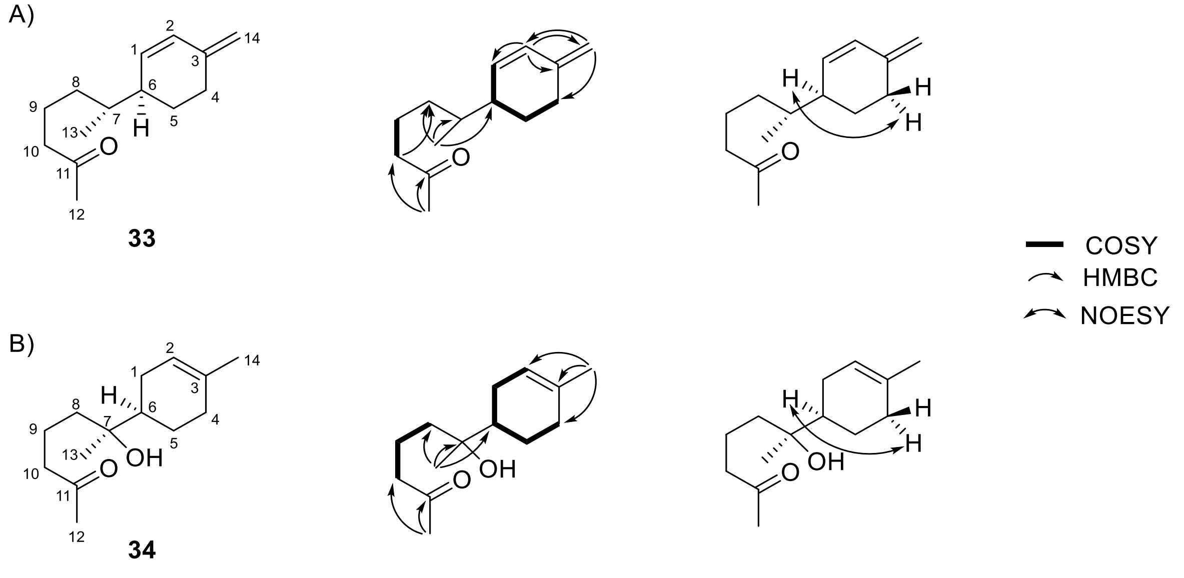

Figure S75. Structure elucidation of A) compound **33** and B) compound **34**. Bold lines indicate $^{1}H,^{1}H$-COSY correlations, single headed arrows HMBC correlations and double headed arrows NOESY correlation.

Table S8. NMR data of **33** recorded in C_6D_6.

C[a]		^{1}H[b]	^{13}C[b]
1	CH	5.60 (ddq, *J* = 10.0, 2.2, 1.0 Hz, 1H)	134.9
2	CH	6.24 (ddd, *J* = 9.3, 2.8, 0.9 Hz, 1H)	130.3
3	C_q	–	143.9
4	CH_2	2.39 – 2.35 (m, 1H)	30.7
		2.26 – 2.22 (m, 1H)	
5	CH_2	1.54 – 1.49 (m, 1H)	24.8
		1.36 – 1.30 (m, 1H)	
6	CH	2.08 – 2.03 (m, 1H)	40.8
7	CH	1.36 – 1.31 (m, 1H)	37.3
8	CH_2	1.17 – 1.11 (m, 1H)	33.9
		0.97 – 0.92 (m, 1H)	
9	CH_2	1.51 – 1.46 (m, 1H)	22.1
		1.38 – 1.33 (m, 1H)	
10	CH_2	1.89 (ddd, J = 7.6, 6.8, 2.7 Hz, 2H)	43.5
11	C_q	–	206.1
12	CH_3	1.66 (s, 3H)	29.4
13	CH_3	0.76 (d, *J* = 6.9 Hz, 3H)	16.1
14	CH_3	4.86 (td, *J* = 2.3, 1.0 Hz, 1H)	110.5
		4.82 (ddq, *J* = 3.4, 1.7, 1.0 Hz, 1H)	

[a] Carbon numbering as shown in Figure S75. [b] Chemical Shifts δ in ppm, coupling constants *J* in Hertz.

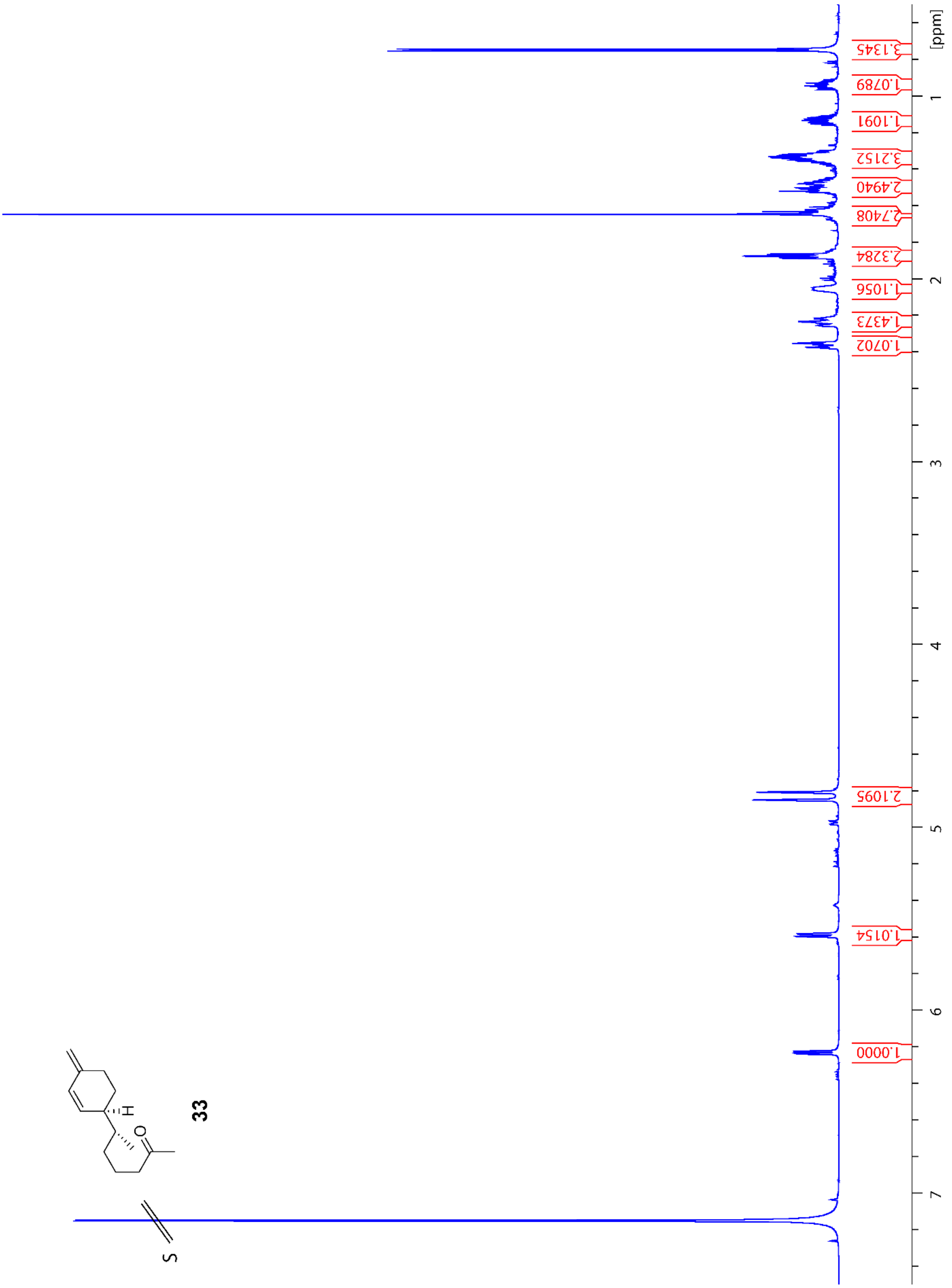

Figure S76. ^{1}H-NMR spectrum (C_6D_6, 700 MHz) of **33**. S indicates solvent peak.

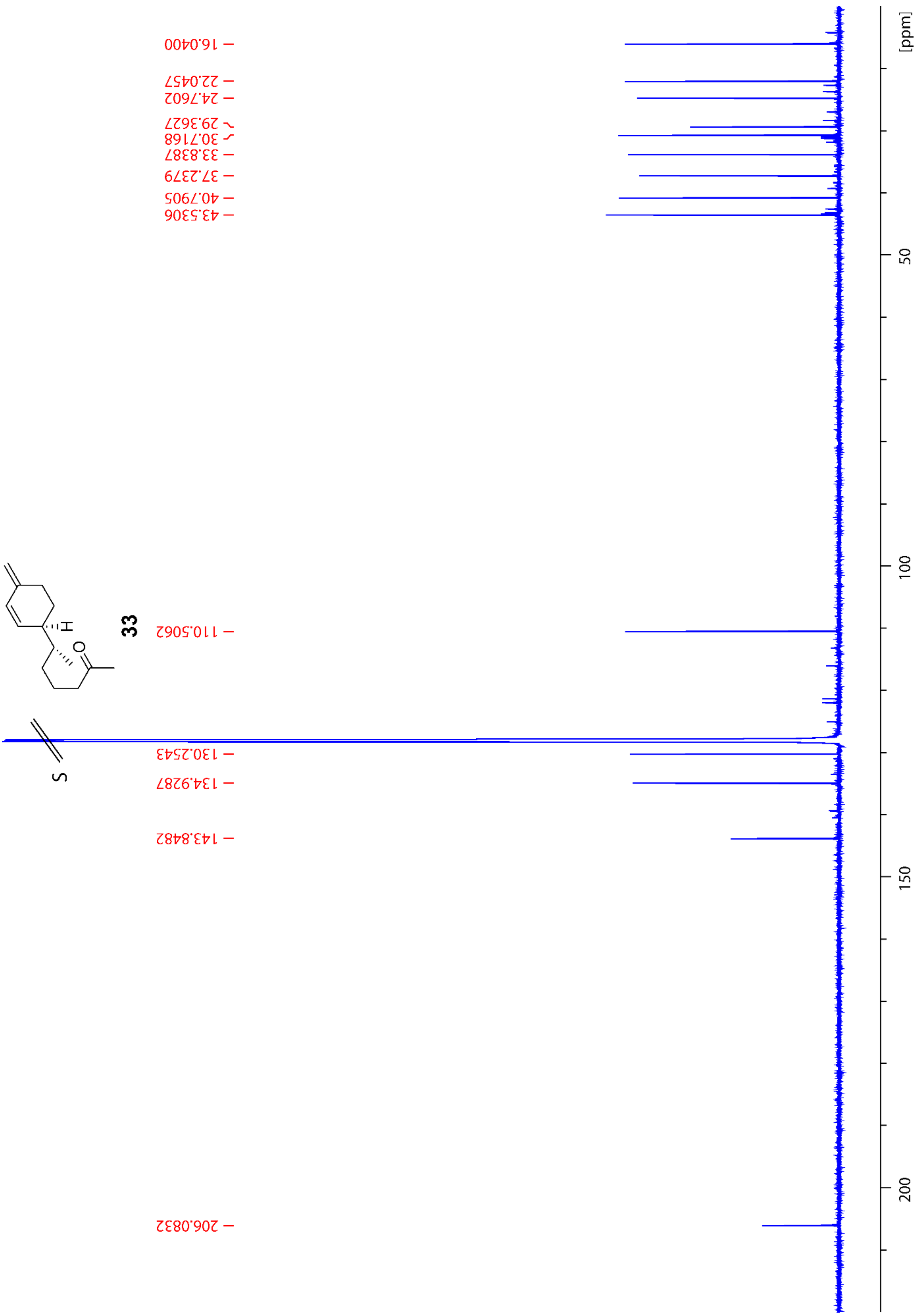

Figure S77. ^{13}C-NMR spectrum (C_6D_6, 175 MHz) of **33**. S indicates solvent peak.

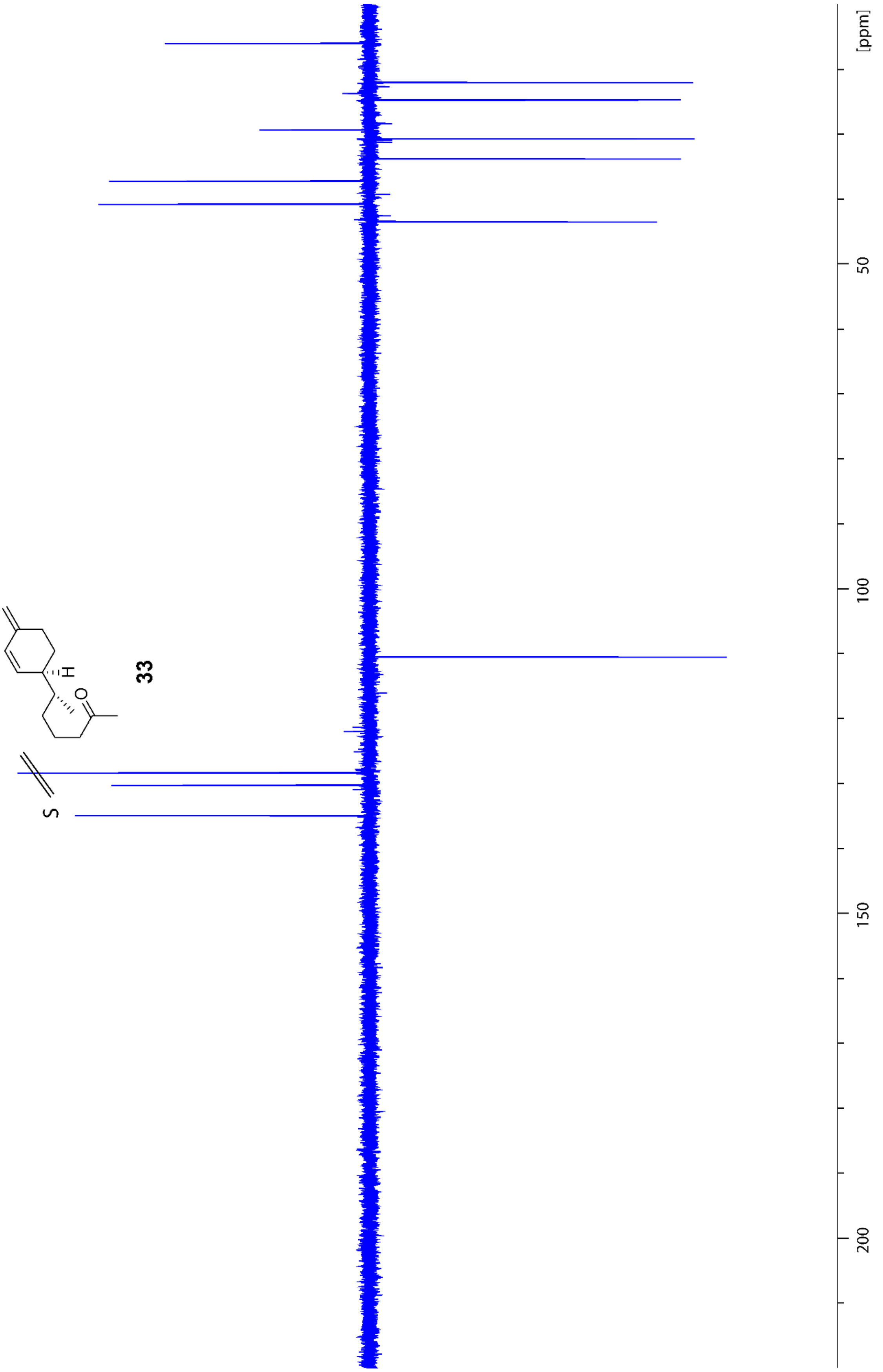

Figure S78. ^{13}C-DEPT135 spectrum (C_6D_6, 175 MHz) of **33**. S indicates solvent peak.

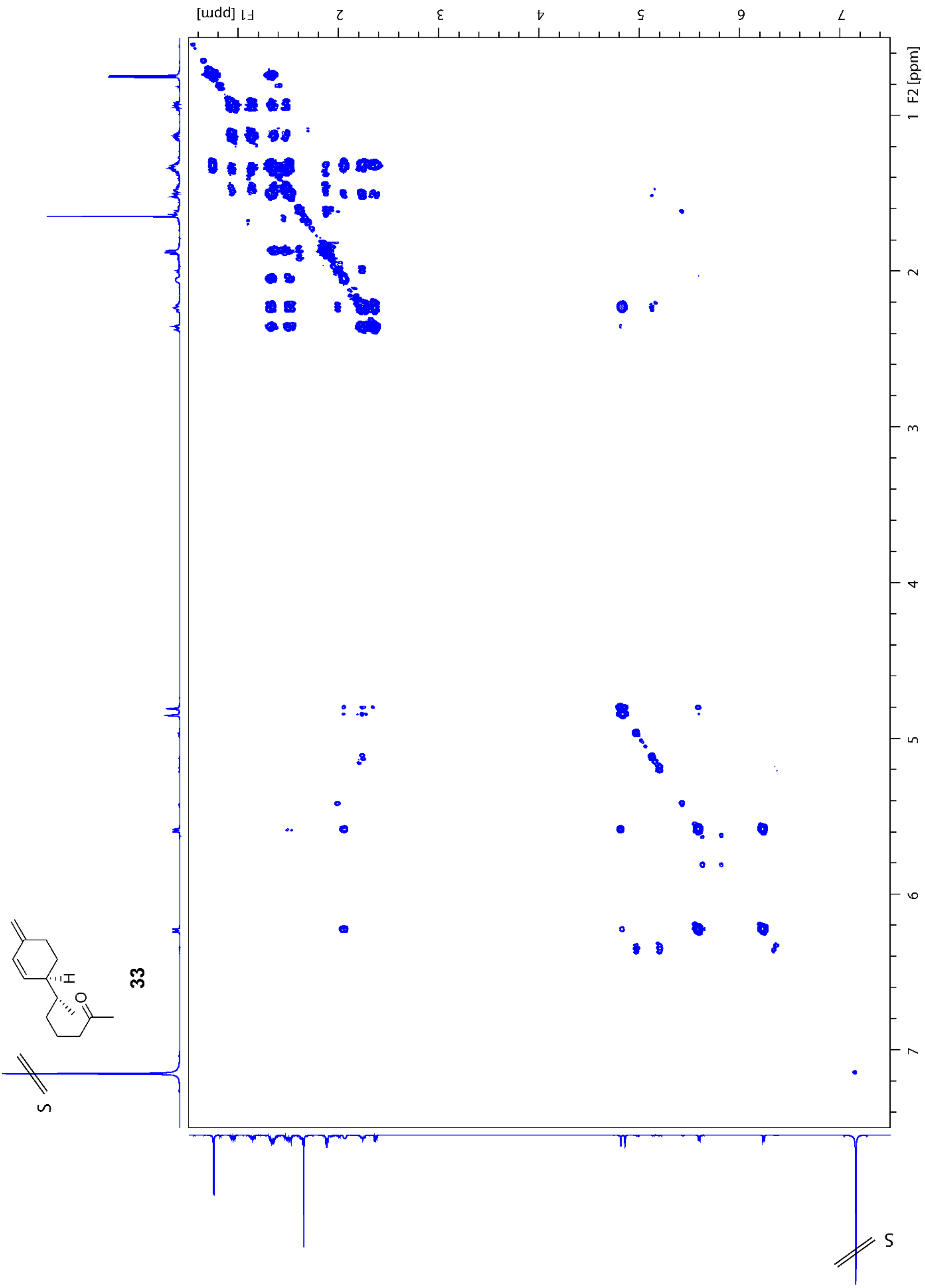

Figure S79. $^{1}H,^{1}H$-COSY spectrum (C_6D_6) of **33**. S indicates solvent peaks.

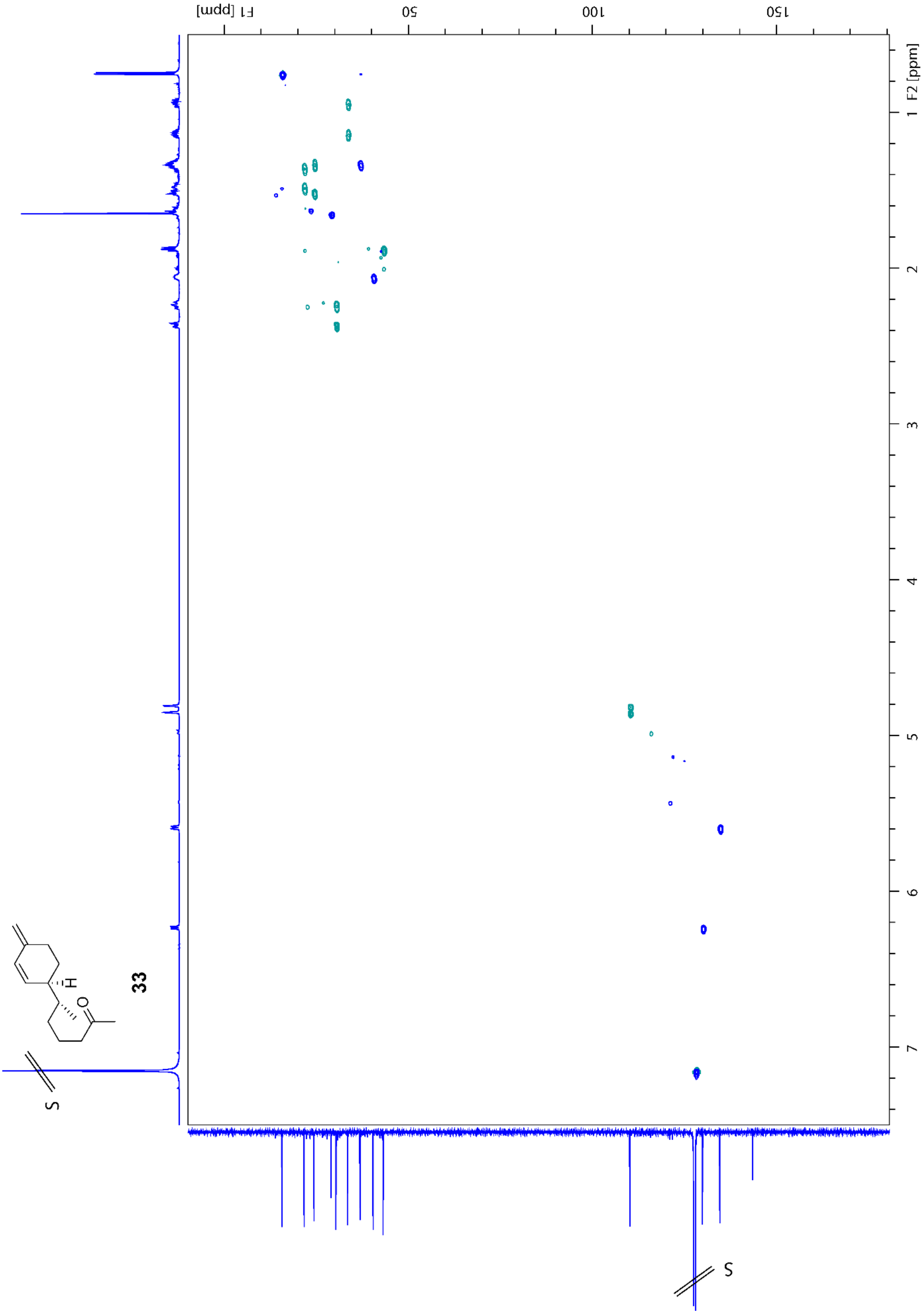

Figure S80. HSQC spectrum (C_6D_6) of **33**. S indicates solvent peaks.

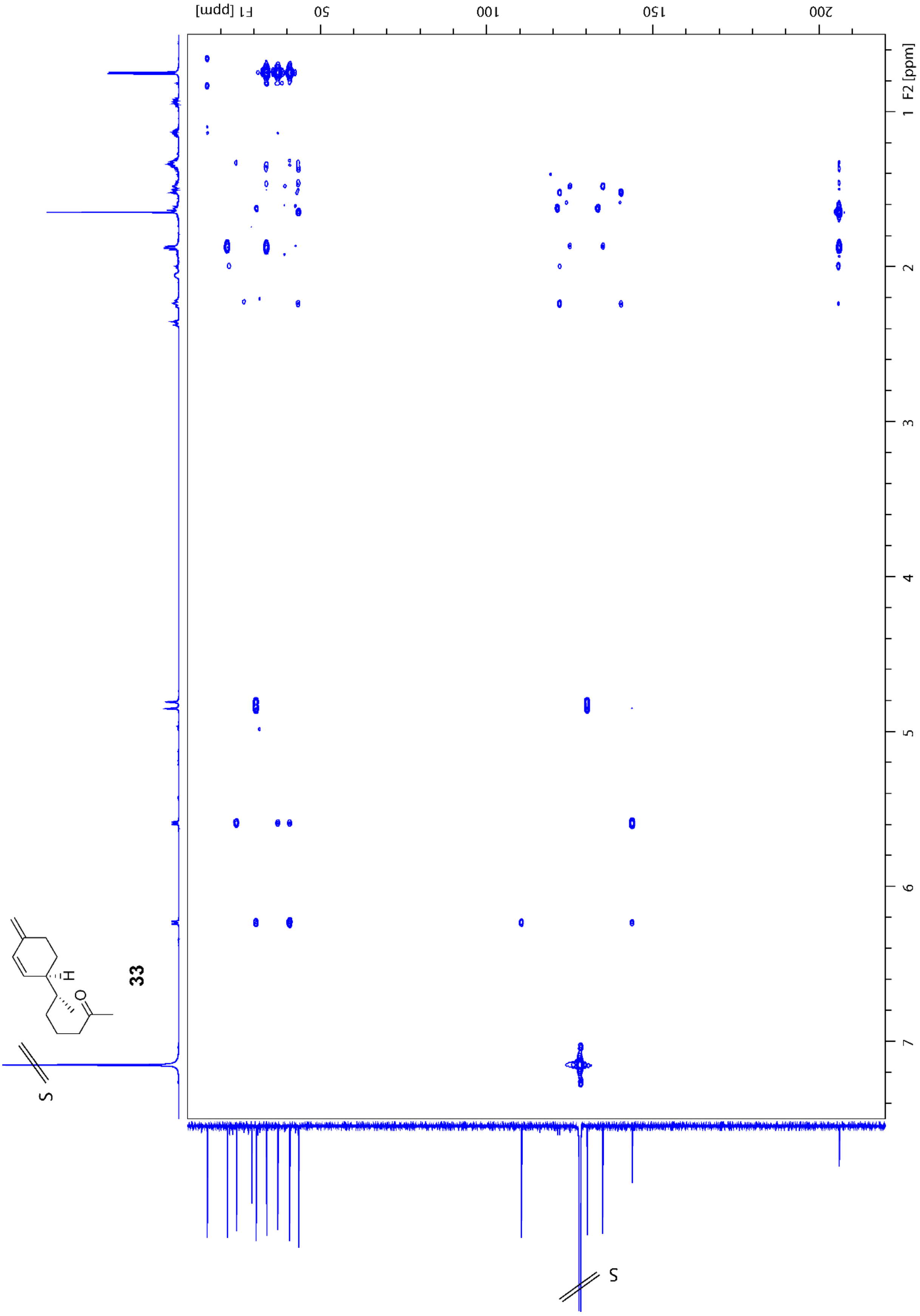

Figure S81. HMBC spectrum (C_6D_6) of **33**. S indicates solvent peaks.

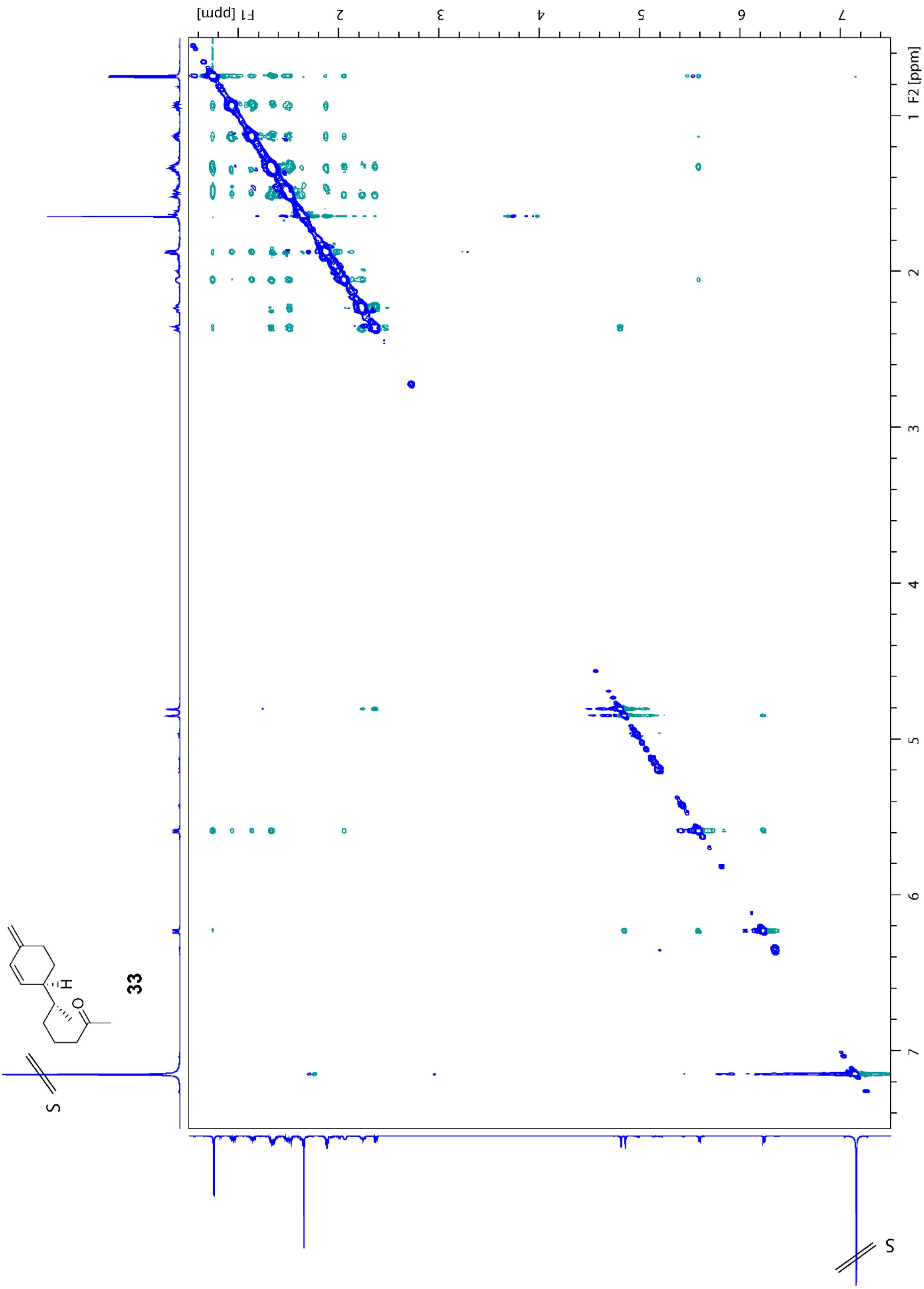

Figure S82. NOESY spectrum (C_6D_6) of **33**. S indicates solvent peaks.

Table S9. NMR data of **34** recorded in C_6D_6.

C[a]		^1H[b]	^{13}C[b]
1	CH_2	2.05 – 2.00 (m, 1H)	26.3
		1.85 – 1.82 (m, 1H)	
2	CH	5.46 – 5.44 (m, 1H)	121.5
3	C_q	–	133.5
4	CH_2	1.97 – 1.90 (m, 1H)	31.4
		1.89 – 1.83 (m, 1H)	
5	CH_2	1.69 (ddt, *J* = 12.2, 6.0, 2.2 Hz, 1H)	24.3
		1.18 (qd, *J* = 12.3, 5.4 Hz, 1H)	
6	CH	1.49 – 1.43 (m, 1H)	43.7
7	C_q	–	73.5
8	CH_2	1.27 – 1.23 (m, 2H)	39.1
9	CH_2	1.60 – 1.54 (m, 1H)	18.1
		1.54 – 1.49 (m, 1H)	
10	CH_2	1.93 – 1.89 (m, 2H)	43.7
11	C_q	–	206.4
12	CH_3	1.65 (s, 3H)	29.4
13	CH_3	0.95 (s, 3H)	24.2
14	CH_3	1.65 (s, 3H)	23.6

[a] Carbon numbering as shown in Figure S75. [b] Chemical Shifts δ in ppm, coupling constants *J* in Hertz.

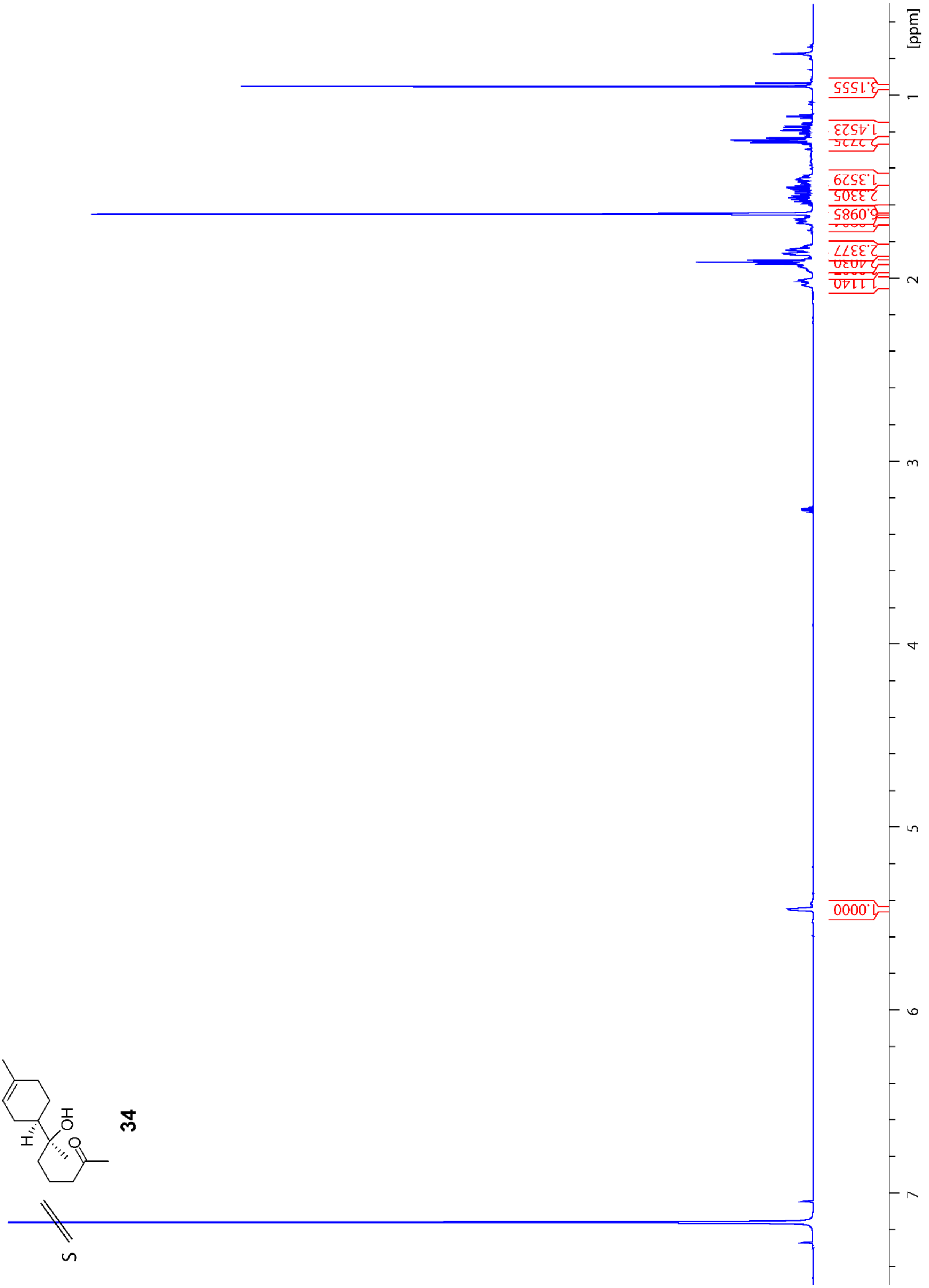

Figure S83. ^{1}H-NMR spectrum (C_6D_6, 700 MHz) of **34**. S indicates solvent peak.

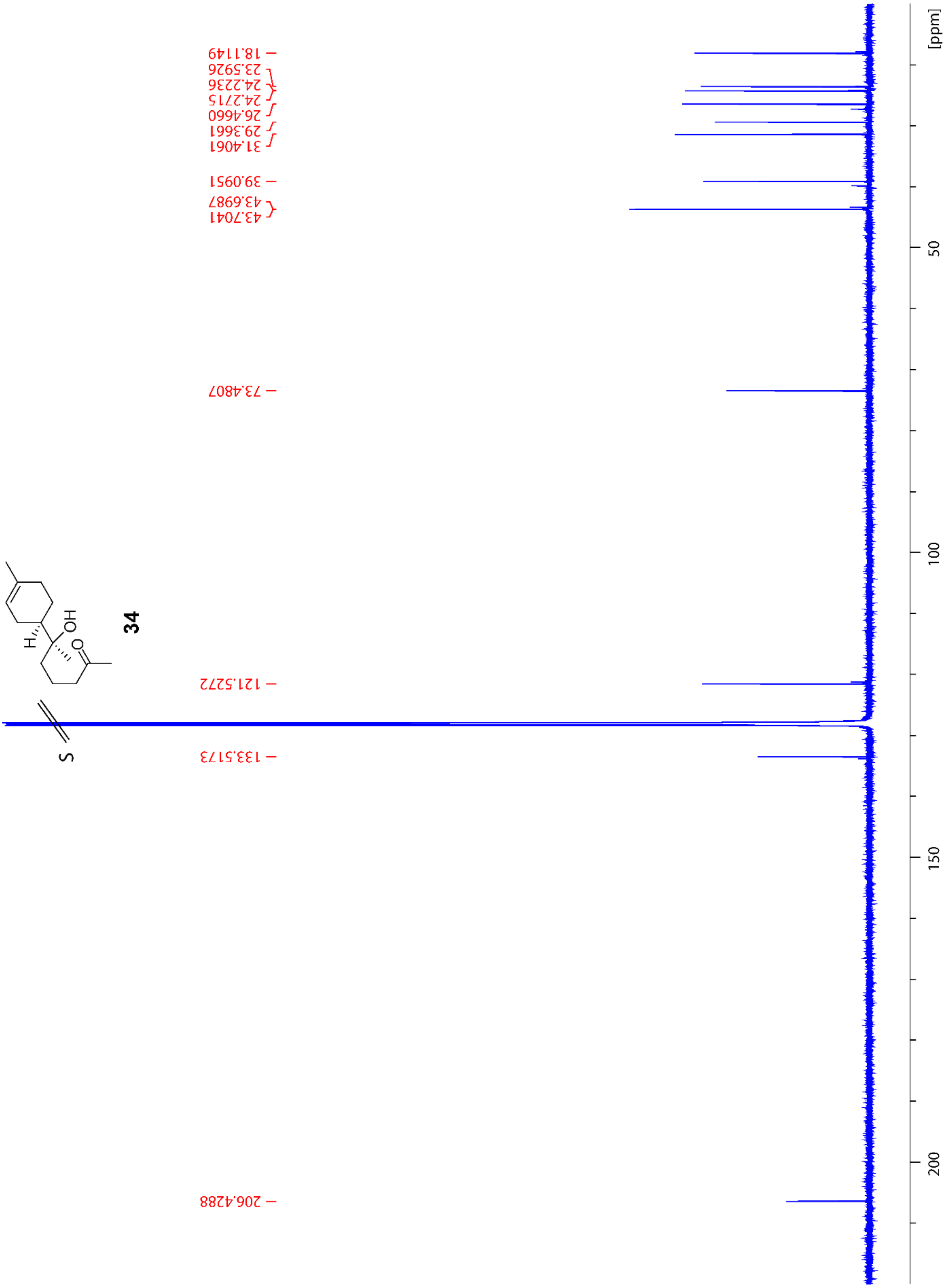

Figure S84. ^{13}C-NMR spectrum (C_6D_6, 175 MHz) of **34**. S indicates solvent peak.

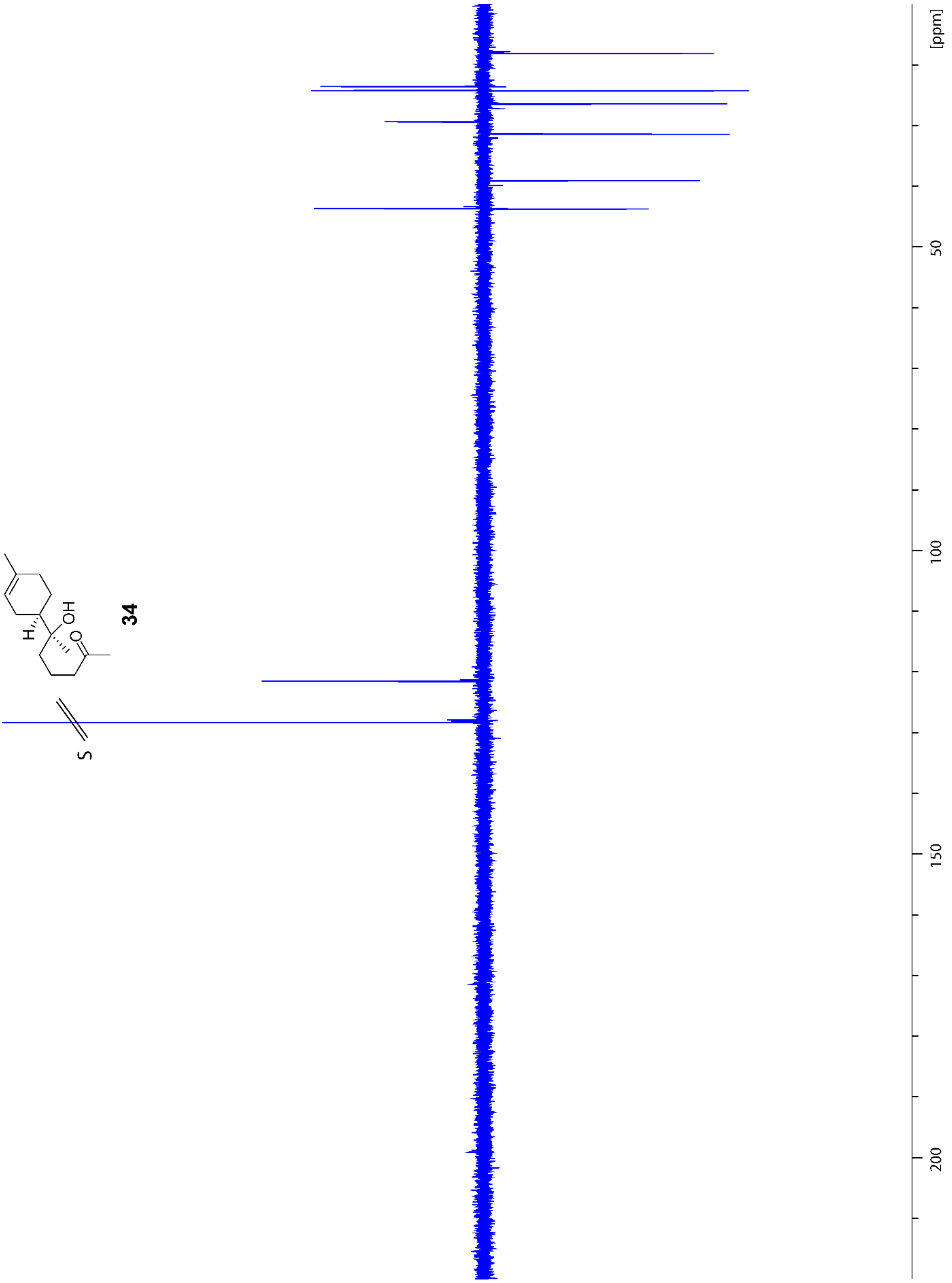

Figure S85. ^{13}C-DEPT135 spectrum (C_6D_6, 175 MHz) of **34**. S indicates solvent peak.

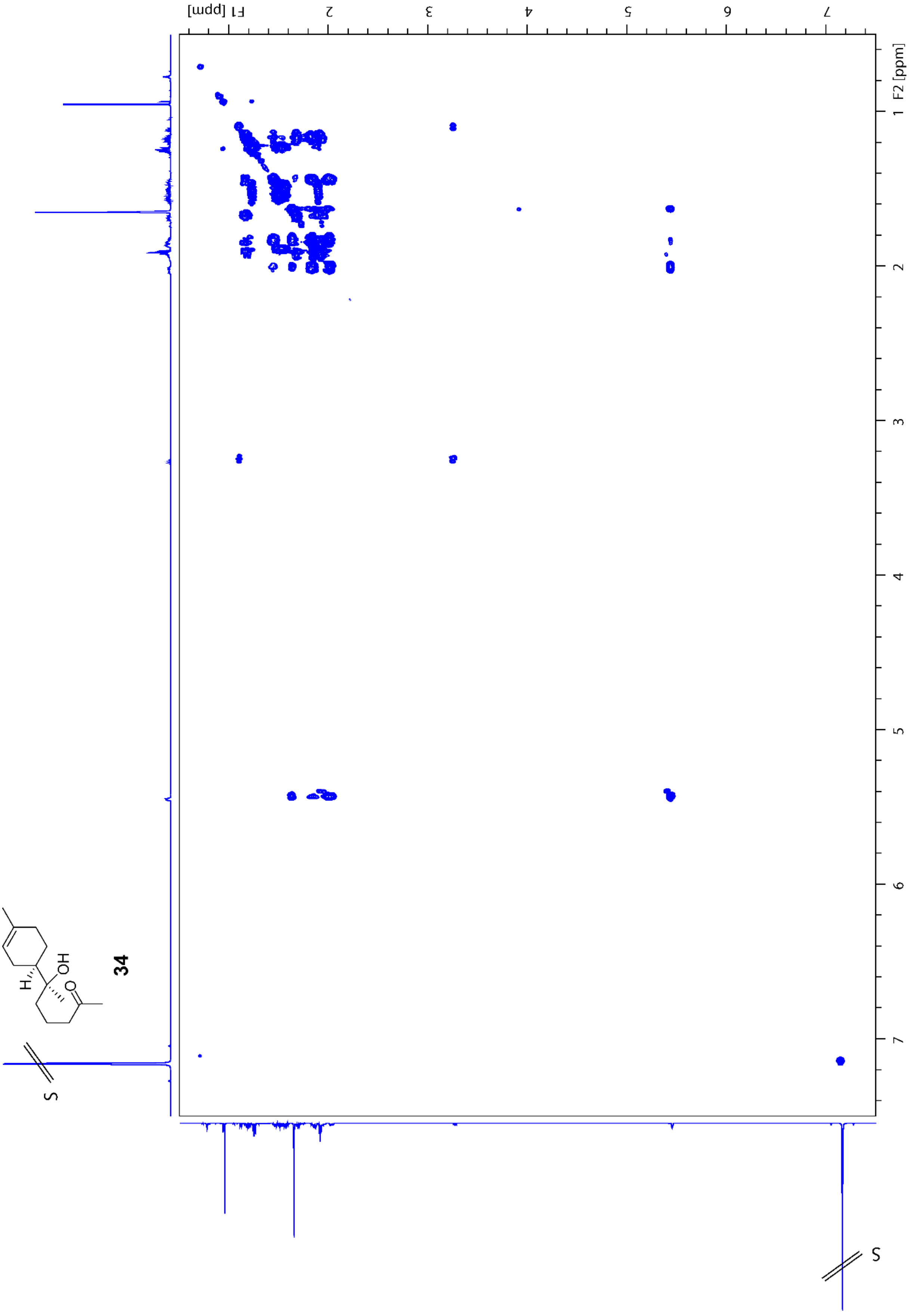

Figure S86. $^{1}H,^{1}H$-COSY spectrum (C_6D_6) of **34**. S indicates solvent peaks.

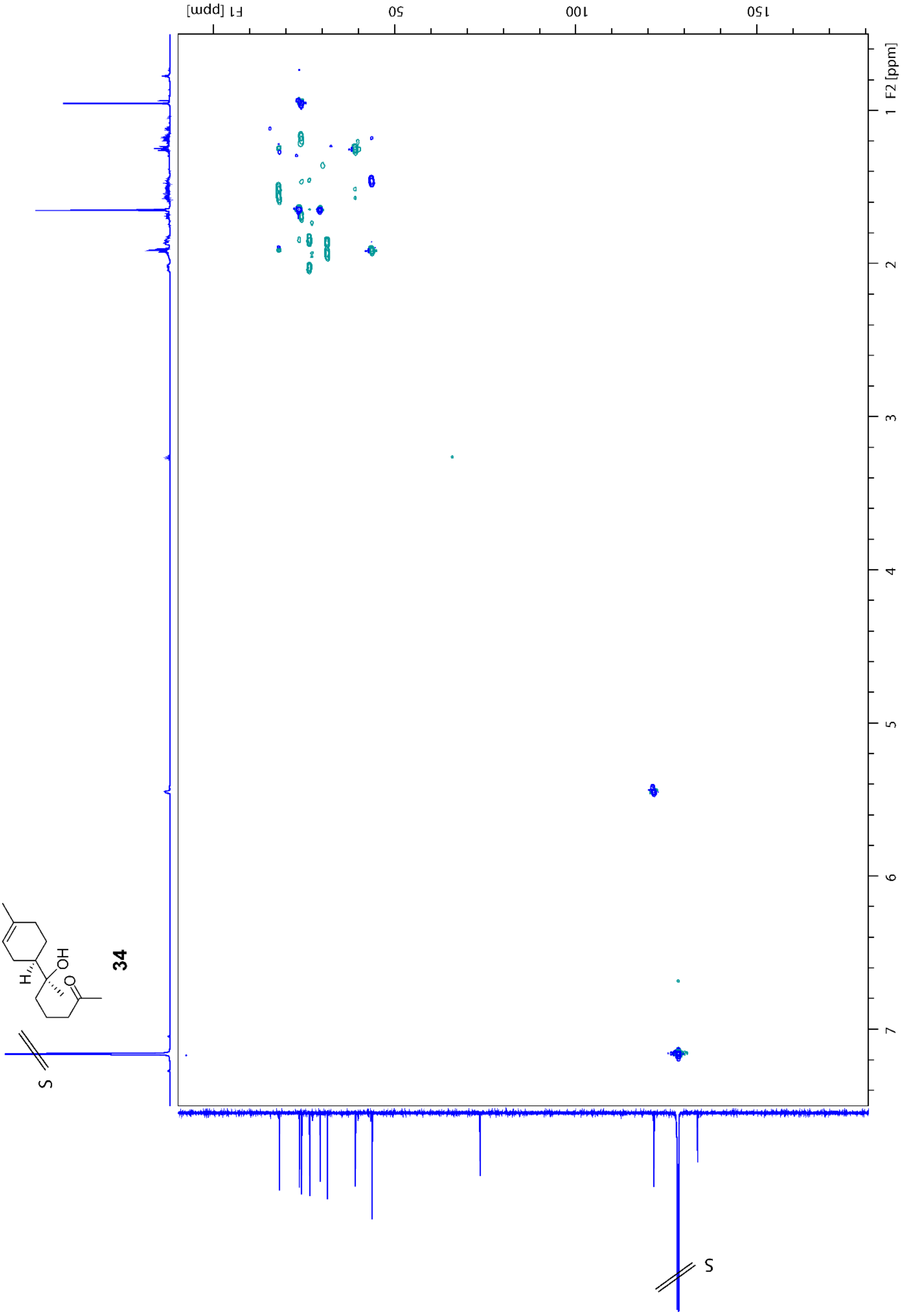

Figure S87. HSQC spectrum (C_6D_6) of **34**. S indicates solvent peaks.

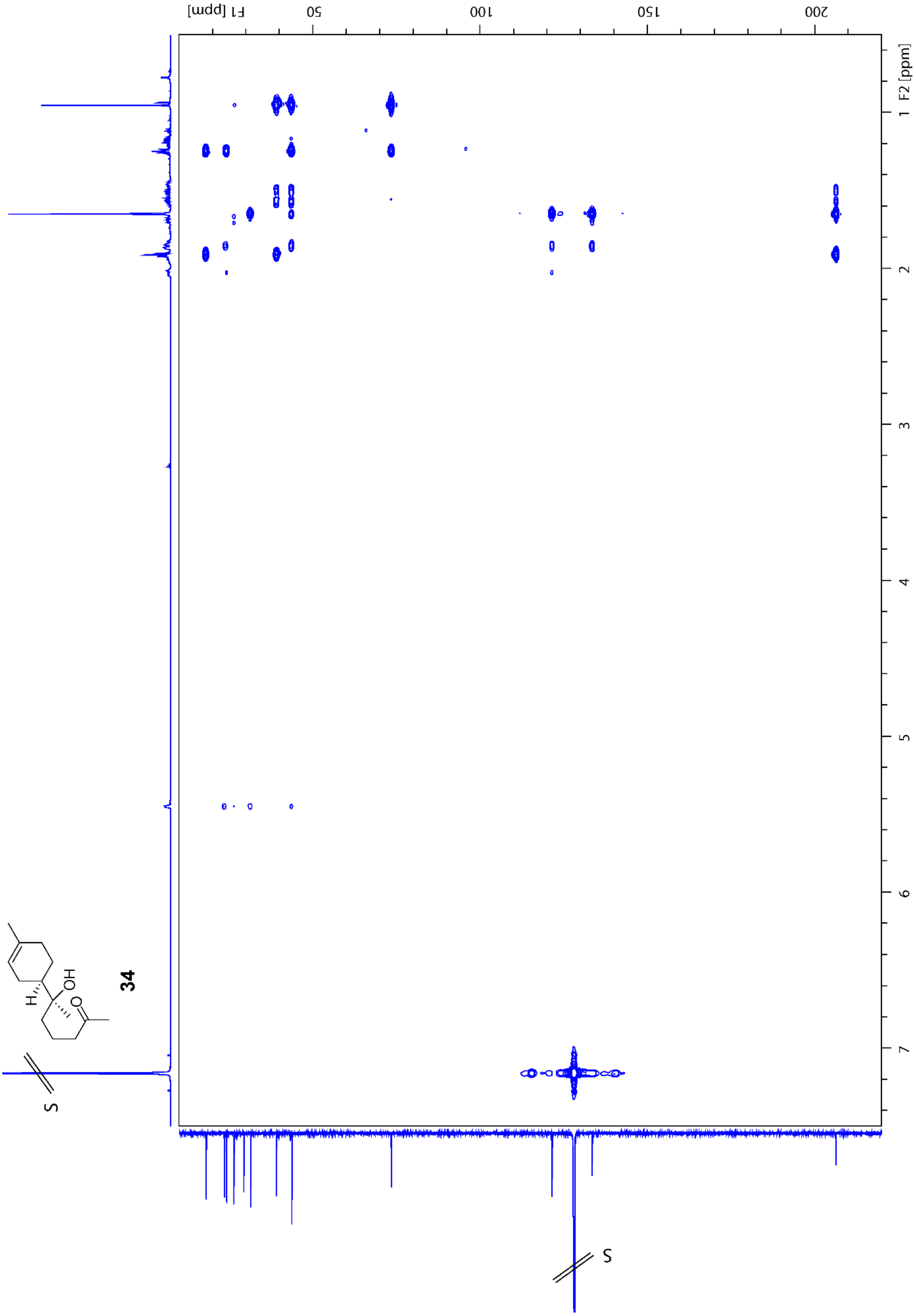

Figure S88. HMBC spectrum (C_6D_6) of **34**. S indicates solvent peaks.

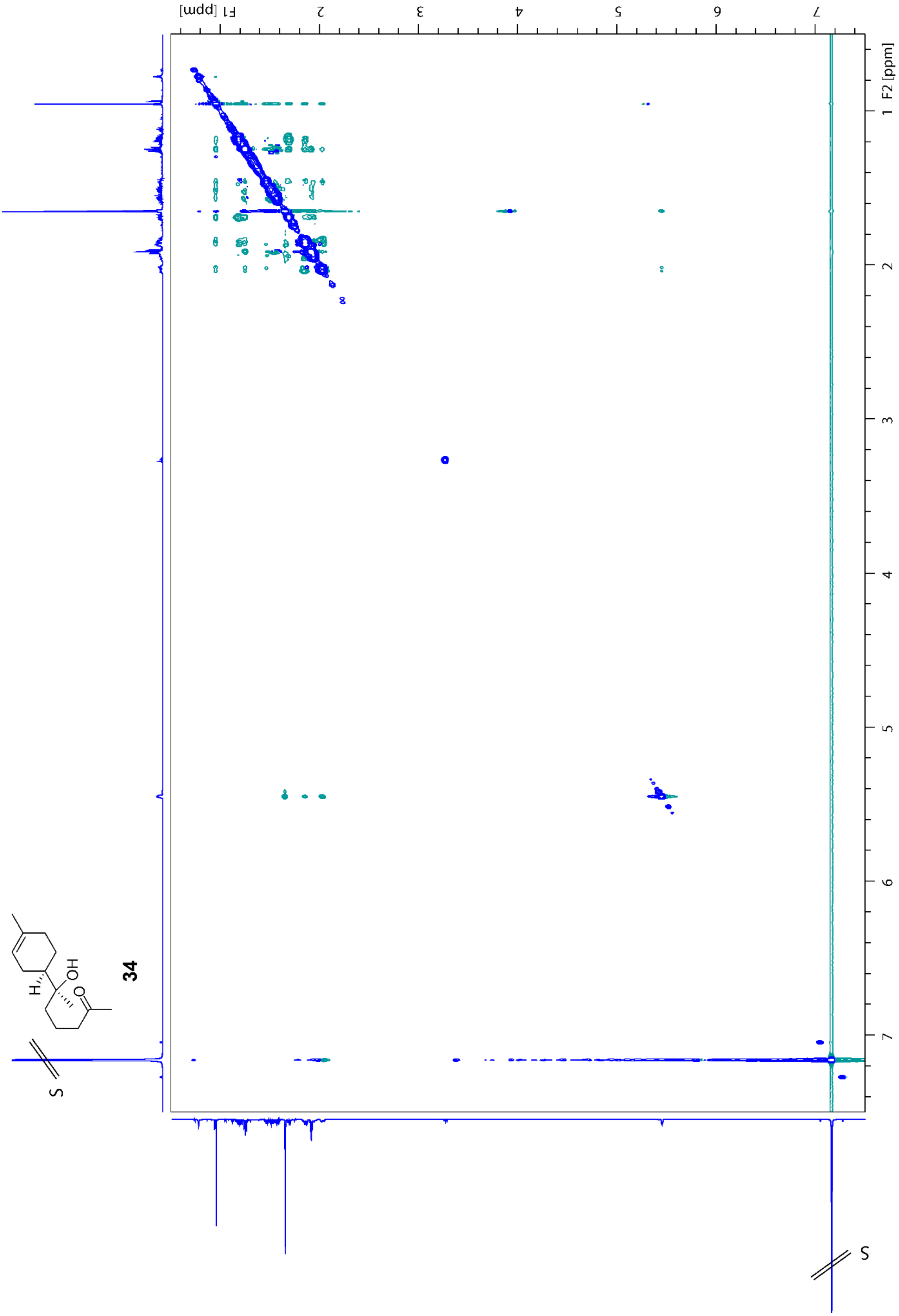

Figure S89. NOESY spectrum (C_6D_6) of **34**. S indicates solvent peaks.

Synthetic route to trisammonium (2*E*)-3,7-dimethylocta-2,7-dien-1-yl diphosphate (36) and trisammonium (2*E*)-3-methyl-7-oxooct-2-en-1-yl diphosphate (37)

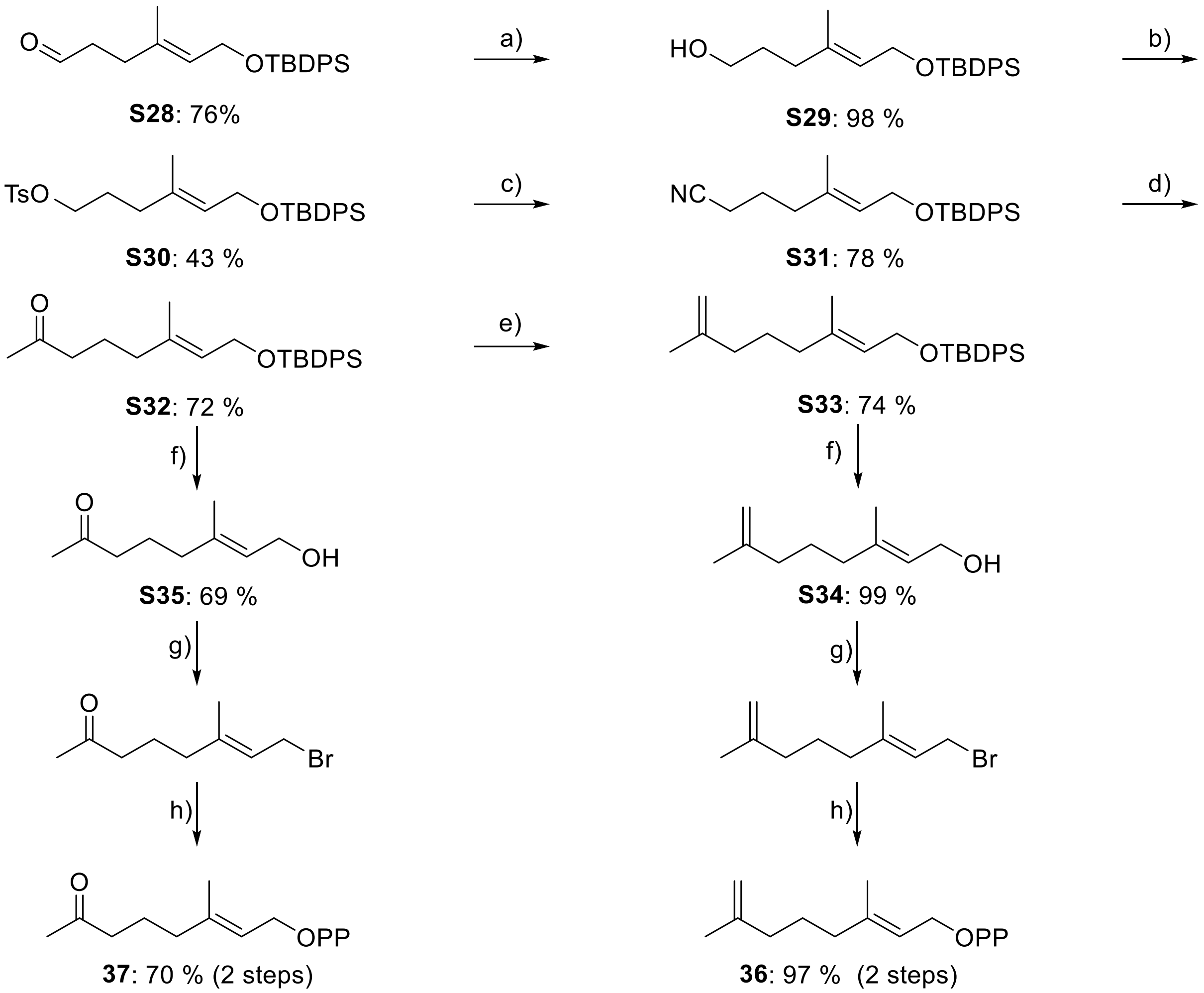

Scheme S6. Synthesis of GPP analogues **36** and **37**. a) $NaBH_4$, EtOH, 0 °C, 1 h; b) TsCl, DMAP, CH_2Cl_2, overnight; c) KCN, DMSO, overnight; d) MeLi, Et_2O, –78 °C to room temperature, overnight; e) CH_3PPh_3I, *n*-BuLi, THF, –78 °C to room temperature, overnight; f) TBAF, THF, 1.5 h; g) PBr_3, Et_2O, 0 °C, 1 h; h) $(NBu_4)_3HP_2O_7$, MeCN, overnight.

Compounds **36** and **37** were synthesized by the same procedures as compounds **15** and **16** reported above.

(*E*)-6-((*tert*-Butyldiphenylsilyl)oxy-4methylhex-4-enal (S28): Colorless oil. Yield: 4.33 g, 11.81 mmol, 76%. EI-MS (70 eV): *m*/*z* (%) = 309 (9), 253 (1), 231 (22), 213 (5), 199 (100), 181 (13), 169 (2), 152 (5), 139 (13), 121 (5), 105 (6), 91 (5), 77 (12), 67 (3), 57 (28), 41 (19). GC (HP5-MS): I = 2560. ^{1}H-NMR (C_6D_6, 500 MHz): δ = 9.26 (t, $^3J_{H,H}$ = 1.6 Hz, 1H), 7.84 – 7.78 (m, 4H), 7.28 – 7.21 (m, 6H), 5.40 (tq, $^3J_{H,H}$ = 6.3 Hz, $^4J_{H,H}$ =1.4 Hz, 1H), 4.25 (dq, $^3J_{H,H}$ = 6.3 Hz, $^4J_{H,H}$ =1.0 Hz, 2H), 1.96 – 1.91 (m, 2H), 1.89 – 1.84 (m, 2H), 1.19 (s, 9H), 1.14 (d, $^4J_{H,H}$ = 1.2 Hz, 3H) ppm. ^{13}C-NMR (C_6D_6, 126 MHz): δ = 200.15 (CH), 136.06 (4 x CH), 135.44 (C_q), 134.41 (2 x C_q), 129.99 (2 x CH), 128.11 (4 x CH), 125.17 (CH), 61.34 (CH_2), 41.78 (CH_2), 31.60 (CH_2), 27.09 (3 x CH_3), 19.46 (C_q), 16.22 (CH_3) ppm.

(*E*)-6-((*tert*-Butyldiphenylsilyl)oxy-4methylhex-4-en-1-ol (S29): Colorless oil. Yield: 4.28 g, 11.6 mmol, 98%. TLC (cyclohexane/ethyl acetate, 4:1): R_f = 0.27. EI-MS (70 eV): *m*/*z* (%) = 311 (21), 281 (11), 233 (7), 199 (100), 181 (14), 173 (2), 152 (5), 139 (8), 121 (6), 105 (6),

95 (12), 77 (13), 67 (9), 57 (32), 41 (21). GC (HP5-MS): I = 2631. ^{1}H-NMR (C_6D_6, 500 MHz): δ = 7.87 – 7.80 (m, 4H), 7.28 – 7.20 (m, 6H), 5.56 (tq, $^{3}J_{H,H}$ = 6.4 Hz, $^{4}J_{H,H}$ =1.3 Hz, 1H), 4.32 (dq, $^{3}J_{H,H}$ = 6.4 Hz, $^{4}J_{H,H}$ = 0.9 Hz, 2H), 3.30 (t, $^{3}J_{H,H}$ = 6.5 Hz, 2H), 1.95 – 1.85 (m, 2H), 1.46 – 1.38 (m, 2H), 1.29 (d, $^{4}J_{H,H}$ = 1.3 Hz, 3H), 1.19 (s, 9H) ppm. ^{13}C-NMR (C_6D_6, 126 MHz): δ = 137.19 (C_q), 136.08 (4 x CH), 134.51 (2 x C_q), 129.94 (2 x CH), 128.08 (4 x CH), 124.71 (CH), 62.32 (CH_2), 61.46 (CH_2), 36.04 (CH_2), 31.03 (CH_2), 27.11 (3 x CH_3), 19.48 (C_q), 16.20 (CH_3) ppm.

(*E*)-6-((*tert*-Butyldiphenylsilyl)oxy-4methylhex-4-en-1-yl 4-methylbenzenesulfonate (S30): Colorless oil. Yield: 2.77 g, 5.3 mmol, 43%. TLC (cyclohexane/ethyl acetate, 10:1): R_f = 0.27. ^{1}H-NMR (C_6D_6, 500 MHz): δ = 7.82 – 7.79 (m, 4H), 7.78 – 7.75 (m, 2H), 7.28 – 7.20 (m, 6H), 6.74 – 6.67 (m, 2H), 5.36 (tq, $^{3}J_{H,H}$ = 6.3 Hz, $^{4}J_{H,H}$ = 1.3 Hz, 1H), 4.26 – 4.19 (m, 2H), 3.79 (t, $^{3}J_{H,H}$ = 6.5 Hz, 2H), 1.83 (s, 3H), 1.72 – 1.64 (m, 2H), 1.43 – 1.33 (m, 2H), 1.18 (s, 9H), 1.11 (d, $^{4}J_{H,H}$ = 1.2 Hz, 3H) ppm. ^{13}C-NMR (C_6D_6, 126 MHz): δ = 144.10 (C_q), 136.05 (4 x CH), 135.54 (C_q), 134.67 (C_q), 134.42 (2 x C_q), 129.99 (2 x CH), 129.80 (2 x CH), 128.18 (2 x CH), 128.10 (4 x CH), 125.37 (CH), 69.76 (CH_2), 61.30 (CH_2), 35.10 (CH_2), 27.10 (3 x CH_3), 21.14 (CH_3), 19.46 (C_q), 15.97 (CH_3) ppm.

(*E*)-7-((*tert*-Butyldiphenylsilyl)oxy)-5-methylhept-5-enenitrile (S31): Colorless oil. Yield: 1.56 g, 4.13 mmol, 78%. TLC (cyclohexane/ethyl acetate, 10:1): R_f = 0.28. EI-MS (70 eV): *m*/*z* (%) = 320 (65), 290 (52), 242 (7), 199 (100), 181 (15), 152 (3), 135 (6), 121 (6), 105 (6), 91 (2), 77 (13), 67 (3), 57 (35), 41 (21). GC (HP5-MS): I = 2749. ^{1}H-NMR (C_6D_6, 500 MHz): δ = 7.83 – 7.78 (m, 4H), 7.29 – 7.22 (m, 6H), 5.38 (tq, $^{3}J_{H,H}$ = 6.3 Hz, $^{4}J_{H,H}$ = 1.3 Hz, 1H), 4.25 (d, $^{3}J_{H,H}$ = 6.3 Hz, 2H), 1.63 – 1.55 (m, 2H), 1.34 (t, $^{3}J_{H,H}$ = 7.2 Hz, 2H), 1.19 (s, 9H), 1.08 (q, $^{4}J_{H,H}$ = 1.0 Hz, 3H), 1.10 – 1.01 (m, 2H) ppm. ^{13}C-NMR (C_6D_6, 126 MHz): δ = 136.05 (4 x CH), 134.87 (C_q), 134.37 (2 x C_q), 130.05 (2 x CH), 128.11 (4 x CH), 126.07 (CH), 119.21 (C_q), 61.26 (CH_2), 37.99 (CH_2), 27.08 (3 x CH_3), 23.34 (CH_2), 19.45 (C_q), 15.93 (CH_2), 15.76 (CH_3) ppm.

(*E*)-8-((*tert*-Butyldiphenylsilyl)oxy)-6-methyloct-6-en-2-one (S32): Colorless oil. Yield: 1.18 g, 3.00 mmol, 72%. TLC (cyclohexane/ethyl acetate, 10:1): R_f = 0.28. EI-MS (70 eV): *m*/*z* (%) = 337 (4), 267 (2), 259 (10), 239 (2), 229 (3), 199 (100), 181 (10), 163 (1), 152 (3), 139 (6), 121 (17), 105 (4), 93 (2), 77 (8), 67 (2), 57 (15), 43 (24). GC (HP5-MS): I = 2737. ^{1}H-NMR (C_6D_6, 499 MHz): δ = 7.85 – 7.81 (m, 4H), 7.27 – 7.20 (m, 6H), 5.53 (tq, $^{3}J_{H,H}$ = 6.3 Hz, $^{4}J_{H,H}$ = 1.3 Hz, 1H), 4.32 (dq, $^{3}J_{H,H}$ = 6.4 Hz, $^{5}J_{H,H}$ = 0.8 Hz, 2H), 1.88 (t, $^{3}J_{H,H}$ = 7.3 Hz, 2H), 1.83 – 1.76 (m, 2H), 1.65 (s, 3H), 1.61 – 1.51 (m, 2H), 1.28 (d, $^{4}J_{H,H}$ = 1.2 Hz, 3H), 1.19 (s, 9H) ppm. ^{13}C-NMR (C_6D_6, 126 MHz): δ = 206.04 (C_q), 136.75 (C_q), 136.07 (4 x CH), 134.48 (2 x C_q), 129.97 (2 x C_q), 128.10 (4 x CH), 125.25 (CH), 61.45 (CH_2), 42.46 (CH_2), 38.99 (CH_2), 29.45 (CH_3), 27.11 (3 x CH_2), 21.77 (CH_2), 19.48 (C_q), 15.94 (CH_3) ppm.

(*E*)-*tert*-Butyl(3,7-dimethylocta-2,7-dien-1-yl)oxy)diphenylsilane (S33): Colorless oil. Yield: 370 mg, 0.94 mmol, 74%. TLC (cyclohexane/ethyl acetate, 100/1): R_f = 0.21. EI-MS (70 eV): *m*/*z* (%) = 335 (10), 305 (1), 293 (3), 229 (2), 257 (27), 239 (32), 227 (2), 213 (4), 199 (100), 181 (18), 161 (8), 152 (4), 135 (7), 121 (8), 105 (5), 97 (4), 77 (11), 67 (5), 56 (29), 41 (22). GC (HP5-MS): I = 2563. ^{1}H-NMR (C_6D_6, 700 MHz): δ = 7.88 – 7.82 (m, 4H), 7.27 – 7.22 (m, 6H), 5.60 (tq, $^{3}J_{H,H}$ = 6.3 Hz, $^{3}J_{H,H}$ = 1.3 Hz, 1H), 4.83 – 4.77 (m, 2H), 4.35 (dq, $^{3}J_{H,H}$ = 6.2 Hz, $^{5}J_{H,H}$ = 0.9 Hz, 2H), 1.92 – 1.87 (m, 4H), 1.63 (s, 3H), 1.51 – 1.44 (m, 2H), 1.31 (d, $^{4}J_{H,H}$ = 1.3 Hz, 3H), 1.20 (s, 9H) ppm. ^{13}C-NMR (C_6D_6, 176 MHz): δ = 145.67 (C_q), 137.14 (C_q), 136.09 (4 x CH), 134.56 (2 x C_q), 129.93 (2 x CH), 128.07 (4 x CH), 124.86 (CH), 110.45 (CH), 61.53 (CH_2), 39.32 (CH_2), 37.59 (CH_2), 27.11 (3 x CH_3), 25.91 (CH_2), 22.48 (CH_3), 19.49 (C_q), 16.17 (CH_3) ppm.

(*E*)- 3,7-Dimethylocta-2,7-dien-1-ol (S34): Colorless oil. Yield: 150 mg, 0.97 mmol, 99%. TLC (pentane/diethyl ether, 1/1): R_f = 0.38. EI-MS (70 eV): *m/z* (%) = 136 (5), 121 (6), 109 (11), 96 (21), 83 (32), 69 (47), 55 (49), 41 (100). GC (HP5-MS): *I* = 1253. ^{1}H-NMR (C_6D_6, 500 MHz): δ = 5.37 (tq, $^3J_{H,H}$ = 6.7 Hz, $^4J_{H,H}$ =1.3 Hz, 1H), 4.83 – 4.78 (m, 2H), 3.97 (d, $^3J_{H,H}$ = 6.7 Hz, 2H), 1.96 – 1.83 (m, 4H), 1.63 (t, $^4J_{H,H}$ = 1.1 Hz, 3H), 1.51 – 1.45 (m, 2H), 1.44 (d, $^4J_{H,H}$ = 1.3 Hz, 3H) ppm. ^{13}C-NMR (C_6D_6, 126 MHz): δ = 145.64 (C_q), 138.12 (C_q), 125.01 (CH), 110.47 (CH_2), 59.36 (CH_2), 39.33 (CH_2), 37.64 (CH_2), 25.97 (CH_2), 22.46 (CH_3), 16.07 (CH_3) ppm.

(2*E*)-3,7-Dimethyl-7-oxooct-2-en-1-ol (S35): Colorless oil. Yield: 82 mg, 0.53 mmol, 69%. TLC (diethyl ether, 100%): R_f = 0.35. EI-MS (70 eV): *m/z* (%) = 138 (5), 123 (4), 109 (4), 95 (26), 81 (11), 71 (15), 55 (12), 43 (100). GC (HP5-MS): *I* = 1353. ^{1}H-NMR (C_6D_6, 700 MHz): δ = 5.31 (tq, $^3J_{H,H}$ = 6.7 Hz, $^4J_{H,H}$ = 1.3 Hz, 1H), 3.94 (d, $^3J_{H,H}$ = 6.6 Hz, 2H), 1.87 (t, $^3J_{H,H}$ = 7.2 Hz, 2H), 1.82 – 1.76 (m, 2H), 1.64 (s, 3H), 1.58 – 1.52 (m, 2H), 1.41 (d, $^4J_{H,H}$ = 1.3 Hz, 3H), 0.64 (br s, 1H) ppm. ^{13}C-NMR (C_6D_6, 176 MHz): δ = 206.18 (C_q), 137.63 (C_q), 125.39 (CH), 59.29 (CH_2), 42.47 (CH_2), 38.99 (CH_2), 29.42 (CH_3), 21.77 (CH_2), 15.88 (CH_3) ppm.

Trisammonium (2*E*)-3,7-dimethylocta-2,7-dien-1-yl diphosphate (36): White powder. Yield: 160 mg, 0.44 mmol, 97%. ^{1}H-NMR (D_2O, 500 MHz) δ = 5.51 – 5.46 (m, 1H), 4.77 – 4.76 (m, 2H), 4.49 (t, $^3J_{H,H}$ = 6.6 Hz, 2H), 2.10 – 2.01 (m, 4H), 1.74 (s, 3H), 1.73 (s, 3H), 1.65 – 1.56 (m, 2H) ppm. ^{13}C-NMR (D_2O, 126 MHz): δ = 148.35 (C_q), 143.05 (C_q), 119.94 (d, $^3J_{C,P}$ = 8.5 Hz), 109.34 (CH_2), 62.54 (d, $^2J_{C,P}$ = 5.2 Hz), 38.51 (CH_2), 36.77 (CH_2), 25.09 (CH_2), 21.64 (CH_3), 15.62 (CH_3) ppm. ^{31}P NMR (202 MHz, D_2O): δ = –6.47 (d, $^2J_{P,P}$ = 22.2 Hz), –10.31 (d, $^2J_{P,P}$ = 22.4 Hz) ppm.

Trisammonium (2*E*)-3-methyl-7-oxooct-2-en-1-yl diphosphate (37): White powder. Yield: 137 mg, 0.37 mmol, 70%. ^{1}H-NMR (D_2O, 500 MHz): δ = 5.50 – 5.43 (m, 1H), 4.53 – 4.45 (m, 2H), 2.56 (t, $^3J_{H,H}$ = 7.4 Hz, 2H), 2.21 (s, 3H), 2.07 (t, $^3J_{H,H}$ = 7.4 Hz, 2H), 1.71 (d, $^4J_{H,H}$ = 1.7 Hz, 3H), 1.77 – 1.66 (m, 2H) ppm. ^{13}C-NMR (D_2O, 126 MHz) δ = 217.49 (C_q), 141.75 (C_q), 120.76 (d, $^3J_{C,P}$ = 8.5 Hz), 62.48 (d, $^2J_{C,P}$ = 5.2 Hz), 42.58 (CH_2), 38.02 (CH_2), 29.38 (CH_3), 21.17 (CH_2), 15.37 (CH_3). ^{31}P NMR (D_2O, 202 MHz): δ = –6.48 (d, $^2J_{P,P}$ = 22.3 Hz), –10.33 (d, $^2J_{P,P}$ = 22.3 Hz) ppm.

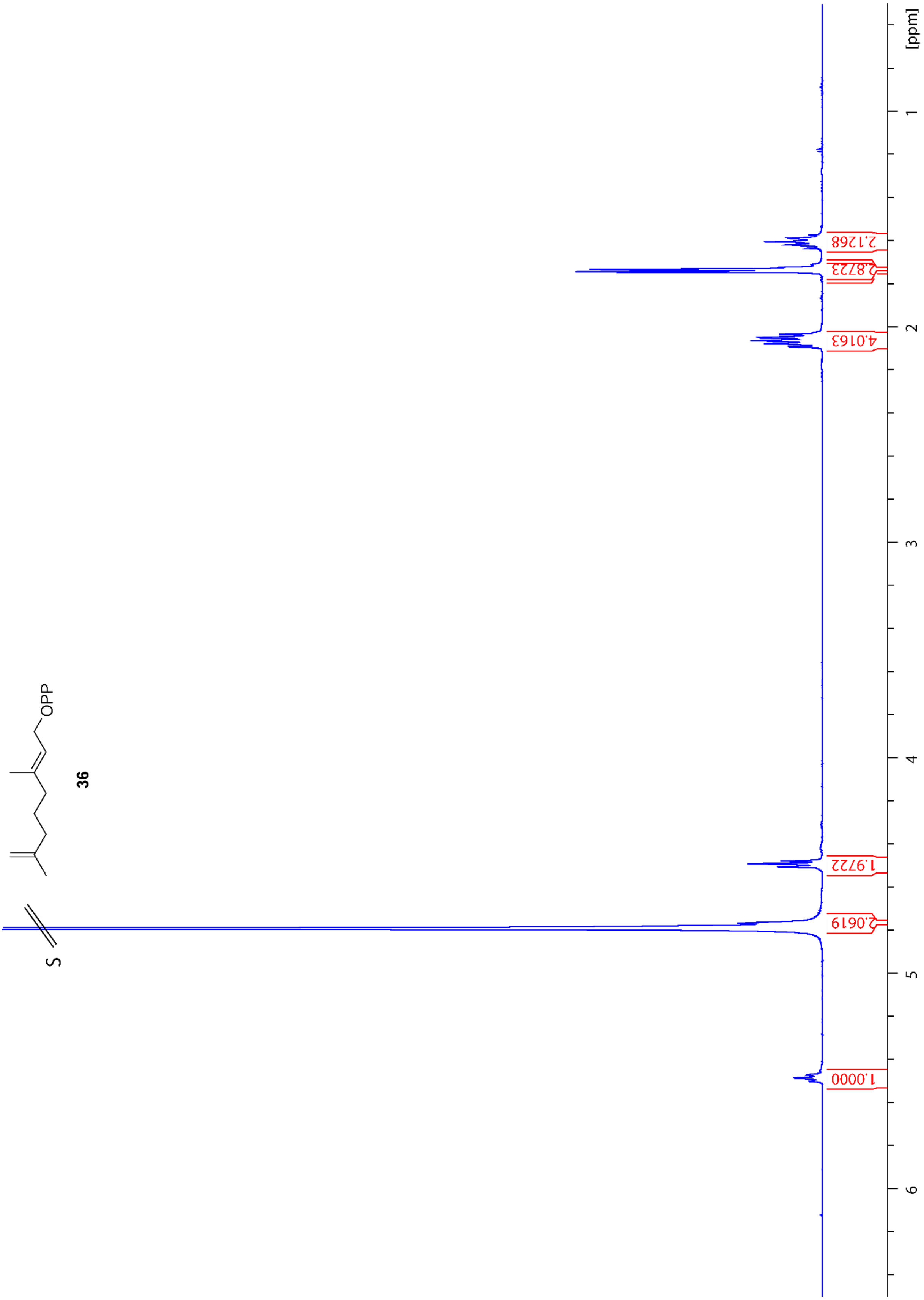

Figure S90. ^{1}H-NMR spectrum (D_2O, 500 MHz) of **36**. S indicates solvent peak.

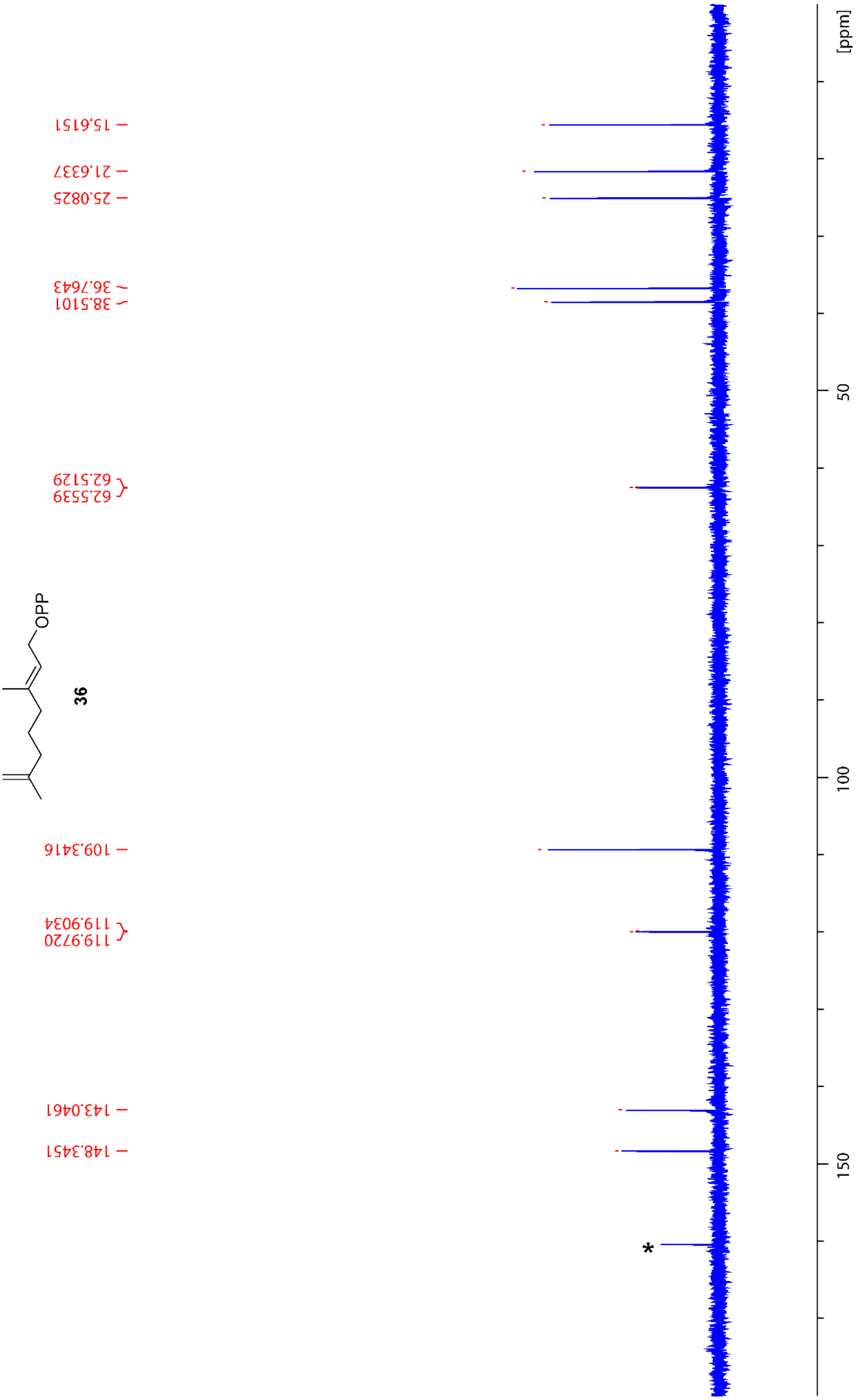

Figure S91. ^{13}C-NMR spectrum (D_2O, 126 MHz) of **36**. Asterisk indicates the peak from NH_4HCO_3.

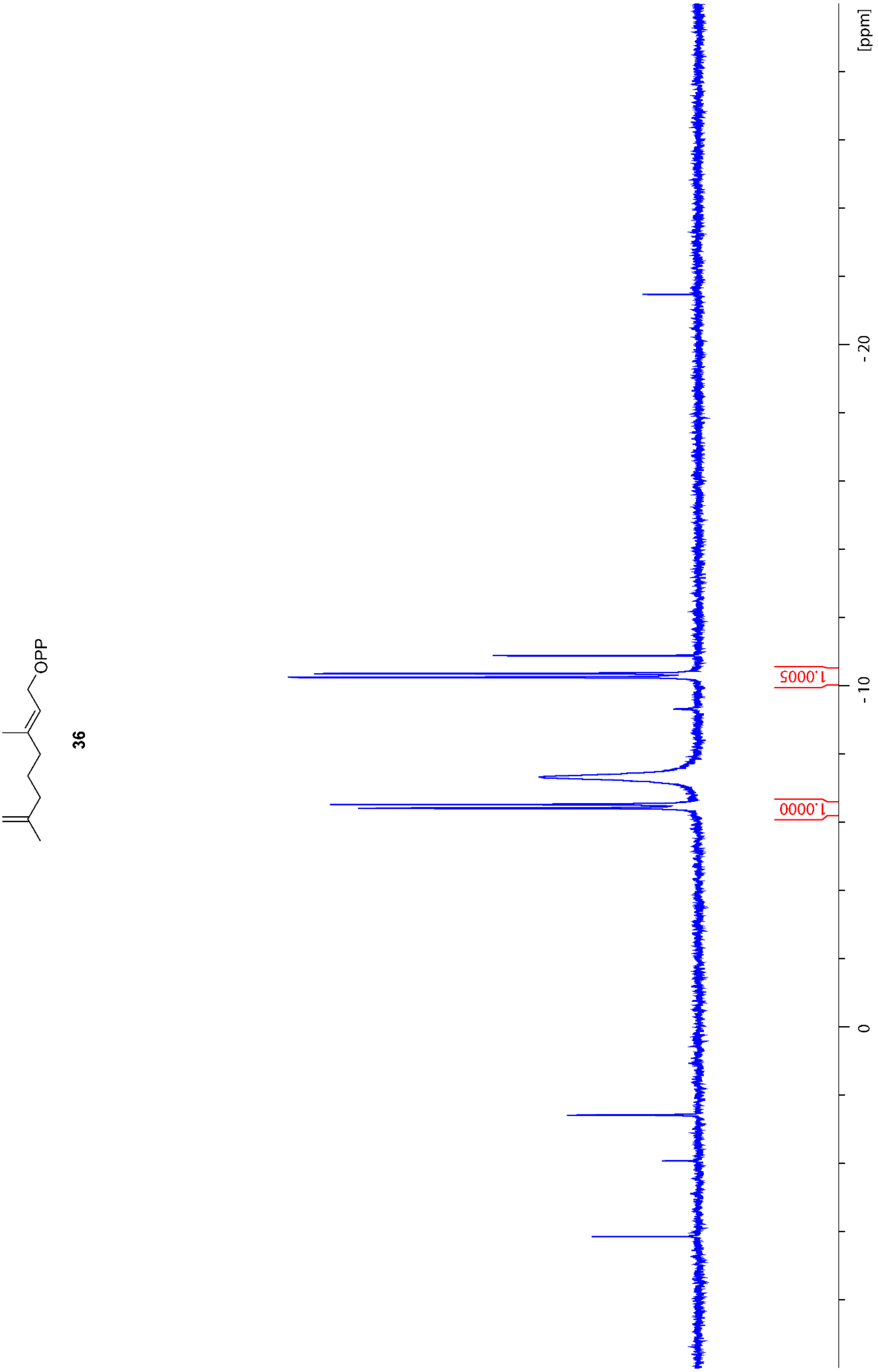

Figure S92. ^{31}P-NMR spectrum (D_2O, 202 MHz) of **36**.

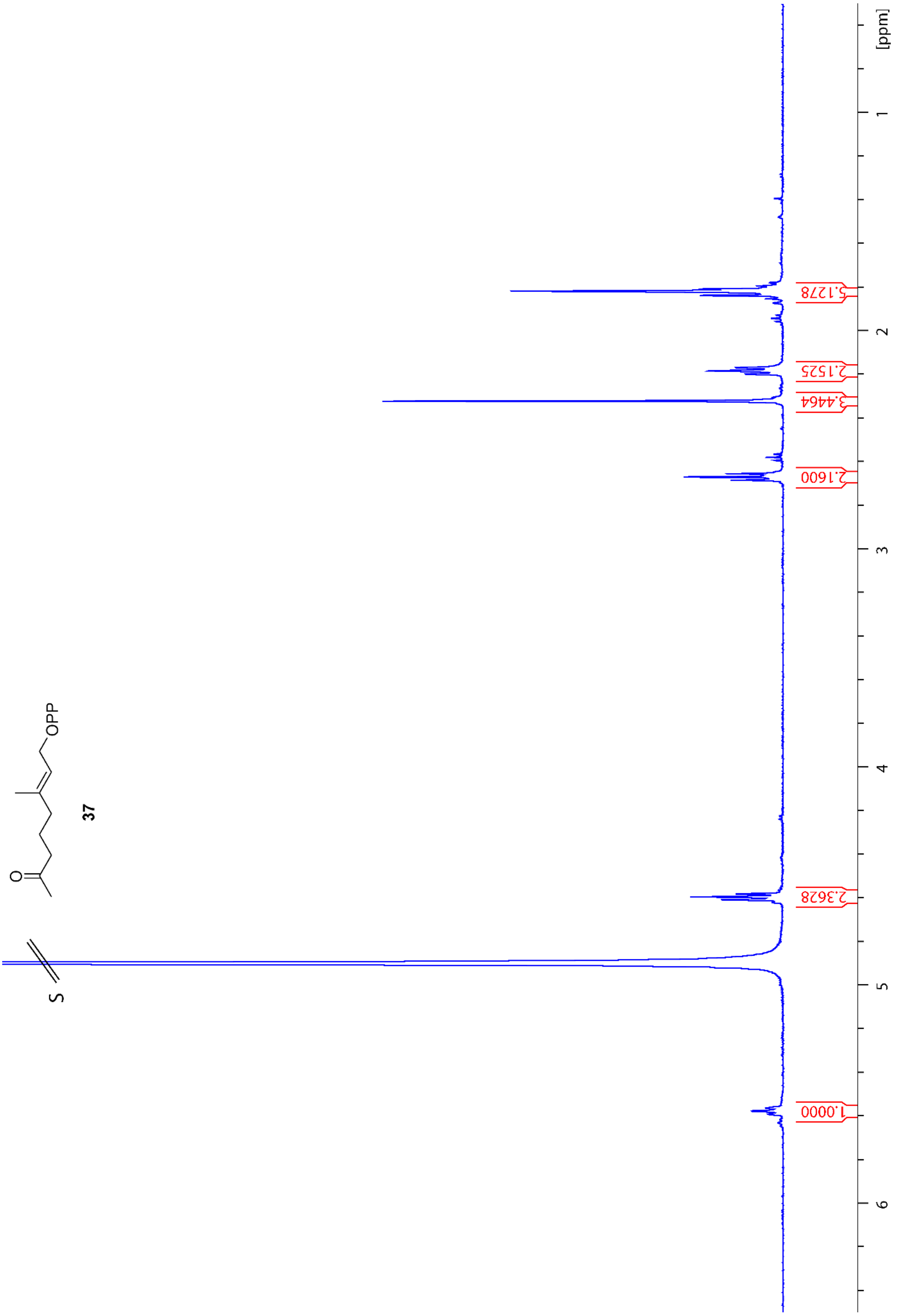

Figure S93. ^{1}H-NMR spectrum (D_2O, 500 MHz) of **37**. S indicates solvent peak.

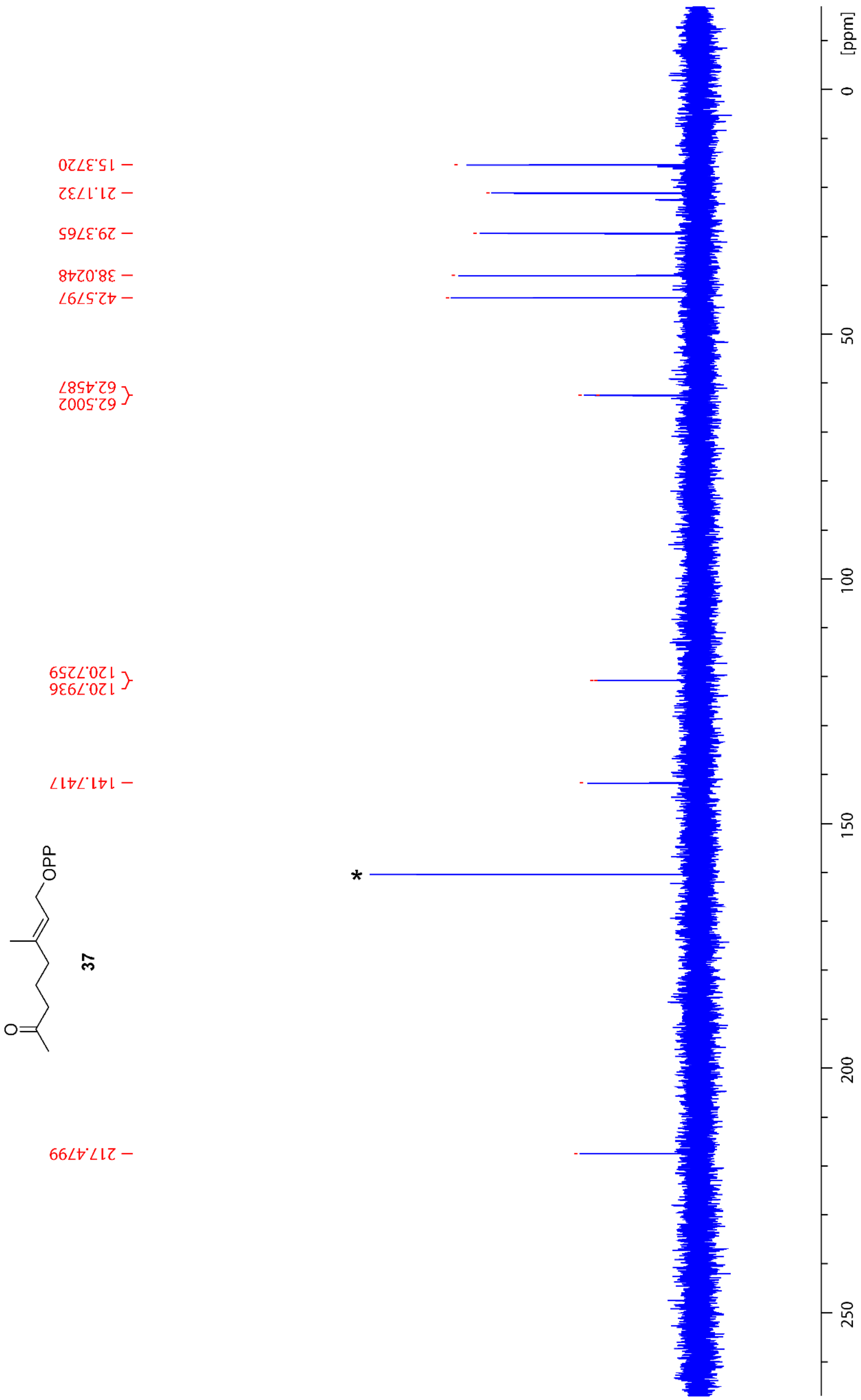

Figure S94. ^{13}C-NMR spectrum (D_2O, 126 MHz) of **37**. Asterisk indicates the peak from NH_4HCO_3.

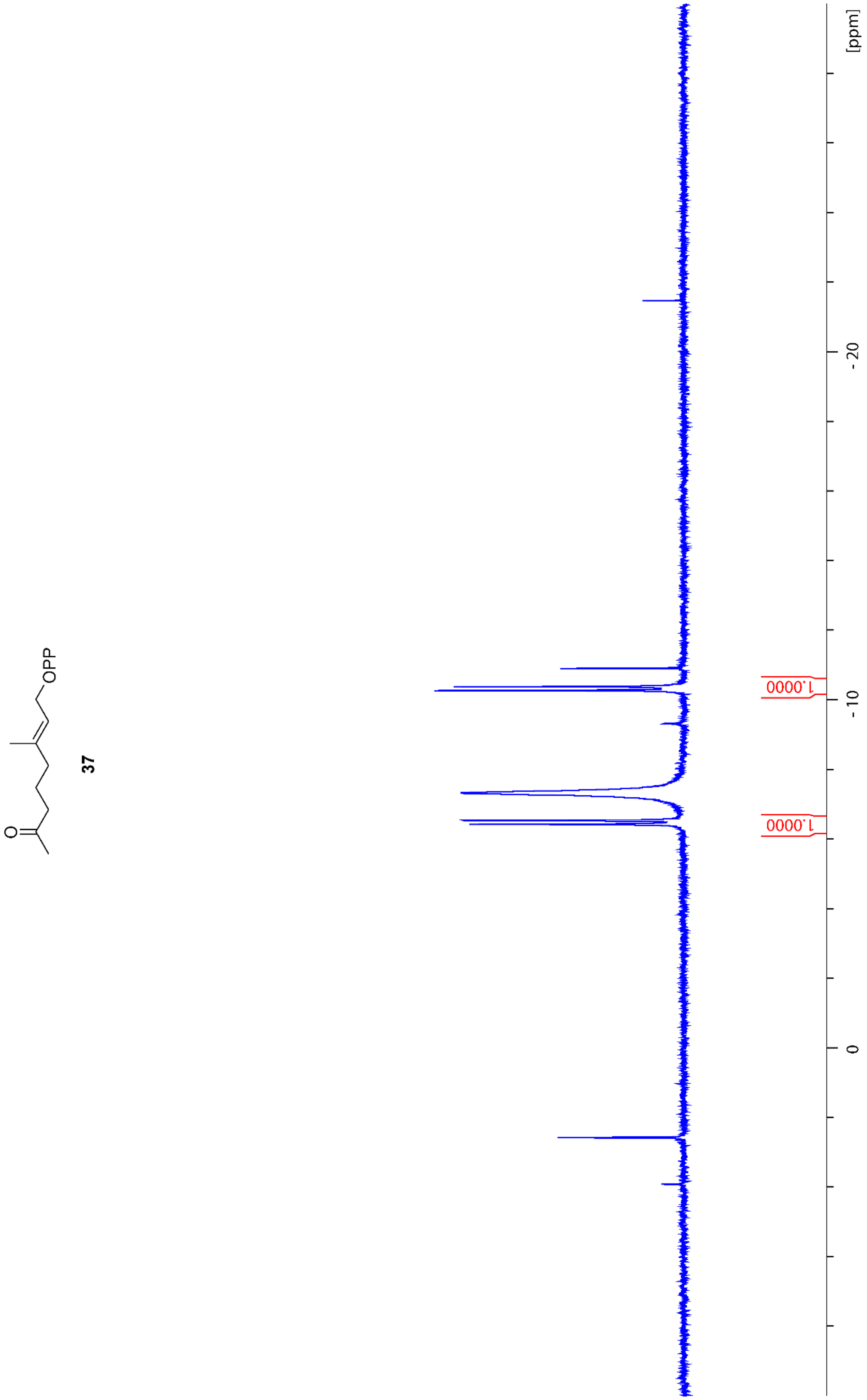

Figure S95. ^{31}P-NMR spectrum (D_2O, 202 MHz) of **37**.

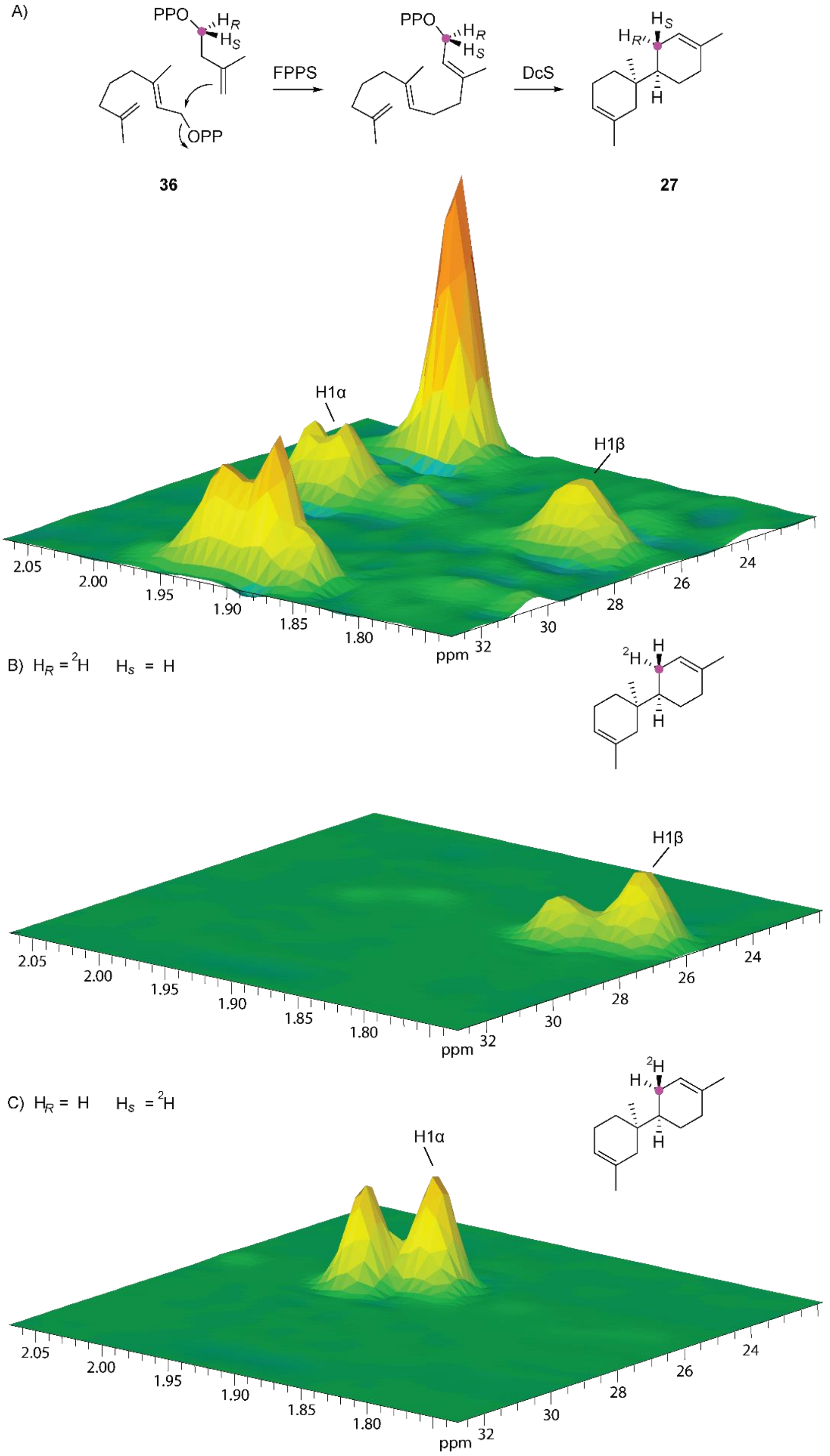

Figure S96. Absolute configuration of **27**. A) Partial HSQC spectra of purified **27**. Spectra resulting from incubation of DcS, FPPS and **36** with B) (*R*)-(1-^{13}C,1-^{2}H)IPP and C) (*S*)-(1-^{13}C,1-^{2}H)IPP.

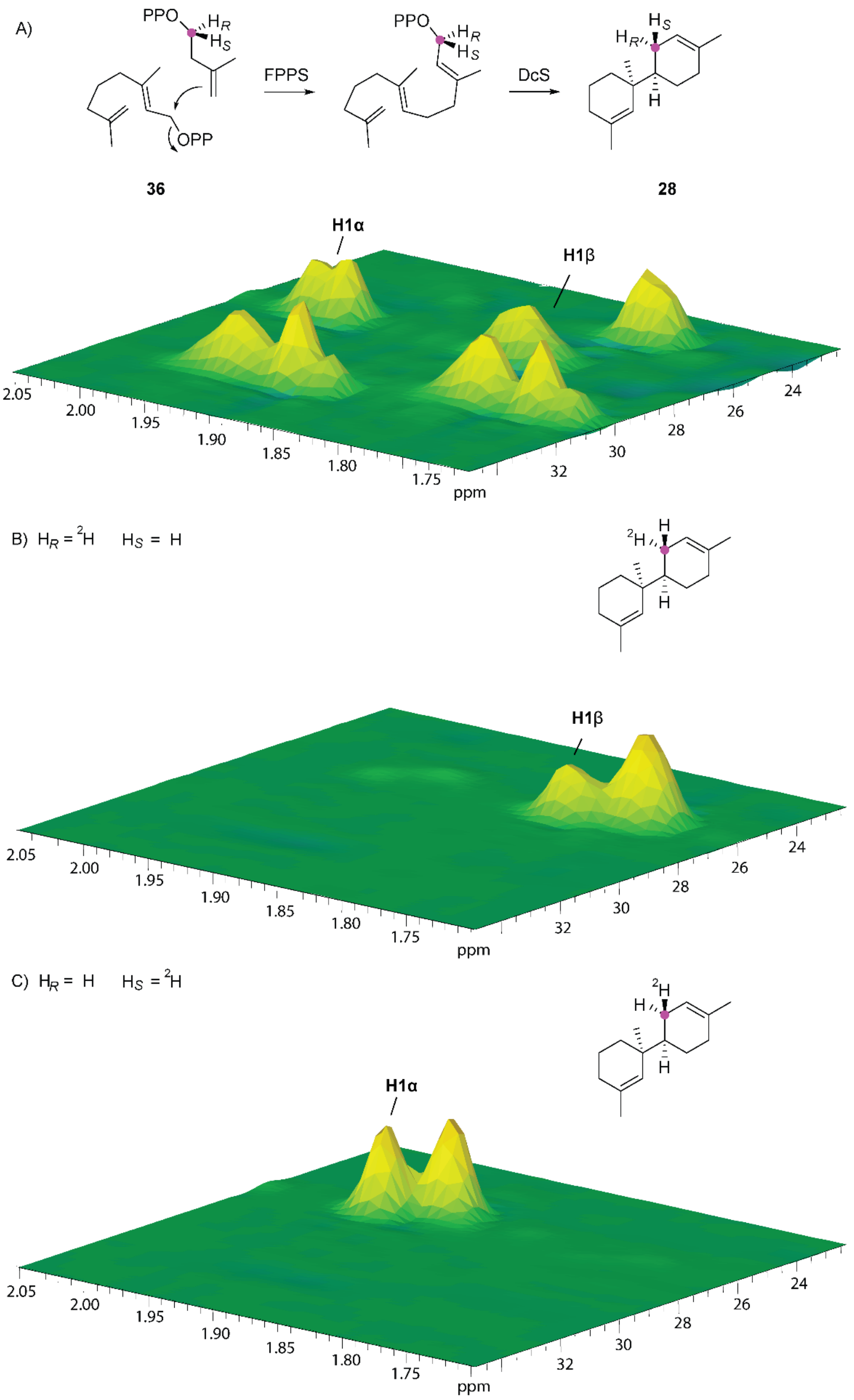

Figure S97. Absolute configuration of **28**. A) Partial HSQC spectra of purified **28**. Spectra resulting from incubation of DcS, FPPS and **36** with B) (*R*)-(1-^{13}C,1-^{2}H)IPP and C) (*S*)-(1-^{13}C,1-^{2}H)IPP.

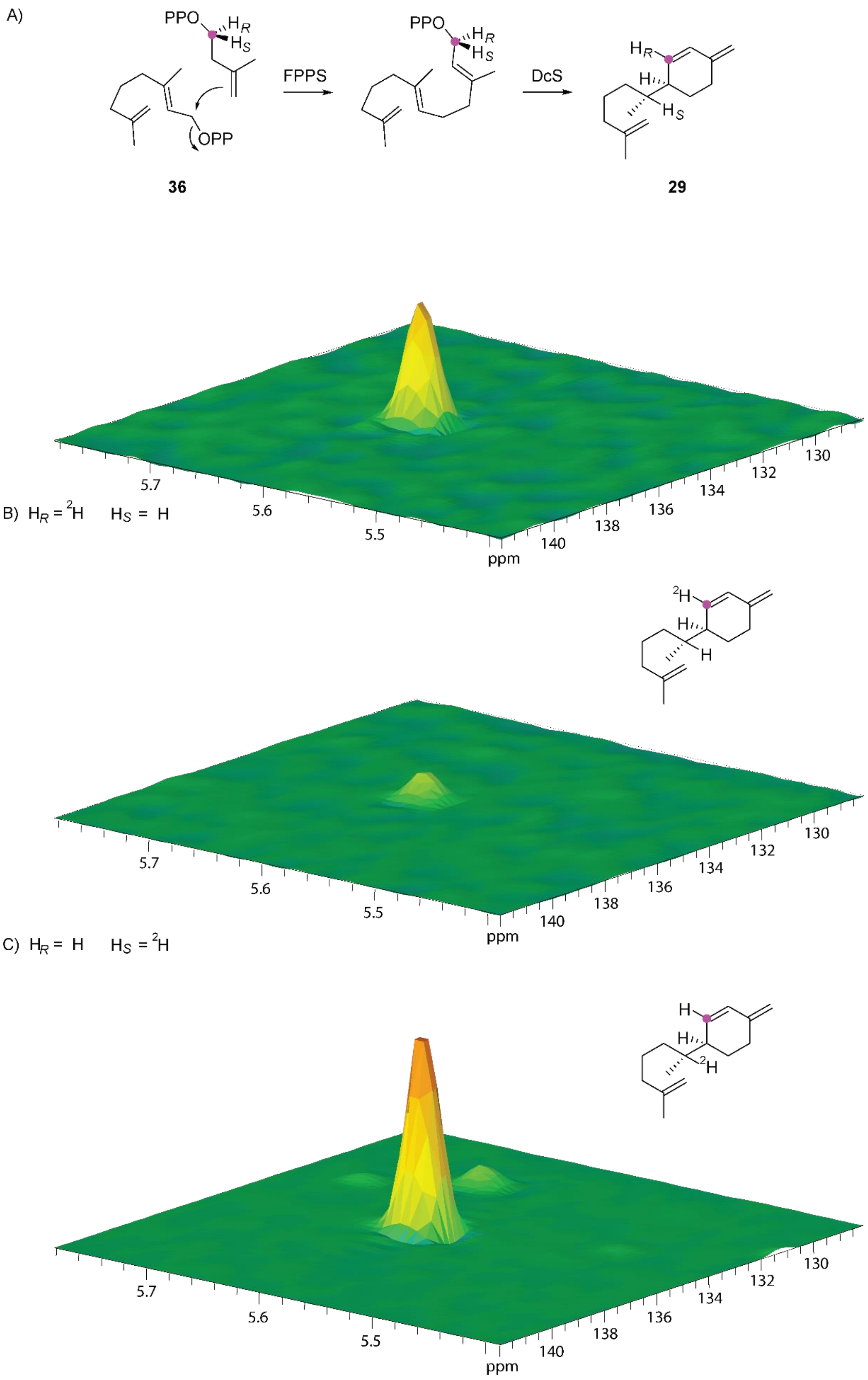

Figure S98. Absolute configuration of **29**. A) Partial HSQC spectra of purified **29**. Spectra resulting from incubation of DcS, FPPS and **36** with B) (R)-(1-^{13}C,1-^{2}H)IPP and C) (S)-(1-^{13}C,1-^{2}H)IPP.

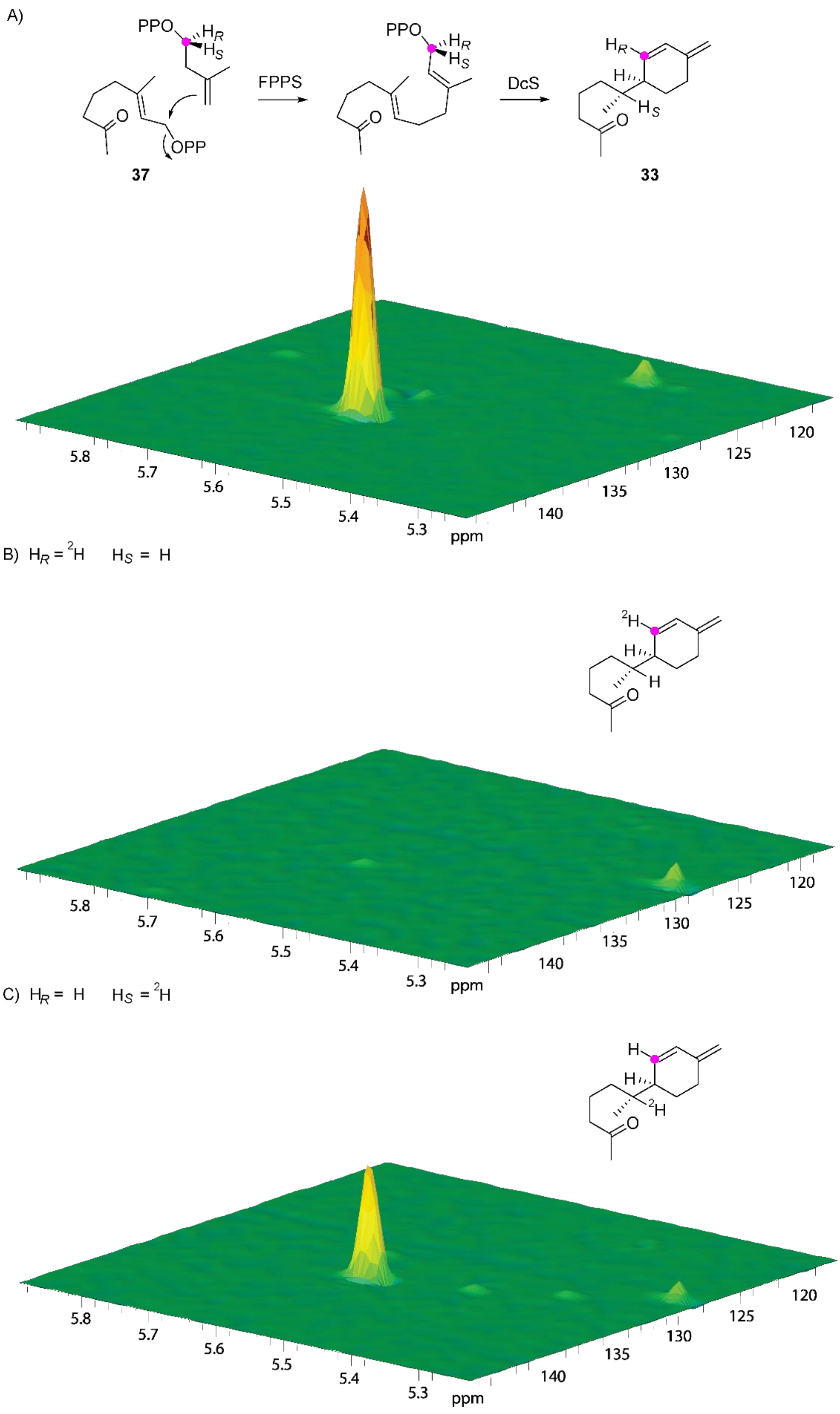

Figure S99. Absolute configuration of **33**. A) Partial HSQC spectra of purified **33**. Spectra resulting from incubation of DcS, FPPS and **37** with B) (*R*)-(1-^{13}C,1-^{2}H)IPP and C) (*S*)-(1-^{13}C,1-^{2}H)IPP.

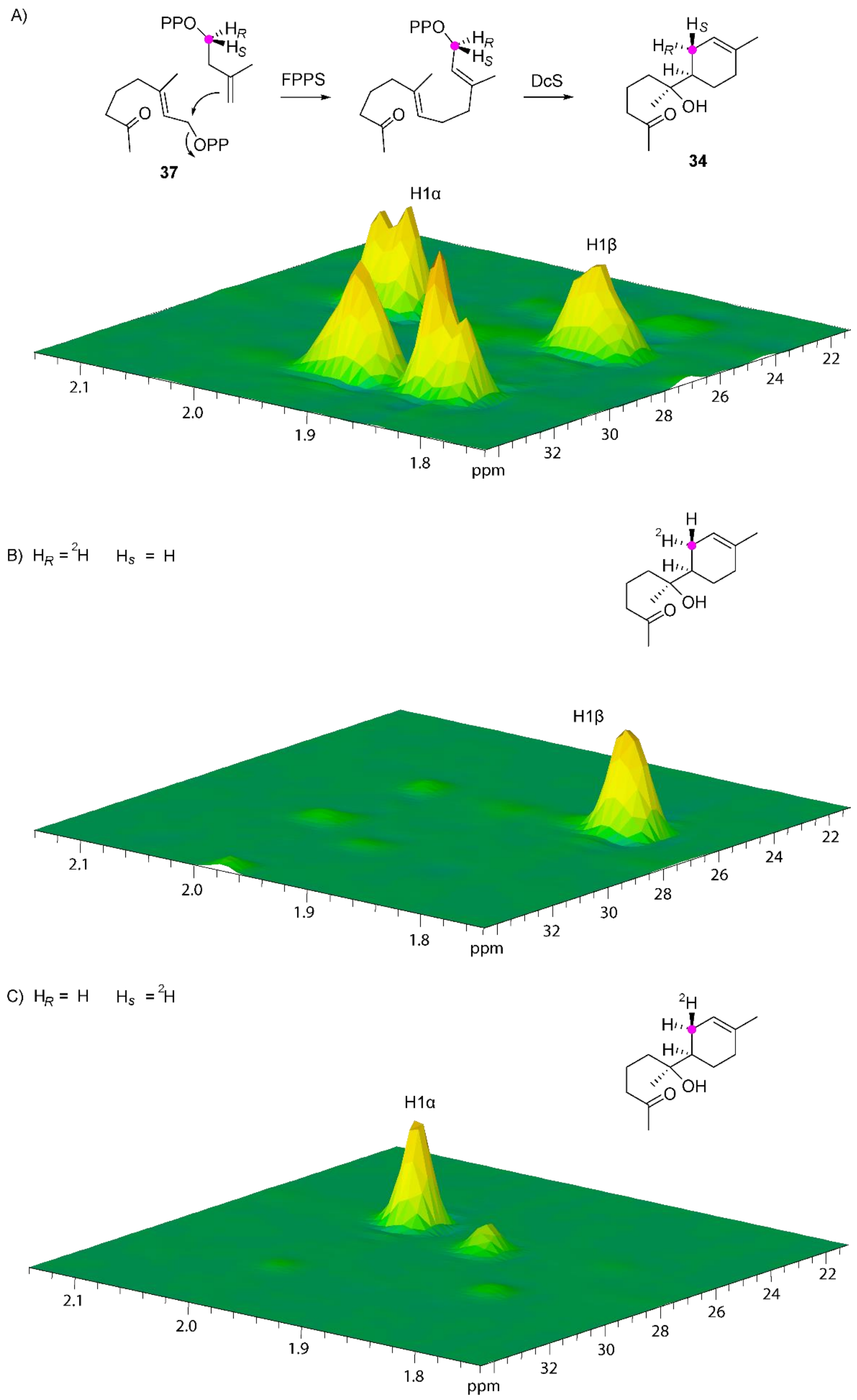

Figure S100. Absolute configuration of **34**. A) Partial HSQC spectra of purified **34**. Spectra resulting from incubation of DcS, FPPS and **37** with B) (*R*)(1-^{13}C,1-^{2}H)IPP and C) (*S*)-(1-^{13}C,1-^{2}H)IPP.

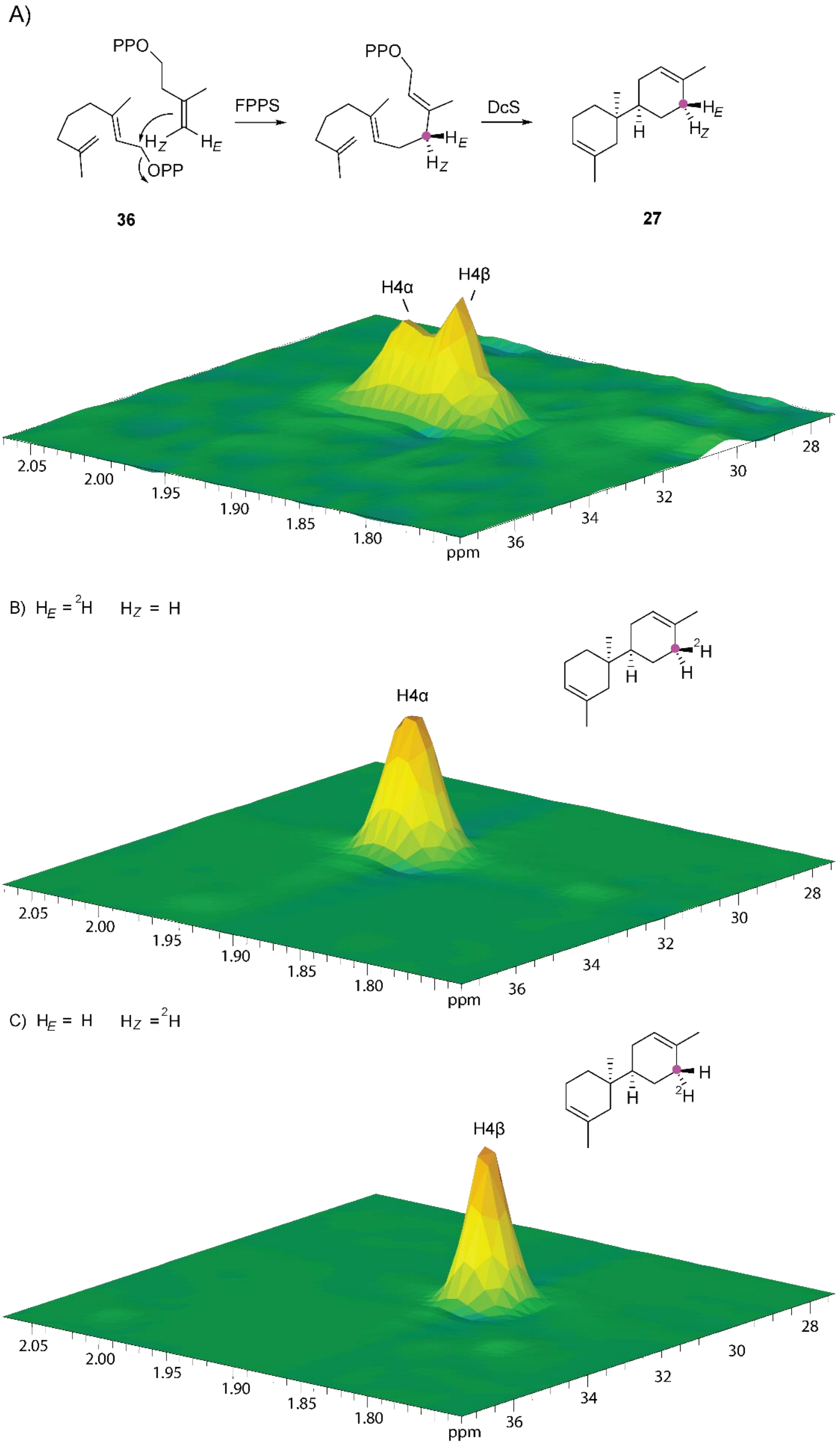

Figure S101. Absolute configuration of **27**. A) Partial HSQC spectra of purified **27**. Spectra resulting from incubation of DcS, FPPS and **36** with B) (*E*)-(4-^{13}C,4-^{2}H)IPP and C) (*Z*)-(4-^{13}C,4-^{2}H)IPP.

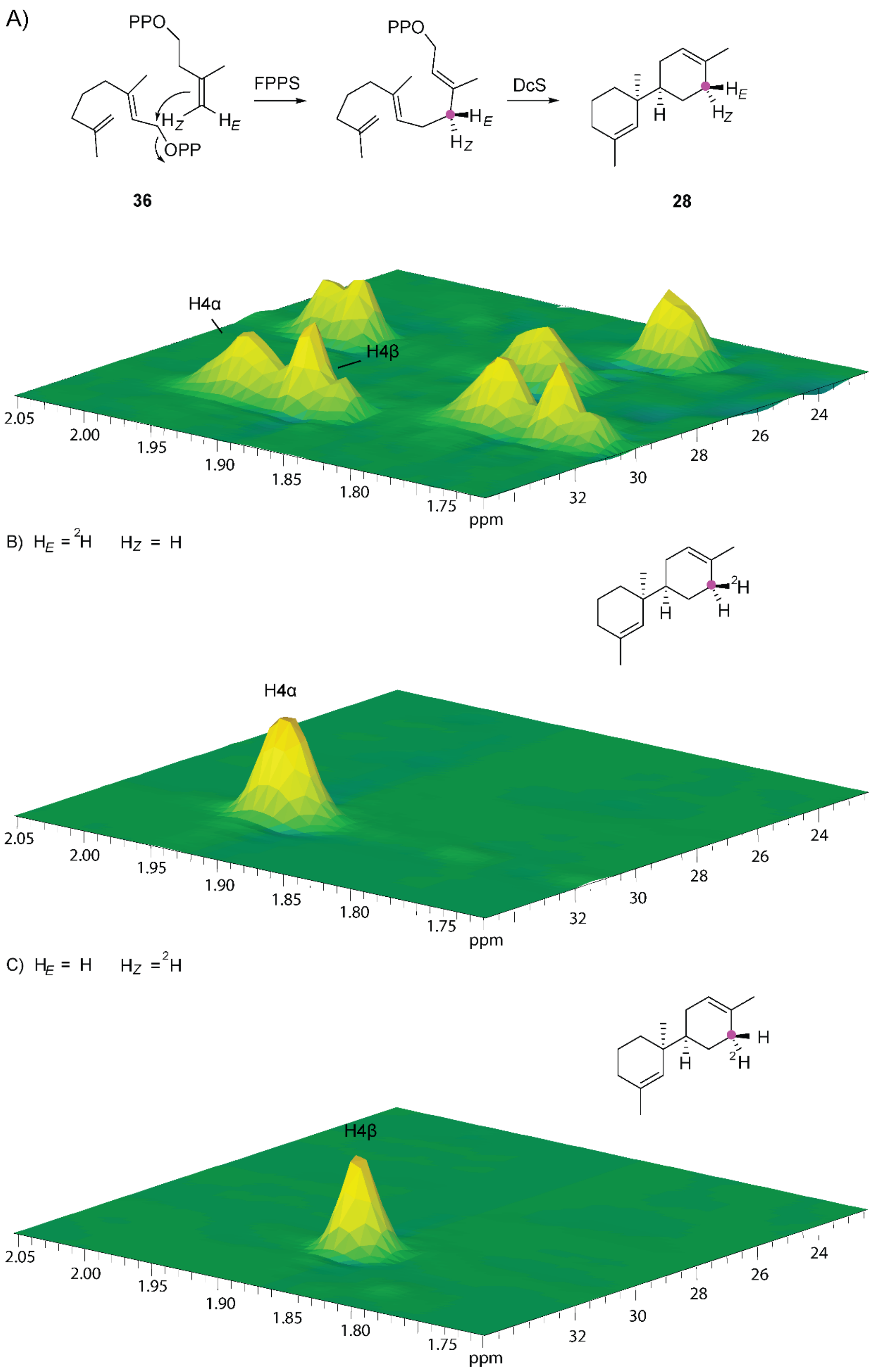

Figure S102. Absolute configuration of **28**. A) Partial HSQC spectra of purified **28**. Spectra resulting from incubation of DcS, FPPS and **36** with B) (*E*)-(4-^{13}C,4-^{2}H)IPP and C) (*Z*)-(4-^{13}C,4-^{2}H)IPP.

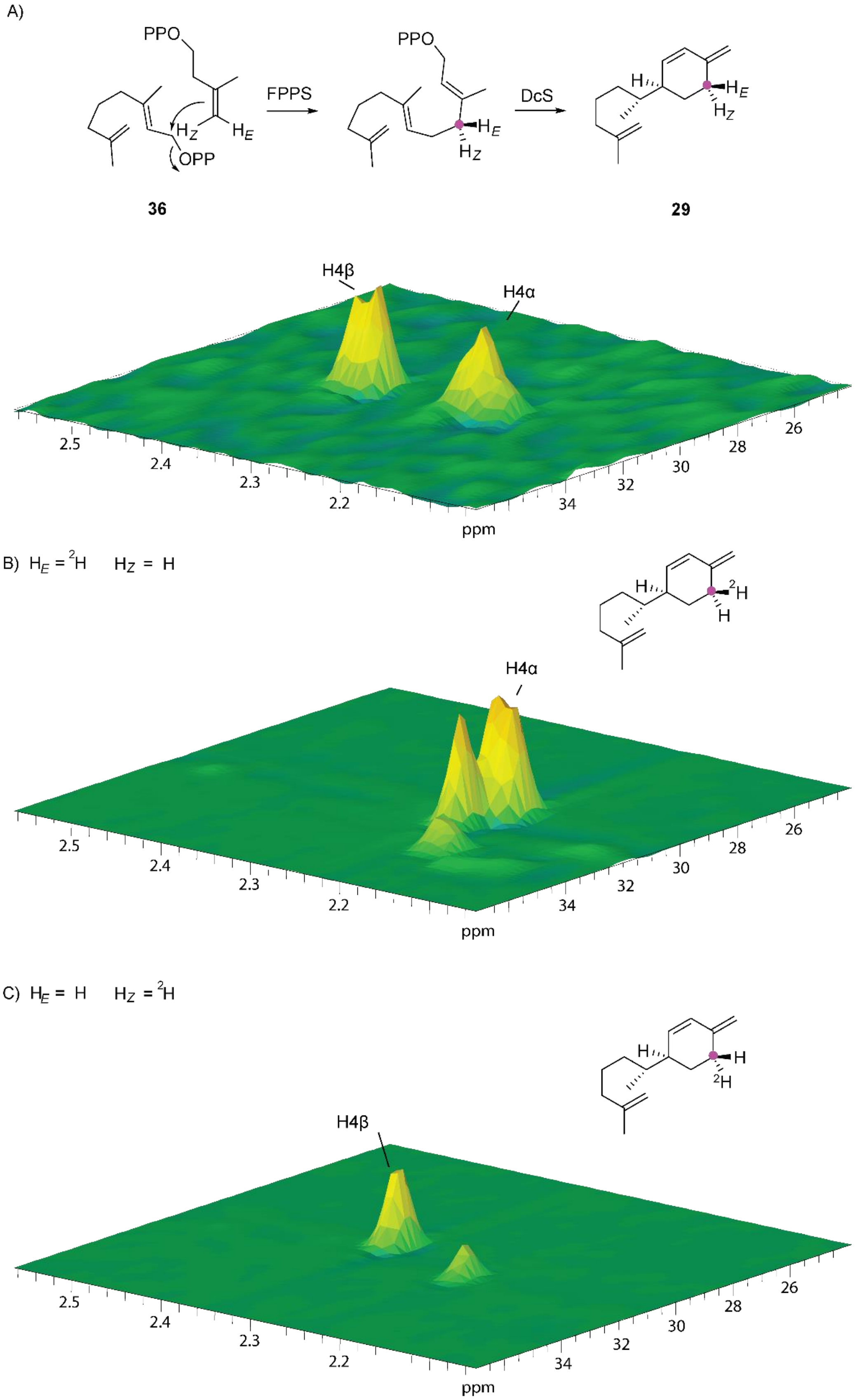

Figure S103. Absolute configuration of **29**. A) Partial HSQC spectra of purified **29**. Spectra resulting from incubation of DcS, FPPS and **36** with B) (*E*)-(4-^{13}C,4-^{2}H)IPP and C) (*Z*)-(4-^{13}C,4-^{2}H)IPP.

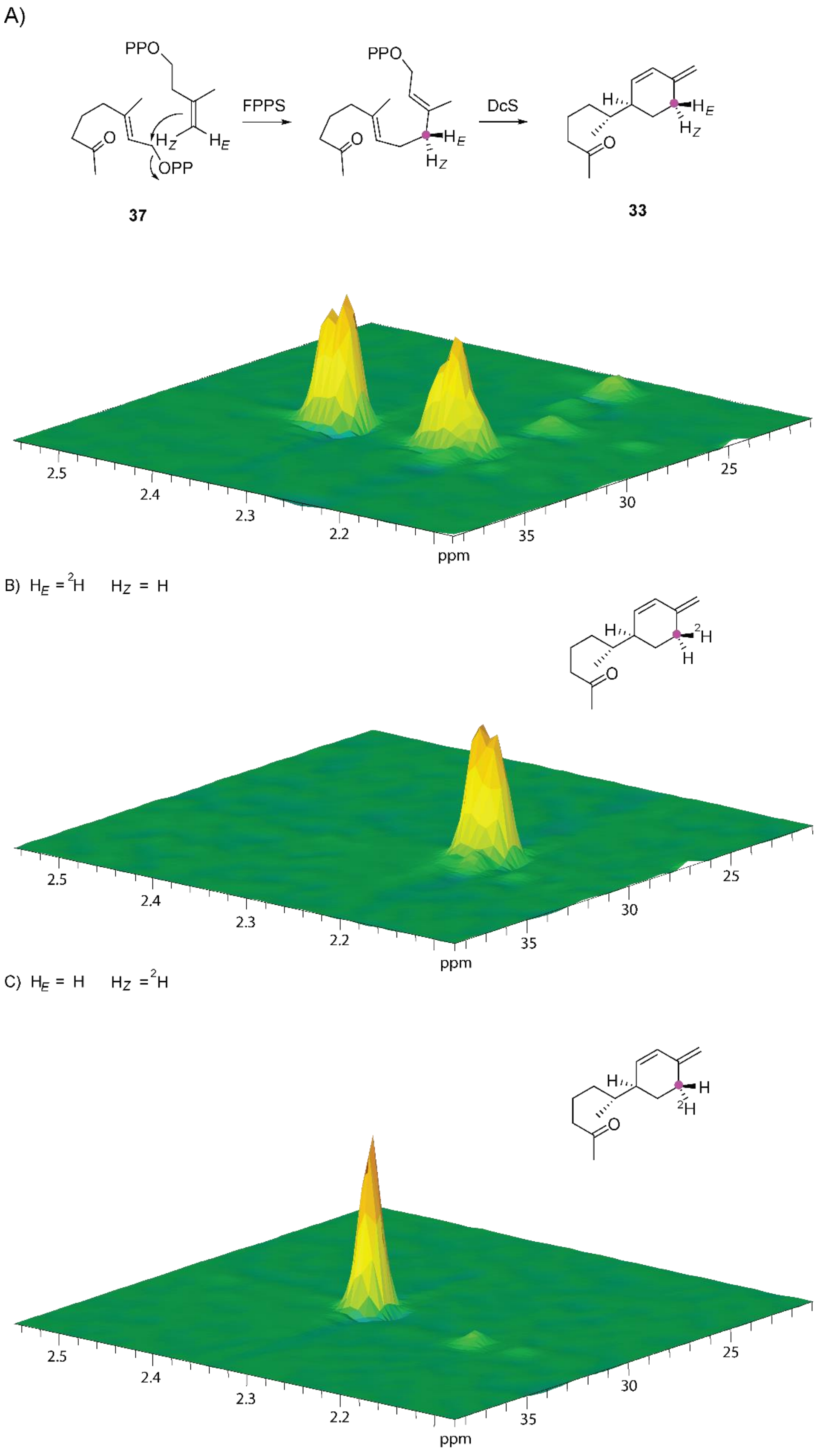

Figure S104. Absolute configuration of **33**. A) Partial HSQC spectra of purified **33**. Spectra resulting from incubation of DcS, FPPS and **37** with B) (*E*)-(4-^{13}C,4-^{2}H)IPP and C) (*Z*)-(4-^{13}C,4-^{2}H)IPP.

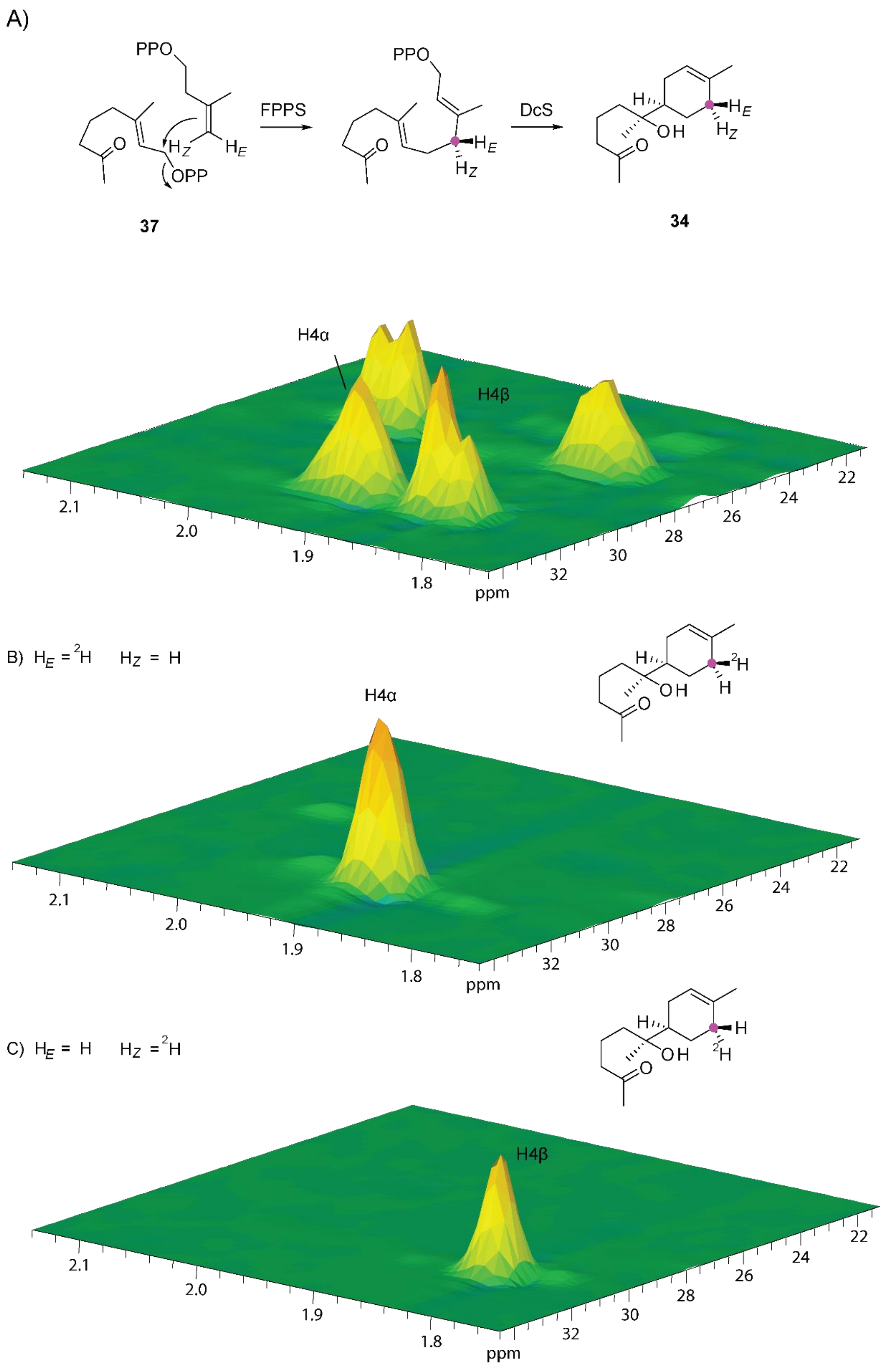

Figure S105. Absolute configuration of **34**. A) Partial HSQC spectra of purified **34**. Spectra resulting from incubation of DcS, FPPS and **37** with B) (*E*)-(4-^{13}C,4-^{2}H)IPP and C) (*Z*)-(4-^{13}C,4-^{2}H)IPP.

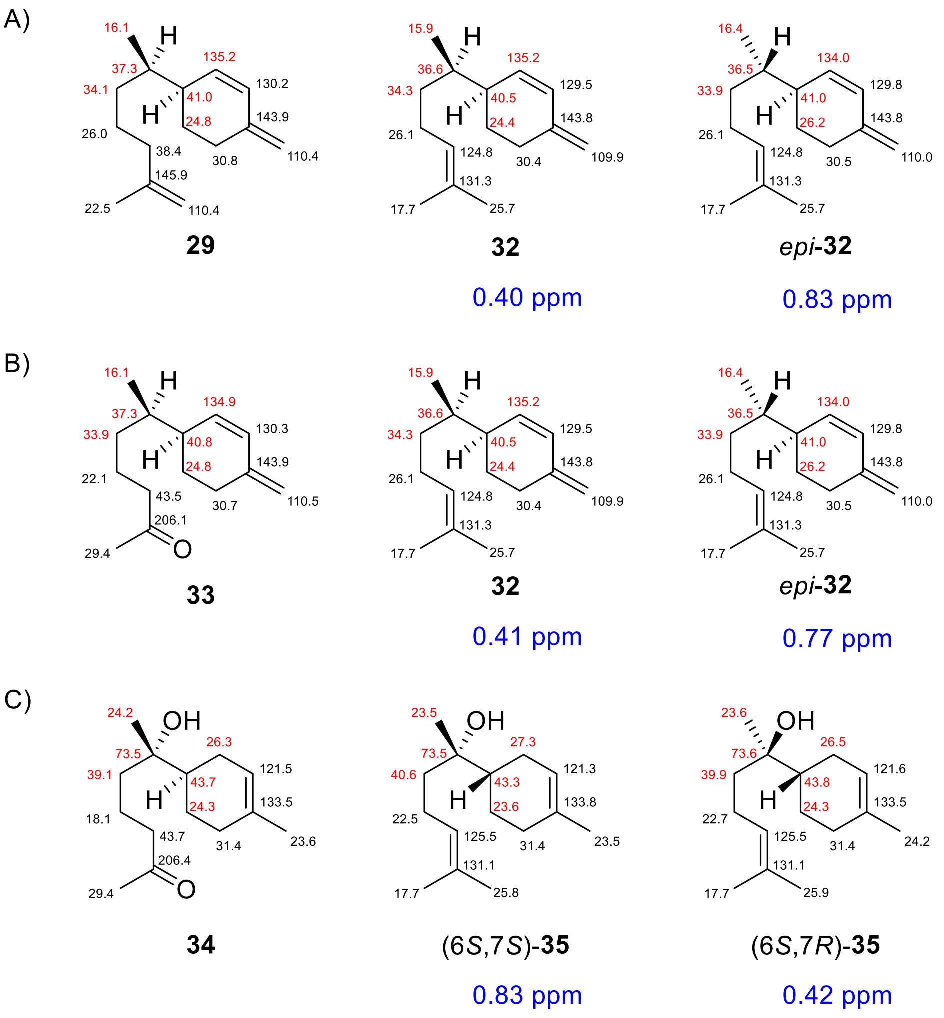

Figure S106. Comparison of ^{13}C-NMR data of A) compound **29** to the *ent*-**32** and 6-*epi*-**32**,[9] B) compound **33** to the *ent*-**32** and 6-*epi*-**32**, C) compound **34** to the (6*S*,7*S*)-**35** and (6*S*,7*R*)-**35**.[10] Data at structures indicate ^{13}C chemical shifts in ppm. Data in blue are root mean square deviations for the ^{13}C chemical shifts shown in red determined for the six carbons around the stereogenic centers at C6 and C7.

Synthetic route to (*S*)-6-hydroxy-6-((*S*)-4-methylcyclohex-3-en-1-yl)heptan-2-one ((6*S*,7*S*)-34)

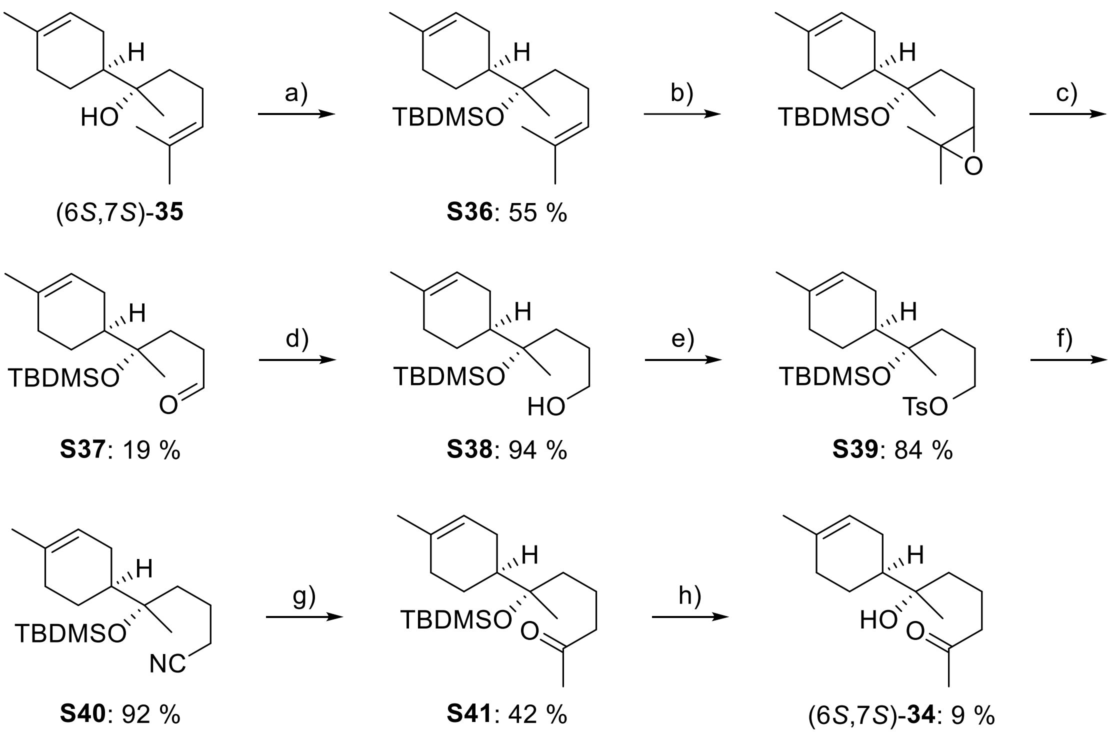

Scheme S7. Synthesis of compound (6*S*,7*S*)-**34**. a) TBDMS-OTf, Et_3N, CH_2Cl_2, 3 h; b) *m*CPBA, CH_2Cl_2, 0 °C to room temperature, overnight; c) H_5IO_6, THF/Et_2O, 0 °C, 0.5 h; d) $NaBH_4$, EtOH, 0 °C, 1 h; e) TsCl, DMAP, Et_3N, CH_2Cl_2, overnight; f) KCN, DMSO, overnight; g) MeLi, Et_2O, –78 °C to room temperature, overnight; h) TBAF, THF, 40 °C to 50 °C, overnight.

Synthesis of *tert*-butyldimethyl(((*S*)-6-methyl-2-((*S*)-4-methylcyclohex-3-en-1-yl)hept-5-en-2-yl)oxy) silane (S36)

(–)-α-Bisabolol ((6*S*,7*S*)-**35**) (2.00 g, 9.00 mmol) was dissolved in dichloromethane (40 mL) at 0 °C, followed by the addition of triethylamine (1.82 g, 18.00 mmol, 2.0 eq). To this solution, TBDMS-OTf (2.42 g, 9.18 mmol, 1.02 eq) was added dropwise and the mixture was stirred at room temperature for 3 h. The reaction was quenched by pouring into ice sold half saturated NH_4Cl solution. The product was extracted with Et_2O (3 x 80 mL), the combined organic layers were dried with $MgSO_4$ and concentrated under reduced pressure. The silane **S36** (1.67 g, 4.96 mmol, 55%) was obtained via silica gel chromatography (pentane). TLC: (pentane): R_f = 0.63. $[\alpha]_D^{20}$ = –36.6 (*c* 1.86, CH_2Cl_2). EI-MS (70 eV): *m*/*z* (%) = 321 (1), 279 (17), 253 (6), 241 (34), 227 (1), 204 (11), 185 (10), 173 (4), 161 (4), 147 (5), 133 (4), 119 (30), 109 (18), 93 (14), 75 (100), 69 (41), 55 (8), 41 (20). GC (HP5-MS): *I* = 1996. ^{1}H-NMR (C_6D_6, 500 MHz): δ = 5.47 (br s, 1H), 5.25 – 5.18 (m, 1H), 2.19 – 2.03 (m, 2H), 2.05 – 1.86 (m, 5H), 1.69 (d, *J* = 1.4 Hz, 3H), 1.65 (d, *J* = 2.2 Hz, 3H), 1.67 – 1.62 (m, 1H), 1.61 (s, 3H), 1.54 (ddd, *J* = 13.6, 12.0, 5.3 Hz, 1H), 1.44 – 1.32 (m, 1H), 1.09 (s, 3H), 1.01 (s, 9H), 0.14 (s, 3H), 0.13 (s, 3H) ppm. ^{13}C-NMR (C_6D_6, 126 MHz): δ = 133.78 (C_q), 131.03 (C_q), 125.35 (CH), 121.53 (CH), 77.67 (C_q), 42.84 (CH), 41.45 (CH_2), 31.50 (CH_2), 27.15 (CH_2), 26.34 (3 x CH_3), 25.90 (CH_3), 24.90 (CH_3), 24.07 (CH_2), 23.62 (CH_3), 23.15 (CH_2), 18.82 (C_q), 17.76 (CH_3), –1.58 (CH_3), –1.62 (CH_3) ppm.

Synthesis of (*S*)-4-((*tert*-butyldimethylsilyl)oxy)-4-((*S*)-4-methylcyclohex-3-en-1-yl) pentanal (S37)

To a dichloromethane (25 mL) solution of **S36** (1.2 g, 3.56 mmol) *m*CPBA (suspended in 5 mL CH_2Cl_2) was added dropwise at 0 °C. The mixture was stirred overnight without further cooling, followed by the addition of sat. $Na_2S_2O_3$ solution (100 mL). The mixture was extracted with Et_2O (3 x 100 mL), the combined organic layers were dried with $MgSO_4$ and concentrated under reduced pressure. The residue was purified via silica gel chromatography (cyclohexane/ethyl acetate, 30:1), the fractions of R_f = 0.30 were collected and used as crude epoxides for the next step directly.

H_5IO_6 (494 mg, 2.17 mmol, 1.2 eq) was suspended in THF/Et_2O (5.5 mL/1.2 mL) and stirred at room temperature for 10 min. The mixture was cooled to 0 °C, followed by the addition of Et_2O (4 mL). To this mixture, the crude epoxide solution (635 mg, 1.80 mmol, in 2 mL Et_2O) was added dropwise and stirred for 30 min at the same temperature. The reaction was quenched by the addition of sat. $Na_2S_2O_3$ solution (50 mL) and extracted with Et_2O (3 x 50 mL). The combined organic layers were dried with $MgSO_4$ and concentrated under reduced pressure. The aldehyde **S37** (205 mg, 0.66 mmol, 19%) was obtained via silica gel chromatography (cyclohexane/ethyl acetate, 30:1). TLC: (cyclohexane/ethyl acetate, 30:1): R_f = 0.28. Optical rotation: $[\alpha]_D^{20}$ = –46.9 (*c* 1.93, CH_2Cl_2). HRMS (ESI): *m*/*z* = 293.2295 (calc. for $[C_{18}H_{34}O_2Si– OH]^+$ = 293.2296). EI-MS (70 eV): *m*/*z* (%) = 295 (1), 253 (9), 215 (32), 201 (2), 185 (7), 159 (100), 145 (12), 131 (10), 119 (15), 105 (26), 93 (18), 75 (72), 67 (11), 55 (10), 41 (12). GC (HP5-MS): *I* = 1957. ^{1}H-NMR (C_6D_6, 500 MHz): δ = 9.41 (t, *J* = 1.5 Hz, 1H), 5.43 – 5.36 (m, 1H), 2.12 (dddd, *J* = 17.3, 10.5, 5.2, 1.4 Hz, 1H), 2.00 (dddd, *J* = 17.5, 10.4, 5.7, 1.5 Hz, 1H), 1.95 – 1.84 (m, 2H), 1.86 – 1.68 (m, 2H), 1.64 (br s, 3H), 1.70 – 1.54 (m, 2H), 1.39 (tdd, *J* = 11.1, 5.2, 2.3 Hz, 1H), 1.21 (qd, *J* = 12.1, 5.7 Hz, 1H), 0.94 (s, 9H), 0.92 (s, 3H), 0.06 (s, 3H), 0.05 (s, 3H) ppm. ^{13}C-NMR (C_6D_6, 126 MHz): δ = 200.45 (CH), 133.81 (C_q), 121.19 (CH), 77.06 (C_q), 43.18 (CH), 39.02 (CH_2), 32.34 (CH_2), 31.41 (CH_2), 27.04 (CH_2), 26.28 (3 x CH_3), 24.37 (CH_3), 24.03 (CH_2), 23.56 (CH_3), 18.73 (C_q), –1.71 (CH_3), –1.77 (CH_3) ppm.

Synthesis of (*S*)-4-((*tert*-butyldimethylsilyl)oxy)-4-((*S*)-4-methylcyclohex-3-en-1-yl) pentan-1-ol (S38)

Compound **S37** (205 mg, 0.66 mmol) was dissolved in EtOH (8 mL) and the solution was cooled to 0 °C. $NaBH_4$ (50 mg, 1.32 mmol, 2.0 eq) was added and the solution was stirred at 0 °C for 1 h. The reaction was quenched by pouring the mixture into ice-water (100 mL) and the aqueous phase was extracted with Et_2O (3 x 50 mL). The combined organic layers were dried with $MgSO_4$ and concentrated under reduced pressure. Purification via flash chromatography (cyclohexane/ethyl acetate, 4:1) provided compound **S38** (194 mg, 0.62 mmol, 94%) as a colorless oil. TLC (cyclohexane/ethyl acetate, 4:1): R_f = 0.38. Optical rotation: $[\alpha]_D^{20}$ = –51.48 (*c* 0.27, CH_2Cl_2). HRMS (EI): *m*/*z* = 297.2245 (calc. for $[C_{18}H_{36}O_2Si– CH_3]^+$ = 297.2245). EI-MS (70 eV): *m*/*z* (%) = 253 (12), 217 (13), 180 (11), 163 (19), 145 (16), 132 (4), 119 (60), 107 (17), 93 (20), 85 (58), 75 (100), 67 (12), 55 (9), 43 (17). GC (HP5-MS): *I* = 2016. ^{1}H-NMR (C_6D_6, 500 MHz): δ = 5.49 – 5.44 (br s, 1H), 3.32 (q, *J* = 5.6 Hz, 2H), 2.02 – 1.82 (m, 6H), 1.66 (s, 3H), 1.61 – 1.42 (m, 4H), 1.41 – 1.29 (m, 1H), 1.05 (s, 3H), 1.00 (s, 9H), 0.53 (t, *J* = 5.1 Hz, 1H), 0.13 (s, 3H), 0.12 (s, 3H) ppm. ^{13}C-NMR (C_6D_6, 126 MHz): δ = 133.77 (C_q), 121.49 (CH), 77.57 (C_q), 63.22 (CH_2), 42.82 (CH), 37.51 (CH_2), 31.48 (CH_2), 27.59 (CH_2), 27.08 (CH_2), 26.34 (3 x CH_3), 24.93 (CH_3), 23.98 (CH_2), 23.61 (CH_3), 18.80 (C_q), –1.59 (CH_3), –1.64 (CH_3) ppm.

Synthesis of (*S*)-4-((*tert*-butyldimethylsilyl)oxy)-4-((*S*)-4-methylcyclohex-3-en-1-yl)pentyl 4-methyl benzenesulfonate (S39)

The alcohol **S38** (194 mg, 0.62 mmol), Et_3N (150 mg, 1.49 mmol, 2.4 eq) and DMAP (38 mg, 0.31 mmol, 0.5 eq) were dissolved in CH_2Cl_2 (5 mL), and the solution was cooled to 0 °C. TsCl (suspended in 3 mL CH_2Cl_2) was added dropwise to the reaction solution. The mixture was stirred at room temperature for 2 h, followed by adding Et_2O (50 mL) and H_2O (50 mL),. The aqueous phase was extracted with Et_2O (2 x 50 mL). The organic layers were combined and dried with $MgSO_4$ and evaporated under reduced pressure. The product **S39** (242 mg, 0.52 mmol, 84%) was purified by silica gel chromatography (cyclohexane/ethyl acetate, 10:1). TLC (cyclohexane/ethyl acetate, 10:1): R_f = 0.39. Optical rotation: $[\alpha]_D^{20}$ = –35.86 (*c* 0.29, CH_2Cl_2). HRMS (EI): *m*/*z* = 451.2335 (calc. for $[C_{25}H_{42}O_4SSi – CH_3]^+$ = 451.2333). ^{1}H-NMR (C_6D_6, 700 MHz): δ = 7.82 – 7.76 (m, 2H), 6.71 (d, *J* = 7.8 Hz, 2H), 5.42 – 5.38 (m, 1H), 3.92 – 3.84 (m, 2H), 1.94 – 1.85 (m, 2H), 1.84 (s, 3H), 1.83 – 1.68 (m, 3H), 1.64 (s, 3H), 1.57 – 1.48 (m, 1H), 1.45 – 1.36 (m, 2H), 1.37 – 1.27 (m, 2H), 1.21 (qd, *J* = 12.2, 5.5 Hz, 1H), 0.94 (s, 9H), 0.91 (s, 3H), 0.06 (s, 3H), 0.03 (s, 3H) ppm. ^{13}C-NMR (C_6D_6, 176 MHz): δ = 144.11 (C_q), 134.72 (C_q), 133.74 (C_q), 129.82 (2 x CH), 128.18 (2 x CH), 121.28 (CH), 77.21 (C_q), 70.95 (CH_2), 42.94 (CH), 36.78 (CH_2), 31.40 (CH_2), 27.00 (CH_2), 26.27 (3 x CH_3), 24.48 (CH_3), 23.95 (CH_2), 23.84 (CH_2), 23.57 (CH_3), 21.13 (CH_3), 18.72 (C_q), –1.70 (CH_3), –1.76 (CH_3) ppm.

Synthesis of (*S*)-5-((*tert*-butyldimethylsilyl)oxy)-5-((*S*)-4-methylcyclohex-3-en-1-yl) hexanenitrile (S40)

To a DMSO (5 mL) solution of compound **S39** (242 mg, 0.52 mmol) was added KCN (68 mg, 1.04 mmol, 2.0 eq). The mixture was stirred at room temperature overnight, followed by the addition of H_2O (50 mL). The product was extracted with Et_2O (3 x 50 mL) and dried with $MgSO_4$. The solvent was removed under reduced pressure and the nitrile **S40** (154 mg, 0.48 mmol, 92%) was obtained via silica gel chromatography (cyclohexane/ethyl acetate, 10:1). TLC (cyclohexane/ethyl acetate, 10:1): R_f = 0.46. Optical rotation: $[\alpha]_D^{20}$ = –51.3 (*c* 0.23, CH_2Cl_2). HRMS (EI): *m*/*z* = 306.2247 (calc. for $[C_{19}H_{35}NOSi – CH_3]^+$ = 306.2248). EI-MS (70 eV): *m*/*z* (%) = 306 (4), 264 (64), 253 (5), 226 (38), 212 (1), 189 (4), 170 (8), 159 (2), 146 (8), 128 (15), 121 (13), 115 (16), 105 (4), 93 (11), 75 (100), 67 (9), 59 (8), 53 (4), 47 (4), 41 (11). GC (HP5-MS): *I* = 2124. ^{1}H-NMR (C_6D_6, 700 MHz): δ = 5.43 – 5.40 (m, 1H), 1.96 – 1.85 (m, 2H), 1.84 – 1.69 (m, 3H), 1.65 (s, 3H), 1.42 – 1.36 (m, 3H), 1.33 – 1.20 (m, 4H), 1.15 – 1.07 (m, 1H), 0.96 (s, 9H), 0.91 (s, 3H), 0.08 (s, 6H) ppm. ^{13}C-NMR (C_6D_6, 176 MHz): δ = 133.86 (C_q), 121.16 (CH), 119.27 (C_q), 77.12 (C_q), 42.91 (CH), 39.94 (CH_2), 31.38 (CH_2), 27.01 (CH_2), 26.24 (3 x CH_3), 24.44 (CH_3), 23.93 (CH_2), 23.56 (CH_3), 20.10 (CH_2), 18.72 (C_q), 17.14 (CH_2), –1.69 (CH_3), –1.72 (CH_3) ppm.

Synthesis of (*S*)-6-((*tert*-butyldimethylsilyl)oxy)-6-((*S*)-4-methylcyclohex-3-en-1-yl) heptan-2-one (S41)

To a Et_2O (4 mL) solution of **S40** (154 mg, 0.28 mmol) at –78 °C MeLi (1.6 M in Et_2O, 1.05 mL, 1.68 mmol, 3.5 eq) was added dropwise. The mixture was stirred overnight without further cooling, and then was quenched by pouring into ice-cold NH_4Cl solution (20 mL sat. NH_4Cl with 100 mL ice-water). The aqueous layer was extracted with Et_2O (3 x 50 mL) and the combined organic extracts were dried with $MgSO_4$. The solvent was removed under reduced pressure and compound **S41** (69 mg, 0.20 mmol, 42%) was obtained via silica gel chromatography (cyclohexane/ethyl acetate, 10:1) as a colorless oil. TLC (cyclohexane/ethyl acetate, 10:1): R_f = 0.46. Optical rotation: $[\alpha]_D^{20}$ = –36.94 (*c* 0.36, CH_2Cl_2). HRMS (APCI): *m*/*z* = 339.2714 (calc. for $[C_{20}H_{38}O_2Si + H]^+$ = 339.2714). EI-MS (70 eV): *m*/*z* (%) = 323 (1), 281 (11), 253 (13), 243 (38), 225 (3), 206 (3), 185 (36), 173 (5), 159 (5), 145 (91), 132 (13), 119 (24), 105 (14), 93 (35), 75 (100), 67 (15), 55 (16), 43 (29). GC (HP5-MS): *I* = 2104. ^{1}H-

NMR (C_6D_6, 500 MHz): δ = 5.51 – 5.45 (m, 1H), 2.05 – 1.95 (m, 2H), 1.94 – 1.86 (m, 5H), 1.66 (m, 6H), 1.63 – 1.46 (m, 3H), 1.46 – 1.31 (m, 3H), 1.07 (s, 3H), 1.01 (s, 9H), 0.15 (s, 3H), 0.13 (s, 3H) ppm. ^{13}C-NMR (C_6D_6, 126 MHz): δ = 206.00 (C_q), 133.75 (C_q), 121.50 (CH), 77.65 (C_q), 43.71 (CH_2), 42.73 (CH), 40.88 (CH_2), 31.45 (CH_2), 29.37 (CH_3), 27.03 (CH_2), 26.32 (3 x CH_3), 24.90 (CH_3), 24.00 (CH_2), 23.61 (CH_3), 18.80 (C_q), 18.51 (CH_2), –1.59 (CH_3), –1.63 (CH_3) ppm.

Synthesis of (*S*)-6-hydroxy-6-((*S*)-4-methylcyclohex-3-en-1-yl)heptan-2-one ((6*S*,7*S*)-34)
S41 (69 mg, 0.20 mmol) was dissolved in THF (2 mL) and TBAF (1 M in THF, 0.64 mL, 0.64 mmol, 3.2 eq) was added dropwise. The mixture was stirred at 40 °C overnight and at 50 °C for 3 h. The reaction was quenched by adding ice-water (100 mL), and the aqueous phase was extracted with Et_2O (3 x 40 mL). The combined organic layers were dried with $MgSO_4$ and concentrated under reduced pressure. The product (6*S*,7*S*)-**34** (3.9 mg, 0.017 mmol, 9%) was purified via silica gel chromatography (diethyl ether). TLC (diethyl ether): R_f = 0.49. Optical rotation: $[\alpha]_D^{20}$ = –56.41 (*c* 0.39, CH_2Cl_2). HRMS (APCI): *m*/*z* = 225.1849 (calc. for $[C_{14}H_{24}O_2 + H]^+$ = 225.1850). EI-MS (70 eV): *m*/*z* (%) = 206 (15), 188 (10), 173 (18), 159 (3), 145 (15), 132 (48), 121 (100), 111 (25), 105 (22), 93 (73), 79 (42), 67 (43), 55 (29), 43 (74). GC (HP5-MS): *I* = 1512. ^{1}H-NMR (C_6D_6, 700 MHz): δ = 5.44 – 5.40 (m, 1H), 1.98 – 1.92 (m, 2H), 1.91 (t, *J* = 7.1 Hz, 2H), 1.90 – 1.83 (m, 2H), 1.77 – 1.71 (m, 1H), 1.67 – 1.62 (m, 6H), 1.59 – 1.51 (m, 2H), 1.51 – 1.43 (m, 1H), 1.29 – 1.17 (m, 3H), 0.94 (s, 3H), 0.51 (br s, 1H) ppm. ^{13}C-NMR (C_6D_6, 176 MHz): δ = 206.53 (C_q), 133.82 (C_q), 121.29 (CH), 73.46 (C_q), 43.67 (CH_2), 43.32 (CH), 39.85 (CH_2), 31.38 (CH_2), 29.37 (CH_3), 27.27 (CH_2), 23.64 (CH_2), 23.62 (CH_3), 23.50 (CH_3), 17.85 (CH_2) ppm. The NMR data are also given in Figures S107 – S113.

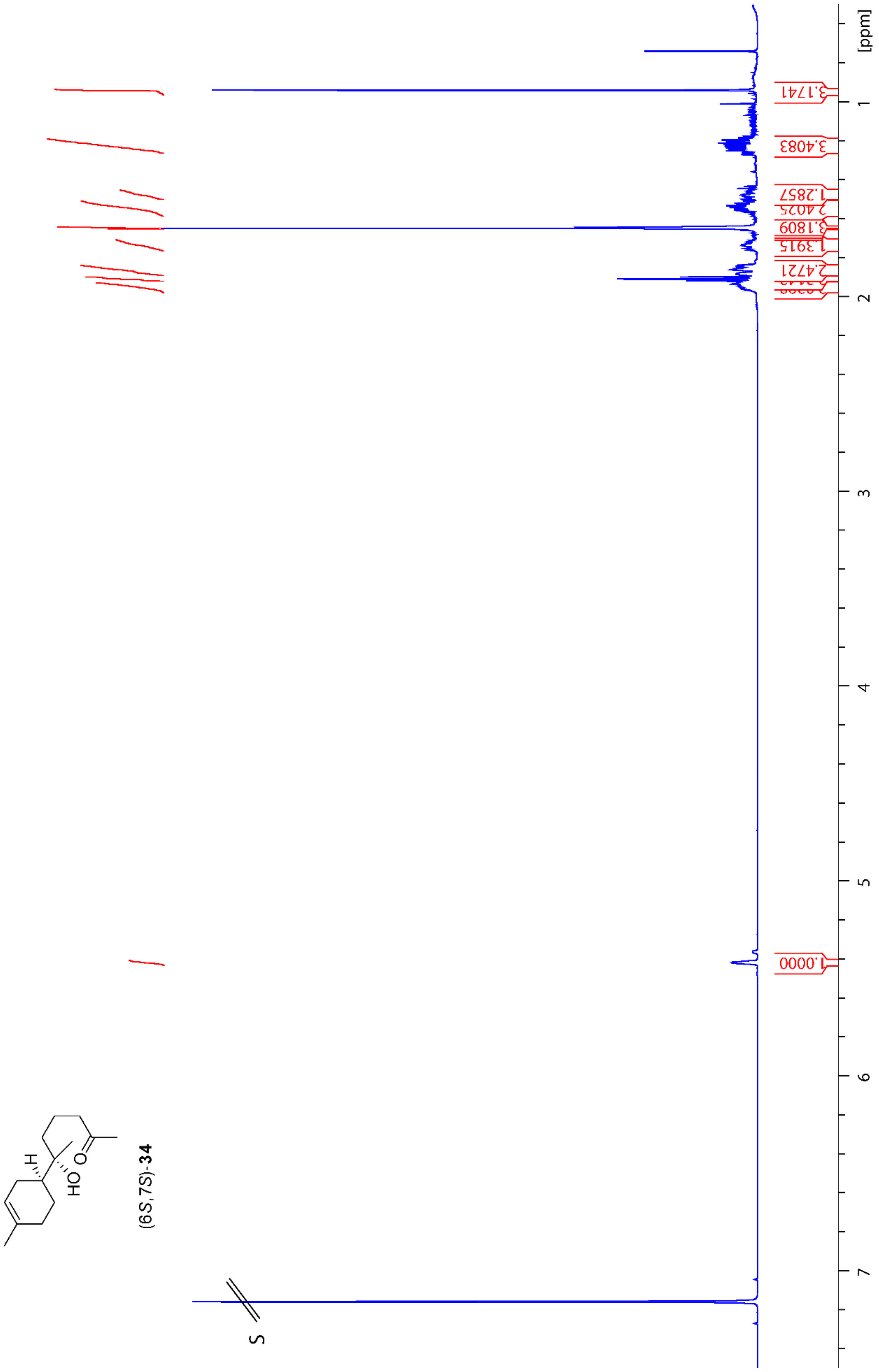

Figure S107. ^{1}H-NMR spectrum (C_6D_6, 700 MHz) of (6*S*,7*S*)-**34**. S indicates solvent peak.

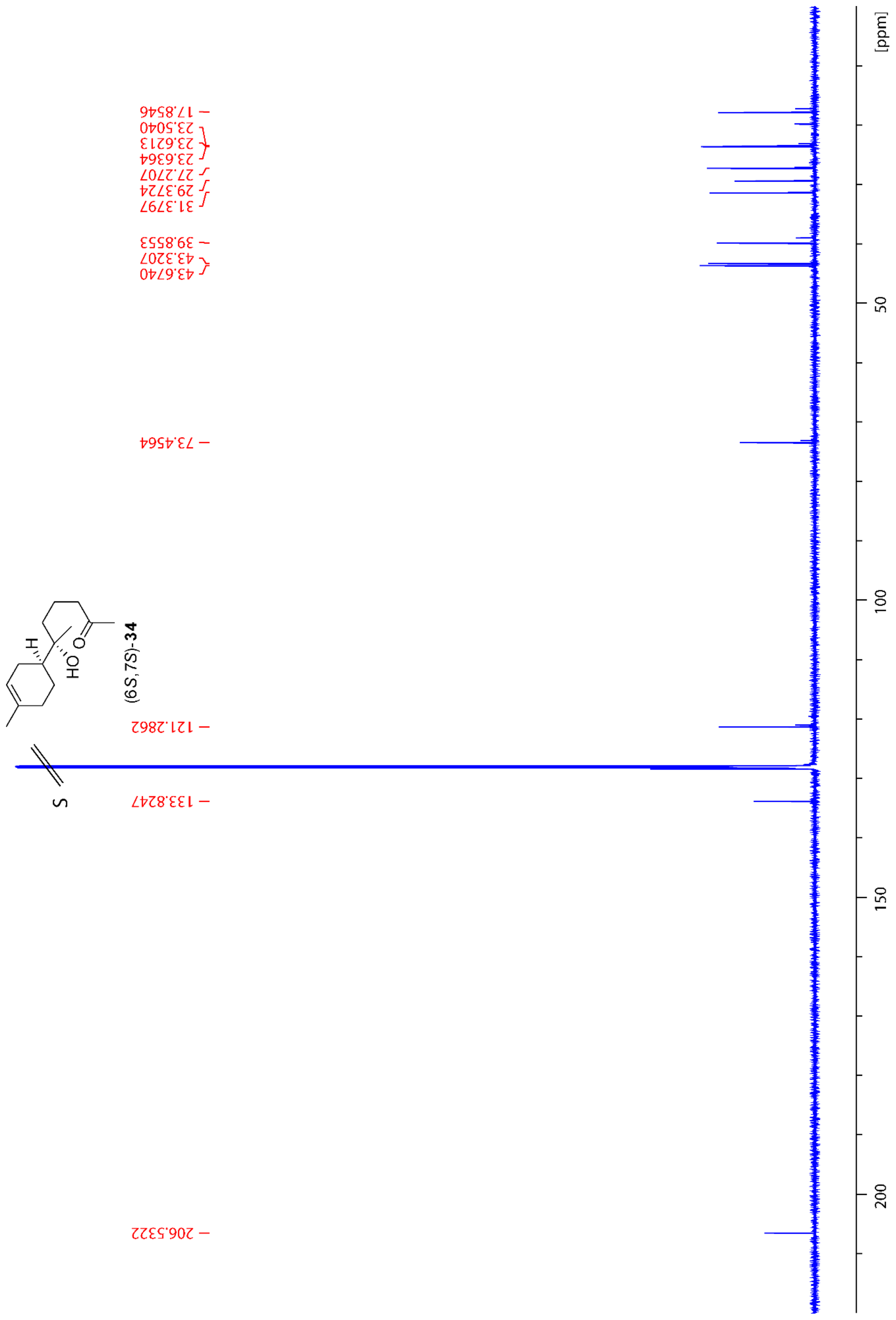

Figure S108. ^{13}C-NMR spectrum (C_6D_6, 175 MHz) of (6*S*,7*S*)-**34**. S indicates solvent peak.

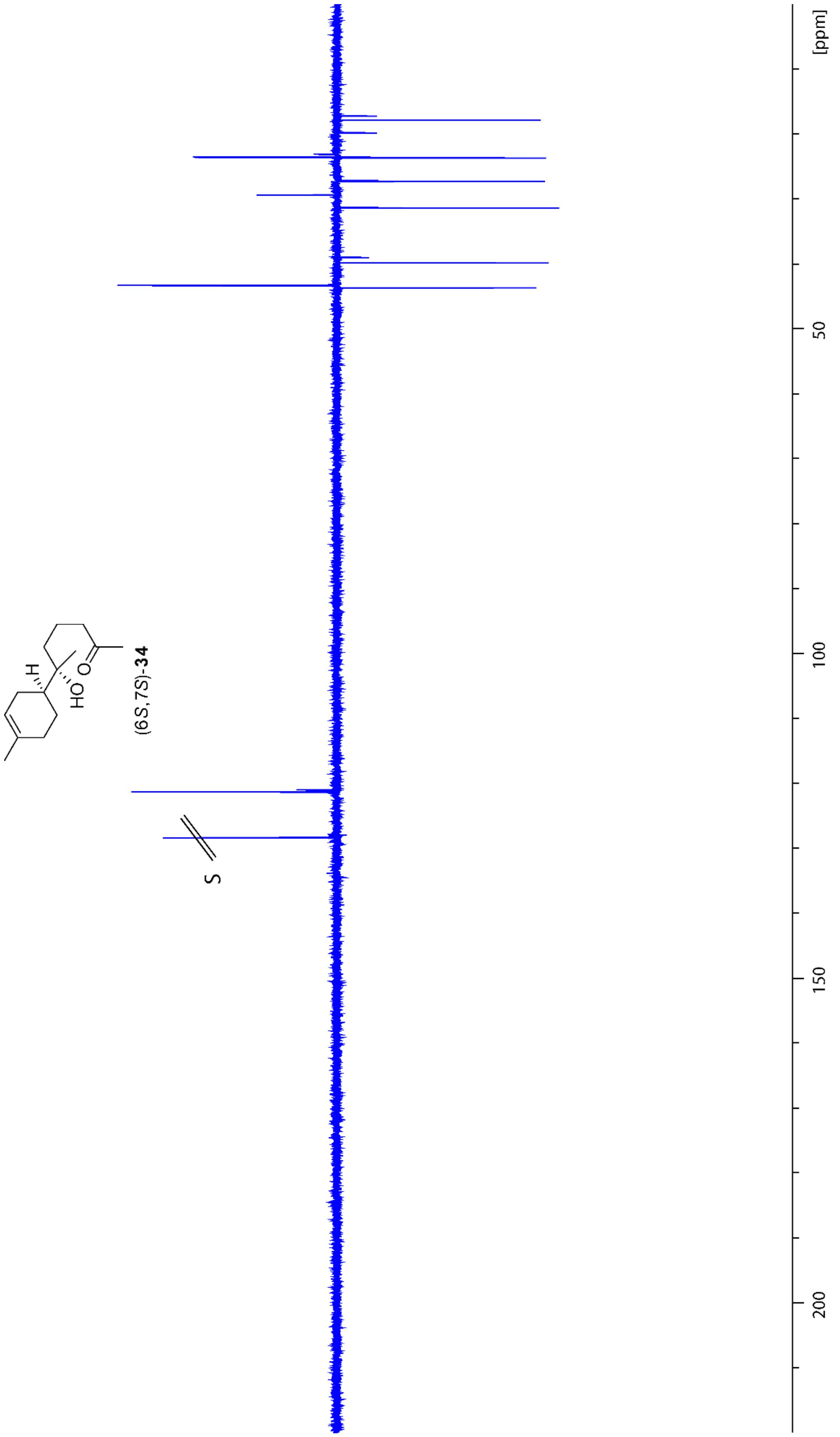

Figure S109. ^{13}C-DEPT135 spectrum (C_6D_6, 175 MHz) of (6*S*,7*S*)-**34**. S indicates solvent peak.

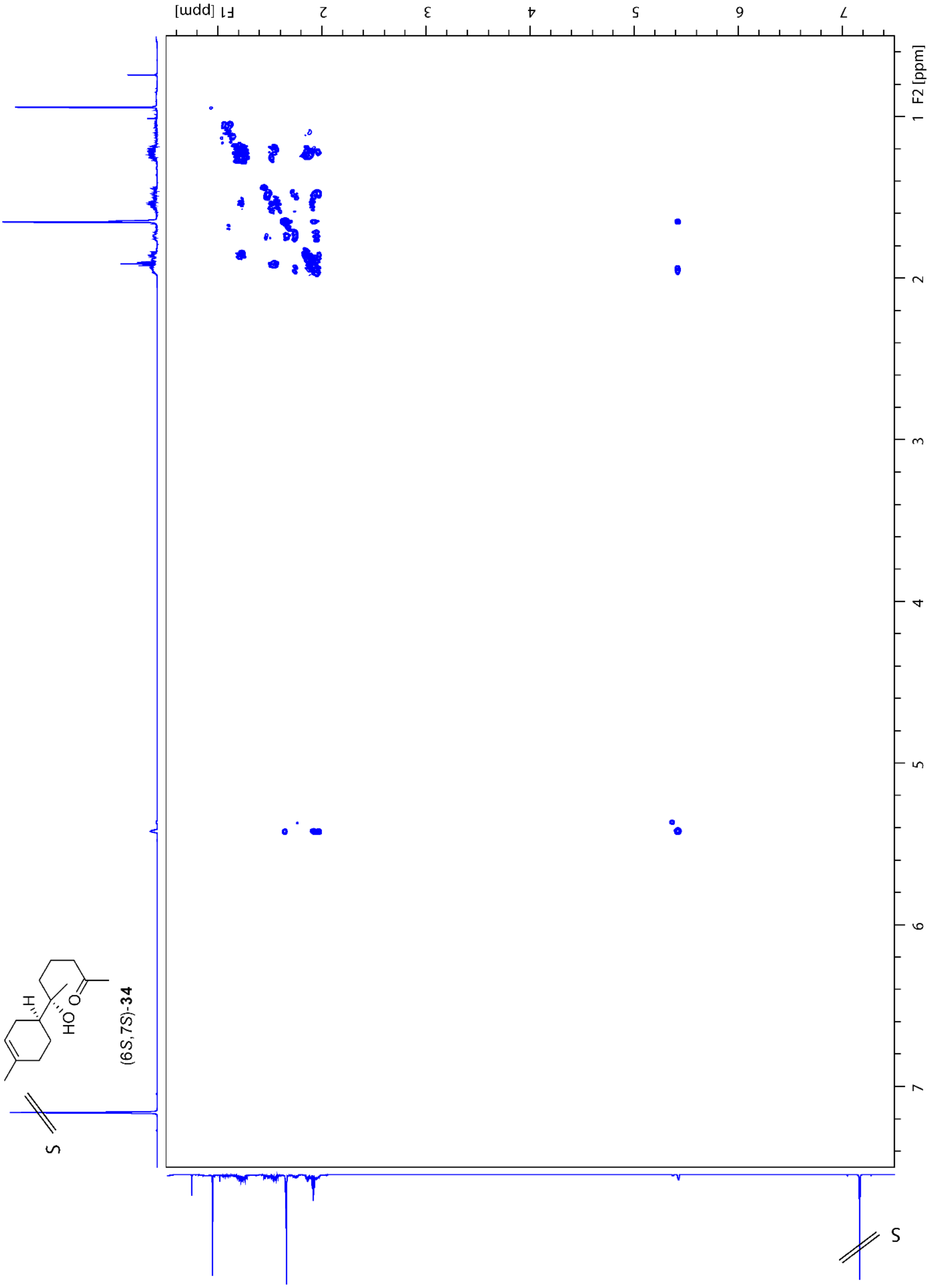

Figure S110. ^1H,^1H-COSY spectrum (C_6D_6) of (6*S*,7*S*)-**34**. S indicates solvent peaks.

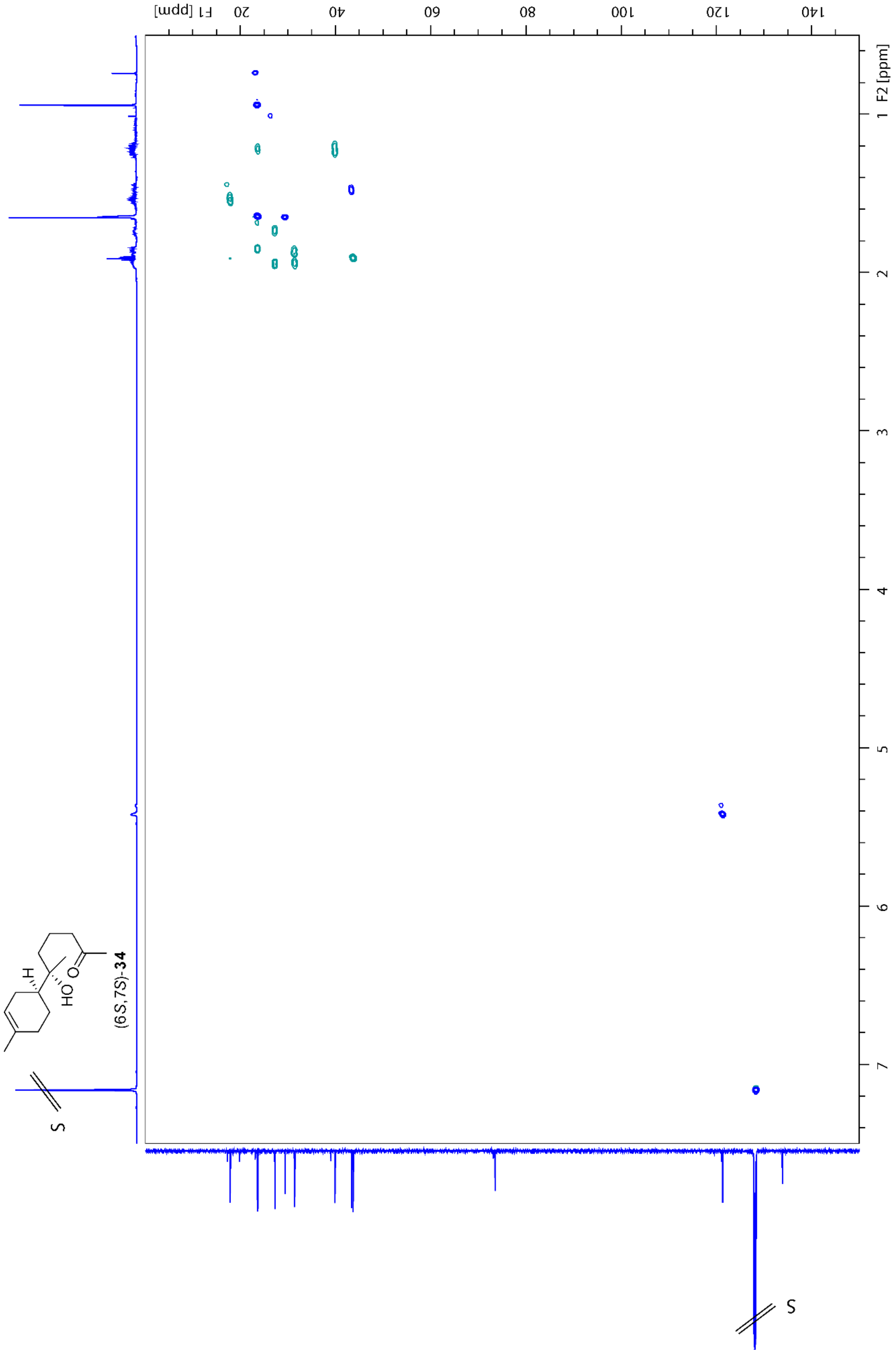

Figure S111. HSQC spectrum (C_6D_6) of (6*S*,7*S*)-**34**. S indicates solvent peaks.

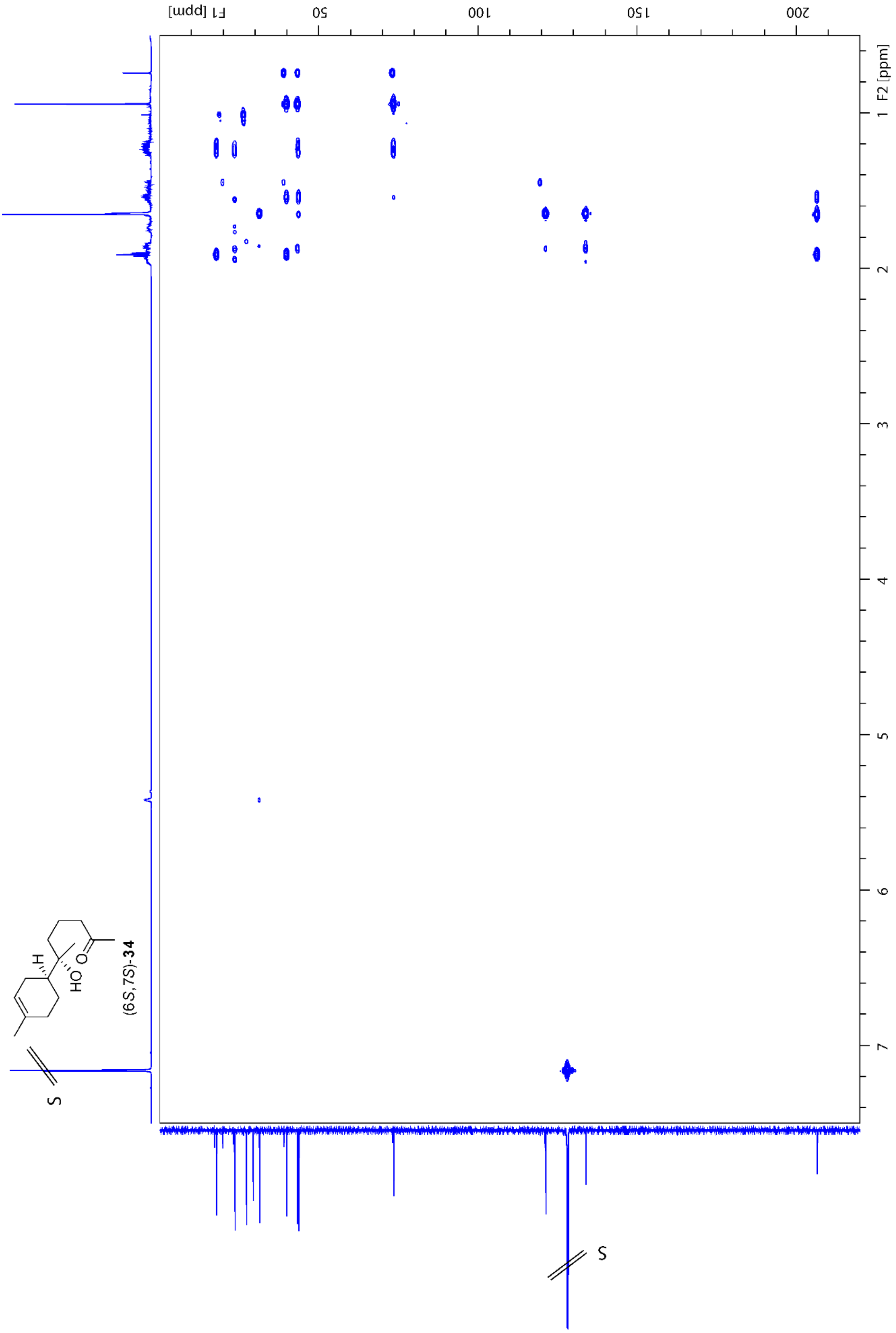

Figure S112. HMBC spectrum (C_6D_6) of (6*S*,7*S*)-**34**. S indicates solvent peaks.

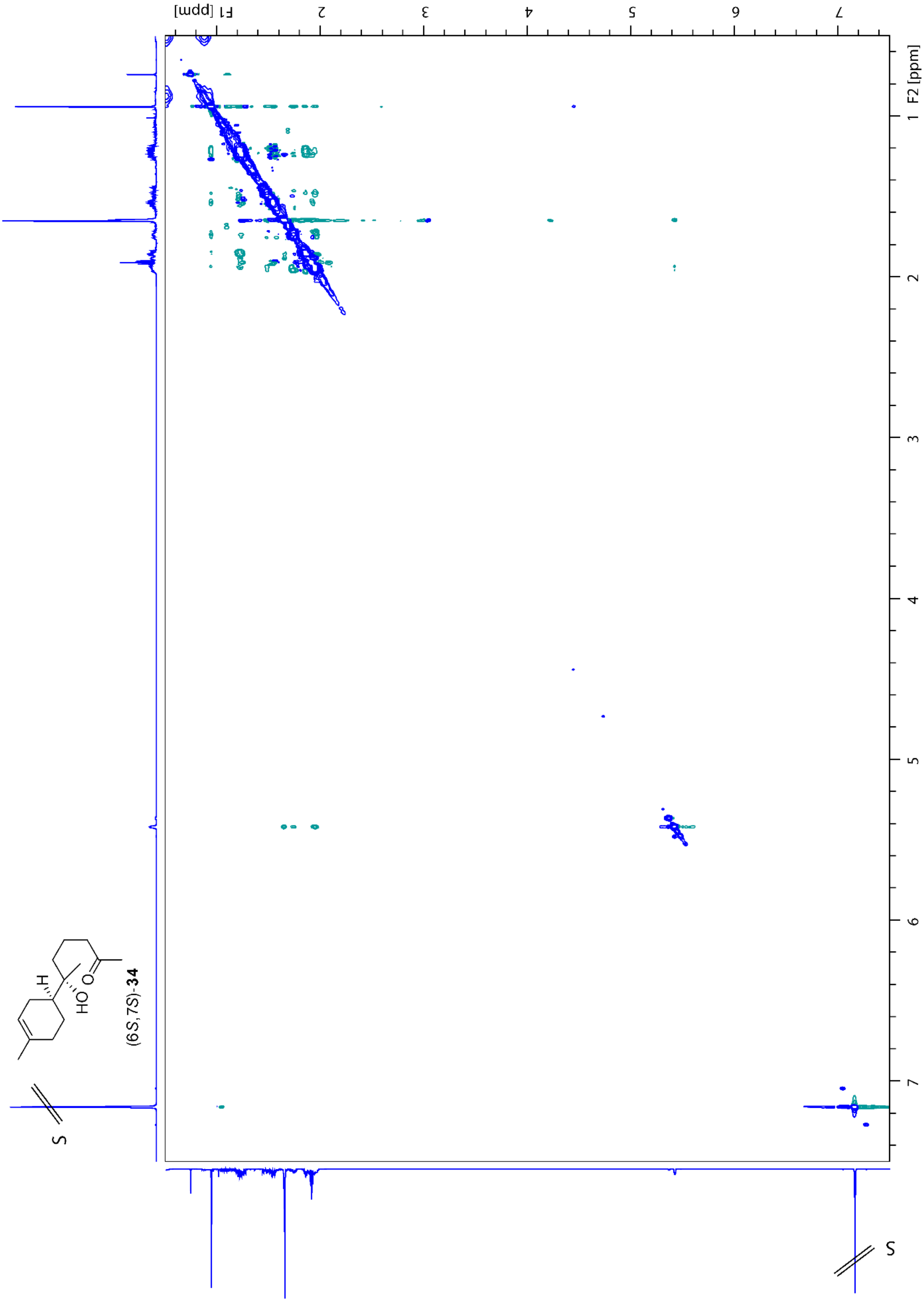

Figure S113. NOESY spectrum (C_6D_6) of (6*S*,7*S*)-**34**. S indicates solvent peaks.

```
MTVRAVELPQLWMPYPLRVNPYLSALREESETWAREMGMLGGEGPSARGAIWTRSQFHAMTV
ELLTAWTLPDASLAGLRLNHRFNIWALAWDDYFASTFKQTGDLPGALDFTARLHAFLRPEAD
ARSPEPVNAVERGIADLQKHLFPPRLAHWRQEFNLALAHYIDAGVQELTNSRGGRVPHLIEY
APFRRESFAAHTAPYSVELATGARIPEQLRHTRTVHALLDAFMDVMGLANDVASYEREVHEE
RDVNNLVVVIGTSLGITLHQAVPAALHMVNARMRDFEHLRHHELPPLVDRFGLRPDEREELE
TWLGGAASFLSGLHAWYTGAPRYAPTDHSVPEQRQRGGSEGAADRHAPAASSLARGAEAQTC
GPTASAGSA
```
Figure S114. Amino acid sequence of DcS. Conserved motifs are marked in yellow; aromatic residues addressed by site directed mutagenesis are marked in green.

Site-directed mutagenesis on dauc-8-en-11-ol synthase

The exchange of specific amino acids in DcS was achieved via polymerase-chain-reaction with site-specific primers (Table S10) carrying exchanged nucleotides compared to wild type DcS. Single mutants were created from wild type Dc, double mutants from single mutants and the triple mutant from a double mutant. Mutations were introduced in the same order they are stated in the name of the recombinant protein. Primer DcS-fw was combined with H58F-rev, H197F-rev or Y241F, and Primer DcS-rev was combined with H58F-fw, H197F-fw and Y241F-fw. Primers were used for the reactions with a final concentration being 2 µM and were mixed with substrate DNA (100 ng), Q5 reaction buffer (10 µL), GC enhancer (10 µL), dNTPS (2 µM, 5 µL), Q5-High-Fidelity DNA polymerase (0.5 µL, New England Biolabs, Ipswich, MA, USA) and sdH_2O (to 50 µL). PCR was performed using the described mixtures following a 3-step procedure starting with 1) denaturation at 95 °C for 5 min, 2) a 3-step cycle of denaturation at 95 °C for 30 sec, annealing at 65 °C for 1 min and elongation at 72 °C for 30 sec, repeated 30 times and 3) final elongation at 72 °C for 5 min. Reactions were checked by gel electrophoresis and successful trials were purified using Wizard SV Gel and PCR Clean-up system (Promega, Madison, WI, USA). The two fragments for each mutation were then combined in another PCR using DcS-fw long and DcS-rev long as primers, following the same protocol as before.

Table S10. Primers used for mutational polymerase chain reactions.

Name	Sequence
DcS-fw	ATGACAGTGCGTGCCGTCGA
DcS-rev	TCATGCGCTTCCTGCGGAGG
DcS-fw long	<u>GGCAGCCATATGGCTAGCATGACTGGTGGA</u>ATGACAGTGCGTGCCGTCGA
DcS-rev long	<u>TCTCAGTGGTGGTGGTGGTGGTGCTCGAGT</u>TCATGCGCTTCCTGCGGAGG
H58F-fw	CCCAGTTC**TT**CGCCATGAC
H58F-rev	GTCATGGCG**AA**GAACTGGG
H197F-fw	CTTCGCCGCC**TT**CACCGC
H197F-rev	GCGGTG**AA**GGCGGCGAAG
Y241F-fw	GTCGCCTCCT**T**CGAACGGG
Y241F-rev	CCCGTTCG**A**AGGAGGCGAC

Sequences of homology arms with pYE-Express for homologous recombination are underlined; exchanged nucleotides for mutation are shown in bold.

Homologous recombination in yeast

The gene fragments with the exchanged nucleotides were used for homologous recombination in yeast following a standard PEG-LiOAc-salmon sperm DNA protocol.[11] A 10 mL culture of yeast was grown from a glycerol stock in YPAD overnight and was used to inoculate a total volume of 50 mL. After 4 h the cells were harvested by centrifugation (3.000 x g, 5 min, 4 °C) and the supernatant was discarded. The pellet was resuspended in sterile distilled H_2O (50 mL) and centrifuged again. The cell pellet was resuspended in LiOAc solution (0.1 M, 1 mL) transferred to a microcentrifuge tube and centrifuged (14.000 x g, 1 min). The supernatant was discarded and the pellet was resuspended in LiOAc solution (0.1 M, 800 μL), to get a cell suspension from which 50 μL were used for each transformant. The cells were centrifuged, the supernatant was discarded and carefully PEG 4000 (50% w/v, 240 μL), LiOAc solution (1 M, 36 μL) and salmon sperm carrier DNA (2mg mL^{-1}, 50 μL) were added. On top a mixture of digested pYE-Express vector (15 μL) and the desired gene (19 μL) was added and the mixture was vortexed vigorously for 2 min. The resulting suspension was incubated for 30 min at 30 °C and 42 °C, centrifuged at top speed and resuspended in sdH_2O (600 μL). The suspended cells were spread on SM-URA plates and incubated for 2 days. Plasmid was isolated from the yeast using the Zymoprep Yeast Plasmid Miniprep System (ZymoResearch, Irvine, CA, USA) and the resulting plasmid solutions were used for electroporation into *E. coli* BL21 (DE3) electrocompetent cells (1800 V). LB plates with kanamycin (50 μg mL^{-1}) were grown overnight and single colonies were picked and grown for 6 h before isolating plasmid using the PureYield Plasmid Miniprep System (Promega). The resulting plasmDcS were digested and analyzed by gel electrophoresis, promising transformants were subjected to sequencing to verify their identity, yielding seven *E. coli* strains each harboring a mutant version of DcS.

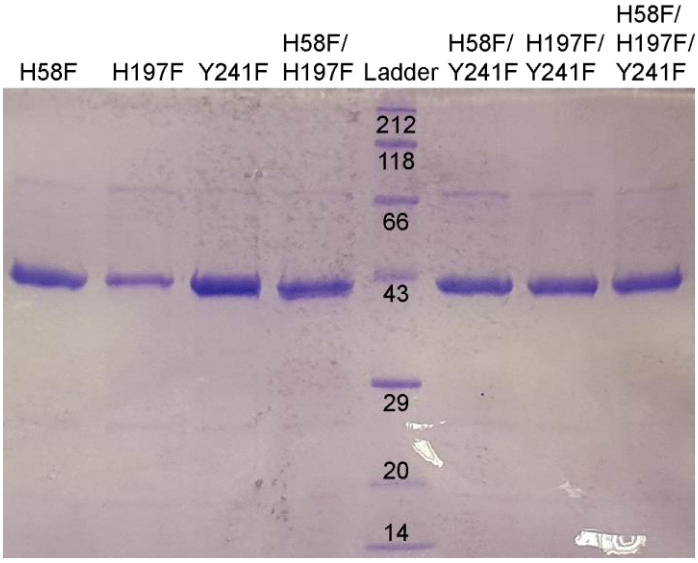

Figure S115. SDS-PAGE gel of heterologously expressed DcS mutants.

Activity tests with dauc-8-en-11-ol synthase mutants

To check the activity of the conceived mutants in comparison to the wild type, 100 mL cultures of each construct were grown and purified as described above. Small scale experiments were performed in triplicates and consisted of protein solution (600 μL, conc: 0.28 mg mL^{-1}), FPP (1 mg), and incubation buffer (600 μL). The mixtures were incubated at 28 °C for 3 h, extracted with *n*-hexane (200 μL) and the extracts were dried with $MgSO_4$ and subjected to GC/MS. The resulting chromatograms were integrated, the average peak area was calculated including a standard deviation and the obtained amounts of dauc-8-en-11-ol were determined relatively to the wild type (Figure 1 of the main text).

For large scale experiments 4 L cultures of the WT or the H197F/Y241F enzyme were grown and purified as described above. Incubations were performed with purified enzyme solution (final concentration 65 µM) with 80 mg (0.19 mmol) of FPP to yield dauc-8-en-11-ol (WT: 4.83 mg, 0.02 mmol, 11%; H197F/Y241F: 18.9 mg, 0.09 mmol, 46%) and with 30 mg (0.07 mmol) 10-Me-FPP to yield **19** (WT: 15%, cf. page S12, H197F/Y241F: 6.86 mg, 0.03 mmol, 42%) and **20** (WT: 4%, cf. page S12, H197F/Y241F: 1.89 mg, 0.01 mmol, 11%).
To check for additional IDI activity each mutant was incubated in an experiment with IPP (1 mg) and FPPS in incubation buffer (total volume 1 mL). Incubations were performed at 28 °C for 3 h, the mixtures were extracted with *n*-hexane (200 µL) and the organic extracts were dried and subjected to GC/MS. None of the mutants showed production of dauc-8-en-11-ol, indicating that the *E. coli* IDI did not co-purify with any of the mutants.

Determination of kinetic parameters

Triplicate incubations with different substrate concentrations were performed for the WT and the H197F/Y241F mutant as described for the activity tests above using an enzyme concentration of 65 µM. Experiments were stopped after 5 minutes by addition of hexane (500 µL, containing 0.5 mg mL^{-1} 1-hexadecanol as a standard). The organic layer was separated, dried and the extracts were subjected to GC analysis. Peak integrals were used to determine the produced amount of dauc-8-en-11-ol according to a reference curve obtained from a dilution series of pure dauc-8-en-11-ol. Plots of the concentration versus time were used to determine the initial velocity v_0 (Figure S116) which was then used for Lineweaver-Burk linearization (Figure S117).[12] The values obtained from FPP concentrations of 10 mM, 5 mM and 0.01 mM were not included since these showed lowered conversion rates by either substrate (10 mM and 5 mM) or product inhibition (0.01 mM). V_{max} was obtained reciprocally from the y-axis section; K_M was obtained reciprocally from the x-axis section. The value for k_{cat} was obtained using $k_{cat} = V_{max}/[E]$ with [E] being the enzyme concentration.

Table S11. Kinetic data for DcS WT and DcS H197F/Y241F.

	V_{max} [mmol L^{-1} s^{-1}]	K_M [mol L^{-1}]	k_{cat} [s^{-1}]	k_{cat}/K_m [L s^{-1} mol^{-1}]
WT	(2.31 ± 0.19) x 10^{-3}	(2.90 ± 0.02) x 10^{-3}	35.56 ± 2.92	1.23 x 10^4
H197F/Y241F	(4.26 ± 0.56) x 10^{-3}	(5.95 ± 0.07) x 10^{-3}	65.48 ± 8.62	1.10 x 10^4

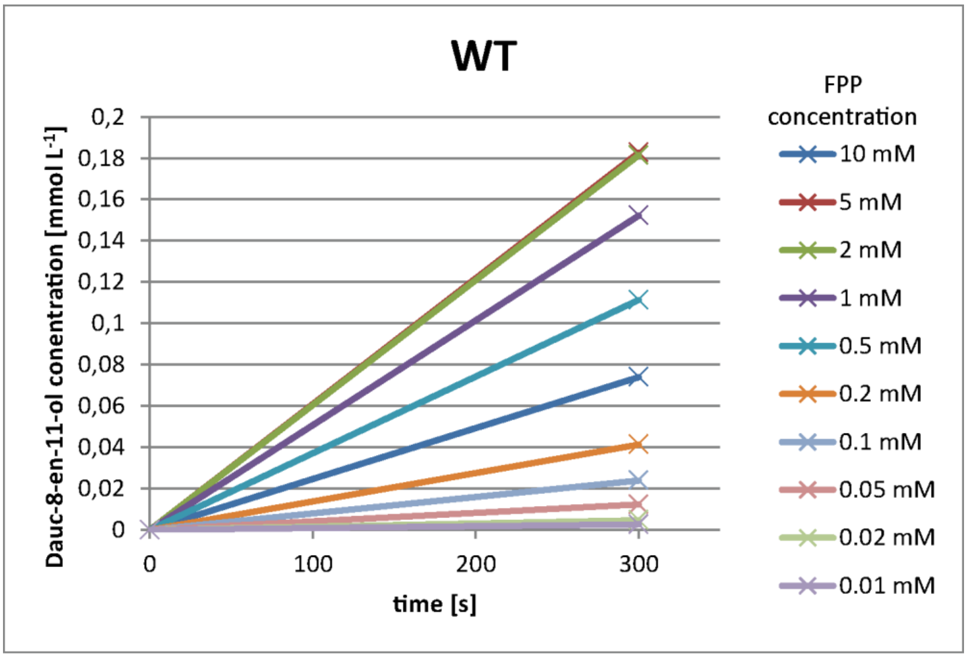

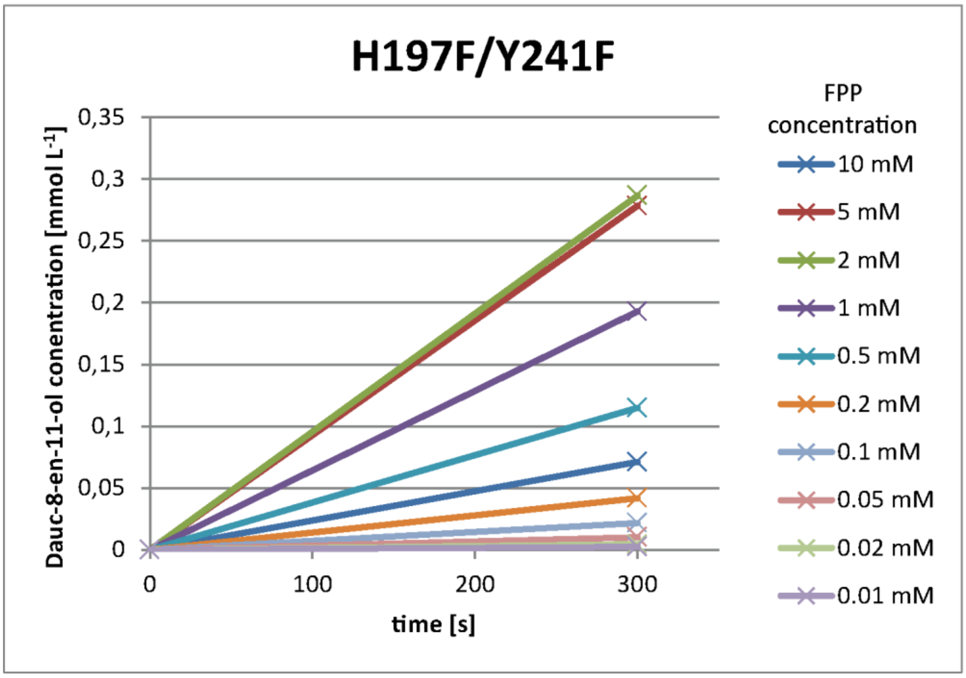

Figure S116. Plot of dauc-8-en-11-ol production versus time, obtained from different starting concentrations of FPP. The slope was used to directly determine v_0.

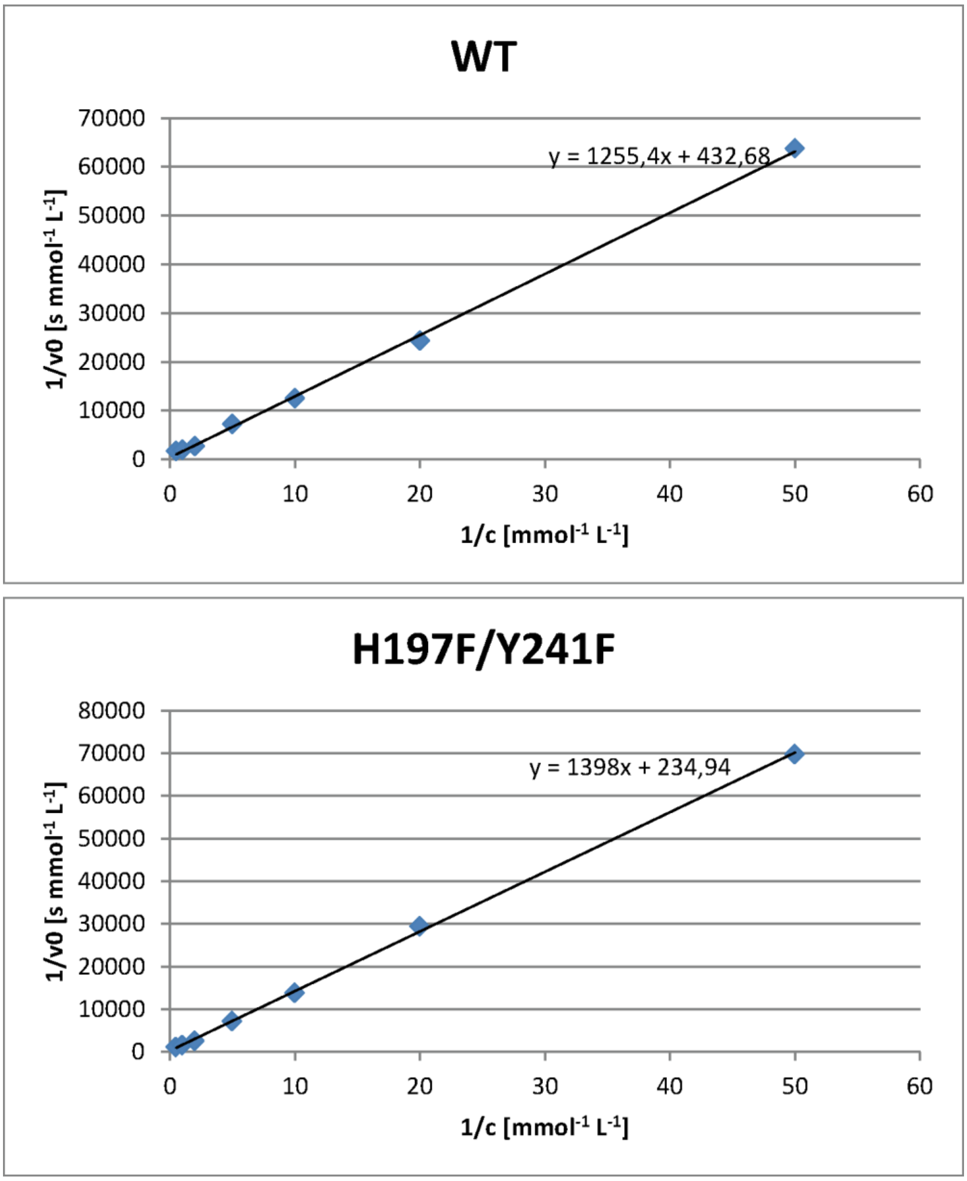

Figure S117. Lineweaver-Burk plots of the initial velocities v_0 versus the concentration of dauc-8-en-11-ol obtained from the kinetic measurements (blue squares). The black line represents the linear regression fit, with the corresponding equation given in the diagram.

References

[1] G. R. Fulmer, A. J. M. Miller, N. H. Sherden, H. E. Gottlieb, A. Nudelman, B. M. Stoltz, J. E. Bercaw, K. I. Goldberg, *Organometallics* **2010**, *29*, 2176–2179.

[2] L. G. Cool, *Phytochemistry* **2001**, *58*, 969.

[3] E. Breitmaier, *Terpenes*; Wiley-VCH: Weinheim, Germany, **2006**; pp 185.

[4] P. Rabe, J. Rinkel, T. A. Klapschinski, L. Barra, J. S. Dickschat, *Org. Biomol. Chem.* **2016**, *14*, 158–164.

[5] P. Rabe, J. Rinkel, B. Nubbemeyer, T. G. Köllner, F. Chen, J. S. Dickschat, *Angew. Chem. Int. Ed.* **2016**, *55*, 15420–15423.

[6] J. Rinkel, J. S. Dickschat, *Org. Lett.* **2019**, *21*, 2426–2429.

[7] L. Lauterbach, J. Rinkel, J. S. Dickschat, *Angew. Chem. Int. Ed.* **2018**, *57*, 8280–8283.

[8] P. Rabe, L. Barra, J. Rinkel, R. Riclea, C. A. Citron, T. A. Klapschinski, A. Janusko, J. S. Dickschat, *Angew. Chem. Int. Ed.* **2015**, *54*, 13448–13451.

[9] W. Kreiser, F. Körner, *Helv. Chim. Acta* **1999**, *82*, 1427.

[10] Y. Hashidoko, S. Tahara, J. Mizutani, *Phytochemistry* **1994**, *35*, 325.

[11] R. Gietz, R. Schiestl, *Nat. Protoc.* **2007**, *2*, 31–34.

[12] H. Lineweaver, D. Burk, *J. Am. Chem. Soc.* **1934**, *56*, 658–666.
